# T-Cell Subsets and Interleukin-10 Levels Are Predictors of Severity and Mortality in COVID-19: A Systematic Review and Meta-Analysis

**DOI:** 10.3389/fmed.2022.852749

**Published:** 2022-04-28

**Authors:** Amal F. Alshammary, Jawaher M. Alsughayyir, Khalid K. Alharbi, Abdulrahman M. Al-Sulaiman, Haifa F. Alshammary, Heba F. Alshammary

**Affiliations:** ^1^Department of Clinical Laboratory Sciences, College of Applied Medical Sciences, King Saud University, Riyadh, Saudi Arabia; ^2^Department of Medical and Molecular Virology, Prince Sultan Military Medical City, Riyadh, Saudi Arabia; ^3^College of Applied Medical Sciences, Riyadh Elm University, Riyadh, Saudi Arabia; ^4^College of Dentistry, Riyadh Elm University, Riyadh, Saudi Arabia

**Keywords:** COVID-19, SARS-CoV-2, coronavirus, interleukin 10, CD4, CD8, IL-10

## Abstract

**Background:**

Many COVID-19 patients reveal a marked decrease in their lymphocyte counts, a condition that translates clinically into immunodepression and is common among these patients. Outcomes for infected patients vary depending on their lymphocytopenia status, especially their T-cell counts. Patients are more likely to recover when lymphocytopenia is resolved. When lymphocytopenia persists, severe complications can develop and often lead to death. Similarly, IL-10 concentration is elevated in severe COVID-19 cases and may be associated with the depression observed in T-cell counts. Accordingly, this systematic review and meta-analysis aims to analyze T-cell subsets and IL-10 levels among COVID-19 patients. Understanding the underlying mechanisms of the immunodepression observed in COVID-19, and its consequences, may enable early identification of disease severity and reduction of overall morbidity and mortality.

**Methods:**

A systematic search was conducted covering PubMed MEDLINE, Scopus, Web of Science, and EBSCO databases for journal articles published from December 1, 2019 to March 14, 2021. In addition, we reviewed bibliographies of relevant reviews and the medRxiv preprint server for eligible studies. Our search covered published studies reporting laboratory parameters for T-cell subsets (CD4/CD8) and IL-10 among confirmed COVID-19 patients. Six authors carried out the process of data screening, extraction, and quality assessment independently. The DerSimonian-Laird random-effect model was performed for this meta-analysis, and the standardized mean difference (SMD) and 95% confidence interval (CI) were calculated for each parameter.

**Results:**

A total of 52 studies from 11 countries across 3 continents were included in this study. Compared with mild and survivor COVID-19 cases, severe and non-survivor cases had lower counts of CD4/CD8 T-cells and higher levels of IL-10.

**Conclusion:**

Our findings reveal that the level of CD4/CD8 T-cells and IL-10 are reliable predictors of severity and mortality in COVID-19 patients. The study protocol is registered with the International Prospective Register of Systematic Reviews (PROSPERO); registration number CRD42020218918.

**Systematic Review Registration:**

https://www.crd.york.ac.uk/prospero/display_record.php?ID=CRD42020218918, identifier: CRD42020218918.

## Introduction

Coronavirus disease 2019 (COVID-19) is a viral infection caused by the severe acute respiratory syndrome coronavirus 2 (SARS-CoV-2), first identified in December 2019 when patients with an unknown type of pneumonia were admitted to Hubei hospital in Wuhan, China (1, 2). The fear of encountering a novel strain from the notorious coronavirus family, of which SARS-CoV-1 and MERS-CoV are members, was thus realized (3). This novel disease spread rapidly from its country of origin to other regions of the world, affecting people in 192 countries and resulting in 280,001,617 confirmed cases and 5,402,083 deaths (4). COVID-19 was declared a global health emergency and a pandemic by the World Health Organization (WHO) in March 2020 (5).

Infection with SARS-CoV-2 does not immediately cause disease, and patients can be divided into four major classes based on their presentation on the clinical spectrum. Patients in the first class are asymptomatic with no clinically reported signs, although anosmia and dysgeusia are common among this group (6–8). The second develop flu-like symptoms with fever, sore throat, and cough (9, 10). Additional signs and symptoms of varying severity are present in the third group, including frequent chest pain, difficulty breathing, and unproductive cough (10, 11). Finally, in the fourth group, life-threatening complications become evident as the disease progresses, and patients begin to exhibit critical signs and symptoms, including pneumonia, acute lung injury (ALI), acute respiratory distress syndrome (ARDS), septic shock, and multiple organ failure (12, 13). Furthermore, the incubation period from infection to the onset of disease varies greatly, ranging from 2 to 14 days (14, 15). The virus's basic reproduction number (R_0_) is estimated to be around 1.4 and 3.8, indicating the potential for a pandemic and recurrent infection within populations (1, 16–18).

It is common for patients with COVID-19 to show a marked decrease in their leukocyte counts, specifically their lymphocyte counts, a condition that translates clinically into immunodepression or immunosuppression (19–25). Outcomes depend on lymphocytopenia status, especially patients' T-cell counts. Recovery is more likely when lymphocytopenia is resolved, and severe complications arising from lymphocytopenia may lead to death (25–29). In addition, studies report elevated levels of IL-10 in severe and non-survivor cases relative to mild or survivor COVID-19 cases (23–26, 30–32). Thus, as an anti-inflammatory cytokine, IL-10 may be responsible for the reduced T-cell counts that lead to immunodepression in COVID-19 patients (33).

It is essential for our understanding of the disease course to identify the influence of T-cell subsets and IL-10 in patients with mild and severe COVID-19, as well as in survivor and non-survivor cases. This systematic review and meta-analysis aimed to analyze T-cell subsets (CD4/CD8) and IL-10 in severe and fatal cases of COVID-19. Our understanding of the mechanisms causing the immunodepression observed in COVID-19—and its consequences—may enable the early identification of disease severity predictors and development of more effective interventions.

## Methods

### Protocol and Registration

**Figure 1:**
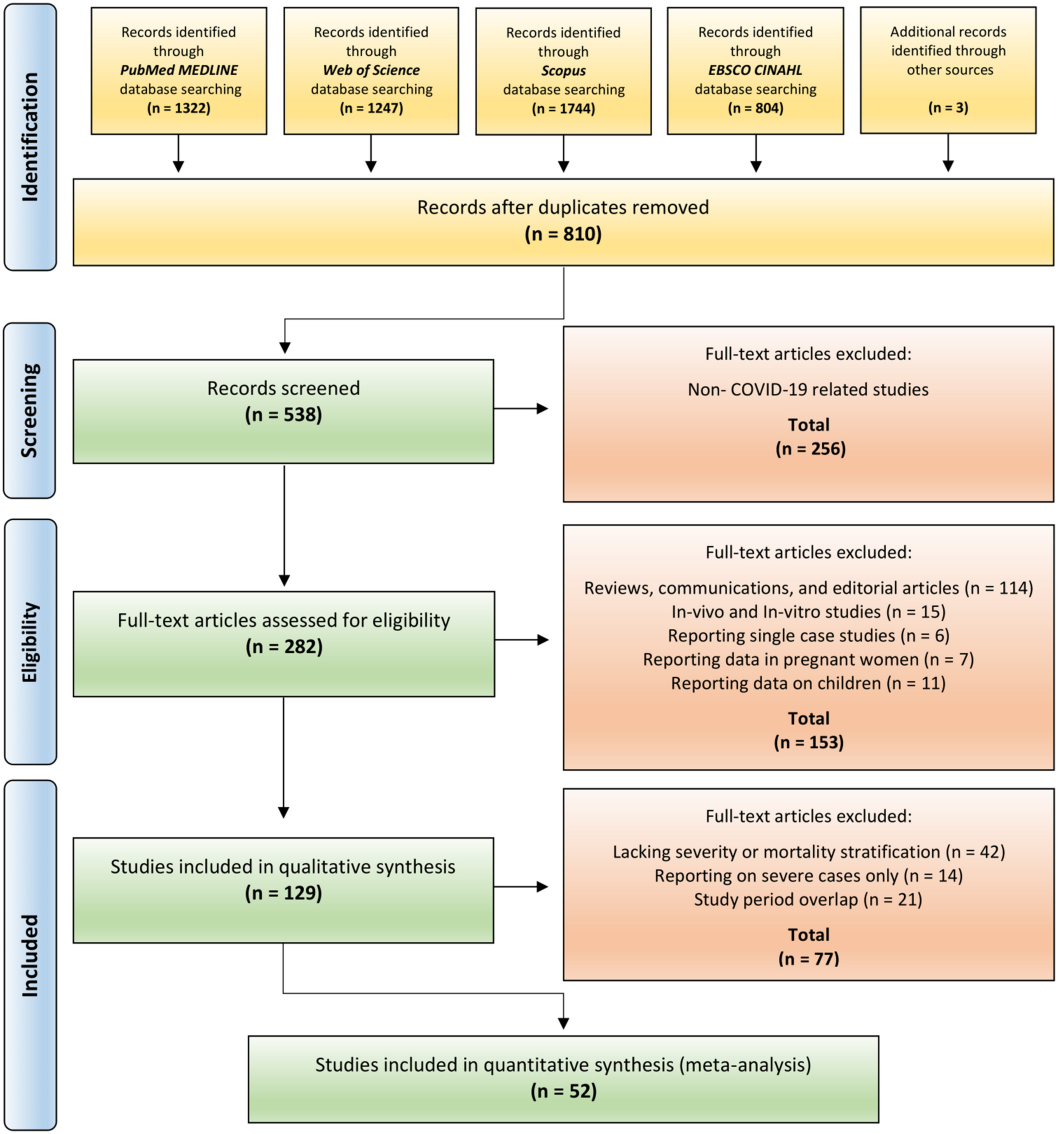
PRISMA flow diagram for T-cell subsets and IL-10 in COVID-19 studies. Figure is adapted from PRISMA flow diagram 2009 (34).

The systematic review and meta-analysis were performed and reported following the guidelines of the Preferred Reporting Items for Systematic Reviews and Meta-Analysis (PRISMA), including the flow diagram (Figure 1) and checklist (Supplementary Table 1.1) (34). Guidelines provided by the Meta-Analyses Of Observational Studies in Epidemiology (MOOSE), including the checklist (Supplementary Table 1.2) (35) were also followed. The study protocol was registered with the International Prospective Register of Systematic Reviews (PROSPERO); registration number CRD42020218918 (36).

### Eligibility Criteria

The PICOS framework—problem/patients/population, intervention/indicator, compare, outcome, and study designs—was used to formulate our research question (37, 38) as follows: Patients: confirmed COVID-19 patients; Indicator: T-cell subsets (CD4/CD8) and IL-10; Comparison: mild vs. severe or survivors vs. non-survivors; Outcome: severity or mortality; Study designs: hospital-based published studies including retrospective, cohort, prospective, descriptive or observational studies, and case series, in which T-cell subsets (CD4/CD8) and IL-10 levels are documented across COVID-19 severity and mortality groups.

Our review also included studies reporting clinical laboratory parameters among confirmed COVID-19 patients, in which clinical diagnosis and classification of patients were carried out following either the WHO guidelines or guidelines published by an official national or regional health governing body. We allowed this criterion to be flexible as the majority of studies only followed WHO guidelines after their recognition of COVID-19 as a pandemic on March 11, 2020 (5, 39). However, studies following national or regional guidelines were included if the criteria for COVID-19 classification was outlined clearly and matches the criteria outlined by the WHO.

The selected studies were hospital-based, and those not reporting on CD4/CD8 T-cells and IL-10, or lacking proper stratification of COVID-19 patients, were excluded. Moreover, to reduce population heterogeneity, we included studies reporting laboratory values for male or female adults and excluded studies reporting on children and pregnant women. However, given that some studies only stratified patients based on sex or provided the mean age of their entire sample, we anticipated a proportion of no more than 10% where studies stated inclusion of children or pregnant women. Furthermore, to eliminate possible differences arising in clinical data obtained from the infection of various SARS-CoV-2 strains, we included studies reported during the first emergence of SARS-CoV-2. Included studies were published in 2020/2021, and data acquisition covered the period from December 2019 to July 2020.

### Information Sources

A systematic search was conducted for published journal articles from databases including PubMed MEDLINE, Scopus, Web of Science, and EBSCO CINAHL. Eligible studies identified from the bibliographies of relevant systematic reviews were also included, and a manual search on the preprint server for health sciences, medRxiv, was used to identify unpublished relevant studies. Reviews, opinion articles, editorial material, communications, conference proceedings and abstracts, *in-vivo*, and *in-vitro* studies were excluded.

### Search Strategy

A systematic search was conducted for journal articles published from December 1, 2019 to March 14, 2021. We used the text words (severe acute respiratory syndrome coronavirus 2), or (SARS-CoV-2), or (coronavirus infection disease 19), or (COVID-19), in conjunction with (T lymphocytes), (T-cells), (CD4), (CD8), (lymphocytes), (interleukin 10), and (IL-10) independently (Supplementary Tables 2.1–2.4). We also applied filters to our search results specifying journal articles and articles published in English.

### Study Selection

Articles obtained from each database were imported into a designated EndNote (Clarivate) folder (40). A new folder was created to combine all articles from each database to eliminate duplicates. Retained articles were then exported into an electronic review manager, Colandr (41), to facilitate title, abstract, and full-text screening using the specified eligibility criteria. While performing full-text screening, we performed a manual search of the full article text, including the Supplementary Material, to confirm T-cell subsets (CD4/CD8) and IL-10 were included in each study. This process was carried out independently by six authors, with articles selected by one author being verified by the others. In any cases of disagreement, the final decision about each study was made by consensus from all authors, and decisions were documented in an Excel spreadsheet (Microsoft Corporation) (42).

### Data Collection Process

Extracted data from the studies were exported into an Excel spreadsheet. For studies with unclear classification of patients, the corresponding authors were contacted for clarification. Authors were also contacted to request data where studies were missing values for T-cell subsets or IL-10. If no response was received, we performed data extraction from study figures using WebPlotDigitizer, a web-based tool for extracting data from figures (Supplementary Table 3) (43).

### Data Items

Data items included author name, publication year, study location (country/city/hospital), study period (duration), study design, assessment of COVID-19 severity (WHO or national/regional health care authority guidelines), timing of proposed classification (on admission or later), sample size (number), sample characteristics (sex, age, underlying conditions), number of cases classified as mild or survivor, number of cases classified as severe or non-survivor, T-cell subsets (CD4/CD8), and IL-10 values stratified by severity or mortality. For laboratory values reported on a continuous scale, the mean and standard deviation (SD), the median with minimum/maximum range, or median and interquartile (IQR) values were extracted. This process was carried out independently by three authors, AFA, JMA, and HFA, and data extracted by one author were verified by the others. Consensus was reached by all authors in any instances of disagreement, and decisions were documented in Excel.

### Assessment of Quality and Risk of Bias in Individual Studies

The Newcastle-Ottawa Scale (NOS) for cohort studies was adopted to evaluate selected studies independently (44). The NOS scale uses a star system (0–9) to judge a study based on three domains: sample selection, sample comparability against controls, and assessment of outcome. A higher NOS score (above 7 stars) reflects better quality and a lower risk of bias. In contrast, lower NOS scores (below 4 stars) reflect lower quality and a higher risk of bias. NOS scores of 4–6 stars indicate moderate quality and risk of bias (Supplementary Table 4). All authors independently rated the included studies. Each author documented their scoring decision and subsequently verified the scoring decision of other authors. The final decision for each study was made by consensus from all authors, and scores were documented in Excel.

### Synthesis of Extracted Data

Extracted data values from the selected studies were reported on a continuous scale. The mean and SD from each study were pooled to compute the effect size or the standardized mean difference (SMD) and 95% confidence intervals (CI). In the case of median and IQR (minimum and maximum) reporting, we computed the mean and SD based on Wan et al.'s formula (45). The mean and SD were calculated using Cochran's formula for median and IQR reporting without minimum and maximum ranges (46). Furthermore, the means and SDs of similar subgroups were combined into a single group using Cochran's formula for combining similar subgroups (46). Formulas used in this study are listed in Supplementary Tables 5.1–5.2.

### Meta-Analysis Summary Measures

Because heterogeneity was expected between individual studies, our meta-analysis used the DerSimonian-Laird inverse variance method for a continuous random-effects model (46, 47). To calculate the SMD, we applied Hedge's g formula to avoid bias in a small study size (48, 49). Statistical analysis of extracted data in this study took place using STATA 17 (StataCorp) (50).

### Synthesis of Meta-Analysis Results

The SMD and 95% CI were computed using the mean, SD, and sample size from each study (47). Meta-analysis results were displayed using forest and Galbraith plots following the same meta-analysis computational measures. Statistical analysis of extracted data, including the construction of the forest and Galbraith plots, also used STATA 17 (StataCorp) (50).

### Assessment of Heterogeneity

Statistical heterogeneity was assessed using *I*^2^ values to measure the degree of inconsistency of collected results produced from the meta-analysis (51, 52). An *I*^2^ cut-off value of 0% indicates no heterogeneity, 25% is low, 50% is moderate, and 75% indicates a high level of heterogeneity (52). The Galbraith plot can also be used to assess heterogeneity visually through detecting outliers located outside the 95% CI shaded region (53). STATA 17 (StataCorp) (50) was used to analyze the *I*^2^ cut-off value and the Galbraith plot.

Between-study variations can be further explored through subgroup analysis to identify the source of heterogeneity if due to a specific study-level covariate. Heterogeneity induced by the relationship between specific-study effect size and study-level covariates can be measured by performing meta-regression, where the adjusted *R*^2^ is used to examine the proportion of between-study variance explained by study-level covariates (54). A bubble plot was also generated following meta-regression as a graphical presentation of the relationship between study-level covariates and the effect size of a specific study (54–56). STATA 17 (StataCorp) was used for the statistical analysis of extracted data, including meta-regression and the construction of the bubble plot (50).

### Small Study Effects and Publication Bias

Standard funnel plots and non-parametric trim and fill funnel plots were constructed to detect possible cumulative bias in the studies. The standard funnel plot is used to detect small study effects and publication bias (57, 58). A symmetrical funnel denotes the absence of bias, while an asymmetrical funnel indicates clear publication bias (46). Publication bias may arise because smaller studies with non-significant results are suppressed from publication, leading to a biased sample. With the nonparametric trim and fill analysis, smaller studies inducing the funnel plot asymmetry are removed to estimate the funnel plot true center. This is followed by the addition of removed studies and their missing counterparts around the center (46, 59, 60). This method enables detection and measurement of the influence of missing studies on the overall effect size (46, 59, 60).

The regression-based Egger test was also performed to statistically assess plot distribution asymmetry: *P* <0.1 indicates publication bias, whereas *P* > 0.1 indicates no publication bias (61, 62). Measurement of publication bias was also performed using Begg's non-parametric rank correlation based on correlating the standardized effect with the variance using Kendall's tau b (63). Begg's test investigates whether Kendall's rank correlation between the effect size and its variance equals zero: a significant correlation indicates bias (*P* < *0*.05), whereas a non-significant correlation indicates the absence of bias (*P* > *0*.05). Standard funnel plots, nonparametric trim and fill funnel plot analysis, regression-based Egger test, and the nonparametric rank correlation (Begg) test were performed using STATA 17 (StataCorp) (50).

### Meta-Analysis Sensitivity Test

The influence of studies harboring a high risk of bias was quantified by performing the Leave-One-Out sensitivity test, where each study is omitted sequentially to measure its influence on the overall combined effect size (64). STATA 17 (StataCorp) was again used for this purpose (50).

## Results

### Study Selection

An outline of the systematic review search results is presented in Figure 1. We identified 5,120 studies by searching four major databases, including PubMed (1,322), Web of Science (1,247), Scopus (1,744), and EBSCO CINAHL (804). In addition, three records were identified from the bibliographies of relevant systematic reviews and the manual search on the preprint server medRxiv. After removal of duplicates, 810 records were screened. Of those, 272 were excluded following title and abstract review. Retained articles were assessed for study eligibility. Of these, 256 records were excluded as they were not COVID-19 related studies. A further 153 papers were excluded as they were reviews, communications, editorial articles, *in-vivo*/*in-vitro* studies or studies reporting on pregnant women or children. Included studies were thoroughly assessed for eligibility criteria. Of those, 77 records were excluded because of lack of stratification based on severity/mortality, for reporting on severe cases only (Supplementary Table 6.1), and because of overlapping study periods (Supplementary Table 6.2). Finally, 52 studies were retained for the synthesis of meta-analysis results (19, 21–25, 27, 30–33, 65–105).

### Study Characteristics

**Figure 2:**
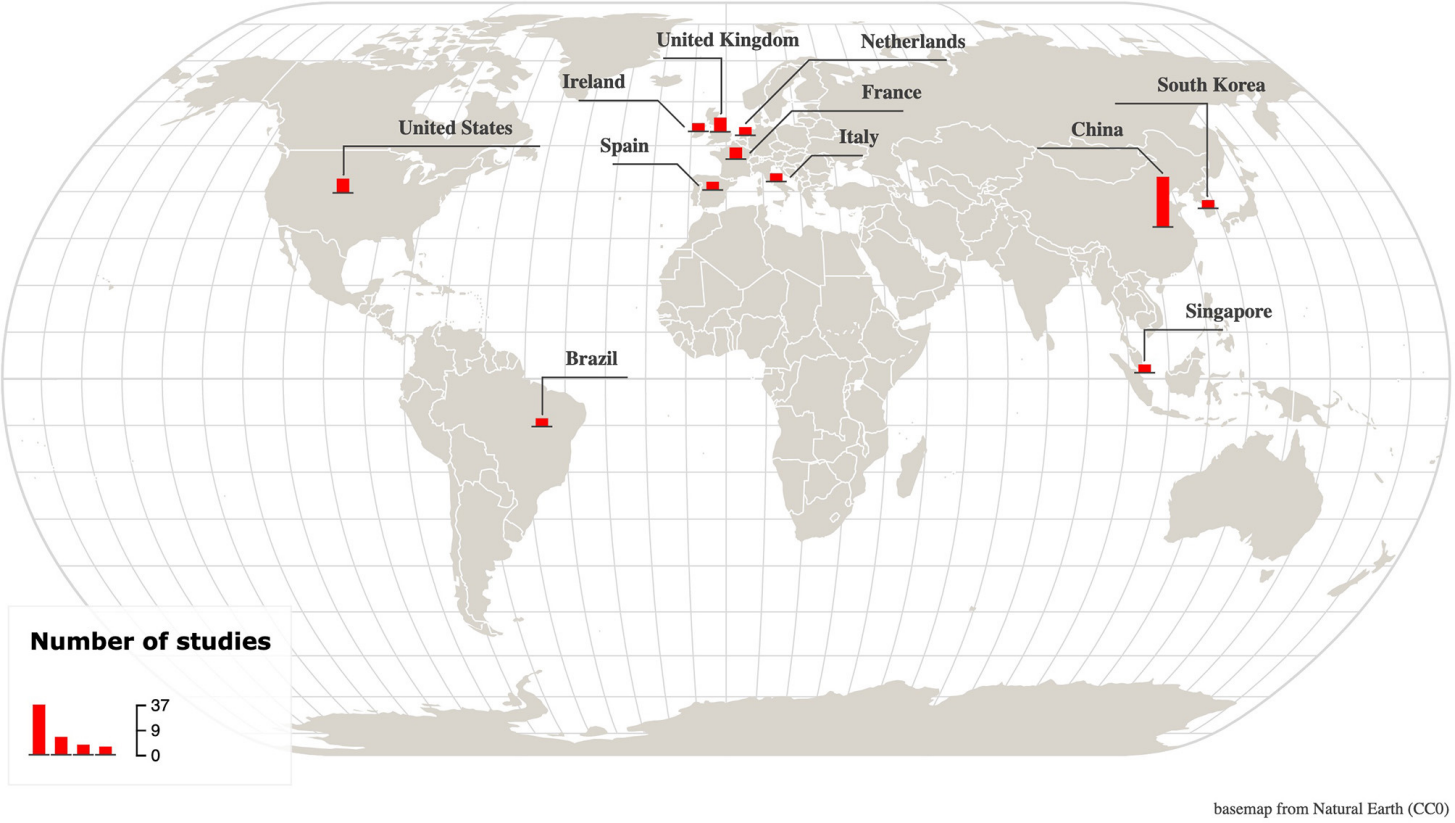
Map showing the location and number of included studies. A total of 52 studies from 11 countries across 3 continents were included. Made with Khartis (106).

The general characteristics of the studies are described in Table 1. They were all hospital-based and included 34 retrospective, 1 retrospective case series, 1 retrospective cohort, 1 prospective, 1 prospective observational, 1 prospective cohort, 10 cohort, and 3 observational cohort studies. The majority of studies were from China with 37 investigations across multiple cities [Wuhan (21), Beijing (4), Hangzhou (3), Guangzhou (2), Taizhou (2), Shanghai (1), Wenzhou (1), Nanchang (1), Nanjing (1), and Jiangsu (1)]. The remaining studies were from the United States [New York (1), New Haven (1), and Cincinnati (1)], United Kingdom [London (2), and Manchester (1)], France [Paris (1), and Creteil (1)], Ireland [Dublin (1)], Italy [Brescia/Monza/Pavia (1)], Spain [Madrid (1)], Netherlands [Breda (1)], Brazil [Curitiba (1)], Korea [Seoul (1)], and Singapore [Singapore (1)] (Figure 2).

**Table 1:** General characteristics of included studies.

First author	Abers, Michael	Hospital	ASST Spedali Civili Brescia; Ospedale San Gerardo; Ospedale S. Matteo
Publication year	2021	Study period	February 25–May 09, 2020
Country	Italy	Study design	Retrospective
City	Brescia; Monza; Pavia	NOS score	6
First author	Azmy, Veronica	Hospital	Not Defined: Tertiary Care Hospital
Publication year	2021	Study period	March 10–31, 2020
Country	United States	Study design	Cohort
City	New Haven, CT	NOS score	7
First author	Cantenys-Molina, S	Hospital	Gregorio Maranon General University Hospital
Publication year	2021	Study period	March 26–May 26, 2020
Country	Spain	Study design	Retrospective
City	Madrid	NOS score	7
First author	Carissimo, Guillaume	Hospital	National Centre for Infectious Diseases
Publication year	2020	Study period	February–April 2020
Country	Singapore	Study design	Observational cohort
City	Singapore	NOS score	8
First author	Chen, Jiaxin	Hospital	Nanjing Medical Hospitals
Publication year	2020	Study period	January 23–March 11, 2020
Country	China	Study design	Retrospective
City	Nanjing	NOS score	6
First author	Chi, Ying	Hospital	Hospital not specified; but the region is specified (Jiangsu province)
Publication year	2020	Study period	Not specified
Country	China	Study design	Cohort
City	Jiangsu	NOS score	6
First author	Deng, Fuxue	Hospital	Sino-French New City Branch of Tongi Hospital
Publication year	2020	Study period	January 30–March 30, 2020
Country	China	Study design	Retrospective
City	Wuhan	NOS score	6
First author	Diao, Bo	Hospital	General Hospital of Central Theater Command or Hanyang Hospital
Publication year	2020	Study period	December 2019–January 2020
Country	China	Study design	Retrospective
City	Wuhan	NOS score	6
First author	Feng, Xiaobo	Hospital	Wuhan Union Hospital
Publication year	2020	Study period	January 23–February 22, 2020
Country	China	Study design	Prospective
City	Wuhan	NOS score	7
First author	Flament, Heloise	Hospital	Bichat or Cochin Hospitals
Publication year	2021	Study period	March 23–October 05, 2020
Country	France	Study design	Cohort
City	Paris	NOS score	9
First author	Gadotti, Ana Carolina	Hospital	Hospital not specified; but the region is specified (Curitiba Parana)
Publication year	2020	Study period	June–July 2020
Country	Brazil	Study design	Prospective cohort
City	Curitiba	NOS score	7
First author	Guan, Jingjing	Hospital	Fifth Affiliated Hospital of Wenzhou Medical University
Publication year	2020	Study period	Not specified; no overlap with hospital location
Country	China	Study design	Cohort
City	Wenzhou	NOS score	6
First author	Han, Huan	Hospital	Renmin Hospital of Wuhan
Publication year	2020	Study period	January–February 2020
Country	China	Study design	Retrospective
City	Wuhan	NOS score	6
First author	He, Bing	Hospital	Renmin Hospital of Wuhan
Publication year	2020	Study period	February 01, 2020
Country	China	Study design	Retrospective
City	Wuhan	NOS score	8
First author	He, Susu	Hospital	Taizhou Public Health Medical Centre
Publication year	2020	Study period	January 17–February 12, 2020
Country	China	Study design	Retrospective
City	Taizhou	NOS score	7
First author	Henry, Brandon Michael	Hospital	University of Cincinnati Medical Centre
Publication year	2021	Study period	April–May 2020
Country	United States	Study design	Retrospective
City	Cincinnati	NOS score	8
First author	Huang, Hong	Hospital	Tongji Hospital
Publication year	2021	Study period	February 07–March 27, 2020
Country	China	Study design	Retrospective
City	Wuhan	NOS score	6
First author	Huang, Wei	Hospital	Wuhan Number 1 Hospital
Publication year	2021	Study period	January 20–March 17, 2020
Country	China	Study design	Retrospective
City	Wuhan	NOS score	7
First author	Hue, Sophie	Hospital	Henri Mondor Hospital Intensive Care Unit (ICU)
Publication year	2020	Study period	March 08–30, 2020
Country	France	Study design	Prospective observational
City	Cretil	NOS score	7
First author	Jin, Xiao-Hong	Hospital	Taizhou Hospital of Zhejiang Province and Taizhou Enze Hospital
Publication year	2020	Study period	January 19–March 11, 2020
Country	China	Study design	Retrospective
City	Taizhou	NOS score	7
First author	Keddie, Stephen	Hospital	University College London Hospital (UCLH)
Publication year	2020	Study period	April 06–May 18, 2020
Country	United Kingdom	Study design	Cohort
City	London	NOS score	7
First author	Kwon, Ji-Soo	Hospital	Asan Medical Centre
Publication year	2020		Chung-Ang University Hospital
Country	Korea		Soonchunhyang University Seoul Hospital
City	Seoul		Inje University Sanggye Paik Hospital
		Study Period	February–April 2020
		Study Design	Prospective
		NOS score	7
First author	Laing, Adam	Hospital	Guy's Hospital and St. Thomas Hospital
Publication year	2020	Study period	March 25–May 14, 2020
Country	United Kingdom	Study design	Observational cohort
City	London	NOS score	8
First author	Li, Chenze	Hospital	Tongji Hospital
Publication year	2020	Study period	January 29–April 01, 2020
Country	China	Study design	Retrospective
City	Wuhan	NOS score	9
First author	Li, Mingyue	Hospital	Wuhan Union Hospital
Publication year	2020	Study period	February 25–27, 2020
Country	China	Study design	Retrospective
City	Wuhan	NOS score	7
First author	Li, Qiang	Hospital	Shanghai Public Health Clinical Centre
Publication year	2020	Study period	January 20–June 23, 2020
Country	China	Study design	Retrospective
City	Shanghai	NOS score	6
First author	Li, Xiaolei	Hospital	The First Affiliated Hospital of Nanchang University
Publication year	2020	Study period	January 24–March 12, 2020
Country	China	Study design	Retrospective
City	Nanchang	NOS score	7
First author	Liao, Baolin	Hospital	Guangzhou Eighth People's Hospital
Publication year	2021	Study period	January 22–April 10, 2020
Country	China	Study design	Retrospective
City	Guangzhou	NOS score	8
First author	Liu, Fangfang	Hospital	Fiifth Medical Centre of PLA General Hospital
Publication year	2020	Study period	January 20–February 23, 2020
Country	China	Study design	Cohort
City	Beijing	NOS score	8
First author	Liu, Jian	Hospital	First Affiliated Hospital
Publication year	2020	Study period	January 22–March 20, 2020
Country	China	Study design	Retrospective
City	Hangzhou	NOS score	7
First author	Liu, Jing	Hospital	Wuhan Union Hospital
Publication year	2020	Study period	January 05–24, 2020
Country	China	Study design	Retrospective
City	Wuhan	NOS score	8
First author	Liu, Lei	Hospital	General Hospital of Central Theater Command of the PLA
Publication year	2020	Study period	February 06–21, 2020
Country	China	Study design	Cohort
City	Beijing	NOS score	7
First author	Liu, Yangli	Hospital	Tongji Hospital
Publication year	2021	Study period	February 09–April 06, 2020
Country	China	Study design	Retrospective
City	Wuhan	NOS score	6
First author	Liu, Xue-Qing	Hospital	Leishenshan Hospital
Publication year	2021	Study period	February 23–April 04, 2020
Country	China	Study design	Retrospective Cohort
City	Wuhan	NOS score	8
First author	Luo, Miao	Hospital	Wuhan Pulmonary Hospital and Tongji Hospital
Publication year	2020	Study period	January 09–March 31, 2020
Country	China	Study design	Retrospective
City	Wuhan	NOS score	9
First author	Mann, Elizabeth	Hospital	Four Hospitals in Greater Manchester Area
Publication year	2020	Study period	March 29–May 07, 2020
Country	United Kingdom	Study design	Observational cohort
City	Manchester	NOS score	7
First author	McElvaney, Oliver	Hospital	Beaumont Hospital
Publication year	2020	Study period	June 2020
Country	Irland	Study design	Cohort
City	Dublin	NOS score	7
First author	Rendeiro, Andre	Hospital	New York Presbyterian Hospital and Lower Manhattan Hospital
Publication year	2020	Study period	April–July 2020
Country	United States	Study design	Retrospective
City	New York	NOS score	9
First author	Schrijver, Benjamin	Hospital	The Peripheral Hospital Amphia Breda
Publication year	2020	Study period	March 24–April 14, 2020
Country	Netherlands	Study design	Retrospective
City	Breda	NOS score	7
First author	Shi, Hongbo	Hospital	Beijing Youan Hospital
Publication year	2020	Study period	Not specified
Country	China	Study design	Retrospective
City	Beijing	NOS score	9
First author	Tan, Mingkai	Hospital	Guangzhou Eighth People's Hospital
Publication year	2020	Study period	January–February 2020
Country	China	Study design	Cohort
City	Guangzhou	NOS score	7
First author	Wang, Zhongliang	Hospital	Wuhan Union Hospital
Publication year	2020	Study period	January 16–29, 2020
Country	China	Study design	Retrospective
City	Wuhan	NOS score	7
First author	Xu, Bo	Hospital	Hubie Provincial Hospital of Traditional Chinese and Western Medicine
Publication year	2020	Study period	December 26, 2019–March 01, 2020
Country	China	Study design	Retrospective
City	Wuhan	NOS score	7
First author	Yang, Ai-Ping	Hospital	Not specified: possibly Zhejiang Xiaoshan Hospital
Publication year	2020	Study period	End date of data collection; February 29, 2020
Country	China	Study design	Retrospective
City	Hangzhou	NOS score	7
First author	Yang, Fan	Hospital	Renmin Hospital
Publication year	2020	Study period	January 01–April 15, 2020
Country	China	Study design	Retrospective
City	Wuhan	NOS score	6
First author	Yi, Ping	Hospital	First Affiliated Hospital of Medical College of Zhejiang University
Publication year	2020	Study period	January 19–February 19, 2020
Country	China	Study design	Retrospective
City	Hangzhou	NOS score	7
First author	Zeng, Hao-Long	Hospital	Tongji Hospital
Publication year	2020	Study period	January 17–February 14, 2020
Country	China	Study design	Retrospective
City	Wuhan	NOS score	6
First author	Zeng, Zhilin	Hospital	Tongji Hospital
Publication year	2020	Study period	January 28–February 12, 2020
Country	China	Study design	Retrospective
City	Wuhan	NOS score	7
First author	Zhang, Bo	Hospital	Tongji Hospital
Publication year	2021	Study period	February–April 2020
Country	China	Study design	Retrospective
City	Wuhan	NOS score	6
First author	Zhang, Jun	Hospital	Tongji Hospital
Publication year	2020	Study period	January 01–February 13, 2020
Country	China	Study design	Retrospective
City	Wuhan	NOS score	7
First author	Zhao, Yan	Hospital	Beijing You'an Hospital
Publication year	2020	Study period	January–March 2020
Country	China	Study design	Cohort
City	Beijing	NOS score	7
First author	Zou, Li	Hospital	Renmin Hospital of Wuhan University
Publication year	2020	Study period	January 16–March 03, 2020
Country	China	Study design	Retrospective
City	Wuhan	NOS score	7

The main characteristics and methods for COVID-19 classification in the selected studies are shown in Supplementary Table 7.1. A total of 15 studies classified COVID-19 severity based on WHO guidelines, while 25 studies followed the Chinese National Health Commission or the Chinese Centre for Disease Control and Prevention, and 12 studies used national or local guidelines. With regard to the clinical laboratory tests performed for COVID-19 patients, 37 studies reported sample acquisition within 24 h of admission, 8 reported sample acquisition 1–7 days post-admission, 1 study reported sample acquisition 7–14 days post-admission, and 6 studies did not specify the sample acquisition time (Supplementary Table 7.2). All studies confirmed COVID-19 infection through RT-PCR. Comorbidities varied, with most studies reporting cardio-cerebrovascular disease, hypertension, diabetes, renal disease, liver disease, chronic obstructive pulmonary disease (COPD), and cancer (Supplementary Table 7.3).

Reporting of COVID-19 severity subgroups (e.g., discharged vs. hospitalized; non-ICU vs. ICU) varied greatly between studies. Therefore, each subgroup was assigned to either mild (including moderate) or severe (including critical) categories based on the clinical presentation described in each study. Similarly, the reporting of COVID-19 mortality contained a slight degree of variability (e.g., cured vs. died; hospitalized vs. deceased). Each subgroup was thus classified into either survivors or non-survivors. Some studies reported more than two groups (up to four), and accordingly, we performed a subgroup combination if the stratification for COVID-19 patients was defined clearly in the study. Thus, our meta-analysis only included studies satisfying this criterion. Supplementary Table 7.4 illustrates the method for subgroup assignment from each study, and Supplementary Table 5.2 shows the formula used for combining subgroups.

The population size and age of COVID-19 patients from the included studies are shown in Supplementary Tables 8.1–8.4. The total number of patients by sex is reported as an approximate number because some studies did not describe gender. The mean population size based on severity was 7,913, including 3,905 males and 3,680 females. Of those, 5,109 (2,357 males/2,493 females) were classified as mild, and 2,804 (1,548 males/1,087 females) were severe. In contrast, the mean population size based on mortality was 3,420, including 1,813 males and 1,508 females. Of those, 2,662 (1,350 males/1,268 females) were sub-grouped as survivors, and 758 (463 males/240 females) as non-survivors. The mean age was 53.03 ± 25.6 for the mild group, and 63.39 ± 22.61 for the severe patients. In contrast, the mean age was 59.36 ± 16.11 for the survivors, and 70.67 ± 12.82 for the non-survivors.

### Risk of Bias Within Studies

Studies were examined for quality to avoid bias, and a score was assigned to each study using the NOS scale for quality assessment of cohort studies, shown in Table 1 and detailed in Supplementary Table 4. The majority of studies received scores indicating high quality: 5 studies received a score of 9, 8 studies received scores of 8, and 26 studies received scores of 7. A total of 13 studies received scores of 6, indicating moderate quality. The sensitivity analysis of our results did not differ markedly following the exclusion of moderate quality studies.

### Meta-Analysis Results of Individual Studies

**Figure 3:**
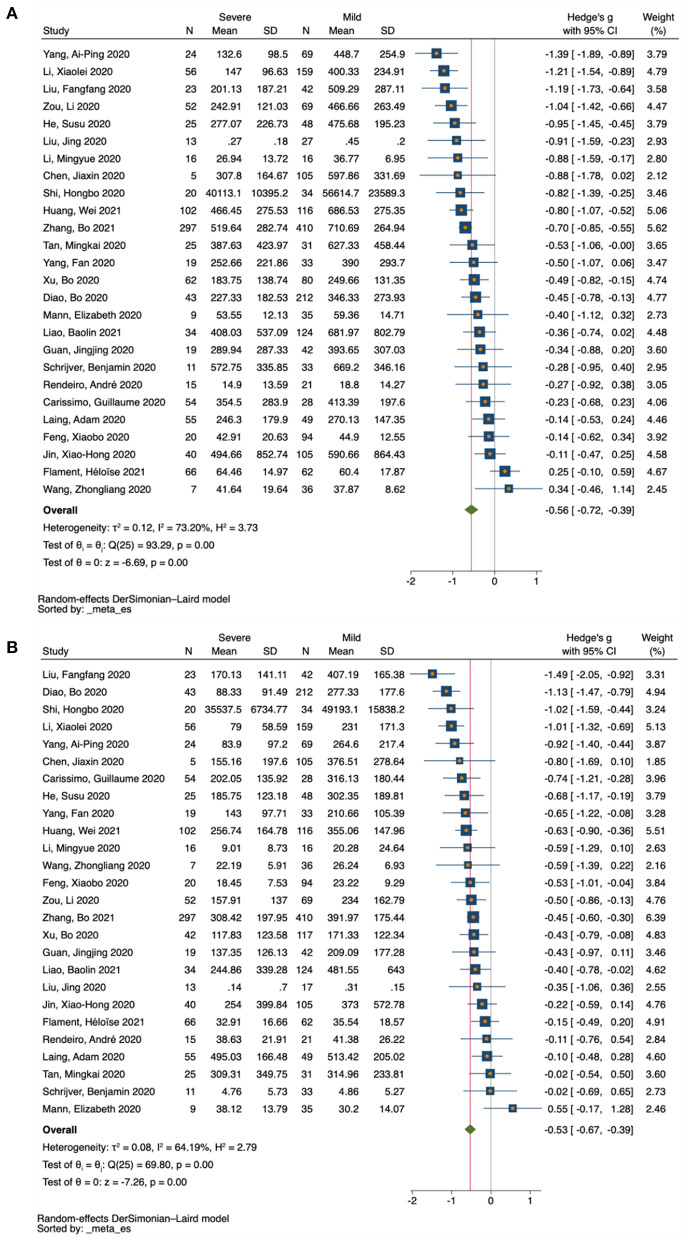
Forest plot of T-cell subsets in COVID-19 severity studies. **(A)** CD4 T-cells in COVID-19 severity studies. **(B)** CD8 T-cells in COVID-19 severity studies. The no-effect line is represented at the value of zero. The diamond symbol represents estimated combined effect size.

**Figure 4:**
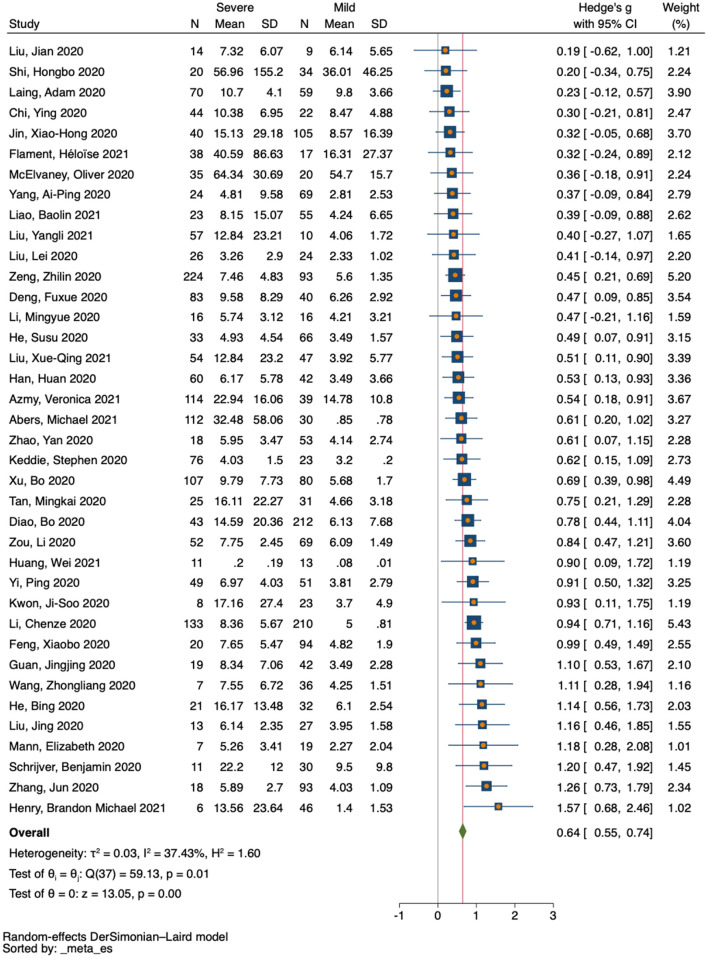
Forest plot of IL-10 in COVID-19 severity studies. The no-effect line is represented at the value of zero. The diamond symbol represents estimated combined effect size.

For the pairwise comparison between mild and severe COVID-19, 24 studies reported decreased counts for CD4 T-cells in the severe cases compared with mild ones (SMD = −1.39 to −0.11), and only 2 studies reported increased counts for CD4 T-cells (SMD = 0.25 to 0.34) (Figure 3A and Supplementary Table 9.1). Similarly, 25 studies reported decreased counts for CD8 T-cells in severe cases relative to mild ones (SMD = −1.49 to 0.02), and only 1 study reported increased counts for CD8 T-cells (SMD = 0.55) (Figure 3B and Supplementary Table 9.2). In contrast, all 38 studies of IL-10 reported increased IL-10 levels in the severe cases relative to mild ones (SMD = 0.19 to 1.57) (Figure 4 and Supplementary Table 9.3).

**Figure 5:**
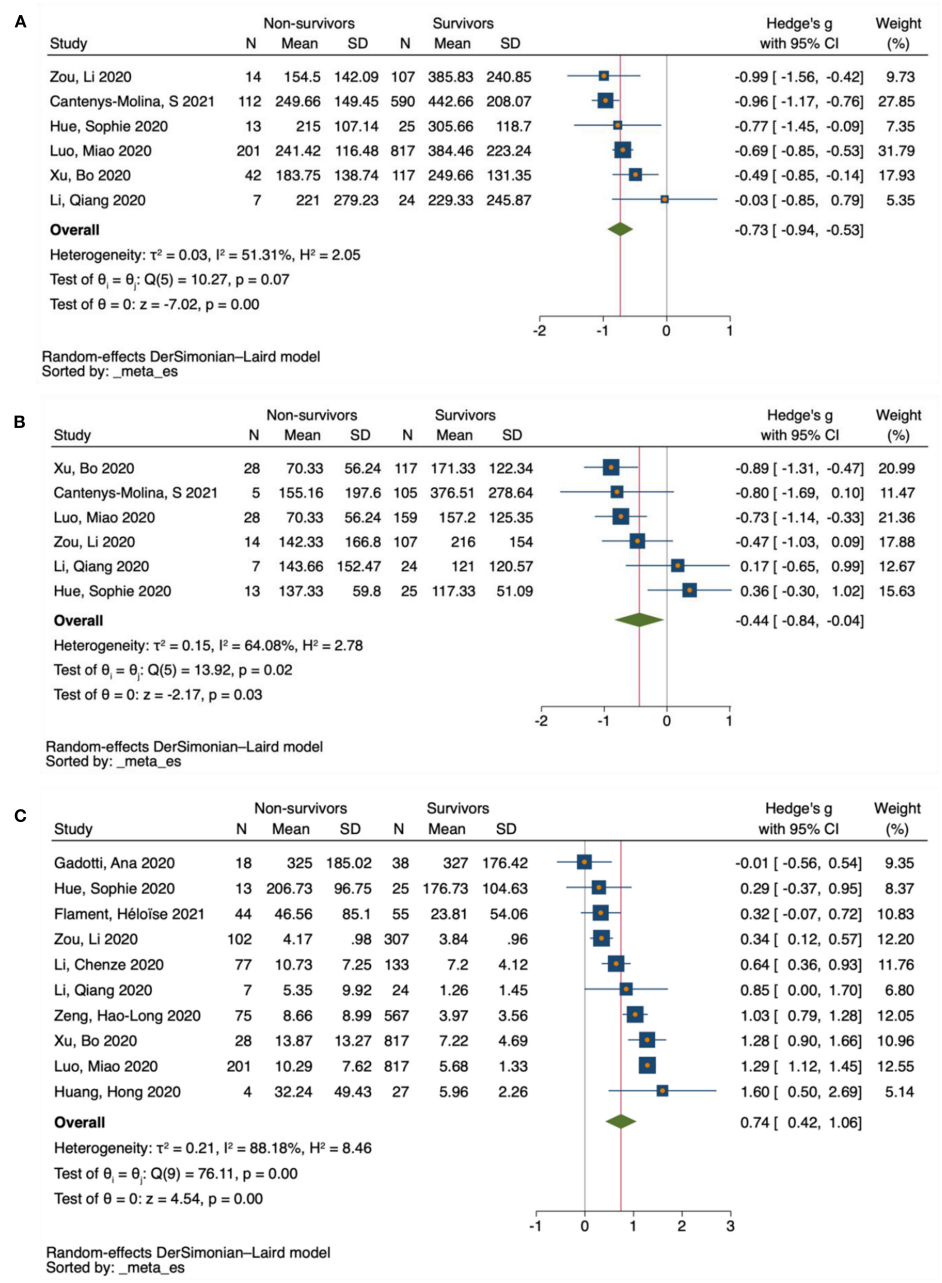
Forest plot of T-cell subsets and IL-10 in COVID-19 mortality studies. **(A)** CD4 T-cells in COVID-19 mortality studies. **(B)** CD8 T-cells in COVID-19 mortality studies. **(C)** IL-10 in COVID-19 mortality studies. The no-effect line is represented at the value of zero. The diamond symbol represents estimated combined effect size.

All included studies for CD4 T-cells (six studies) reported decreased counts in the non-survivors relative to survivors (SMD = −0.99 to −0.03) (Figure 5A and Supplementary Table 9.4), for the pairwise comparison between COVID-19 survivors and non-survivors. Similarly, four studies reported decreased counts for CD8 T-cells among non-survivors compared with survivors (SMD = −0.89 to −0.47), and only two studies reported increased counts for CD8 T-cells (SMD = 0.17 to 0.36) (Figure 5B and Supplementary Table 9.5). In contrast, nine studies reported increased levels of IL-10 in the non-survivors relative to survivors (SMD = 0.29 to 1.6), and only one study reported decreased levels of IL-10 (SMD = −0.01) (Figure 5C and Supplementary Table 9.6).

### Meta-Analysis Results of Combined Studies

#### Forest Plot Meta-Analysis

Based on severity, a total of 26 eligible studies were used for pairwise comparison between mild and severe COVID-19 cases for T-cell subsets. There were 2080 subjects in the mild group vs. 1,112 in the severe group for CD4 T-cells, and 2,107 subjects in the mild group vs. 1,092 in the severe group for CD8 T-cells. Our forest plot meta-analysis revealed an increased effect size of having reduced counts of CD4 and CD8 T-cells in the severe group relative to the mild group (SMD = −0.56, 95% CI = −0.72 to −0.39; SMD = −0.53, 95% CI = −0.67 to −0.39, respectively) (Figures 3A,B). Similarly, a total of 38 eligible studies were used for pairwise comparison between mild and severe COVID-19 cases for IL-10, with a total of 1,981 subjects in the mild group vs. 1,731 in the severe group. The forest plot meta-analysis revealed an increased effect size of having elevated levels of IL-10 in the severe group compared with the mild group (SMD = 0.64, 95% CI = 0.55 to 0.74) (Figure 4).

In terms of mortality, six eligible studies were used for pairwise comparison between survivor and non-survivor COVID-19 cases for T-cell subsets. A total of 1,680 cases were noted in the survivor group vs. 389 in the non-survivor group for CD4 T-cells, and 537 in the survivor group vs. 95 in the non-survivor group for CD8 T-cells. Our forest plot meta-analysis revealed an increased effect size of having reduced CD4 and CD8 T-cell counts in the non-survivor group compared with the survivor group (SMD = −0.73, 95% CI = −0.94 to −0.53, and SMD = −0.44, 95% CI = −0.84 to −0.04, respectively) (Figures 5A,B). Similarly, considering mortality, a total of 10 studies were used for pairwise comparison between survivor and non-survivor COVID-19 cases for IL-10, with a total of 2810 subjects in the survivor group vs. 569 in the non-survivor group. The forest plot meta-analysis revealed an increased effect size of having elevated levels of IL-10 in the non-survivor group compared with the survivor group (SMD = 0.74, 95% CI = 0.42 to 1.06) (Figure 5C).

#### Galbraith Plot Meta-Analysis

**Figure 6:**
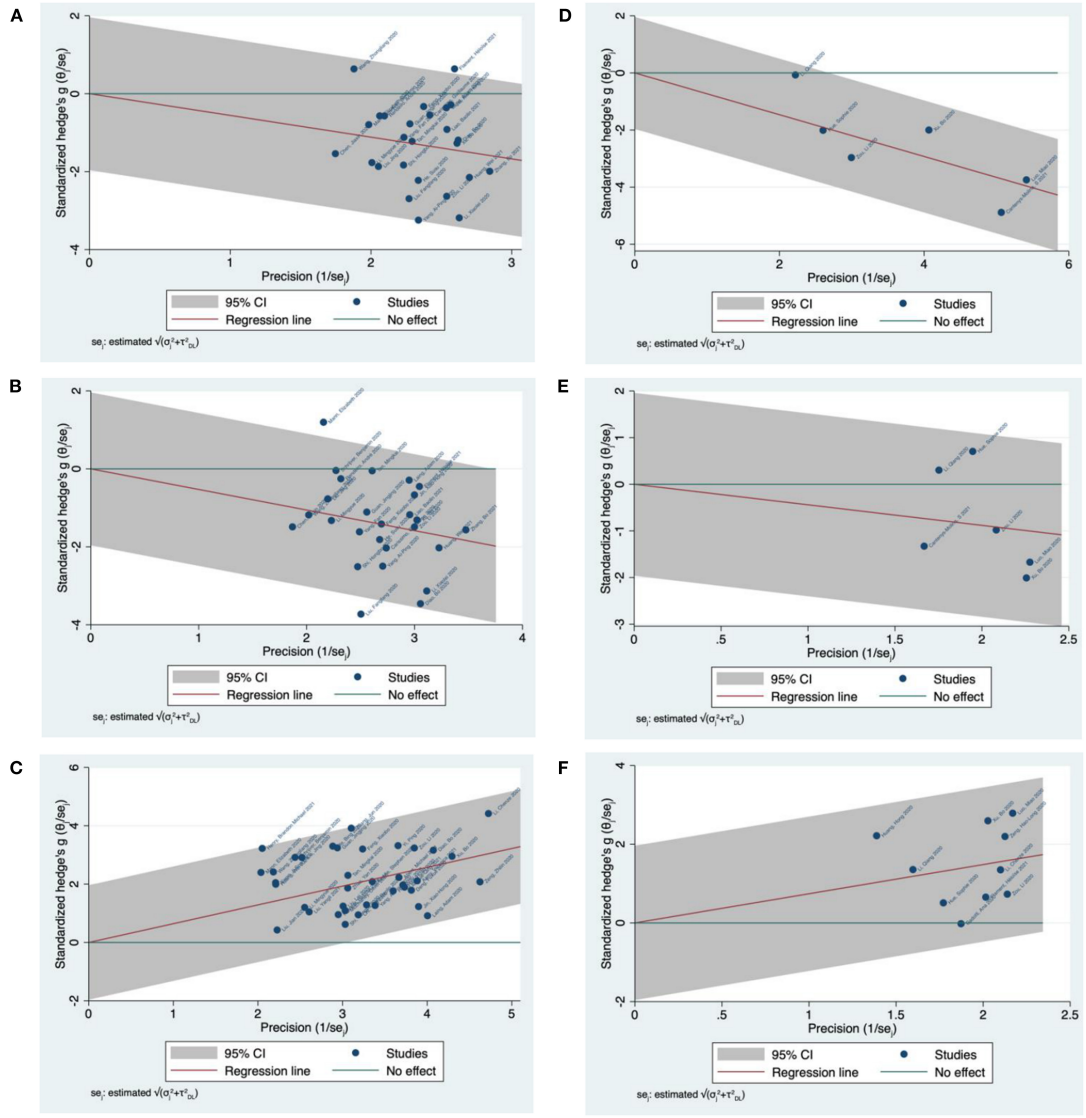
Galbraith plot meta-analysis for CD4/CD8 T-cells and IL-10 in COVID-19 severity and mortality studies. **(A)** CD4 T-cells in COVID-19 severity studies. **(B)** CD8 T-cells in COVID-19 severity studies. **(C)** IL-10 in COVID-19 severity studies. **(D)** CD4 T-cells in COVID-19 mortality studies. **(E)** CD8 T-cells in COVID-19 mortality studies. **(F)** IL-10 in COVID-19 mortality studies.

A total of 26 studies were used for pairwise comparison between mild and severe COVID-19 cases for T-cell subsets. For CD4 T-cell studies, the Galbraith meta-analysis revealed one study located outside the 95% CI region, and one on the 95% CI borderline (Figure 6A). For CD8 T-cell studies, the meta-analysis revealed two studies located outside the 95% CI region (Figure 6B). Similarly, using severity, a total of 38 eligible studies were used for pairwise comparison between mild and severe COVID-19 cases for IL-10. The meta-analysis for IL-10 revealed two studies on the 95% CI borderline (Figure 6C).

Focusing on mortality, six studies were used for pairwise comparison between survivor and non-survivor COVID-19 cases for T-cell subsets. For CD4 and CD8 T-cell studies, all included studies were located within the 95% CI region (Figure 6D) and (Figure 6E), respectively. Similarly, a total of 10 eligible studies were used for pairwise comparison between survivor and non-survivor COVID-19 cases for IL-10. The meta-analysis revealed all included studies were located within the 95% CI region (Figure 6F).

### Assessment of Heterogeneity

The degree of heterogeneity among results from the meta-analysis was assessed using *I*^2^ values. Based on severity, a moderate level of heterogeneity was observed among studies reporting on CD4 T-cells (*P* < *0*.001, *I*^2^ = 73.20%) (Figure 3A), and CD8 T-cells (*P* < *0*.001, *I*^2^ = 64.19%) (Figure 3B). Thus, a low level of heterogeneity was observed among studies reporting on IL-10 (*P* < *0*.001, *I*^2^ = 37.43%) (Figure 4). Similarly, based on mortality, a moderate level of heterogeneity was observed in studies reporting on CD4 T-cells (*P* < *0*.001, I^2^ = 51.31%) (Figure 5A), and CD8 T-cells (*P* < *0*.001, *I*^2^ = 64.08%) (Figure 5B). However, a high level of heterogeneity was observed for studies reporting on IL-10 (*P* < *0*.001, *I*^2^ = 88.18%) (Figure 5C).

#### Subgroup Analysis

A subgroup analysis and meta-regression test were used to assess the source of heterogeneity using study-level covariates stratifying studies based on location (city, country, and continent), study design (retrospective, prospective, or cohort), classification protocol (WHO NCP, CDC, National Health Commission of China, or National/Regional Guidelines), sample acquisition time (at admission or later), population influence (total number of male/female, and mean age of patients), and laboratory influence (test procedure) followed by construction of a bubble plot. Moreover, the covariate of continent was stratified as America, Asia, and Europe. Thus, studies published in America and Europe were sometimes combined due to insufficient studies in each group. The covariate city was stratified as Wuhan or other cities because most studies were based in Wuhan. Similarly, the covariate country was stratified as China or other countries because most studies were based in China. In addition, the total number of male/female patients in each study was indicated as (more or <50), and the mean age of patients was indicated as (more or <50 or 60). We found city, continent, classification protocol, total number of male/female, and mean age of patients were study-level covariates successful in reducing the heterogeneity observed in included studies (in varying degrees based on the parameter being tested).

Based on severity, between-study variability was detected and measured for CD4/CD8 T-cell and IL-10 studies. For CD4 T-cells, the stratification of studies based on the continent covariate revealed a greater heterogeneity among studies conducted in Asia (*P* < *0*.001, *I*^2^ = 66.76%) than for those in Europe and the Americas (*P* < *0*.001, *I*^2^ = 16.79%) (Supplementary Figure 10.3A). Furthermore, the meta-regression analysis revealed that 25.15% of heterogeneity could be explained by the selected covariate (continent) (Supplementary Figure 10.3B) and the effect size increase in studies conducted in Europe or the Americas compared with Asia (Supplementary Figure 10.3C). Similarly, the stratification of CD8 T-cell studies based on the continent covariate revealed a higher heterogeneity among studies conducted in Asia (*P* < *0*.001, *I*^2^ = 56.01%) than for studies conducted in Europe and the Americas (*P* < *0*.001, *I*^2^ = 0.00%) (Supplementary Figure 10.13A). The meta-regression analysis indicated that 42.38% of the heterogeneity could be explained by the selected covariate (continent) (Supplementary Figure 10.13B) and the effect size increase in studies conducted in Europe or the Americas compared with Asia (Supplementary Figure 10.13C). However, the stratification of IL-10 studies, based on the covariate city, revealed greater heterogeneity among studies conducted in Wuhan (*P* < *0*.001, *I*^2^ = 36.10%) compared with other cities (*P* < *0*.001, *I*^2^ = 26.39%) (Supplementary Figure 10.21A). In addition, the meta-regression analysis showed that 10.91% of heterogeneity could be explained by the selected covariate (city) (Supplementary Figure 10.21B) and the effect size increase in studies conducted in Wuhan compared with other cities (Supplementary Figure 10.21C).

The total number of included studies was insufficient to perform a subgroup analysis focusing on mortality. However, between-study variability was measured for CD4/CD8 T-cell and IL-10 studies. For CD4 T-cells, the stratification of studies based on the covariate (classification protocol) revealed more heterogeneity among studies conducted following the WHO NCP or CDC guidelines (*P* < *0*.001, I^2^ = 37.41%) in comparison to those following national or local guidelines (*P* < *0*.001, *I*^2^ = 0.00%) (Supplementary Figure 10.35A). The analysis revealed that 100% of heterogeneity could be explained by the selected covariate (classification protocol) (Supplementary Figure 10.35B) and the effect size increase in studies conducted following WHO NCP or CDC guidelines compared with those following national or local guidelines (Supplementary Figure 10.35C). Stratification of CD8 T-cell studies based on the covariate (total number of male/female patients) revealed homogeneity among studies conducted in studies when the total number of male/female patients is (<50) (*P* < *0*.001, *I*^2^ = 0.00%) or (>50) (*P* < *0*.001, *I*^2^ = 0.00%) (Supplementary Figures 10.47A, 10.48A). Furthermore, the meta-regression analysis revealed that 100% of the heterogeneity was explained by the selected covariate (total number of male/female patients) (Supplementary Figures 10.47B, 10.48B), and the effect size decrease in studies when the number of male/female patients is (>50) compared with those (<50) (Supplementary Figures 10.47C, 10.48C). Moreover, the stratification of IL-10 studies, based on the covariate (mean age), revealed greater heterogeneity among studies when the mean age of patients is (>60) (*P* < *0*.001, *I*^2^ = 82.18%) compared with those (<60) (*P*<*0*.001, *I*^2^ = 0.00%) (Supplementary Figure 10.59A). The analysis revealed that 44.16% of heterogeneity could be explained by the selected covariate (mean age) (Supplementary Figure 10.59B) and the effect size decrease in studies when the mean age of patients is (>60) compared with those (<60) (Supplementary Figure 10.59C).

### Small Study Effects and Publication Bias

**Figure 7:**
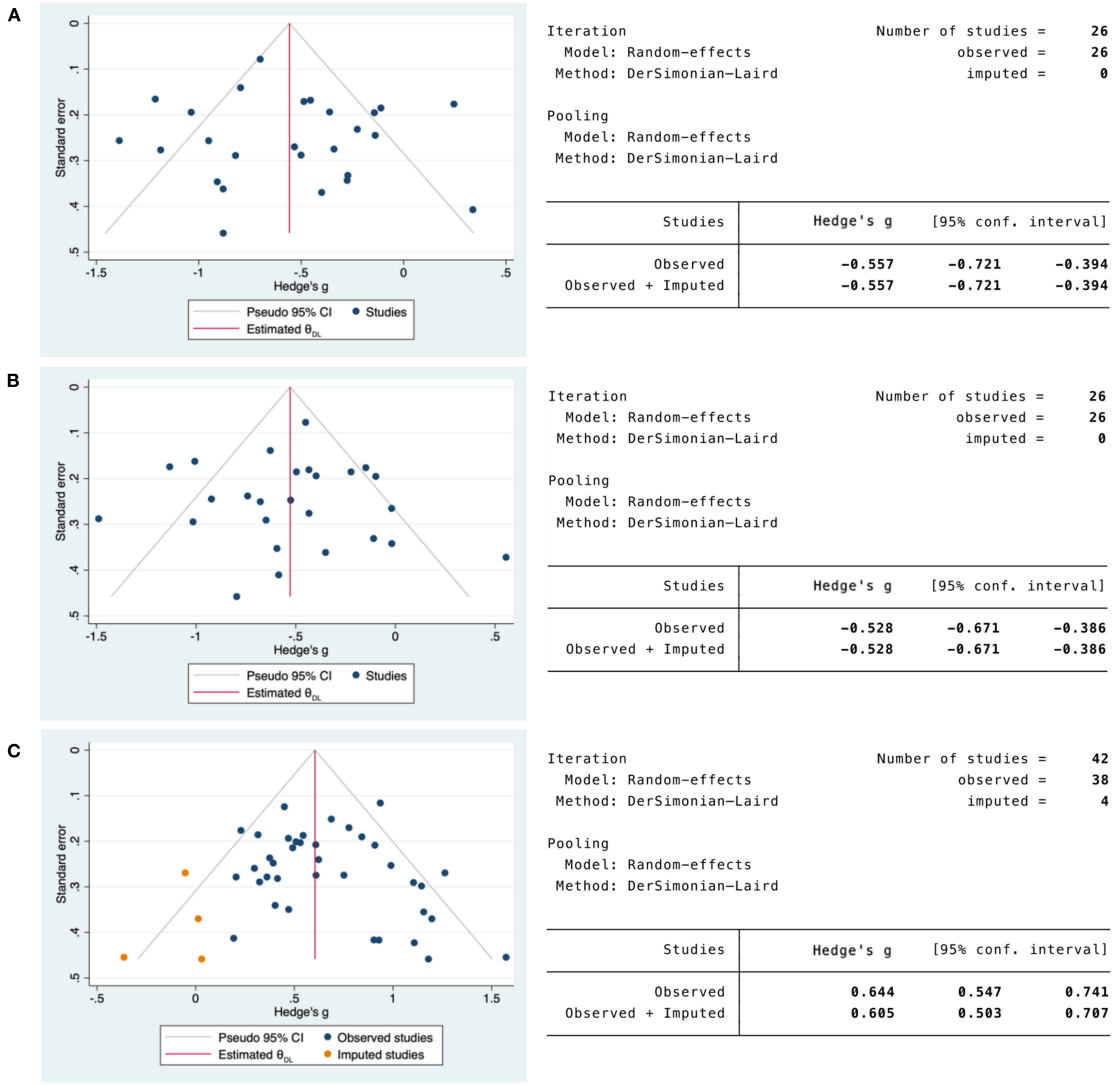
Non-parametric trim and fill funnel plot analysis of publication bias for CD4/CD8 T-cells and IL-10 in COVID-19 severity studies. **(A)** CD4 T-cells in COVID-19 severity studies. **(B)** CD8 T-cells in COVID-19 severity studies. **(C)** IL-10 in COVID-19 severity studies. Observed studies (blue). Imputed studies (orange).

Where heterogeneity was due to small study effects or publication bias, funnel plots were constructed to compare COVID-19 severity and mortality studies. In addition, the regression-based Egger test (using study-level covariates) and the non-parametric rank correlation (Begg) test were performed to statistically evaluate the funnel plot symmetry. Visual inspection of the funnel plot for CD4 T-cell severity studies suggested symmetrical distribution among included studies (Supplementary Figure 11.1A). The regression-based Egger's test with study-level covariates showed no evidence of small study effects or publication bias (*P* > 0.1) (Supplementary Figure 11.1B), a finding supported by the non-parametric rank correlation Begg's test (*P* > *0*.05) (Supplementary Figure 11.1C). Funnel plot symmetry was also confirmed by the non-parametric trim and fill analysis that revealed no missing studies (Figure 7A). Similarly, visual inspection of the funnel plot for CD8 T-cell severity studies suggested symmetrical distribution among the studies (Supplementary Figure 11.2A). The regression-based Egger's test with study-level covariates indicated no small study effects or publication bias (*P* > 0.1) (Supplementary Figure 11.2B), along with the non-parametric rank correlation Begg's test (*P* > 0.05) (Supplementary Figure 11.2C). Funnel plot symmetry was also confirmed by the non-parametric trim and fill analysis that revealed no missing studies (Figure 7B). In contrast, visual inspection of the funnel plot for IL-10 severity studies suggested asymmetrical distribution among included studies (Supplementary Figure 11.3A), and the regression-based Egger's test with study-level covariates indicated small study effects or publication bias (*P* < .1) (Supplementary Figure 11.3B). Although the non-parametric rank correlation Begg's test revealed no evidence of small study effects or publication bias (*P* > *0*.05) (Supplementary Figure 11.3C), funnel plot asymmetry was confirmed by the non-parametric trim and fill analysis that revealed four missing studies. However, the overall effect size did not vary greatly with the addition of the four imputed studies (observed SMD = 0.644, 95% CI = 0.547 to 0.741 and observed + imputed SMD = 0.605, 95% = 0.503 to 0.707) (Figure 7C).

**Figure 8:**
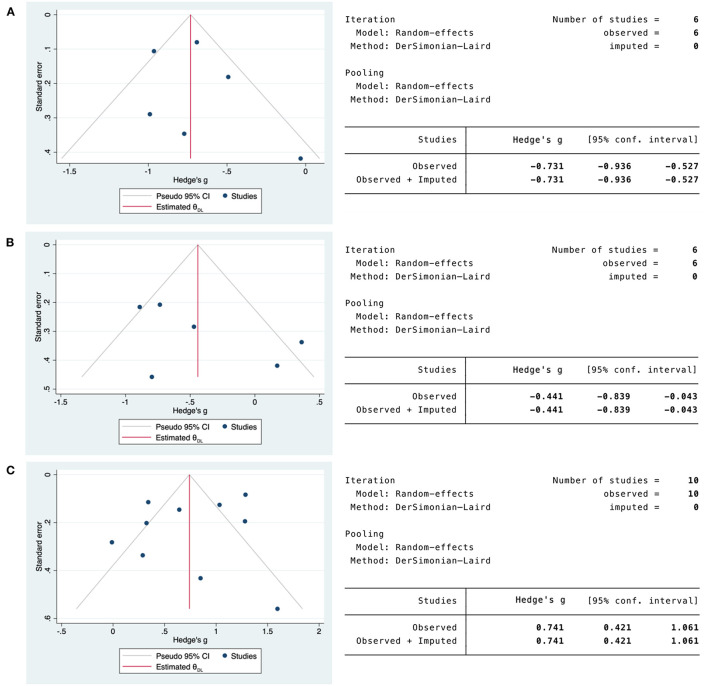
Non-parametric trim and fill funnel plot analysis of publication bias for CD4/CD8 T-cells and IL-10 in COVID-19 mortality studies. **(A)** CD4 T-cells in COVID-19 mortality studies. **(B)** CD8 T-cells in COVID-19 mortality studies. **(C)** IL-10 in COVID-19 mortality studies. Observed studies (blue). Imputed studies (orange).

Visual inspection of the funnel plot for CD4/CD8 T-cell and IL-10 mortality studies suggested symmetrical distribution among included studies (Supplementary Figures 11.4A, 11.5A, 11.6A). The regression-based Egger's test with study-level covariates showed no evidence of small study effects or publication bias (*P* > 0.1) (Supplementary Figures 11.4B, 11.5B, 11.6B), a finding supported by the nonparametric rank correlation Begg's test (*P* > 0.05) (Supplementary Figures 11.4C, 11.5C, 11.6C). Funnel plot symmetry for CD4/CD8 T-cell and IL-10 mortality studies was also confirmed by the non-parametric trim and fill analysis that revealed no missing studies (Figures 8A–C).

### Meta-Analysis Sensitivity Test

We conducted a Leave-One-Out sensitivity analysis to compare mild vs. severe cases and for comparison between survivors vs. non-survivors. Results from each study in the severity group contributed similarly to the overall results for the combined effect size [SMD = −0.52 to −0.58 for CD4 T-cell studies (Supplementary Figure 12.1), SMD = −0.50 to −0.56 for CD8 T-cell studies (Supplementary Figure 12.2), and SMD = 0.65 to 0.63 for IL-10 studies (Supplementary Figure 12.3)]. Results from each study in the mortality group contributed similarly to the overall result of the combined effect size [SMD = −0.70 to −0.77 for CD4 T-cell studies (Supplementary Figure 12.4), (SMD = −0.32 to −0.63 for CD8 T-cell studies (Supplementary Figure 12.5), and SMD = 0.82 to 0.69 for IL-10 studies (Supplementary Figure 12.6)].

## Discussion

The SARS-CoV-2 infection continues to spread globally, with millions of lives lost and many individuals at high risk of developing serious or life-threatening complications. Although vaccines can effectively reduce risks associated with SARS-CoV-2, new variants have been identified worldwide that may challenge the effectiveness of current vaccines (107–109). In addition, vaccination may not be a feasible option for immunocompromised patients or in cases where infection precedes vaccination. Therefore, examining the underlying pathophysiological manifestations of SARS-CoV-2 infection within its human host, as reflected in the resulting clinical picture and laboratory parameters, is paramount to our ability to prevent disease progression or mortality in the present time and the future.

Lymphocytopenia is a common outcome among COVID-19 patients, observed mainly as decreased counts of CD4 and CD8 T-cells (26, 96, 110–112). Several studies have shown that patients with recovered T-cell counts experience improvement in their health status, while the condition of patients with non-recovered T-cell counts worsens and sometimes leads to death (110, 113). Thus, the main objective of this review was to assess the magnitude of this immunodepression and its role in enabling the early identification of mild and severe COVID-19 cases and potential survivor and non-survivor cases. Elucidating the influence of SARS-CoV-2 infection on host immunity among COVID-19 cases allows for a better understanding of the disease course and intervention needed during the early phase following infection.

Our meta-analysis shows that COVID-19 alters the status of human host immunity by driving the depression of adaptive immunity, manifesting in the lower counts of CD4 and CD8 T-cells more evident in severe and non-survivor cases. This observation aligns with recent systematic reviews and meta-analyses in which T-cell subsets were also reduced (114–121). The immune system's ability to overcome the disease by reversing immunosuppression is critical, considering that our systematic review and meta-analysis have shown that mild cases and survivors exhibit a slight depression or possibly recovered T-cell counts compared with severe cases or non-survivors. T-cells play a crucial role in adaptive immunity, essential for fighting infection. Several lines of evidence reveal that in COVID-19, innate immunity is mainly responsible for inducing the inflammation observed following infection with SARS-CoV-2. This translates into a cytokine storm prevalent among patients diagnosed with COVID-19. However, adaptive immunity is essential for virus recognition and immunity against subsequent infection. The fact that this arm of the immune system is paralyzed following infection with SARS-CoV-2 indicates that host ability to produce specific immunity against the virus is compromised. Furthermore, the initiation of the adaptive immune response requires the CD4 T-cells to recognize viral-associated antigens necessary to initiate recognition through antigen-presenting cells (APCs) (122). Similarly, viral clearance through cytotoxic CD8 T-cells is critical following viral infections (123, 124). However, if the very tool that enables antigen recognition and viral clearance is downregulated, the whole arm of adaptive immunity may be affected. This clinical picture is noticed in COVID-19 patients, as described by the cumulative studies in our meta-analysis showing that following infection with SARS-CoV-2, CD4, and CD8 T-cell counts are reduced.

Several mechanisms have been put forward to explain the reduced T-cell counts observed in COVID-19 patients, which may be due to direct infection of T-cells with SARS-CoV-2, impaired T-cell proliferation and activation, T-cell exhaustion and apoptosis, or as a consequence of excessive production of inflammatory cytokines (33, 93, 125–129). Moreover, several cytokines have been found to inversely correlate with T-cell counts in COVID-19, including IL-10, suggesting a possible involvement in T-cell reduction following infection with SARS-CoV-2 (33). This aligns with the fact that during viral infections, the secretion of IL-10 has been found to inhibit the expression of major histocompatibility class II (MHC II) and co-stimulatory molecules CD80 and CD86 on APCs. This restricts CD4 T-cells' ability to recognize viral antigens, and as a consequence CD8 T-cells lose the activation signals initiated by CD4 T-cells (124, 130, 131).

In addition, studies in our meta-analysis revealed that IL-10 concentration is increased following SARS-CoV-2 infection, suggesting that IL-10 may function as a predictor of patients' clinical status and survival. Our meta-analysis results show that IL-10 is increased in severe cases and non-survivors relative to mild or survivor COVID-19 cases. This observation has also been recorded in similar systematic reviews and meta-analysis studies conducted with COVID-19 confirmed patients. Their IL-10 levels were also increased following infection with SARS-CoV-2 (115–117, 119, 120, 132, 133). IL-10 downregulates an exacerbated immune response or excessive inflammation as an anti-inflammatory cytokine (134, 135). However, in COVID-19, IL-10 is associated with severe cases and mortality because the cytokine storm intensifies. Thus, as a counterbalancing cytokine, IL-10 should facilitate the recovery from the cytokine storm initiated primarily through the secretion of IL-6, given that several lines of evidence indicate the involvement of IL-6 in the cytokine storm following SARS-CoV-2 infection (25, 136). Interestingly, IL-6 and IL-10 are increased among severe and non-survivor COVID-19 cases (25). This may indicate that in COVID-19, IL-10 enhances the pro-inflammatory environment. Further, IL-10 inhibits the activation of adaptive immunity by suppressing the function of antigen recognition on APCs, pointing to the possible role of IL-10 as the main driver of the immunosuppression observed in patients with COVID-19. However, more research is needed to verify this hypothesis.

## Conclusion

Our systematic review and meta-analysis revealed that CD4 and CD8 T-cells are reduced in COVID-19 patients. The studies show that this reduction is more evident in the severe and non-survivor cases than in mild or survivor cases. In addition, our meta-analysis indicated that the IL-10 concentration increases, especially in the severe and non-survivor cases relative to mild or survivor cases. We conclude that the immunodepression observed following infection with SARS-CoV-2 is possibly driven by IL-10. Moreover, evidence demonstrates that the levels of CD4 and CD8 T-cells, and IL-10, are associated with severity and mortality, suggesting the importance of including such critical parameters in the routine diagnostic panel for COVID-19 patients as predictors of severity and mortality following SARS-CoV-2 infection.

### Study Limitations

This systematic review and meta-analysis investigated the levels of T-cell subsets and IL-10 in COVID-19 patients. Conclusive data are still emerging because of the relatively short time since the emergence of SARS-CoV-2. Studies that described T-cell subsets together with IL-10 were also scarce, and lymphocyte counts were sometimes reported without dissecting the subsets. These limitations made it challenging to obtain a focused larger study size. In addition, including studies published only in the English language may also have limited our results. However, we believe that the quality of our search and the data obtained could assist in understanding the COVID-19 clinical picture, consistent with the goal of collating reliable sources to aid the scientific community and health care providers in their combat against this novel disease. Nonetheless, further studies are needed to support our conclusions and findings.

## Data Availability Statement

The original contributions presented in the study are included in the article/Supplementary Material, further inquiries can be directed to the corresponding author.

## Author Contributions

AA was involved in study conceptualization, literature search, data curation, analysis and interpretation of the data, generation of the figures, and in drafting and revising the manuscript. JA and HaA were involved in the literature search, data curation, analysis and interpretation of the data, and in revising the manuscript. KA and AA-S were involved in the data curation, analysis and interpretation of the data, and in revising the manuscript. HeA was involved in the data curation, analysis and interpretation of the data, generation of the figures, and in revising the final version of the manuscript. All authors have approved the final version of the manuscript.

## Funding

The research project was supported by a grant from Research Centre of the Female Scientific and Medical Colleges, Deanship of Scientific Research, King Saud University.

## Conflict of Interest

The authors declare that the research was conducted in the absence of any commercial or financial relationships that could be construed as a potential conflict of interest.

## Publisher's Note

All claims expressed in this article are solely those of the authors and do not necessarily represent those of their affiliated organizations, or those of the publisher, the editors and the reviewers. Any product that may be evaluated in this article, or claim that may be made by its manufacturer, is not guaranteed or endorsed by the publisher.
