# Supplementary material for: T-Cell Subsets and Interleukin-10 Levels Are Predictors of Severity and Mortality in COVID-19: A Systematic Review and Meta-Analysis
Source: Front Med (Lausanne). 2022 Apr 28;9:852749. doi: 10.3389/fmed.2022.852749 (PMC9096099; doi:10.3389/fmed.2022.852749)
Supplement: Supplementary file 1 [file Data_Sheet_1.pdf]

Supplementary Material

T-Cell Subsets and Interleukin-10 Levels Are Predictors of Severity and Mortality in COVID-19: A Systematic Review and Meta-Analysis

Amal F. Alshammary[1*], Jawaher M. Alsughayyir[1], Khalid K. Alharbi[1], Abdulrahman M. Al-Sulaiman[2], Haifa F. Alshammary[3], Heba F. Alshammary[4].

[1]Department of Clinical Laboratory Sciences, College of Applied Medical Sciences, King Saud University, Riyadh, Saudi Arabia.

[2]Department of Medical and Molecular Virology, Prince Sultan Military Medical City, Riyadh, Saudi Arabia.

[3]College of Applied Medical Sciences, Riyadh Elm University, Riyadh, Saudi Arabia.

[4]College of Dentistry, Riyadh Elm University, Riyadh, Saudi Arabia.

***Corresponding author: aalshammary@ksu.edu.sa**

Keywords: COVID-19, SARS-CoV-2, coronavirus, Interleukin 10, CD4, CD8, IL-10.

TABLE OF CONTENTS

**PART I**	**CHECKLISTS**	**6**
Table S1.1	PRISMA checklist	7
Table S1.2	MOOSE checklist	10
**PART II**	**DATABASE SEARCH**	**12**
Table S2.1	PubMed MEDLINE	13
Table S2.2	Scopus	14
Table S2.3	Web of Science	15
Table S2.4	EBSCO CINAHL	16
**PART III**	**DATA EXTRACTION FROM ARTICLE FIGURES**	**17**
Table S3	Laboratory data extraction from article figures	18
**PART IV**	**QUALITY ASSESSMENT OF INDIVIDUALE STUDIES**	**20**
Table S4	The Newcastle-Ottawa (NOS) Assessment Scale	21
**PART V**	**PRE- META-ANALYSIS COMPUTATION FORMULAS**	**22**
Table S5.1	Calculating the mean and standard deviation	23
Table S5.2	Combining similar subgroups	24
**PART VI**	**EXCLUDED STUDIES**	**26**
Table S6.1	Excluded studies due to reporting on severe cases	27
Table S6.2	Excluded studies due to overlap with included studies	29
**PART VII**	**GENERAL CHARACTERISTICS OF INCLUDED STUDIES**	**31**
Table S7.1	Classification protocol	32
Table S7.2	Sample acquisition time and test procedure	34
Table S7.3	Reported comorbidities	35
Table S7.4	Subgroup assignment	37

**PART VIII**	**POPULATION CHARACTERISTICS**	**38**
Table S8.1	Population mean size based on severity	39
Table S8.2	Population age based on severity	40
Table S8.3	Population mean size based on mortality	41
Table S8.4	Population age based on mortality	41
**PART IX**	**META-ANALYSIS SUMMARY**	**42**
Table S9.1	Meta-analysis summary for CD4 T-cells severity studies	43
Table S9.2	Meta-analysis summary for CD8 T-cells severity studies	44
Table S9.3	Meta-analysis summary for IL-10 severity studies	45
Table S9.4	Meta-analysis summary for CD4 T-cells mortality studies	46
Table S9.5	Meta-analysis summary for CD8 T-cells mortality studies	47
Table S9.6	Meta-analysis summary for IL-10 mortality studies	48
**PART X**	**INVESTIGATING THE SOURCE OF HETEROGENEITY-SUBGROUP ANALYSIS**	**49**
Figure S10.1	Subgroup analysis based on city for CD4 T-cell severity studies	50
Figure S10.2	Subgroup analysis based on country for CD4 T-cell severity studies	51
Figure S10.3	Subgroup analysis based on continent for CD4 T-cell severity studies	52
Figure S10.4	Subgroup analysis based on design for CD4 T-cell severity studies	53
Figure S10.5	Subgroup analysis based on protocol for CD4 T-cell severity studies	54
Figure S10.6	Subgroup analysis based on sample for CD4 T-cell severity studies	55
Figure S10.7	Subgroup analysis based on male number for CD4 T-cell severity studies	56
Figure S10.8	Subgroup analysis based on female number for CD4 T-cell severity studies	57
Figure S10.9	Subgroup analysis based on mean age for CD4 T-cell severity studies	58
Figure S10.10	Subgroup analysis based on test procedure for CD4 T-cell severity studies	59
Figure S10.11	Subgroup analysis based on city for CD8 T-cell severity studies	60
Figure S10.12	Subgroup analysis based on country for CD8 T-cell severity studies	61
Figure S10.13	Subgroup analysis based on continent for CD8 T-cell severity studies	62
Figure S10.14	Subgroup analysis based on design for CD8 T-cell severity studies	63
Figure S10.15	Subgroup analysis based on protocol for CD8 T-cell severity studies	64
Figure S10.16	Subgroup analysis based on sample for CD8 T-cell severity studies	65
Figure S10.17	Subgroup analysis based on male number for CD8 T-cell severity studies	66
Figure S10.18	Subgroup analysis based on female number for CD8 T-cell severity studies	67
Figure S10.19	Subgroup analysis based on mean age for CD8 T-cell severity studies	68
Figure S10.20	Subgroup analysis based on test procedure for CD8 T-cell severity studies	69
Figure S10.21	Subgroup analysis based on city for IL-10 severity studies	70
Figure S10.22	Subgroup analysis based on country for IL-10 severity studies	71
Figure S10.23	Subgroup analysis based on continent for IL-10 severity studies	72
Figure S10.24	Subgroup analysis based on design for IL-10 severity studies	73
Figure S10.25	Subgroup analysis based on protocol for IL-10 severity studies	74

Figure S10.26 Subgroup analysis based on sample for IL-10 severity studies 75
Figure S10.27 Subgroup analysis based on male number for IL-10 severity studies 76
Figure S10.28 Subgroup analysis based on female number for IL-10 severity studies 77
Figure S10.29 Subgroup analysis based on mean age for IL-10 severity studies 78
Figure S10.30 Subgroup analysis based on test procedure for IL-10 severity studies 79
Figure S10.31 Subgroup analysis based on city for CD4 T-cell mortality studies 80
Figure S10.32 Subgroup analysis based on country for CD4 T-cell mortality studies 81
Figure S10.33 Subgroup analysis based on continent for CD4 T-cell mortality studies 82
Figure S10.34 Subgroup analysis based on design for CD4 T-cell mortality studies 83
Figure S10.35 Subgroup analysis based on protocol for CD4 T-cell mortality studies 84
Figure S10.36 Subgroup analysis based on sample for CD4 T-cell mortality studies 85
Figure S10.37 Subgroup analysis based on male number for CD4 T-cell mortality studies 86
Figure S10.38 Subgroup analysis based on female number for CD4 T-cell mortality studies 87
Figure S10.39 Subgroup analysis based on mean age for CD4 T-cell mortality studies 88
Figure S10.40 Subgroup analysis based on test procedure for CD4 T-cell mortality studies 89
Figure S10.41 Subgroup analysis based on city for CD8 T-cell mortality studies 90
Figure S10.42 Subgroup analysis based on country for CD8 T-cell mortality studies 91
Figure S10.43 Subgroup analysis based on continent for CD8 T-cell mortality studies 92
Figure S10.44 Subgroup analysis based on design for CD8 T-cell mortality studies 93
Figure S10.45 Subgroup analysis based on protocol for CD8 T-cell mortality studies 94
Figure S10.46 Subgroup analysis based on sample for CD8 T-cell mortality studies 95
Figure S10.47 Subgroup analysis based on male number for CD8 T-cell mortality studies 96
Figure S10.48 Subgroup analysis based on female number for CD8 T-cell mortality studies 97
Figure S10.49 Subgroup analysis based on mean age for CD8 T-cell mortality studies 98
Figure S10.50 Subgroup analysis based on test procedure for CD8 T-cell mortality studies 99
Figure S10.51 Subgroup analysis based on city for IL-10 mortality studies 100
Figure S10.52 Subgroup analysis based on country for IL-10 mortality studies 101
Figure S10.53 Subgroup analysis based on continent for IL-10 mortality studies 102
Figure S10.54 Subgroup analysis based on design for IL-10 mortality studies 103
Figure S10.55 Subgroup analysis based on protocol for IL-10 mortality studies 104
Figure S10.56 Subgroup analysis based on sample for IL-10 mortality studies 105
Figure S10.57 Subgroup analysis based on male number for IL-10 mortality studies 106
Figure S10.58 Subgroup analysis based on female number for IL-10 mortality studies 107
Figure S10.59 Subgroup analysis based on mean age for IL-10 mortality studies 108
Figure S10.60 Subgroup analysis based on test procedure for IL-10 mortality studies 109

PART XI SMALL STUDY EFFECT AND PUBLICATION BIAS – FUNNEL PLOT 110

Figure S11.1 Analysis of publication bias for CD4 T-cell in COVID-19 severity studies 111
Figure S11.2 Analysis of publication bias for CD8 T-cell in COVID-19 severity studies 112
Figure S11.3 Analysis of publication bias for IL-10 in COVID-19 severity studies 113
Figure S11.4 Analysis of publication bias for CD4 T-cell in COVID-19 mortality studies 114
Figure S11.5 Analysis of publication bias for CD8 T-cell in COVID-19 mortality studies 115

Figure S11.6 Analysis of publication bias for IL-10 in COVID-19 mortality studies 116

PART XII THE LEAVE-ONE-OUT SENSITIVITY TEST 117

Figure S12.1 CD4 T-cells in COVID-19 severity studies 118
Figure S12.2 CD8 T-cells in COVID-19 severity studies 119
Figure S12.3 IL-10 in COVID-19 severity studies 120
Figure S12.4 CD4 T-cells in COVID-19 mortality studies 121
Figure S12.5 CD8 T-cells in COVID-19 mortality studies 122
Figure S12.6 IL-10 in COVID-19 mortality studies 123

PART I

CHECKLISTS

Table S1.1. PRISMA checklist for systematic review and meta-analysis of T-cell subsets and IL-10 in COVID-19 patients.

Section/topic	#	Checklist item	Reported on page #
**TITLE**			
Title	1	Identify the report as a systematic review, meta-analysis, or both.	1
**ABSTRACT**			
Structured summary	2	Provide a structured summary including, as applicable: background; objectives; data sources; study eligibility criteria, participants, and interventions; study appraisal and synthesis methods; results; limitations; conclusions and implications of key findings; systematic review registration number.	1-2
**INTRODUCTION**			
Rationale	3	Describe the rationale for the review in the context of what is already known.	3-6
Objectives	4	Provide an explicit statement of questions being addressed with reference to participants, interventions, comparisons, outcomes, and study design (PICOS).	5-6
**METHODS**			
Protocol and registration	5	Indicate if a review protocol exists, if and where it can be accessed (e.g., Web address), and, if available, provide registration information including registration number.	6
Eligibility criteria	6	Specify study characteristics (e.g., PICOS, length of follow-up) and report characteristics (e.g., years considered, language, publication status) used as criteria for eligibility, giving rationale.	6-8
Information sources	7	Describe all information sources (e.g., databases with dates of coverage, contact with study authors to identify additional studies) in the search and date last searched.	6-9
Search	8	Present full electronic search strategy for at least one database, including any limits used, such that it could be repeated.	S2.1-S2.4
Study selection	9	State the process for selecting studies (i.e., screening, eligibility, included in systematic review, and, if applicable, included in the meta-analysis).	9-10
Data collection process	10	Describe method of data extraction from reports (e.g., piloted forms, independently, in duplicate) and any processes for obtaining and confirming data from investigators.	10

Data items	11	List and define all variables for which data were sought (e.g., PICOS, funding sources) and any assumptions and simplifications made.	10-11
Risk of bias in individual studies	12	Describe methods used for assessing risk of bias of individual studies (including specification of whether this was done at the study or outcome level), and how this information is to be used in any data synthesis.	11
Summary measures	13	State the principal summary measures (e.g., risk ratio, difference in means).	11-13
Synthesis of results	14	Describe the methods of handling data and combining results of studies, if done, including measures of consistency (e.g., I^2) for each meta-analysis.	11-13
Risk of bias across studies	15	Specify any assessment of risk of bias that may affect the cumulative evidence (e.g., publication bias, selective reporting within studies).	9-10
Additional analyses	16	Describe methods of additional analyses (e.g., sensitivity or subgroup analyses, meta-regression), if done, indicating which were pre-specified.	14-16
**RESULTS**			
Study selection	17	Give numbers of studies screened, assessed for eligibility, and included in the review, with reasons for exclusions at each stage, ideally with a flow diagram.	17-18
Study characteristics	18	For each study, present characteristics for which data were extracted (e.g., study size, PICOS, follow-up period) and provide the citations.	19-26
Risk of bias within studies	19	Present data on risk of bias of each study and, if available, any outcome level assessment (see item 12).	27
Results of individual studies	20	For all outcomes considered (benefits or harms), present, for each study: (a) simple summary data for each intervention group (b) effect estimates and confidence intervals, ideally with a forest plot.	28-33 +S9.1-S9.6
Synthesis of results	21	Present results of each meta-analysis done, including confidence intervals and measures of consistency.	31-33
Risk of bias across studies	22	Present results of any assessment of risk of bias across studies (see Item 15).	34-42
Additional analysis	23	Give results of additional analyses, if done (e.g., sensitivity or subgroup analyses, meta-regression [see Item 16]).	36-43
**DISCUSSION**			
Summary of evidence	24	Summarize the main findings including the strength of evidence for each main outcome; consider their relevance to key groups (e.g., healthcare providers, users, and policy makers).	43-46
Limitations	25	Discuss limitations at study and outcome level (e.g., risk of bias), and at review-level (e.g., incomplete retrieval of identified research, reporting bias).	47
Conclusions	26	Provide a general interpretation of the results in the context of other evidence, and implications for future research.	46

FUNDING			
Funding	27	Describe sources of funding for the systematic review and other support (e.g., supply of data); role of funders for the systematic review.	48

Checklist is adapted from PRISMA checklist 2009 (1).

Reference

1. Moher D, Liberati A, Tetzlaff J, Altman DG, The PG. Preferred Reporting Items for Systematic Reviews and Meta-Analyses: The PRISMA Statement. PLOS Med. 2009;6(7):e1000097.

Table S1.2. MOOSE Checklist for systematic review and meta-analysis of T-cell subsets and IL-10 in COVID-19 patients.

Item No	Recommendation	Reported on Page No
Reporting of background should include		
1	Problem definition	3-5
2	Hypothesis statement	5-6
3	Description of study outcome(s)	6
4	Type of exposure or intervention used	6-7
5	Type of study designs used	7-9
6	Study population	7-9
Reporting of search strategy should include		
7	Qualifications of searchers (eg, librarians and investigators)	6 Investigators, title page
8	Search strategy, including time period included in the synthesis and key words	9
9	Effort to include all available studies, including contact with authors	10
10	Databases and registries searched	9-10
11	Search software used, name and version, including special features used (eg, explosion)	9-10
12	Use of hand searching (eg, reference lists of obtained articles)	9-10
13	List of citations located and those excluded, including justification	S6.1-S6.2
14	Method of addressing articles published in languages other than English	9
15	Method of handling abstracts and unpublished studies	6-8
16	Description of any contact with authors	10
Reporting of methods should include		
17	Description of relevance or appropriateness of studies assembled for assessing the hypothesis to be tested	10-11
18	Rationale for the selection and coding of data (eg, sound clinical principles or convenience)	10-11
19	Documentation of how data were classified and coded (eg, multiple raters, blinding and interrater reliability)	9-11
20	Assessment of confounding (eg, comparability of cases and controls in studies where appropriate)	9-11
21	Assessment of study quality, including blinding of quality assessors, stratification or regression on possible predictors of study results	11-14
22	Assessment of heterogeneity	14-16
23	Description of statistical methods (eg, complete description of fixed or random effects models, justification of whether the	11-14

	chosen models account for predictors of study results, dose-response models, or cumulative meta-analysis) in sufficient detail to be replicated	
24	Provision of appropriate tables and graphics	18-42
Reporting of results should include		
25	Graphic summarizing individual study estimates and overall estimate	28-33 +S9.1-S9.6
26	Table giving descriptive information for each study included	22-26
27	Results of sensitivity testing (eg, subgroup analysis)	S10.1-S10.12
28	Indication of statistical uncertainty of findings	36

Item No	Recommendation	Reported on Page No
Reporting of discussion should include		
29	Quantitative assessment of bias (eg, publication bias)	38-40
30	Justification for exclusion (eg, exclusion of non-English language citations)	34.43
31	Assessment of quality of included studies	27
Reporting of conclusions should include		
32	Consideration of alternative explanations for observed results	43-46
33	Generalization of the conclusions (ie, appropriate for the data presented and within the domain of the literature review)	46
34	Guidelines for future research	47
35	Disclosure of funding source	48

Checklist is adapted from MOOSE checklist for Meta-Analyses Of Observational Studies (1).

Reference

1. Stroup DF, Berlin JA, Morton SC, Olkin I, Williamson GD, Rennie D, et al. Meta-analysis of observational studies in epidemiology: a proposal for reporting. Meta-analysis Of Observational Studies in Epidemiology (MOOSE) group. JAMA. 2000;283(15):2008-12.

PART II

DATABASE SEARCH

AVAILABLE IN EXCEL

Table S2.1. PubMed MEDLINE (available in Excel)

PubMed MEDLINE			
**No.**	**Search Text**	**Results**	**Search Combination**
**1**	(COVID 19) AND (interleukin 10)	**82**	(COVID 19) AND (interleukin 10) AND (journalarticle[Filter]) AND (english[Filter])
**2**	(COVID 19) AND (IL 10)	**272**	(COVID 19) AND (IL 10) AND (journalarticle[Filter]) AND (english[Filter])
**3**	(SARS CoV 2) AND (interleukin 10)	**60**	(SARS CoV 2) AND (interleukin 10) AND (journalarticle[Filter]) AND (english[Filter])
**4**	(SARS CoV 2) AND (IL 10)	**183**	(SARS CoV 2) AND (IL 10) AND (journalarticle[Filter]) AND (english[Filter])
**5**	(COVID 19) AND (interleukin 10) AND (CD8)	**15**	(COVID 19) AND (interleukin 10) AND (CD8) AND (journalarticle[Filter]) AND (english[Filter])
**6**	(COVID 19) AND (interleukin 10) AND (CD4)	**14**	(COVID 19) AND (interleukin 10) AND (CD4) AND (journalarticle[Filter]) AND (english[Filter])
**7**	(COVID 19) AND (IL 10) AND (CD8)	**60**	(COVID 19) AND (IL 10) AND (CD8) AND (journalarticle[Filter]) AND (english[Filter])
**8**	(COVID 19) AND (IL 10) AND (CD4)	**59**	(COVID 19) AND (IL 10) AND (CD4) AND (journalarticle[Filter]) AND (english[Filter])
**9**	(SARS CoV 2) AND (interleukin 10) AND (CD8)	**12**	(SARS CoV 2) AND (interleukin 10) AND (CD8) AND (journalarticle[Filter]) AND (english[Filter])
**10**	(SARS CoV 2) AND (interleukin 10) AND (CD4)	**10**	(SARS CoV 2) AND (interleukin 10) AND (CD4) AND (journalarticle[Filter]) AND (english[Filter])
**11**	(SARS CoV 2) AND (IL 10) AND (CD8)	**50**	(SARS CoV 2) AND (IL 10) AND (CD8) AND (journalarticle[Filter]) AND (english[Filter])
**12**	(SARS CoV 2) AND (IL 10) AND (CD4)	**47**	(SARS CoV 2) AND (IL 10) AND (CD4) AND (journalarticle[Filter]) AND (english[Filter])
**13**	(COVID 19) AND (interleukin 10) AND (T cell)	**20**	(COVID 19) AND (interleukin 10) AND (T cell) AND (journalarticle[Filter]) AND (english[Filter])
**14**	(COVID 19) AND (IL 10) AND (T cell)	**77**	(COVID 19) AND (IL 10) AND (T cell) AND (journalarticle[Filter]) AND (english[Filter])
**15**	(SARS CoV 2) AND (interleukin 10) AND (T cell)	**19**	(SARS CoV 2) AND (interleukin 10) AND (T cell) AND (journalarticle[Filter]) AND (english[Filter])
**16**	(SARS CoV 2) AND (IL 10) AND (T cell)	**66**	(SARS CoV 2) AND (IL 10) AND (T cell) AND (journalarticle[Filter]) AND (english[Filter])
**17**	(COVID 19) AND (interleukin 10) AND (lymphocyte)	**40**	(COVID 19) AND (interleukin 10) AND (lymphocyte) AND (journalarticle[Filter]) AND (english[Filter])
**18**	(COVID 19) AND (IL 10) AND (lymphocyte)	**115**	(COVID 19) AND (IL 10) AND (lymphocyte) AND (journalarticle[Filter]) AND (english[Filter])
**19**	(SARS CoV 2) AND (interleukin 10) AND (lymphocyte)	**30**	(SARS CoV 2) AND (interleukin 10) AND (lymphocyte) AND (journalarticle[Filter]) AND (english[Filter])
**20**	(SARS CoV 2) AND (IL 10) AND (lymphocyte)	**91**	(SARS CoV 2) AND (IL 10) AND (lymphocyte) AND (journalarticle[Filter]) AND (english[Filter])
**Total exported into endnote under group name 1. PubMed: T-cells+IL-10**		1322	

Table S2.2. SCOPUS (available in Excel)

Scopus			
**No.**	**Search Text**	**Results**	**Search Combination**
**1**	(COVID 19) AND (interleukin 10)	**162**	(ALL(COVID 19) AND ALL(interleukin 10)) AND DOCTYPE(ar) AND PUBYEAR > 2018 AND (LIMIT-TO (PUBSTAGE,"aip")) AND (LIMIT-TO (DOCTYPE,"ar")) AND (LIMIT-TO (LANGUAGE,"English"))
**2**	(COVID 19) AND (IL 10)	**305**	(ALL(COVID 19) AND ALL(IL 10)) AND DOCTYPE(ar) AND PUBYEAR > 2018 AND (LIMIT-TO (PUBSTAGE,"aip")) AND (LIMIT-TO (DOCTYPE,"ar")) AND (LIMIT-TO (LANGUAGE,"English"))
**3**	(SARS CoV 2) AND (interleukin 10)	**124**	(ALL(SARS CoV 2) AND ALL(interleukin 10)) AND DOCTYPE(ar) AND PUBYEAR > 2018 AND (LIMIT-TO (PUBSTAGE,"aip")) AND (LIMIT-TO (DOCTYPE,"ar")) AND (LIMIT-TO (LANGUAGE,"English"))
**4**	(SARS CoV 2) AND (IL 10)	**191**	(ALL(SARS CoV 2) AND ALL(IL 10)) AND DOCTYPE(ar) AND PUBYEAR > 2018 AND (LIMIT-TO (PUBSTAGE,"aip")) AND (LIMIT-TO (DOCTYPE,"ar")) AND (LIMIT-TO (LANGUAGE,"English"))
**5**	(COVID 19) AND (interleukin 10) AND (CD8)	**28**	(ALL(COVID 19) AND ALL(interleukin 10) AND ALL(CD8)) AND DOCTYPE(ar) AND PUBYEAR > 2018 AND (LIMIT-TO (PUBSTAGE,"aip")) AND (LIMIT-TO (DOCTYPE,"ar")) AND (LIMIT-TO (LANGUAGE,"English"))
**6**	(COVID 19) AND (interleukin 10) AND (CD4)	**30**	(ALL(COVID 19) AND ALL(interleukin 10) AND ALL(CD4)) AND DOCTYPE(ar) AND PUBYEAR > 2018 AND (LIMIT-TO (PUBSTAGE,"aip")) AND (LIMIT-TO (DOCTYPE,"ar")) AND (LIMIT-TO (LANGUAGE,"English"))
**7**	(COVID 19) AND (IL 10) AND (CD8)	**36**	(ALL(COVID 19) AND ALL(IL 10) AND ALL(CD8)) AND DOCTYPE(ar) AND PUBYEAR > 2018 AND (LIMIT-TO (PUBSTAGE,"aip")) AND (LIMIT-TO (DOCTYPE,"ar")) AND (LIMIT-TO (LANGUAGE,"English"))
**8**	(COVID 19) AND (IL 10) AND (CD4)	**34**	(ALL(COVID 19) AND ALL(IL 10) AND ALL(CD4)) AND DOCTYPE(ar) AND PUBYEAR > 2018 AND (LIMIT-TO (PUBSTAGE,"aip")) AND (LIMIT-TO (DOCTYPE,"ar")) AND (LIMIT-TO (LANGUAGE,"English"))
**9**	(SARS CoV 2) AND (interleukin 10) AND (CD8)	**24**	(ALL(SARS CoV 2) AND ALL(interleukin 10) AND ALL(CD8)) AND DOCTYPE(ar) AND PUBYEAR > 2018 AND (LIMIT-TO (PUBSTAGE,"aip")) AND (LIMIT-TO (DOCTYPE,"ar")) AND (LIMIT-TO (LANGUAGE,"English"))
**10**	(SARS CoV 2) AND (interleukin 10) AND (CD4)	**24**	(ALL(SARS CoV 2) AND ALL(interleukin 10) AND ALL(CD4)) AND DOCTYPE(ar) AND PUBYEAR > 2018 AND (LIMIT-TO (PUBSTAGE,"aip")) AND (LIMIT-TO (DOCTYPE,"ar")) AND (LIMIT-TO (LANGUAGE,"English"))
**11**	(SARS CoV 2) AND (IL 10) AND (CD8)	**32**	(ALL(SARS CoV 2) AND ALL(IL 10) AND ALL(CD8)) AND DOCTYPE(ar) AND PUBYEAR > 2018 AND (LIMIT-TO (PUBSTAGE,"aip")) AND (LIMIT-TO (DOCTYPE,"ar")) AND (LIMIT-TO (LANGUAGE,"English"))
**12**	(SARS CoV 2) AND (IL 10) AND (CD4)	**29**	(ALL(SARS CoV 2) AND ALL(IL 10) AND ALL(CD4)) AND DOCTYPE(ar) AND PUBYEAR > 2018 AND (LIMIT-TO (PUBSTAGE,"aip")) AND (LIMIT-TO (DOCTYPE,"ar")) AND (LIMIT-TO (LANGUAGE,"English"))
**13**	(COVID 19) AND (interleukin 10) AND (T cell)	**116**	(ALL(COVID 19) AND ALL(interleukin 10) AND ALL(T cell)) AND DOCTYPE(ar) AND PUBYEAR > 2018 AND (LIMIT-TO (PUBSTAGE,"aip")) AND (LIMIT-TO (DOCTYPE,"ar")) AND (LIMIT-TO (LANGUAGE,"English"))
**14**	(COVID 19) AND (IL 10) AND (T cell)	**169**	(ALL(COVID 19) AND ALL(IL 10) AND ALL(T cell)) AND DOCTYPE(ar) AND PUBYEAR > 2018 AND (LIMIT-TO (PUBSTAGE,"aip")) AND (LIMIT-TO (DOCTYPE,"ar")) AND (LIMIT-TO (LANGUAGE,"English"))
**15**	(SARS CoV 2) AND (interleukin 10) AND (T cell)	**93**	(ALL(SARS Cov 2) AND ALL(interleukin 10) AND ALL(T cell)) AND DOCTYPE(ar) AND PUBYEAR > 2018 AND (LIMIT-TO (PUBSTAGE,"aip")) AND (LIMIT-TO (DOCTYPE,"ar")) AND (LIMIT-TO (LANGUAGE,"English"))
**16**	(SARS CoV 2) AND (IL 10) AND (T cell)	**140**	(ALL(SARS Cov 2) AND ALL(IL 10) AND ALL(T cell)) AND DOCTYPE(ar) AND PUBYEAR > 2018 AND (LIMIT-TO (PUBSTAGE,"aip")) AND (LIMIT-TO (DOCTYPE,"ar")) AND (LIMIT-TO (LANGUAGE,"English"))
**17**	(COVID 19) AND (interleukin 10) AND (lymphocyte)	**51**	(ALL(COVID 19) AND ALL(interleukin 10) AND ALL(lymphocyte)) AND DOCTYPE(ar) AND PUBYEAR > 2018 AND (LIMIT-TO (PUBSTAGE,"aip")) AND (LIMIT-TO (DOCTYPE,"ar")) AND (LIMIT-TO (LANGUAGE,"English"))
**18**	(COVID 19) AND (IL 10) AND (lymphocyte)	**62**	(ALL(COVID 19) AND ALL(IL 10) AND ALL(lymphocyte)) AND DOCTYPE(ar) AND PUBYEAR > 2018 AND (LIMIT-TO (PUBSTAGE,"aip")) AND (LIMIT-TO (DOCTYPE,"ar")) AND (LIMIT-TO (LANGUAGE,"English"))
**19**	(SARS CoV 2) AND (interleukin 10) AND (lymphocyte)	**40**	(ALL(SARS CoV 2) AND ALL(interleukin 10) AND ALL(lymphocyte)) AND DOCTYPE(ar) AND PUBYEAR > 2018 AND (LIMIT-TO (PUBSTAGE,"aip")) AND (LIMIT-TO (DOCTYPE,"ar")) AND (LIMIT-TO (LANGUAGE,"English"))
**20**	(SARS CoV 2) AND (IL 10) AND (lymphocyte)	**54**	(ALL(SARS CoV 2) AND ALL(IL 10) AND ALL(lymphocyte)) AND DOCTYPE(ar) AND PUBYEAR > 2018 AND (LIMIT-TO (PUBSTAGE,"aip")) AND (LIMIT-TO (DOCTYPE,"ar")) AND (LIMIT-TO (LANGUAGE,"English"))
**Total exported into Endnote under group name 2. Scopus: T-cells+IL-10**		**1744**	

Table S2.3. Web of Science (available in Excel)

Web of Science					
**No.**	**Search Text**	**Results**	**Search Combination**		
**1**	(COVID 19) AND (interleukin 10)	**240**	You searched for: TOPIC: (COVID 19) *AND* TOPIC: (interleukin 10)	Refined by: DOCUMENT TYPES: (ARTICLE) AND LANGUAGES: (ENGLISH)	Timespan: Year to date. Indexes: SCI-EXPANDED, SSCI, A&HCI, CPCI-S, CPCI-SSH, ESCI, CCR-EXPANDED, IC.
**2**	(COVID 19) AND (IL 10)	**284**	You searched for: TOPIC: (COVID 19) *AND* TOPIC: (IL 10)	Refined by: DOCUMENT TYPES: (ARTICLE) AND LANGUAGES: (ENGLISH)	Timespan: Year to date. Indexes: SCI-EXPANDED, SSCI, A&HCI, CPCI-S, CPCI-SSH, ESCI, CCR-EXPANDED, IC.
**3**	(SARS CoV 2) AND (interleukin 10)	**144**	You searched for: TOPIC: (SARS CoV 19) *AND* TOPIC: (interleukin 10)	Refined by: DOCUMENT TYPES: (ARTICLE) AND LANGUAGES: (ENGLISH)	Timespan: Year to date. Indexes: SCI-EXPANDED, SSCI, A&HCI, CPCI-S, CPCI-SSH, ESCI, CCR-EXPANDED, IC.
**4**	(SARS CoV 2) AND (IL 10)	**198**	You searched for: TOPIC: (SARS CoV 19) *AND* TOPIC: (IL 10)	Refined by: DOCUMENT TYPES: (ARTICLE) AND LANGUAGES: (ENGLISH)	Timespan: Year to date. Indexes: SCI-EXPANDED, SSCI, A&HCI, CPCI-S, CPCI-SSH, ESCI, CCR-EXPANDED, IC.
**5**	(COVID 19) AND (interleukin 10) AND (CD8)	**9**	You searched for: TOPIC: (COVID 19) *AND* TOPIC: (interleukin 10) AND TOPIC: (CD8)	Refined by: DOCUMENT TYPES: (ARTICLE) AND LANGUAGES: (ENGLISH)	Timespan: Year to date. Indexes: SCI-EXPANDED, SSCI, A&HCI, CPCI-S, CPCI-SSH, ESCI, CCR-EXPANDED, IC.
**6**	(COVID 19) AND (interleukin 10) AND (CD4)	**11**	You searched for: TOPIC: (COVID 19) *AND* TOPIC: (interleukin 10) AND TOPIC: (CD4)	Refined by: DOCUMENT TYPES: (ARTICLE) AND LANGUAGES: (ENGLISH)	Timespan: Year to date. Indexes: SCI-EXPANDED, SSCI, A&HCI, CPCI-S, CPCI-SSH, ESCI, CCR-EXPANDED, IC.
**7**	(COVID 19) AND (IL 10) AND (CD8)	**35**	You searched for: TOPIC: (COVID 19) *AND* TOPIC: (IL 10) AND TOPIC: (CD8)	Refined by: DOCUMENT TYPES: (ARTICLE) AND LANGUAGES: (ENGLISH)	Timespan: Year to date. Indexes: SCI-EXPANDED, SSCI, A&HCI, CPCI-S, CPCI-SSH, ESCI, CCR-EXPANDED, IC.
**8**	(COVID 19) AND (IL 10) AND (CD4)	**30**	You searched for: TOPIC: (COVID 19) *AND* TOPIC: (IL 10) AND TOPIC: (CD4)	Refined by: DOCUMENT TYPES: (ARTICLE) AND LANGUAGES: (ENGLISH)	Timespan: Year to date. Indexes: SCI-EXPANDED, SSCI, A&HCI, CPCI-S, CPCI-SSH, ESCI, CCR-EXPANDED, IC.
**9**	(SARS CoV 2) AND (interleukin 10) AND (CD8)	**6**	You searched for: TOPIC: (SARS CoV 19) *AND* TOPIC: (interleukin 10) AND TOPIC: (CD8)	Refined by: DOCUMENT TYPES: (ARTICLE) AND LANGUAGES: (ENGLISH)	Timespan: Year to date. Indexes: SCI-EXPANDED, SSCI, A&HCI, CPCI-S, CPCI-SSH, ESCI, CCR-EXPANDED, IC.
**10**	(SARS CoV 2) AND (interleukin 10) AND (CD4)	**6**	You searched for: TOPIC: (SARS CoV 19) *AND* TOPIC: (interleukin 10) AND TOPIC: (CD4)	Refined by: DOCUMENT TYPES: (ARTICLE) AND LANGUAGES: (ENGLISH)	Timespan: Year to date. Indexes: SCI-EXPANDED, SSCI, A&HCI, CPCI-S, CPCI-SSH, ESCI, CCR-EXPANDED, IC.
**11**	(SARS CoV 2) AND (IL 10) AND (CD8)	**23**	You searched for: TOPIC: (SARS CoV 19) *AND* TOPIC: (IL 10) AND TOPIC: (CD8)	Refined by: DOCUMENT TYPES: (ARTICLE) AND LANGUAGES: (ENGLISH)	Timespan: Year to date. Indexes: SCI-EXPANDED, SSCI, A&HCI, CPCI-S, CPCI-SSH, ESCI, CCR-EXPANDED, IC.
**12**	(SARS CoV 2) AND (IL 10) AND (CD4)	**19**	You searched for: TOPIC: (SARS CoV 19) *AND* TOPIC: (IL 10) AND TOPIC: (CD4)	Refined by: DOCUMENT TYPES: (ARTICLE) AND LANGUAGES: (ENGLISH)	Timespan: Year to date. Indexes: SCI-EXPANDED, SSCI, A&HCI, CPCI-S, CPCI-SSH, ESCI, CCR-EXPANDED, IC.
**13**	(COVID 19) AND (interleukin 10) AND (T cell)	**16**	You searched for: TOPIC: (COVID 19) *AND* TOPIC: (interleukin 10) AND TOPIC: (T cell)	Refined by: DOCUMENT TYPES: (ARTICLE) AND LANGUAGES: (ENGLISH)	Timespan: Year to date. Indexes: SCI-EXPANDED, SSCI, A&HCI, CPCI-S, CPCI-SSH, ESCI, CCR-EXPANDED, IC.
**14**	(COVID 19) AND (IL 10) AND (T cell)	**48**	You searched for: TOPIC: (COVID 19) *AND* TOPIC: (IL 10) AND TOPIC: (T cell)	Refined by: DOCUMENT TYPES: (ARTICLE) AND LANGUAGES: (ENGLISH)	Timespan: Year to date. Indexes: SCI-EXPANDED, SSCI, A&HCI, CPCI-S, CPCI-SSH, ESCI, CCR-EXPANDED, IC.
**15**	(SARS CoV 2) AND (interleukin 10) AND (T cell)	**11**	You searched for: TOPIC: (SARS CoV 2) *AND* TOPIC: (interleukin 10) *AND* TOPIC: (T cell)	Refined by: DOCUMENT TYPES: (ARTICLE) AND LANGUAGES: (ENGLISH)	Timespan: Year to date. Indexes: SCI-EXPANDED, SSCI, A&HCI, CPCI-S, CPCI-SSH, ESCI, CCR-EXPANDED, IC.
**16**	(SARS CoV 2) AND (IL 10) AND (T cell)	**29**	You searched for: TOPIC: (SARS CoV 2) *AND* TOPIC: (IL 10) *AND* TOPIC: (T cell)	Refined by: DOCUMENT TYPES: (ARTICLE) AND LANGUAGES: (ENGLISH)	Timespan: Year to date. Indexes: SCI-EXPANDED, SSCI, A&HCI, CPCI-S, CPCI-SSH, ESCI, CCR-EXPANDED, IC.
**17**	(COVID 19) AND (interleukin 10) AND (lymphocyte)	**33**	You searched for: TOPIC: (COVID 19) *AND* TOPIC: (interleukin 10) *AND* TOPIC: (lymphocyte)	Refined by: DOCUMENT TYPES: (ARTICLE) AND LANGUAGES: (ENGLISH)	Timespan: Year to date. Indexes: SCI-EXPANDED, SSCI, A&HCI, CPCI-S, CPCI-SSH, ESCI, CCR-EXPANDED, IC.
**18**	(COVID 19) AND (IL 10) AND (lymphocyte)	**56**	You searched for: TOPIC: (COVID 19) *AND* TOPIC: (IL 10) *AND* TOPIC: (lymphocyte)	Refined by: DOCUMENT TYPES: (ARTICLE) AND LANGUAGES: (ENGLISH)	Timespan: Year to date. Indexes: SCI-EXPANDED, SSCI, A&HCI, CPCI-S, CPCI-SSH, ESCI, CCR-EXPANDED, IC.
**19**	(SARS CoV 2) AND (interleukin 10) AND (lymphocyte)	**18**	You searched for: TOPIC: (SARS CoV 2) *AND* TOPIC: (interleukin 10) *AND* TOPIC: (lymphocyte)	Refined by: DOCUMENT TYPES: (ARTICLE) AND LANGUAGES: (ENGLISH)	Timespan: Year to date. Indexes: SCI-EXPANDED, SSCI, A&HCI, CPCI-S, CPCI-SSH, ESCI, CCR-EXPANDED, IC.
**20**	(SARS CoV 2) AND (IL 10) AND (lymphocyte)	**31**	You searched for: TOPIC: (SARS CoV 2) *AND* TOPIC: (IL 10) *AND* TOPIC: (lymphocyte)	Refined by: DOCUMENT TYPES: (ARTICLE) AND LANGUAGES: (ENGLISH)	Timespan: Year to date. Indexes: SCI-EXPANDED, SSCI, A&HCI, CPCI-S, CPCI-SSH, ESCI, CCR-EXPANDED, IC.
**Total exported into Endnote under group name 3. Web of Science: T-cells+IL-10**		1247			

Table S2.4. EBSCO (available in Excel)

EBSCO			
**No.**	**Search Text**	**Results**	**Search Combination**
1	(COVID 19) AND (interleukin 10)	2	**Find all my search terms:** COVID 19 AND interleukin 10 **Expanders:** XAlso search within the full text of the articles **Limiters:** XPublished Date: 20190101-20210522 **English Language** **Publication Type:** Journal Article
2	(COVID 19) AND (IL 10)	5	**Find all my search terms:** COVID 19 AND IL 10 **Expanders:** XAlso search within the full text of the articles **Limiters:** XPublished Date: 20190101-20210522 **English Language** **Publication Type:** Journal Article
3	(SARS CoV 2) AND (interleukin 10)	3	**Find all my search terms:** SARS CoV 2 AND interleukin 10 **Expanders:** XAlso search within the full text of the articles **Limiters:** XPublished Date: 20190101-20210522 **English Language** **Publication Type:** Journal Article
4	(SARS CoV 2) AND (IL 10)	6	**Find all my search terms:** SARS CoV 2 AND IL 10 **Expanders:** XAlso search within the full text of the articles **Limiters:** XPublished Date: 20190101-20210522 **English Language** **Publication Type:** Journal Article
5	(COVID 19) AND (interleukin 10) AND (CD8)	5	**Find all my search terms:** COVID 19 AND interleukin 10 AND CD8 **Expanders:** XAlso search within the full text of the articles **Limiters:** XPublished Date: 20190101-20210522 **English Language** **Publication Type:** Journal Article
6	(COVID 19) AND (interleukin 10) AND (CD4)	5	**Find all my search terms:** COVID 19 AND interleukin 10 AND CD4 **Expanders:** XAlso search within the full text of the articles **Limiters:** XPublished Date: 20190101-20210522 **English Language** **Publication Type:** Journal Article
7	(COVID 19) AND (IL 10) AND (CD8)	14	**Find all my search terms:** COVID 19 AND IL 10 AND CD8 **Expanders:** XAlso search within the full text of the articles **Limiters:** XPublished Date: 20190101-20210522 **English Language** **Publication Type:** Journal Article
8	(COVID 19) AND (IL 10) AND (CD4)	14	**Find all my search terms:** COVID 19 AND IL 10 AND CD4 **Expanders:** XAlso search within the full text of the articles **Limiters:** XPublished Date: 20190101-20210522 **English Language** **Publication Type:** Journal Article
9	(SARS CoV 2) AND (interleukin 10) AND (CD8)	182	**Find all my search terms:** SARS CoV 2 AND interleukin 10 AND CD8 **Expanders:** XAlso search within the full text of the articles **Limiters:** XPublished Date: 20190101-20210522 **English Language** **Publication Type:** Journal Article
10	(SARS CoV 2) AND (interleukin 10) AND (CD4)	182	**Find all my search terms:** SARS CoV 2 AND interleukin 10 AND CD4 **Expanders:** XAlso search within the full text of the articles **Limiters:** XPublished Date: 20190101-20210522 **English Language** **Publication Type:** Journal Article
11	(SARS CoV 2) AND (IL 10) AND (CD8)	184	**Find all my search terms:** SARS CoV 2 AND IL 10 AND CD8 **Expanders:** XAlso search within the full text of the articles **Limiters:** XPublished Date: 20190101-20210522 **English Language** **Publication Type:** Journal Article
12	(SARS CoV 2) AND (IL 10) AND (CD4)	184	**Find all my search terms:** SARS CoV 2 AND IL 10 AND CD4 **Expanders:** XAlso search within the full text of the articles **Limiters:** XPublished Date: 20190101-20210522 **English Language** **Publication Type:** Journal Article
13	(COVID 19) AND (interleukin 10) AND (T cell)	2	**Find all my search terms:** COVID 19 AND interleukin 10 AND T cell **Expanders:** XAlso search within the full text of the articles **Limiters:** XPublished Date: 20190101-20210522 **English Language** **Publication Type:** Journal Article
14	(COVID 19) AND (IL 10) AND (T cell)	2	**Find all my search terms:** COVID 19 AND IL 10 AND T cell **Expanders:** XAlso search within the full text of the articles **Limiters:** XPublished Date: 20190101-20210522 **English Language** **Publication Type:** Journal Article
15	(SARS CoV 2) AND (interleukin 10) AND (T cell)	2	**Find all my search terms:** SARS CoV 2 AND interleukin 10 AND T cell **Expanders:** XAlso search within the full text of the articles **Limiters:** XPublished Date: 20190101-20210522 **English Language** **Publication Type:** Journal Article
16	(SARS CoV 2) AND (IL 10) AND (T cell)	3	**Find all my search terms:** SARS CoV 2 AND IL 10 AND T cell **Expanders:** XAlso search within the full text of the articles **Limiters:** XPublished Date: 20190101-20210522 **English Language** **Publication Type:** Journal Article
17	(COVID 19) AND (interleukin 10) AND (lymphocyte)	2	**Find all my search terms:** COVID 19 AND interleukin 10 AND lymphocyte **Expanders:** XAlso search within the full text of the articles **Limiters:** XPublished Date: 20190101-20210522 **English Language** **Publication Type:** Journal Article
18	(COVID 19) AND (IL 10) AND (lymphocyte)	2	**Find all my search terms:** COVID 19 AND IL 10 AND lymphocyte **Expanders:** XAlso search within the full text of the articles **Limiters:** XPublished Date: 20190101-20210522 **English Language** **Publication Type:** Journal Article
19	(SARS CoV 2) AND (interleukin 10) AND (lymphocyte)	2	**Find all my search terms:** SARS CoV 2 AND interleukin 10 AND lymphocyte **Expanders:** XAlso search within the full text of the articles **Limiters:** XPublished Date: 20190101-20210522 **English Language** **Publication Type:** Journal Article
20	(SARS CoV 2) AND (IL 10) AND (lymphocyte)	3	**Find all my search terms:** SARS CoV 2 AND IL 10 AND lymphocyte **Expanders:** XAlso search within the full text of the articles **Limiters:** XPublished Date: 20190101-20210522 **English Language** **Publication Type:** Journal Article
**Total exported into Endnote under group name 4. EBSCO: T-cells+IL-10**		**804**	

PART III

DATA EXTRACTION
FROM ARTICLE FIGURES

Laboratory data extracted from article figures

Table S3. Laboratory parameters extracted from article figures

Study	Laboratory parameter		
	**CD4 T-cell**	**CD8 T-cell**	**IL-10**
Azmy, Veronica 2021 (1)*	N/A	N/A	Figure 1C
Diao, Bo 2020 (2)	N/A	N/A	Figure 2A
Flament, Heloise 2021 (3)	Figure 1C	Figure 1C	Figure 6A
Guan, Jingling 2020 (4)	Figure 3B	Figure 3B	Figure 4B
He, Bing 2020 (5)	N/A	N/A	Figure 1E
Li, Mingyue 2020 (6)	Figure 3A	Figure 3A	Figure 1A
Mann, Elizabeth 2020 (7)	Figure 3B	Figure 3A	Figure 1E
Rendeiro, Andre 2020 (8)	Figure 2B	Figure 2b	N/A
Shi, Hongbo 2020 (9)	Figure 2C	Figure 2H	Figure 3D
Schrijver, Benjamin 2020 (10)	Figure 3A	Figure 3A	N/A
Tan, Mingkai 2020 (11)	Figure 2D	Figure 2E	Figure 5C

* IL-10 values were extracted from article figure because laboratory values were provided without SD values.

References

1. Azmy V, Kaman K, Tang D, Zhao H, Dela Cruz C, Topal JE, et al. Cytokine Profiles Before and After Immune Modulation in Hospitalized Patients with COVID-19. Journal of clinical immunology. 2021:1-10.

2. Diao B, Wang C, Tan Y, Chen X, Liu Y, Ning L, et al. Reduction and Functional Exhaustion of T Cells in Patients With Coronavirus Disease 2019 (COVID-19). Front Immunol. 2020;11:827.

3. Flament H, Rouland M, Beaudoin L, Toubal A, Bertrand L, Lebourgeois S, et al. Outcome of SARS-CoV-2 infection is linked to MAIT cell activation and cytotoxicity. Nature Immunology. 2021;22(3):322-35.

4. Guan J, Wei X, Qin S, Liu X, Jiang Y, Chen Y, et al. Continuous tracking of COVID-19 patients' immune status. Int Immunopharmacol. 2020;89(Pt A):107034.

5. He B, Wang J, Wang Y, Zhao J, Huang J, Tian Y, et al. The Metabolic Changes and Immune Profiles in Patients With COVID-19. Frontiers in Immunology. 2020;11.

6. Li M, Guo W, Dong Y, Wang X, Dai D, Liu X, et al. Elevated Exhaustion Levels of NK and CD8(+) T Cells as Indicators for Progression and Prognosis of COVID-19 Disease. Front Immunol. 2020;11:580237.

7. Mann ER, Menon M, Knight SB, Konkel JE, Jagger C, Shaw TN, et al. Longitudinal immune profiling reveals key myeloid signatures associated with COVID-19. Sci Immunol. 2020;5(51).

8. Rendeiro AF, Casano J, Vorkas CK, Singh H, Morales A, DeSimone RA, et al. Profiling of immune dysfunction in COVID-19 patients allows early prediction of disease progression. Life science alliance. 2020;4(2):e202000955.

9. Shi H, Wang W, Yin J, Ouyang Y, Pang L, Feng Y, et al. The inhibition of IL-2/IL-2R gives rise to CD8(+) T cell and lymphocyte decrease through JAK1-STAT5 in critical patients with COVID-19 pneumonia. Cell Death Dis. 2020;11(6):429.

10. Schrijver B, Assmann JLJC, van Gammeren AJ, Vermeulen RCH, Portengen L, Heukels P, et al. Extensive longitudinal immune profiling reveals sustained innate immune activation in COVID-19 patients with unfavorable outcome. Eur Cytokine Netw. 2020;31(4):154-67.

11. Tan M, Liu Y, Zhou R, Deng X, Li F, Liang K, et al. Immunopathological characteristics of coronavirus disease 2019 cases in Guangzhou, China. Immunology. 2020;160(3):261-8.

PART IV

QUALITY ASSESSMENT OF INDIVIDUALE STUDIES

THE NEWCASTLE-OTTAWA (NOS) ASSESSMENT SCALE

AVAILABLE IN EXCEL

The Newcastle-Ottawa (NOS) assessment scale

Table S4. The Newcastle-Ottawa (NOS) assessment scale (available in Excel)

The Necastle-Ottawa Quality Assessment Scale (NOS) for Cohort Studies	Study Name	Abers, Mic	Azmy, Ver	Cantenys-M	Carissimo, (	Chen, Jiaxin	Chi, Ying	Deng, Fuxu	Diao, Bo	Feng, Xiaob	Flament, H	Gadotti, An	Guan, Jingji	Han, Huan	He, Bing	He, Susu	Henry, Bra	Huang, Ho	Huang, We	Hue, Sophi	Jin, Xiao-Ho	Keddie, Ste	Kwon, Ji-So	Laing, Adar	Li, Chenze	Li, Mingyue	Li, Qiang	Li, Xiaolei	Liao, Baolin	Liu, Fangfa	Liu, Jian	Liu, Jing	Liu, Lei	Liu, Yangli	Liu, Xue-Qin	Luo, Miao	Mann, Eliz	McElvaney	Rendeiro, A	Schrijver, B	Shi, Hongb	Tan, Mingk	Wang, Zho	Xu, Bo	Yang, Ai-Pi	Yang, Fan	Yi, Ping	Zeng, Hao-	Zeng, Zhilin	Zhang, Bo	Zhang, Jun	Zhao, Yan	Zou, Li
	Year	2020	2020	2020	2020	2020	2020	2020	2020	2020	2021	2020	2020	2020	2020	2020	2021	2020	2021	2020	2020	2020	2020	2020	2020	2020	2020	2020	2021	2020	2020	2020	2020	2021	2021	2020	2020	2020	2020	2020	2020	2020	2020	2020	2020	2020	2020	2020	2020	2021	2020	2020	2020
**Selection**	Studies																																																				
1. Representativeness of the exposed cohort																																																					
a) truly representative of the average _______________ (describe) in the community ★		★	★	★	★	★	★	★	★	★	★													★	★					★						★			★					★					★			★	★
b) somewhat representative of the average ______________ in the community ★												★	★	★	★	★	★	★	★	★	★	★	★			★	★	★	★		★	★	★	★	★		★	★	★	★	★	★	★		★	★	★	★		★	★		
c) selected group of users eg nurses, volunteers																																																					
d) no description of the derivation of the cohort																																																					
2. Selection of the non exposed cohort																																																					
a) drawn from the same community as the exposed cohort ★					★		★						★		★									★		★									★		★	★			★	★										★	
b) drawn from a different source																																																					
c) no description of the derivation of the non exposed cohort																																																					
3. Ascertainment of exposure																																																					
a) secure record (eg surgical records) ★		★	★	★	★	★	★	★	★	★	★	★	★	★	★	★	★	★	★	★	★	★	★	★	★	★	★	★	★	★	★	★	★	★	★	★	★	★	★	★	★	★	★	★	★	★	★	★	★	★	★	★	★
b) structured interview ★																									★																												
c) written self report																																																					
d) no description																																																					
4. Demonstration that outcome of interest was not present at start of study																																																					
a) yes ★			★	★													★							★		★			★	★			★				★	★	★		★	★										★	
b) no																																																					
**Comparability**																																																					
1. Comparability of cohorts on the basis of the design or analysis																																																					
a) study controls for _____________ (select the most important factor) ★		★	★	★	★	★	★	★	★	★	★	★	★	★	★	★	★	★	★	★	★	★	★	★	★	★	★	★	★	★	★	★	★	★	★	★	★	★	★	★	★	★	★	★	★		★		★		★	★	★
b) study controls for any additional factor ★ (This criteria could be modified to indicate specific control for a second important factor.)																																																					
**Outcome**																																																					
1. Assessment of outcome																																																						
a) independent blind assessment ★		★	★	★	★	★	★	★	★	★	★	★	★	★	★	★	★	★	★	★	★	★	★	★	★	★	★	★	★	★	★	★	★	★	★	★	★	★	★	★	★	★	★	★	★	★	★	★	★	★	★	★	★
b) record linkage ★		★	★	★	★	★	★	★	★	★	★	★	★		★	★	★	★	★	★	★	★	★	★	★	★	★	★	★	★	★	★	★		★	★	★	★	★	★	★	★	★	★	★	★	★	★	★	★	★	★	★
c) self report																																																					
d) no description																																																					
2. Was follow-up long enough for outcomes to occur																																																					
a) yes (select an adequate follow up period for outcome of interest) ★				★	★	★				★	★	★		★	★	★	★		★	★	★	★	★	★	★	★	★	★	★	★	★	★	★	★	★	★	★		★	★		★	★	★	★	★	★	★	★	★	★		★
b) no																																																					
3. Adequacy of follow up of cohorts																																																						
a) complete follow up - all subjects accounted for ★		★	★		★			★	★	★	★	★		★	★	★	★	★	★	★	★	★	★	★	★	★		★	★	★	★	★	★	★	★	★	★		★	★		★	★	★	★	★	★	★	★	★	★		★
b) subjects lost to follow up unlikely to introduce bias - small number lost - > ____ % (select an adequate %) follow up, or description provided of those lost) ★																																																					
c) follow up rate < ____% (select an adequate %) and no description of those lost																																																					
d) no statement																																																					
	Score	6	7	7	8	6	6	6	6	7	7	7	6	6	8	7	8	6	7	7	7	7	7	9	8	9	6	7	8	8	7	7	8	6	8	7	9	7	9	7	7	9	7	7	7	6	7	6	7	6	7	7	7

PART V

PRE- META-ANALYSIS
COMPUTATION FORMULAS

Pre-meta-analysis computational formulas

Table S5.1. Pre- meta-analysis computational formulas for calculating the mean and standard deviation.

**Formulas for calculating the mean and standard deviation from median and IQR (maximum – minimum) or median and IQR**	
**Calculating the mean from (median, q1, and q3)** (1)	$\bar{x} = \frac{q_1 + m + q_3}{3}$
	Excel formula = *(q_1+m+ q_3)/3*
**Calculating the SD from (median, q1, q3, and n)** (1)	$SD = \frac{q_3 - q_1}{2 \times \theta^{-1}\left(\frac{0.75 \times n - 0.125}{n + 0.25}\right)}$
	Excel formula = *(q_3– q_1)/(2*NORM.INV((0.75*n–0.125)/(n+0.25),0,1))*
**Calculating the mean and SD from (median)** (2)	*Median = Mean*<br>$SD = \frac{IQR}{1.35}$
	Excel formula = *IQR/1.35*

Table S5.2. Computational formulas for combining similar subgroups

**Formulas for combining similar subgroups**	
**Combining the mean for similar subgroups** (2)	$\bar{x}_C = \frac{(n_1 \times m_1) + (n_2 \times m_2)}{n_1 + n_2}$
	Excel formula = *((n₁*m₁)+(n₂* m₂))/n₁+n₂*
**Combining the standard deviation for similar subgroups** (2)	$SD_c = \sqrt{\frac{\left((n_1 - 1) \times {SD_1}^2\right) + \left((n_2 - 1) \times {SD_2}^2\right) + \left(\frac{n1 \times n2}{n1 + n2}\right) \times \left(({m_1}^2 + {m_2}^2) - (2 \times (m_1 \times m_2))\right)}{(n_1 + n_2) - 1}}$
	Excel formula = *SQRT(((n₁–1)*POWER(SD₁,2))+((n₂–1)*POWER(SD₂,2))+((n₁*n₂)/(n₁+n₂))* ((POWER(m₁,2)+(POWER(m₂,2))-((2*(m₁*m₂))/((n₁+n₂)-1)))))*
**Combining the sample number for similar groups** (2)	$n_C = n_1 + n_2$
	Excel formula =*(n₁+n₂)*

Symbols	
m_1	Group 1 mean
m_2	Group 2 mean
SD_1	Group 1 standard deviation
SD_2	Group 2 standard deviation
q_1	1st quartile
q_3	3rd quartile
n_1	Group 1 sample number
n_2	Group 2 sample number
$\bar{x}_C$	Mean of combined subgroups
SD_C	Standard deviation of combined subgroups
n_C	Sample number of combined subgroups
θ	0.75

References

1. Wan X, Wang W, Liu J, Tong T. Estimating the sample mean and standard deviation from the sample size, median, range and/or interquartile range. BMC Medical Research Methodology. 2014;14(1):135.

2. Julian Higgins JT, Jacqueline Chandler, Miranda Cumpston, Tianjing Li, Mathew Page, Vivian Welch. Cochrane Handbook for Systematic Reviews of Interventions. 2020.

PART VI

EXCLUDED STUDIES

Excluded studies due to reporting on severe COVID-19 cases

Table S6.1. List of studies excluded due to reporting on severe COVID-19 cases (1-14):

1. Qian S-Z, Hong W-d, Lingjie-mao, Chenfeng-lin, Zhendong-fang, Pan J-Y. Clinical Characteristics and Outcomes of Severe and Critical Patients With 2019 Novel Coronavirus Disease (COVID-19) in Wenzhou: A Retrospective Study. Frontiers in Medicine. 2020;7(597).

2. Han H, Xu Z, Cheng X, Zhong Y, Yuan L, Wang F, et al. Descriptive, Retrospective Study of the Clinical Characteristics of Asymptomatic COVID-19 Patients. mSphere. 2020;5(5).

3. Blot M, Jacquier M, Aho Glele LS, Beltramo G, Nguyen M, Bonniaud P, et al. CXCL10 could drive longer duration of mechanical ventilation during COVID-19 ARDS. Crit Care. 2020;24(1):632.

4. Kong Y, Han J, Wu X, Zeng H, Liu J, Zhang H. VEGF-D: a novel biomarker for detection of COVID-19 progression. Crit Care. 2020;24(1):373.

5. Xia Q, Xu K, Yu L, Zhang H, Li L. Application value of artificial liver support system in the treatment of cytokine storm in patients with COVID-19. Int Immunopharmacol. 2020:107120.

6. Song Y, Gao P, Ran T, Qian H, Guo F, Chang L, et al. High Inflammatory Burden: A Potential Cause of Myocardial Injury in Critically Ill Patients With COVID-19. Front Cardiovasc Med. 2020;7:128.

7. Ni M, Tian FB, Xiang DD, Yu B. Characteristics of inflammatory factors and lymphocyte subsets in patients with severe COVID-19. J Med Virol. 2020;92(11):2600-6.

8. Yu B, Li C, Chen P, Zhou N, Wang L, Li J, et al. Low dose of hydroxychloroquine reduces fatality of critically ill patients with COVID-19. Sci China Life Sci. 2020;63(10):1515-21.

9. Notz Q, Schmalzing M, Wedekink F, Schlesinger T, Gernert M, Herrmann J, et al. Pro- and Anti-Inflammatory Responses in Severe COVID-19-Induced Acute Respiratory Distress Syndrome-An Observational Pilot Study. Front Immunol. 2020;11:581338.

10. Zhong Y, Cao Y, Zhong X, Peng Z, Jiang S, Tang T, et al. Immunity and Coagulation/Fibrinolytic Processes may Reduce the Risk of Severe Illness in Pregnant Women with COVID-19. Am J Obstet Gynecol. 2020.

11. Li X, Marmar T, Xu Q, Tu J, Yin Y, Tao Q, et al. Predictive indicators of severe COVID-19 independent of comorbidities and advanced age: a nested case-control study. Epidemiol Infect. 2020;148:e255.

12. Fengmin S, Zhang H, Zhu G, Yan L, Lu Y, Fang Q. The absorbing filter Oxiris in severe COVID-19 patients: A case series. Artif Organs. 2020.

13. Zhang B, Zhou X, Qiu Y, Song Y, Feng F, Feng J, et al. Clinical characteristics of 82 cases of death from COVID-19. PLoS One. 2020;15(7):e0235458.

14. Hong R, Zhao H, Wang Y, Chen Y, Cai H, Hu Y, et al. Clinical characterization and risk factors associated with cytokine release syndrome induced by COVID-19 and chimeric antigen receptor T-cell therapy. Bone Marrow Transplant. 2020:1-11.

Excluded studies due to overlap with included studies

Table S6.2. List of studies excluded due to overlap with included studies (1-21):

1. Zhang B, Zhou X, Zhu C, Song Y, Feng F, Qiu Y, et al. Immune Phenotyping Based on the Neutrophil-to-Lymphocyte Ratio and IgG Level Predicts Disease Severity and Outcome for Patients With COVID-19. Front Mol Biosci. 2020;7:157.

2. Wang F, Hou H, Luo Y, Tang G, Wu S, Huang M, et al. The laboratory tests and host immunity of COVID-19 patients with different severity of illness. JCI Insight. 2020;5(10).

3. Wang F, Yang Y, Dong K, Yan Y, Zhang S, Ren H, et al. Clinical Characteristics of 28 Patients with Diabetes and Covid-19 in Wuhan, China. Endocr Pract. 2020;26(6):668-74.

4. Hou H, Zhang B, Huang H, Luo Y, Wu S, Tang G, et al. Using IL-2R/lymphocytes for predicting the clinical progression of patients with COVID-19. Clin Exp Immunol. 2020;201(1):76-84.

5. Long X, Zhang Z, Zou W, Ling J, Li D, Jing L, et al. Coagulopathy of Patients with COVID-19 is Associated with Infectious and Inflammatory Markers. Risk Manag Healthc Policy. 2020;13:1965-75.

6. Dong Y, Zhou H, Li M, Zhang Z, Guo W, Yu T, et al. A novel simple scoring model for predicting severity of patients with SARS-CoV-2 infection. Transbound Emerg Dis. 2020.

7. Lv Z, Cheng S, Le J, Huang J, Feng L, Zhang B, et al. Clinical characteristics and co-infections of 354 hospitalized patients with COVID-19 in Wuhan, China: a retrospective cohort study. Microbes Infect. 2020;22(4-5):195-9.

8. Zhao Y, Nie HX, Hu K, Wu XJ, Zhang YT, Wang MM, et al. Abnormal immunity of non-survivors with COVID-19: predictors for mortality. Infect Dis Poverty. 2020;9(1):108.

9. Bao Y, Ling Y, Chen Y-Y, Tian D, Zhao G-P, Zhang X-H, et al. Dynamic anti-spike protein antibody profiles in COVID-19 patients. International journal of infectious diseases : IJID : official publication of the International Society for Infectious Diseases. 2021;103:540-8.

10. Xiong L, Zang X, Feng G, Zhao F, Wang S, Zeng W, et al. Clinical characteristics and peripheral immunocyte subsets alteration of 85 COVID-19 deaths. Aging. 2021;13.

11. Qian F, Gao G, Song Y, Xu Y, Wang A, Wang S, et al. Specific dynamic variations in the peripheral blood lymphocyte subsets in COVID-19 and severe influenza A patients: a retrospective observational study. BMC Infect Dis. 2020;20(1):910.

12. Chen X, Huang J, Huang Y, Chen J, Huang Y, Jiang X, et al. Characteristics of immune cells and cytokines in patients with coronavirus disease 2019 in Guangzhou, China. Hum Immunol. 2020.

13. Wan S, Yi Q, Fan S, Lv J, Zhang X, Guo L, et al. Relationships among lymphocyte subsets, cytokines, and the pulmonary inflammation index in coronavirus (COVID-19) infected patients. Br J Haematol. 2020;189(3):428-37.

14. Chen G, Wu D, Guo W, Cao Y, Huang D, Wang H, et al. Clinical and immunological features of severe and moderate coronavirus disease 2019. J Clin Invest. 2020;130(5):2620-9.

15. Gan J, Li J, Li S, Yang C. Leucocyte Subsets Effectively Predict the Clinical Outcome of Patients With COVID-19 Pneumonia: A Retrospective Case-Control Study. Front Public Health. 2020;8:299-.

16. Gao M, Liu Y, Guo M, Wang Q, Wang Y, Fan J, et al. Regulatory CD4(+) and CD8(+) T cells are negatively correlated with CD4(+) /CD8(+) T cell ratios in patients acutely infected with SARS-CoV-2. J Leukoc Biol. 2020.

17. Guo H, Zheng J, Huang G, Xiang Y, Lang C, Li B, et al. Xuebijing injection in the treatment of COVID-19: a retrospective case-control study. Ann Palliat Med. 2020;9(5):3235-48.

18. Qun S, Wang Y, Chen J, Huang X, Guo H, Lu Z, et al. Neutrophil-to-Lymphocyte Ratios Are Closely Associated With the Severity and Course of Non-mild COVID-19. Front Immunol. 2020;11:2160.

19. Tang Y, Li Y, Sun J, Pan H, Yao F, Jiao X. Selection of an Optimal Combination Panel to Better Triage COVID-19 Hospitalized Patients. J Inflamm Res. 2020;13:773-87.

20. Zhang Q, Wei Y, Chen M, Wan Q, Chen X. Clinical analysis of risk factors for severe COVID-19 patients with type 2 diabetes. J Diabetes Complications. 2020;34(10):107666.

21. Zhang L, Han C, Zhang S, Duan C, Shang H, Bai T, et al. Diarrhea and altered inflammatory cytokine pattern in severe coronavirus disease 2019: Impact on disease course and in-hospital mortality. J Gastroenterol Hepatol. 2020:9.

PART VII

GENERAL CHARACTERISTICS OF INCLUDED STUDIES

General characteristics of included studies

Table S7.1. Classification protocol.

Classification protocol	
**Study**	**Protocol**
Abers, Michael 2021	National Health Commission of China
Azmy, Veronica 2021	National Guidelines
Cantenys-Molina, S 2021	National Guidelines
Carissimo, Guillaume 2020	WHO
Chen, Jiaxin 2020	National Health Commission of China (version 6)
Chi, Ying 2020	National Health Commission of China
Deng, Fuxue 2020	National Health Commission of China (version 5)
Diao, Bo 2020	National Health Commission of China (version 5)
Feng, Xiaobo 2020	WHO NCP Interim Guidelines (7th Edition)
Flament, Heloise 2021	National Guidelines
Gadotti, Ana Carolina 2020	WHO
Guan, Jingjing 2020	National Health Commission of China (version 7)
Han, Huan 2020	National Guidelines
He, Bing 2020	Specified for mild and severe cases
He, Susu 2020	WHO NCP Interim Guidelines
Henry, Brandon Michael 2021	National Guidelines
Huang, Hong 2021	National Health Commission of China (version 5)
Huang, Wei 2021	WHO NCP Interim Guidelines (6th Edition)
Hue, Sophie 2020	National Guidelines
Jin, Xiao-Hong 2020	National Health Commission of China (version 7)
Keddie, Stephen 2020	WHO COVID-19 Guidelines
Kwon, Ji-Soo 2020	WHO COVID-19 Guidelines
Laing, Adam 2020	WHO COVID-19 Guidelines
Li, Chenze 2020	National Health Commission of China
Li, Mingyue 2020	National Health Commission of China
Li, Qiang 2020	Chinese CDC Protocol
Li, Xiaolei 2020	WHO NCP Interim Guidelines
Liao, Baolin 2021	National Health Commission of China (version 7)
Liu, Fangfang 2020	National Health Commission of China (version 6)
Liu, Jian 2020	WHO NCP Interim Guidelines
Liu, Jing 2020	Chinese CDC Protocol
Liu, Lei 2020	WHO NCP Interim Guidelines
Liu, Yangli 2021	National Health Commission of China (version 7-15)
Liu, Xue-Qing 2021	National Health Commission of China (version 7)
Luo, Miao 2020	WHO NCP Interim Guidelines
Mann, Elizabeth 2020	CIRCO
McElvaney, Oliver 2020	Specified for ICU and stable patients
Rendeiro, Andre 2020	National Guidelines
Schrijver, Benjamin 2020	National Guidelines
Shi, Hongbo 2020	National Health Comission of China
Tan, Mingkai 2020	National Health Commission of China (version 6)

Wang, Zhongliang 2020	National Guidelines
Xu, Bo 2020	CDC and WHO Guidelines
Yang, Ai-Ping 2020	WHO NCP Interim Guidelines
Yang, Fan 2020	National Health Commission of China (version 7)
Yi, Ping 2020	National Health Commission of China (version 3-7)
Zeng, Hao-Long 2020	National Health Commission of China (version 7)
Zeng, Zhilin 2020	National Health Commission of China (version 6)
Zhang, Bo 2021	National Health Commission of China (version 7)
Zhang, Jun 2020	WHO COVID-19 Guidelines
Zhao, Yan 2020	National Health Commission of China
Zou, Li 2020	National Health Commission of China (version 3-7)

Table S7.2. Sample acquisition time and test procedure.

Sample acquisition time and test procedure						
Study	CD4 and CD8 T-cells			IL-10		
	Sample acquisition time	Procedure/Test	Value	Sample acquisition time	Procedure/Test	Value
Abers, Michael 2021	N/A	N/A	N/A	Within 7 days of admission	MESO QuickPlex SQ 120 (Meso Scale Discovery)	pg/mL
Azmy, Veronica 2021	N/A	N/A	N/A	Admission	Multiplex bead assay	pg/mL
Cantenys-Molina, S 2021	Admission	Four-color flow cytometry single platform (BD Biosciences)	Cells/microliter	N/A	N/A	N/A
Carissimo, Guillaume 2020	Admission	BD LSRII 5 (BD Biosciences)	x10^2/microliter	N/A	N/A	N/A
Chen, Jiaxin 2020	Admission	Not specified/clinical records	Cells/microliter	N/A	N/A	N/A
Chi, Ying 2020	N/A	N/A	N/A	1-11 days post admission	Luminex 200 (Luminex)	pg/mL
Deng, Fuxue 2020	N/A	N/A	N/A	Admission	Not specified/clinical records	pg/mL
Diao, Bo 2020	Not specified	Not specified/clinical records	x10^6/L	Not specified	Not specified/clinical records	pg/mL
Feng, Xiaobo 2020	Admission	Not specified/clinical records	%	Admission	Not specified/clinical records	pg/mL
Flament, Heloise 2021	Not specified	LSRF Fortesa x-20, FACSARIA III Cell Sorter (BD Biosciences)	%	Not specified	FACSLyric (BD Biosciences)	pg/mL
Gadotti, Ana Carolina 2020	N/A	N/A	N/A	Admission	ELISA kit for IL-10	pg/mL
Guan, Jingjing 2020	1-3 days post admission	FACSCanto II (BD Biosciences)	%	1-3 days post admission	FACSCanto II (BD Biosciences)	pg/mL
Han, Huan 2020	N/A	N/A	N/A	Admission	FACSCalibur (BD Biosciences)	pg/mL
He, Bing 2020	Admission	FACSCanto II (BD Biosciences)	x10^9/microliter	Admission	Th1/Th2 Kit for IL-10 (BD Biosciences)	pg/mL
He, Susu 2020	Admission	Not specified/clinical records	x10^9/microliter	Admission	Not specified/clinical records	pg/mL
Henry, Brandon Michael 2021	N/A	N/A	N/A	Admission	U-Plex assay (Meso Scale Discovery)	pg/mL
Huang, Hong 2021	N/A	N/A	N/A	Not specified	Not specified/clinical records	pg/mL
Huang, Wei 2021	Admission	Navios (Beckman Coulter)	Cells/microliter	Admission	Immulite 1000 (Siemens)	pg/mL
Hue, Sophie 2020	48 hours post ICU admission	10 multi-colour (Beckman Coulter)	Cells/mm3	48 hours post ICU admission	Luminex Multiplex (R&D Systems)	pg/mL
Jin, Xiao-Hong 2020	Admission	Not specified/clinical records	Cells/microliter	Admission	Not specified/clinical records	pg/mL
Keddie, Stephen 2020	N/A	N/A	N/A	Admission	Four-PLEX ELISA (Meso Scale Discovery)	pg/mL
Kwon, Ji-Soo 2020	N/A	N/A	N/A	Admission	FACSCanto II (BD Biosciences)	pg/mL
Laing, Adam 2020	Admission	LSR Fortress, FACSAria III cell sorter (BD Biosciences)	Cells/microliter	Admission	LSR Fortress x20 (BD Biosciences)	pg/mL
Li, Chenze 2020	N/A	N/A	N/A	Admission	Not specified/clinical records	pg/mL
Li, Mingyue 2020	1-3 days post admission	FACSCanto II (BD Biosciences)	%	1-3 days post admission	FACSCanto II (BD Biosciences)	pg/mL
Li, Qiang 2020	Admission	Not specified/clinical records	Cells/microliter	Admission	Not specified/clinical records	pg/mL
Li, Xiaolei 2020	Admission	FC 500 (Beckman Coulter)	x10^9/microliter	N/A	N/A	N/A
Liao, Baolin 2021	2nd week after onset of symptoms	FACS Diva (BD Biosciences)	x10^6/L	2nd week after onset of symptoms	Th1/Th2 Kit for IL-10 (Weimi BioTech)	pg/mL
Liu, Fangfang	Admission	Not specified/clinical records	Cells/microliter	N/A	N/A	N/A
Liu, Jian 2020	N/A	N/A	N/A	Admission	ELISA/not specified	pg/mL
Liu, Jing 2020	Admission	Not specified/clinical records	x10^9/L	Admission	Not specified/clinical records	pg/mL
Liu, Lei 2020	N/A	N/A	N/A	Not specified	Not specified/clinical records	pg/mL
Liu, Yangli 2021	N/A	N/A	N/A	Admission	FACSCanto II (BD Biosciences)	pg/mL
Liu, Xue-Qing 2021	N/A	N/A	N/A	Admission	Not specified/clinical records	pg/mL
Luo, Miao 2020	First 3 days of hospitalization	FACS Diva (BD Biosciences)	Cells/microliter	First 3 days of hospitalization	Immulite (DiaSorin)	pg/mL
Mann, Elizabeth 2020	Admission	LSRF Fortesa x20 (BD Biosciences)	%	Admission	LEGENDplex assays (BioLegend)	pg/mL
McElvaney, Oliver 2020	N/A	N/A	N/A	7-days post admission	ELISA (R&D)	pg/mL
Rendeiro, Andre 2020	Admission	FACSCanto (BD Biosciences)	%	N/A	N/A	N/A
Schrijver, Benjamin 2020	Admission	FACSCanto (BD Biosciences)	Cells/microliter	Admission	Luminex Multiplex (R&D Systems)	pg/mL
Shi, Hongbo 2020	Admission	Helios mass cytometer (Fluidigm)	Cells/microliter	Admission	Luminex Multiplex (R&D Systems)	pg/mL
Tan, Mingkai 2020	Admission	FACSCanto Plus (BD Biosciences)	Cells/microliter	Admission	FACSCanto Plus (BD Biosciences)	pg/mL
Wang, Zhongliang 2020	Admission	Not specified/clinical records	%	Admission	Not specified/clinical records	pg/mL
Xu, Bo 2020	Admission	Not specified/clinical records	x10^9/microliter	Admission	Not specified/clinical records	pg/mL
Yang, Ai-Ping 2020	Not specified	Not specified/clinical records	Cells/microliter	Not specified	Not specified/clinical records	pg/mL
Yang, Fan 2020	Not specified	Not specified/clinical records	Cells/microliter	N/A	N/A	N/A
Yi, Ping 2020	Admission	FACS Calibur (BD Biosciences)	Cells/microliter	Admission	Human cytokine standard 27-plex assay and Bio-PLEX 200 system (Bio-I	pg/mL
Zeng, Hao-Long 2020	N/A	N/A	N/A	Admission	Immulite 1000 (Siemens)	pg/mL
Zeng, Zhilin 2020	N/A	N/A	N/A	Admission	Immulite (DiaSorin)	pg/mL
Zhang, Bo 2021	Admission	FACSCanto (BD Biosciences)	x10^6/L	N/A	N/A	N/A
Zhang, Jun 2020	N/A	N/A	N/A	Admission	Not specified/clinical records	pg/mL
Zhao, Yan 2020	N/A	N/A	N/A	4-7 days post admission	Not specified/clinical records	pg/mL
Zou, Li 2020	Admission	Not specified/clinical records	x10^9/microliter	Admission	Not specified/clinical records	pg/mL

Table S.7.3. Reported comorbidities from included studies.

Reported comorbidities	
**Study**	**Comorbidity: n (%)**
Abers, Michael 2021	Hypertension: 64 (37.2%); Diabetrs mellitus: 36 (20.9%); Cardiovascular disease: 32 (18.6%); Autoimmune disease: 29 (16.9%); Neurologic disease: 25 (14.5%); Chronic respiratory disease: 15 (8.7%); Thyroid disease: 15 (8.7%); Solid organ malignancy: 15 (8.7%); Chronic kidney disease: 14 (8.1%); Hyperlipidemia: 13 (7.6); Chronic gastrointestinal disease: 13 (7.6%); Chronic liver disease: 8 (4.7%); Asthma: 5 (2.9%); Benign hematologic disease: 8 (2.9%); Solid organ transplantation: 4 (2.3%).
Azmy, Veronica 2021	Diabetes: 91 (38%); Uncontrolled diabetes: 40 (17%); Immunosuppressed: 36 (15%); Chronic lung disease: 91 (38%); Hypertension: 142 (59%); Chronic heart disease: 71 (30%).
Cantenys-Molina, S 2021	Hypertension: 305 (43.5%); Dyslipidemia: 224 (32%); Diabetes mellitus: 123 (17.5%); Cardiovascular disease: 128 (18.3%); COPD: 44 (6.3%); Malignancy: 81 (11%); Kidney disease: 44 (6.3%).
Carissimo, Guillaume 2020	Diabetes: 9 (16.7%); Hypertension: 14 (25.9%).
Chen, Jiaxin 2020	Not specified.
Chi, Ying 2020	Diabetes: 4 (6%); Hypertension: 10 (15%); Cardiac disease: 2 (3%); Renal disease: 1 (1.5%); Liver disease: 1 (1.5%); Lung disease: 2 (3%).
Deng, Fuxue 2020	Not specified.
Diao, Bo 2020	Not specified.
Feng, Xiaobo 2020	Hypertension: 62 (54.4%); Diabetes: 39 (24.2%); Hyperlipidemia: 17 (14.9); Cardiovascular disease: 31 (27.2%); Cerebrovascular disease: 6 (5.3%); Renal disease: 6 (5.3%) Liver disease: 4 (3.5%); COPD: 11 (9.6); Cancer: 10 (8.8%); Neuropsychiatric disorder: 3 (2.6%).
Flament, Heloise 2021	Diabetes: 23 (24.7%)
Gadotti, Ana Carolina 2020	Hypertension: 28 (50%); Diabetes: 14 (25%); Chronic heart faulure: 6 (10.7%); Chronic coronary disease: 7 (12.5%); Arrhytmia: 5 (8.9%); Asthma: 5 (8.9%); Chronic pulmonary disease: 9 (16.1%); Dilipidemia: 4 (7.1%); Chronic renal failure: 2 (3.6%).
Guan, Jingjing 2020	Not specified.
Han, Huan 2020	Not specified.
He, Bing 2020	Diabetes: 3 (5.6%); Cardiovascular and cerebrovascular: 8 (15%); Respiratory system disease: 1 (1.8%); Chronic renal disease: 2 (3.7%); Chronic liver disease: 1 (1.8%); Nervous system disease: 1 (1.8%); Malignant tumor: 1 (1.8%).
He, Susu 2020	Hypertension: 14 (15%); Diabetes: 7 (8%); COPD: 2 (2%).
Henry, Brandon Michael 2021	Coronary artery disease: 8 (15.4%); Heart failure: 9 (17.3%); Hypertension: 26 (50%); Hyperlipidemia: 15 (28.8%); Diabetes: 21 (40.4%); COPD: 8 (15.4%); Asthma: 8 (15.4%); Chronic kidney disease: 6 (11.5%); Chronic liver disease: 7 (13.5%); Cerebrovascular disease: 7 (13.5%); Cancer: 4 (8%).
Huang, Hong 2020	Not specified.
Huang, Wei 2021	Hypertension: 65 (30%); Diabetes: 39 (18%); COPD: 7 (3%); Cardiovascular disease: 26 (12%); Chronic liver disease: 1 (0.5%); Malignancy: 7 (3%).
Hue, Sophie 2020	Diabetes: 12 (32%); Chronic heart failure: 6 (16%); COPD: 5 (13%); Sickle cell anemia: 1 (3%).
Jin, Xiao-Hong 2020	Chronic heart disease: 1 (0.6%); Diabetes: 18 (12.3%); Hypertension: 23 (15.7%); Chronic renal disease: 2(1.3%); Chronic liver disease: 10 (6.8%); Chronic lung disease: 8 (5.4%); Cancer: 3 (18.7%).
Keddie, Stephen 2020	Hypertension: 37 (37%); Diabetes: 25 (25%); Cardiovascular disease: 12 (12%); Cerebrovascular disease: 8 (8%); Malignancy: 10 (10%).
Kwon, Ji-Soo 2020	Hypertension: 9 (29%); Diabetes: 4 (13%); COPD: 2 (6%).
Laing, Adam 2020	Hpertesion: 27 (42.9%); Diabetes: 17 (27%); Chronic lung disease: 8 (12.7%); Asthma: 11 (17.5); Ischaemic heart disease: 8 (12.7%); Cholesterolaemia: 5 (7.9%); Malignancy: 9 (14.3%).
Li, Chenze 2020	Hypertension: 722 (34.9); Diabetes: 292 (14.1%); Coronary heart disease: 182 (8.8%); COPD: 32 (1.5%); Renal disease: 31 (1.5%); Cancer: 75 (3.6%).
Li, Mingyue 2020	Comorbidities: 15 (46.9%); Not specified.
Li, Qiang 2020	Hypertension: 8 (25.8%); Diabetes mellitus: 8 (25.8%); Chronic pulmpnary disease: 3 (9.7%); Cardiovascular disease: 7 (22.6%); Cerebrovascular disease: 2 (6.5%); Chronic kidney disease: 2 (6.5%); Malignancy: 1 (3.2%).

Li, Xiaolei 2020	Not specified
Liao, Baolin 2021	Comorbidities: 58 (36.7%); Not specified.
Liu, Fangfang 2020	22 patients (33.85%) had at least one concurrent disease (i.e. hypertension, diabetes, malignancy, endocrine disease, and tumor).
Liu, Jian 2020	Diabetes: 1 (4%); Hypertension: 8 (35%); Valvular heart disease, 1 (4%); Liver transplantation: 1 (4%).
Liu, Jing 2020	Diabetes: 6 (15%); Hypertension: 6 (15%); Pituitary adenoma: 2 (5%); Thyroid disease: 2 (5%); Malignancy: 2 (5%).
Liu, Lei 2020	Diabetes: 35 (11.9%); Hypertension: 70 (23%); Cardio-cerebrovascular disease: 42 (14.3%); Respiratory disease: 16 (5.4%); Malignancy: 24 (8.2%).
Liu, Yangli 2021	Hypertension: 4 (40%); Diabetes melitus: 1 (10%); Malignancy: 1 (10%).
Liu, Xue-Qing 2021	Hypertension: 50 (49.5); Diabetes: 25 (24.8%); COPD: 9 (8.9%); Heart disease: 22 (21.8%); Chronic liver disease: 11 (10.9%); Chronic renal disease: 18 (17.8%); Cerebrovascular diseases: 13 (8.9%); Rheumatism: 2 (2%).
Luo, Miao 2020	Diabetes: 164 (16.1%); Hypertension: 365 (35.9%); Coronary heart disease: 83 (8.2%); Pulmonary disease: 56 (5.5%).
Mann, Elizabeth 2020	Hypertension: 14 (28.6%); Diabetes: 8 (16.3%); Ischemic heart disease: 5 (10.2%); COPD: 9 (18.4%); Asthma: 5 (10.2%); Malignancy: 3 (6.1%).
McElvaney, Oliver 2020	Diabetes: 8 (20%); Hypertension: 16 (40%); Coronary artery disease: 7 (18%); Chronic lung disease: 11 (28%); Chronic renal disease: 9 (23%).
Rendeiro, Andre 2020	Hypertension: 16 (16.67%); Diabetes: 9 (25%)
Schrijver, Benjamin 2020	Diabetes: 8 (18.8%); COPD: 9 (20.45%); Heart Failure: 7 (15.91%); Kidney failure: 2 (4.5%).
Shi, Hongbo 2020	Not specified.
Tan, Mingkai 2020	Not specified.
Wang, Zhongliang 2020	Hypertension: 9 (13%); Cardiovascular disease: 8 (12%); Diabetes: 7 (10%); COPD: 4 (6%); Asthma: 2 (3%); Chronic hepatitis: 1 (1%); Malignancy: 4 (6%).
Xu, Bo 2020	Hypertension: 50 (26.7%); Cardio-cerebrovascular disease: 19 (10.2%); Lung disease: 3 (1.6%); Metabolic: 21 (11.2%).
Yang, Ai-Ping 2020	Diabetes: 21 (22.5%); Hypertension: 23 (24.7%); Heart disease: 13 (13.9%); Renal disease: 10 (10.7); Abnormal liver function: 13 (13.9).
Yang, Fan 2020	Not specified.
Yi, Ping 2020	Hypertension: 37 (37%); Diabetes mellitus: 11 (11%); Cardiac disease: 4 (4%).
Zeng, Hao-Long 2020	Hypertension: 226 (35.2%); Diabetes: 97 (15.11%); Coronary heart disease: 61 (9.5%); Chronic lung disease: 43 6.7%); Chronic kidney disease: 21 (3.27%); Cerebrovascular disease: 17 (2.65%); Chronic liver disease: 11 (1.71%).
Zeng, Zhilin 2020	Hypertension: 124 (39.1%); Coronary artery disease (30 (9.5%); Diabetes mellitus: 63 (19.9%); Chronic kidney disease: 4 (1.3%); Tumor: 6 (1.9%).
Zhang, Bo 2021	Hypertension: 268 (37.9%); Diabetes: 135 (19.1%); Cardiovascular disease: 79 (11.1%); Malignancy: 31 (4.4%); Chronic kidney disease: 29 (3.8%); Chronic liver disease: 27 (3.8%); COPD: 24 (3.4%); Thyroid disease: 26 (3.7%).
Zhang, Jun 2020	Hypertension: 15 (13.5%); Cardiovascular disease: 3 (2.7%); COPD: 3 (2.7%); Diabetes: 14 (12.6%); Chronic liver disease: 1 (0.9%); Malignancy: 8 (7.2%).
Zhao, Yan 2020	Diabetes: 5 (7%); Hypertension: 13 (18.3%); Cardiovascular disease: 8 (11.27%); Respiratory disease: 3 (4.2%); Renal disease: 1 (1.4%); Liver disease: 4 (5.6%).
Zou, Li 2020	Diabetes: 25 (20.7%); Hypertension: 49 (40.5%); Cardio-cerebrovascular disease: 27 (22.3%); Chronic pulmonary diseases: 19 (15.7%); Cancer: 10 (8.3%).

Table S7.4. Subgroup assignment.

Subgroup assignment				
**Study**	**COVID-19 subgroups assigned by study**	**Classified under mild or survivors**	**Classified under severe or non-survivors**	**Outcome**
Abers, Michael 2021	Moderate vs Severe vs Critical	Moderate	Severe + Critical	Severity
Azmy, Veronica 2021	Moderate vs Severe vs Critical	Moderate	Severe + Critical	Severity
Cantenys-Molina, S 2021	Alive vs Dead	Alive	Dead	Mortality
Carissimo, Guillaume 2020	Recovered vs Acute	Recovered	Acute	Severity
Chen, Jiaxin 2020	Mild vs Ordinary vs Severe vs Critical	Mild + Ordinary	Severe + Critical	Severity
Chi, Ying 2020	Mid vs Moderate vs Severe	Mild + Moderate	Severe	Severity
Deng, Fuxue 2020	Moderate vs Severe vs Critical	Moderate	Serious + Critical	Severity
Diao, Bo 2020	Non-ICU vs ICU	Non-ICU	ICU	Severity
Feng, Xiaobo 2020	Good vs Poor outcome	Good Outcome	Bad Outcome	Severity
Flament, Heloise 2021	Infectious Disease Unit (IDU) vs Intensive Care Unit (ICU)	Infectious Disease Unit (IDU)	Intensive Care Unit (ICU)	Severity
	Infectious Disease Unit (IDU) vs Intensive Care Unit (ICU) vs Deceased	Infectious Disease Unit (IDU) + Intensive Care Unit (ICU)	Deceased	Mortality
Gadotti, Ana Carolina 2020	Survival vs Death	Survival	Death	Mortality
Guan, Jingjing 2020	Mild vs Severe	Mild	Severe	Severity
Han, Huan 2020	Moderate vs Severe vs Critical	Moderate	Severe + Critical	Severity
He, Bing 2020	Mild vs Severe	Mild	Severe	Severity
He, Susu 2020	Mild vs Severe: including Critical	Mild	Severe	Severity
Henry, Brandon Michael 2021	Mild/Moderate vs Severe	Mild + Moderate	Severe	Severity
Huang, Hong 2020	Alive vs Dead	Alive	Dead	Mortality
Huang, Wei 2021	Moderate vs Severe vs Critical	Moderate	Severe + Critical	Severity
Hue, Sophie 2020	Survival vs Death	Survival	Death	Mortality
Jin, Xiao-Hong 2020	Non-severe vs Severe	Non-severe	Severe	Severity
Keddie, Stephen 2020	WHO 3 Hospitalised vs WHO 4 & 5 Oxygen NIV vs WHO 6 & 7 Ventilated	WHO 3 Hospitalised + WHO 4 & 5 Oxygen NIV	WHO 6 & 7 Ventilated	Severity
Kwon, Ji-Soo 2020	Mild vs Moderate vs Severe	Mild + Moderate	Severe	Severity
Laing, Adam 2020	Low vs Moderate vs Severe	Low + Moderate	Severe	Severity
Li, Chenze 2020	A. Non-Critical vs Critical	Non-Critical	Critical	Severity
	B. Surivial vs Death	Survival	Death	Mortality
Li, Mingyue 2020	Mild vs Severe	Mild	Severe	Severity
Li, Qiang 2020	Survival vs Death	Survival	Death	Mortality
Li, Xiaolei 2020	Non-Severe including Mild vs Severe	Non-Severe	Severe	Severity
Liao, Baolin	Mid vs Moderate vs Severe	Mild + Moderate	Severe	Severity
Liu, Fangfang 2020	Mild vs General vs Severe vs Critical	Mild + General	Severe + Critical	Severity
Liu, Jian 2020	ICU not requiring MV vs ICU requiring MV	ICU not requiring MV	ICU requiring MV	Severity
Liu, Jing 2020	Mild vs Severe	Mild	Severe	Severity
Liu, Lei 2020	Non-Severe vs Severe	Non-Severe	Severe	Severity
Liu, Yangli 2021	Moderate vs Severe vs Critical	Moderate	Severe + Critical	Severity
Liu, Xue-Qing 2021	Moderate vs Severe vs Critical	Moderate	Severe + Critical	Severity
Luo, Miao 2020	Survivors vs Non-Survivors	Survivors	Non-Survivors	Mortality
Mann, Elizabeth 2020	Mild vs Moderate vs Severe	Mild + Moderate	Severe	Severity
McElvaney, Oliver 2020	Stable vs ICU vs CAP ICU (7-days after admission)	Stable	ICU + CAP ICU	Severity
Rendeiro, Andre 2020	Mild vs Severe	Mild	Severe	Severity
Schrijver, Benjamin 2020	Favorable vs Unfavorable	Favorable	Unfavorable	Severity
Shi, Hongbo 2020	Common vs Severe vs Critical	Common	Severe + Critical	Severity
Tan, Mingkai 2020	Mild vs Severe	Mild	Severe	Severity
Wang, Zhongliang 2020	SpO2 ≥ 90% vs SpO2 < 90%	SpO2 ≥ 90%	SpO2 < 90%	Severity
Xu, Bo 2020	A. Discharged vs Stay in hospital	Discharged	Stay in hospital	Severity
	B. Discharged vs Died vs Stay in Hospital	Discharged + Stay in Hospital	Died	Mortality
Yang, Ai-Ping 2020	Non-Severe vs Severe: including Critical	Non-Severe	Severe	Severity
Yang, Fan 2020	Mild vs Severe	Mild	Severe	Severity
Yi, Ping 2020	Non-severity vs severity	Non-severity	Severity	Severity
Zeng, Hao-Long 2020	Hospitalized vs Discharged vs Died	Discharged + Hospitalized	Died	Mortality
Zeng, Zhilin 2020	Moderate vs Severe vs Critical	Moderate	Severe + Critical	Severity
Zhang, Bo 2021	Moderate vs Severe vs Critical	Moderate	Severe + Critical	Severity
Zhang, Jun	Discharge vs Deterioation	Discharge	Deterioation	Severity
Zhao, Yan 2020	Mild vs Severe	Mild	Severe	Severity
Zou, Li 2020	A. Non-Severe vs Severe	Non-Severe	Severe	Severity
	B. Survival vs Death	Survival	Death	Mortality

PART VIII

POPULATION CHARACTERISTICS

Population characteristics

Table S8.1. Sex and total number of COVID-19 patients based on severity.

Sex and total number of COVID-19 patients based on severity						
**Study**	**Mild**			**Severe**		
	**Total**	**Male**	**Female**	**Total**	**Male**	**Female**
Abers, Michael 2021*	30	-	-	145	-	-
Azmy, Veronica 2021	114	54	60	125	72	53
Carissimo, Guillaume 2020	28	19	9	54	50	4
Chen, Jiaxin 2020	567	302	265	31	20	11
Chi, Ying 2020	58	32	26	8	5	3
Deng, Fuxue 2020	17	6	11	83	52	51
Diao, Bo 2020*	212	-	-	43	-	-
Feng, Xiaobo 2020	95	58	36	20	13	7
Flament, Heloise 2021	51	34	17	42	9	51
Guan, Jingjing 2020	42	25	17	19	13	6
Han, Huan 2020	42	20	22	60	30	30
He, Bing 2020	32	15	17	21	13	8
He, Susu 2020	60	31	29	33	18	15
Henry, Brandon Michael 2021	21	20	16	92	62	30
Huang, Wei 2021	116	49	67	102	57	45
Jin, Xiao-Hong 2020	106	58	48	40	19	21
Keddie, Stephen 2020	24	17	7	76	30	46
Kwon, Ji-Soo 2020	23	9	14	8	4	4
Laing, Adam 2020	32	18	14	31	25	6
Li, Chenze 2020	1592	723	869	476	282	194
Li, Mingyue 2020	16	7	9	16	13	3
Li, Xiaolei 2020	159	91	68	56	36	20
Liao, Baolin 2021	124	65	61	34	23	11
Liu, Fangfang 2020	42	24	18	23	12	11
Liu, Lei 2020	27	8	19	13	7	6
Liu, Jing 2020	27	8	19	13	7	6
Liu, Jian 2020	9	8	1	14	10	4
Liu, Lei 2020	202	100	102	92	62	30
Liu, Yangli 2021	10	6	4	57	33	24
Liu, Xue-Qing 2021	47	21	26	54	35	19
Mann, Elizabeth 2020	39	24	15	10	7	3
McElvaney, Oliver 2020	20	12	8	20	13	7
Rendeiro, Andre 2020	21	12	9	15	10	5
Schrijver, Benjamin 2020	33	21	12	11	8	3
Shi, Hongbo 2020	34	14	20	20	8	12
Wang, Zhongliang 2020	55	25	30	14	7	7
Tan, Mingkai 2020	31	17	14	25	18	7
Xu, Bo 2020	80	30	50	107	73	34
Yang, Ai-Ping 2020	69	38	31	24	18	6
Yang, Fan 2020*	33	-	-	19	-	-
Yi, Ping 2020	51	25	26	49	38	11
Zeng, Zhilin 2020	93	41	52	224	121	103
Zhang, Bo 2021	410	181	229	297	162	135
Zhang, Jun 2020	93	32	61	18	14	4
Zhao, Yan 2020	53	23	30	18	7	11
Zou, Li 2020	69	34	35	52	32	20
**Total****	**5109**	**2357**	**2493**	**2804**	**1548**	**1087**

* Total number of patients based on sex (male/female) were not reported.
** Total number of patients based on sex is reported as an approximate number, this is because the male/female size from three studies were not reported.

Table S8.2. Mean age of COVID-19 patients based on severity.

Mean age of COVID-19 patients based on severity				
Study	Mild		Severe	
	Mean age	SD	Mean age	SD
Abers, Michael 2021*	–	–	–	–
Azmy, Veronica 2021	60.66	57.06	60.26	53.02
Carissimo, Guillaume 2020	51.33	14.84	49.26	17.36
Chen, Jiaxin 2020	45.14	17.39	60.84	13.55
Chi, Ying 2020	41.76	14.64	54	12.38
Deng, Fuxue 2020	47	17.78	68.15	13.62
Diao, Bo 2020	50.03	68.67	62.75	13.61
Feng, Xiaobo 2020	62.85	13.65	69.15	11.08
Flament, Heloise 2021	61	16.4	57.6	13.3
Guan, Jingjing 2020	53.02	13.78	65.16	14.94
Han, Huan 2020	58.3	12.6	60.84	14.48
He, Bing 2020	41.33	21.72	58	15.1
He, Susu 2020	33.66	-24.3	40.13	32.08
Henry, Brandon Michael 2021	47	17.75	63.33	14.63
Huang, Wei 2021	56	15.76	68.07	12.55
Jin, Xiao-Hong 2020	43.33	57.87	55.66	46.13
Keddie, Stephen 2020	56.33	55.16	60	53.73
Kwon, Ji-Soo 2020	42.4	19.02	71.9	12.9
Laing, Adam 2020	58.82	25.96	58.33	12.43
Li, Chenze 2020	59.33	14.09	69	13.38
Li, Mingyue 2020	58.33	14.63	62.66	26.01
Li, Xiaolei 2020	42.66	14.96	49.5	39.56
Liao, Baolin 2021	43.97	22.88	62.7	14.2
Liu, Fangfang 2020*	-	-	-	-
Liu, Lei 2020	47	17.75	63.33	14.63
Liu, Jing 2020	43.2	12.3	59.7	10.1
Liu, Jian 2020	71.58	20.33	60.5	17.71
Liu, Lei 2020	50.6	21.13	63.1	12.57
Liu, Yangli 2021	46	22.79	64.51	11.52
Liu, Xue-Qing 2021	51.5	16.44	71.5	14.07
Mann, Elizabeth 2020	59.48	17.76	63.5	17.63
McElvaney, Oliver 2020	56.6	17.3	54.3	18.2
Rendeiro, Andre 2020	64	41.34	65	24.5
Schrijver, Benjamin 2020	69	11	78	6
Shi, Hongbo 2020	50	15	22.66	4.11
Wang, Zhongliang 2020	40	14.46	69.83	12.35
Tan, Mingkai 2020*	44.5	–	66	–
Xu, Bo 2020	55.66	17.36	64.23	16.53
Yang, Ai-Ping 2020	42.1	18.6	57.9	11.8
Yang, Fan 2020*	–	–	–	–
Yi, Ping 2020	48	16.78	60.66	14.51
Zeng, Zhilin 2020	57.83	16.94	62.36	14.3
Zhang, Bo 2021	55	16.36	66.07	12.57
Zhang, Jun 2020	38.18	12.42	63.33	26.54
Zhao, Yan 2020	44.83	16.38	63.91	18.3
Zou, Li 2020	60	12.11	70.25	13.91
**Total****	**53.03**	**25.6**	**63.39**	**22.61**

*Abers, Michael 2021: Reported the age of each individual patient separately. Liu, Fangfang 2020: reported age as <65 or >65. Tan, Mingkai 2020: reported age with no SD values. Yang, Fan 2020: did not report mean age or SD values.

** Mean age indicates an approximate number; this is because the mean age from four studies were not calculated due to missing mean age or SD values. Mean age and SD are calculated using the Cochrane's formula for combining subgroups.

Table S8.3. Sex and total number of COVID-19 patients based on mortality.

Sex and total number of COVID-19 patients based on mortality						
Study	Survivors			Non-Survivors		
	Total	Male	Female	Total	Male	Female
Cantenys-Molina, S 2021	590	334	256	112	71	41
Flament, Héloïse 2021*	44	–	–	55	–	–
Gadotti, Ana Carolina 2020	38	23	15	18	16	2
Huang, Hong 2020	40	18	22	10	5	5
Hue, Sophie 2020	25	22	3	13	10	3
Li, Chenze 2020	293	161	132	183	121	62
Li, Qiang 2020	24	19	5	7	5	2
Luo, Miao 2020	817	388	429	201	133	68
Xu, Bo 2020	117	59	58	70	44	26
Zeng, Hao-Long 2020	567	269	298	75	49	26
Zou, Li 2020	107	57	50	14	9	5
**Total****	**2662**	**1350**	**1268**	**758**	**463**	**240**

* Total number of patients based on sex (male/female) were not reported.
** Total number of patients based on sex is reported as an approximate number, this is because the male/female size from one study was not reported.

Table S8.4. Mean age of COVID-19 patients based on mortality.

Mean age of COVID-19 patients based on mortality				
Study	Survivors		Non-Survivors	
	Mean age	SD	Mean age	SD
Cantenys-Molina, S 2021	60.33	17.83	78.33	9.01
Flament, Héloïse 2021*	–	–	–	–
Gadotti, Ana Carolina 2020	57	22.34	66.33	16.89
Huang, Hong 2020	36.91	10.18	38.58	11.39
Hue, Sophie 2020	57.04	12.43	68.15	10.37
Li, Chenze 2020	68	13.41	70.66	12.7
Li, Qiang 2020	64	12	70	21
Luo, Miao 2020	56.33	14.85	69.66	11.94
Xu, Bo 2020	55	17.26	69.65	11.91
Zeng, Hao-Long 2020	60.25	14.57	69.66	11.33
Zou, Li 2020	62.33	14.15	66.33	10.52
**Total****	**59.36**	**16.11**	**70.68**	**12.82**

* Did not report mean age or SD values.
** Mean age indicates an approximate number; this is because the mean age from one study was not calculated due to missing mean age or SD values. Mean age and SD are calculated using the Cochrane's formula for combining subgroups.

PART IX

META-ANALYSIS SUMMARY

CD4 T-cells severity studies meta-analysis (summary)

Table S9.1. Meta-analysis summary for CD4 T-cells in COVID-19 severity studies.

```
Meta-analysis summary                          Number of studies =     26
Random-effects model                           Heterogeneity:
Method: DerSimonian-Laird                                   tau2 =  0.1175
                                                          I2 (%) =   73.20
                                                              H2 =    3.73
```

Study	Hedge's g	[95% conf.	interval]	% weight
Yang, Ai-Ping 2020	-1.388	-1.890	-0.886	3.79
Li, Xiaolei 2020	-1.213	-1.537	-0.888	4.79
Liu, Fangfang 2020	-1.186	-1.728	-0.644	3.58
Zou, Li 2020	-1.037	-1.418	-0.656	4.47
He, Susu 2020	-0.952	-1.455	-0.449	3.79
Liu, Jing 2020	-0.910	-1.588	-0.231	2.93
Li, Mingyue 2020	-0.881	-1.590	-0.172	2.80
Chen, Jiaxin 2020	-0.881	-1.779	0.018	2.12
Shi, Hongbo 2020	-0.821	-1.387	-0.255	3.46
Huang, Wei 2021	-0.796	-1.072	-0.521	5.06
Zhang, Bo 2021	-0.700	-0.854	-0.547	5.62
Tan, Mingkai 2020	-0.533	-1.062	-0.004	3.65
Yang, Fan 2020	-0.501	-1.065	0.063	3.47
Xu, Bo 2020	-0.487	-0.822	-0.152	4.74
Diao, Bo 2020	-0.455	-0.784	-0.125	4.77
Mann, Elizabeth 2020	-0.400	-1.124	0.324	2.73
Liao, Baolin 2021	-0.361	-0.741	0.018	4.48
Guan, Jingjing 2020	-0.340	-0.878	0.198	3.60
Schrijver, Benjamin 2020	-0.276	-0.948	0.397	2.95
Rendeiro, André 2020	-0.272	-0.923	0.378	3.05
Carissimo, Guillaume 2020	-0.226	-0.680	0.227	4.06
Laing, Adam 2020	-0.143	-0.526	0.240	4.46
Feng, Xiaobo 2020	-0.139	-0.618	0.341	3.92
Jin, Xiao-Hong 2020	-0.111	-0.473	0.252	4.58
Flament, Héloïse 2021	0.246	-0.100	0.591	4.67
Wang, Zhongliang 2020	0.338	-0.460	1.136	2.45
theta	-0.557	-0.721	-0.394	

```
Sorted by: _meta_es
Test of theta = 0: z = -6.69                          Prob > |z| = 0.0000
Test of homogeneity: Q = chi2(25) = 93.29               Prob > Q = 0.0000
```

CD8 T-cells severity studies meta-analysis (summary)

Table S9.2. Meta-analysis summary for CD8 T-cells in COVID-19 severity studies.

```
Meta-analysis summary                          Number of studies =     26
Random-effects model                           Heterogeneity:
Method: DerSimonian-Laird                                 tau2 =  0.0769
                                                        I2 (%) =   64.19
                                                            H2 =    2.79
```

Study	Hedge's g	[95% conf.	interval]	% weight
Liu, Fangfang 2020	-1.489	-2.053	-0.925	3.31
Diao, Bo 2020	-1.132	-1.474	-0.791	4.94
Shi, Hongbo 2020	-1.015	-1.592	-0.438	3.24
Li, Xiaolei 2020	-1.006	-1.324	-0.688	5.13
Yang, Ai-Ping 2020	-0.923	-1.402	-0.444	3.87
Chen, Jiaxin 2020	-0.796	-1.693	0.101	1.85
Carissimo, Guillaume 2020	-0.741	-1.208	-0.275	3.96
He, Susu 2020	-0.678	-1.168	-0.187	3.79
Yang, Fan 2020	-0.649	-1.219	-0.079	3.28
Huang, Wei 2021	-0.628	-0.899	-0.356	5.51
Li, Mingyue 2020	-0.594	-1.285	0.097	2.63
Wang, Zhongliang 2020	-0.585	-1.390	0.219	2.16
Feng, Xiaobo 2020	-0.526	-1.010	-0.041	3.84
Zou, Li 2020	-0.497	-0.860	-0.133	4.76
Zhang, Bo 2021	-0.451	-0.602	-0.300	6.39
Xu, Bo 2020	-0.434	-0.788	-0.080	4.83
Guan, Jingjing 2020	-0.433	-0.974	0.107	3.46
Liao, Baolin 2021	-0.398	-0.778	-0.018	4.62
Liu, Jing 2020	-0.350	-1.059	0.358	2.55
Jin, Xiao-Hong 2020	-0.223	-0.586	0.140	4.76
Flament, Héloïse 2021	-0.148	-0.494	0.197	4.91
Rendeiro, André 2020	-0.110	-0.758	0.539	2.84
Laing, Adam 2020	-0.098	-0.481	0.284	4.60
Tan, Mingkai 2020	-0.019	-0.539	0.500	3.60
Schrijver, Benjamin 2020	-0.018	-0.688	0.652	2.73
Mann, Elizabeth 2020	0.555	-0.174	1.284	2.46
theta	-0.528	-0.671	-0.386	

```
Sorted by: _meta_es
Test of theta = 0: z = -7.26                            Prob > |z| = 0.0000
Test of homogeneity: Q = chi2(25) = 69.80                 Prob > Q = 0.0000
```

IL-10 severity studies meta-analysis (summary)

Table S9.3. Meta-analysis summary for IL-10 in COVID-19 severity studies.

```
Meta-analysis summary                                  Number of studies =     38
Random-effects model                                   Heterogeneity:
Method: DerSimonian-Laird                                          tau2 =  0.0313
                                                                 I2 (%) =   37.43
                                                                     H2 =    1.60
```

Study	Hedge's g	[95% conf.	interval]	% weight
Liu, Jian 2020	0.192	-0.617	1.001	1.21
Shi, Hongbo 2020	0.205	-0.341	0.751	2.24
Laing, Adam 2020	0.229	-0.116	0.575	3.90
Chi, Ying 2020	0.297	-0.211	0.806	2.47
Jin, Xiao-Hong 2020	0.316	-0.049	0.680	3.70
Flament, Héloïse 2021	0.324	-0.243	0.891	2.12
McElvaney, Oliver 2020	0.361	-0.185	0.907	2.24
Yang, Ai-Ping 2020	0.375	-0.089	0.839	2.79
Liao, Baolin 2021	0.393	-0.093	0.879	2.62
Liu, Yangli 2021	0.403	-0.265	1.070	1.65
Liu, Lei 2020	0.414	-0.138	0.967	2.20
Zeng, Zhilin 2020	0.449	0.206	0.693	5.20
Deng, Fuxue 2020	0.470	0.090	0.849	3.54
Li, Mingyue 2020	0.471	-0.214	1.156	1.59
He, Susu 2020	0.491	0.071	0.912	3.15
Liu, Xue-Qing 2021	0.508	0.114	0.902	3.39
Han, Huan 2020	0.530	0.132	0.928	3.36
Azmy, Veronica 2021	0.544	0.178	0.911	3.67
Abers, Michael 2021	0.609	0.202	1.015	3.27
Zhao, Yan 2020	0.610	0.071	1.148	2.28
Keddie, Stephen 2020	0.623	0.152	1.094	2.73
Xu, Bo 2020	0.687	0.390	0.984	4.49
Tan, Mingkai 2020	0.751	0.213	1.289	2.28
Diao, Bo 2020	0.776	0.443	1.110	4.04
Zou, Li 2020	0.842	0.469	1.215	3.60
Huang, Wei 2021	0.903	0.087	1.719	1.19
Yi, Ping 2020	0.908	0.499	1.317	3.25
Kwon, Ji-Soo 2020	0.928	0.111	1.745	1.19
Li, Chenze 2020	0.935	0.708	1.163	5.43
Feng, Xiaobo 2020	0.989	0.493	1.486	2.55
Guan, Jingjing 2020	1.104	0.534	1.673	2.10
Wang, Zhongliang 2020	1.107	0.279	1.936	1.16
He, Bing 2020	1.144	0.560	1.729	2.03
Liu, Jing 2020	1.155	0.459	1.851	1.55
Mann, Elizabeth 2020	1.179	0.281	2.078	1.01
Schrijver, Benjamin 2020	1.197	0.471	1.922	1.45
Zhang, Jun 2020	1.263	0.735	1.791	2.34
Henry, Brandon Michael 2021	1.573	0.682	2.463	1.02
theta	0.644	0.547	0.741	

```
Sorted by: _meta_es
Test of theta = 0: z = 13.05                                Prob > |z| = 0.0000
Test of homogeneity: Q = chi2(37) = 59.13                     Prob > Q = 0.0119
```

CD4 T-cells mortality studies meta-analysis (summary)

Table S9.4. Meta-analysis summary for CD4 T-cells in COVID-19 mortality studies.

```
Meta-analysis summary                          Number of studies =      6
Random-effects model                           Heterogeneity:
Method: DerSimonian-Laird                                  tau2 =  0.0277
                                                         I2 (%) =   51.31
                                                             H2 =    2.05
```

Study	Hedge's g	[95% conf.	interval]	% weight
Zou, Li 2020	-0.990	-1.558	-0.423	9.73
Cantenys-Molina, S 2021	-0.964	-1.172	-0.756	27.85
Hue, Sophie 2020	-0.772	-1.451	-0.093	7.35
Luo, Miao 2020	-0.692	-0.849	-0.535	31.79
Xu, Bo 2020	-0.492	-0.847	-0.137	17.93
Li, Qiang 2020	-0.032	-0.852	0.788	5.35
theta	-0.731	-0.936	-0.527	

```
Sorted by: _meta_es
Test of theta = 0: z = -7.02                        Prob > |z| = 0.0000
Test of homogeneity: Q = chi2(5) = 10.27              Prob > Q = 0.0679
```

CD8 T-cells mortality studies meta-analysis (summary)

Table S9.5. Meta-analysis summary for CD8 T-cells in COVID-19 mortality studies.

```
Meta-analysis summary                          Number of studies =      6
Random-effects model                           Heterogeneity:
Method: DerSimonian–Laird                                   tau2 =  0.1499
                                                          I2 (%) =   64.08
                                                              H2 =    2.78
```

Study	Hedge's g	[95% conf.	interval]	% weight
Xu, Bo 2020	-0.890	-1.313	-0.467	20.99
Cantenys-Molina, S 2021	-0.796	-1.693	0.101	11.47
Luo, Miao 2020	-0.734	-1.141	-0.327	21.36
Zou, Li 2020	-0.471	-1.028	0.086	17.88
Li, Qiang 2020	0.173	-0.648	0.994	12.67
Hue, Sophie 2020	0.362	-0.300	1.023	15.63
theta	-0.441	-0.839	-0.043	

```
Sorted by: _meta_es
Test of theta = 0: z = -2.17                         Prob > |z| = 0.0299
Test of homogeneity: Q = chi2(5) = 13.92               Prob > Q = 0.0161
```

IL-10 mortality studies meta-analysis (summary)

Table S9.6. Meta-analysis summary for IL-10 in COVID-19 mortality studies.

```
Meta-analysis summary                          Number of studies =     10
Random-effects model                           Heterogeneity:
Method: DerSimonian-Laird                                  tau2 =  0.2054
                                                        I2 (%) =   88.18
                                                            H2 =    8.46
```

Study	Hedge's g	[95% conf.	interval]	% weight
Gadotti, Ana 2020	-0.011	-0.564	0.542	9.35
Hue, Sophie 2020	0.288	-0.372	0.947	8.37
Flament, Héloïse 2021	0.325	-0.071	0.721	10.83
Zou, Li 2020	0.341	0.117	0.566	12.20
Li, Chenze 2020	0.642	0.356	0.929	11.76
Li, Qiang 2020	0.849	0.002	1.695	6.80
Zeng, Hao-Long 2020	1.033	0.786	1.280	12.05
Xu, Bo 2020	1.280	0.899	1.662	10.96
Luo, Miao 2020	1.285	1.121	1.449	12.55
Huang, Hong 2020	1.595	0.498	2.693	5.14
theta	0.741	0.421	1.061	

```
Sorted by: _meta_es
Test of theta = 0: z = 4.54                         Prob > |z| = 0.0000
Test of homogeneity: Q = chi2(9) = 76.11              Prob > Q = 0.0000
```

PART X

INVESTIGATING THE SOURCE OF HETEROGENEITY

SUBGROUP ANALYSIS AND

META-REGRESSION

Subgroup analysis under moderator (city) for CD4 T-cell in COVID-19 severity studies

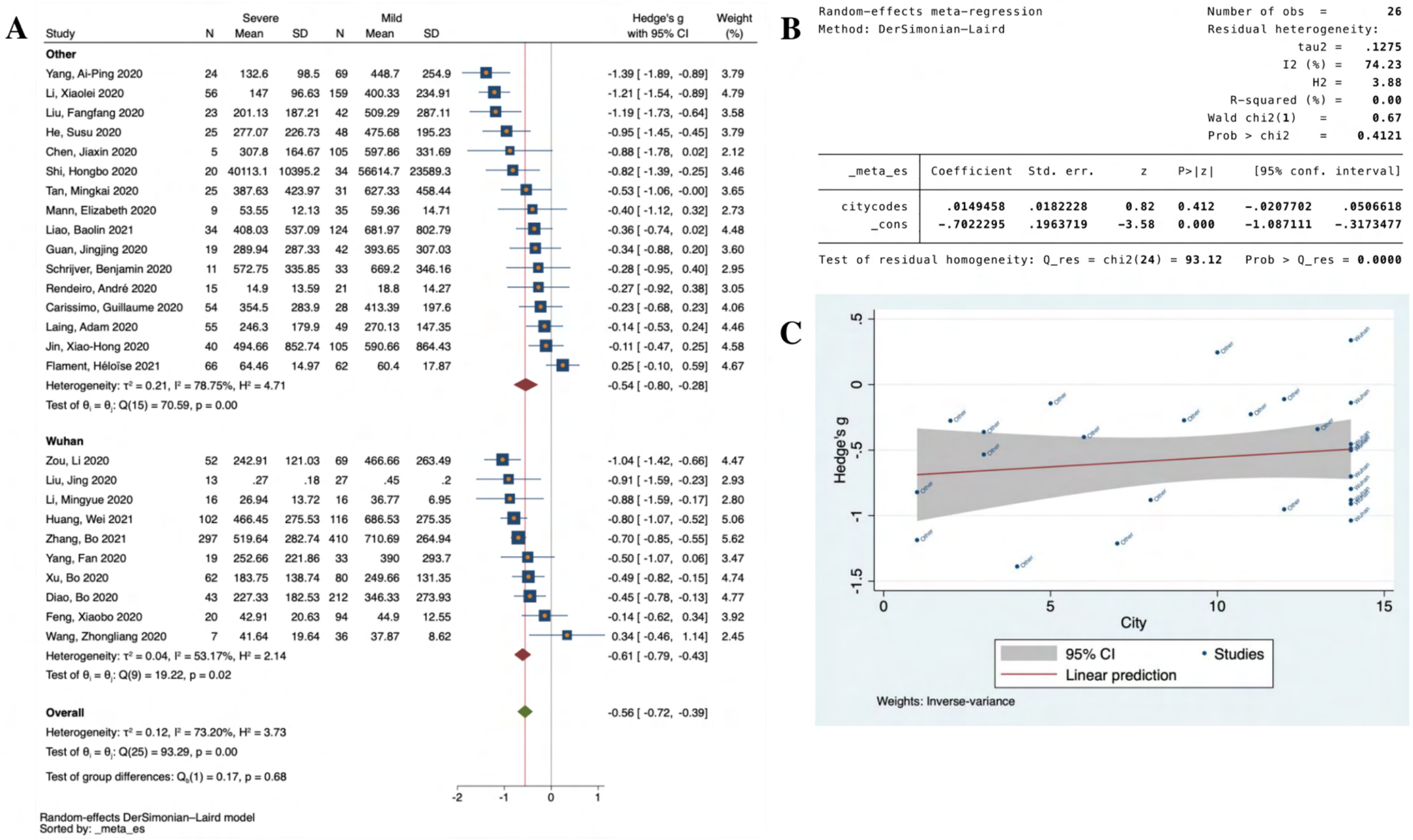

Figure S10.1. Subgroup analysis performed under the moderator (city) for CD4 T-cells in COVID-19 severity studies. (A) Subgroup forest plot. The no-effect line is represented at the value of zero. The diamond symbol represents estimated combined effect. (B) Subgroup meta-regression. (C) Subgroup meta-regression bubble plot. Studies are represented as (bubbles). The regression line (red). The horizontal axis represents cities.

Subgroup analysis under moderator (country) for CD4 T-cell in COVID-19 severity studies

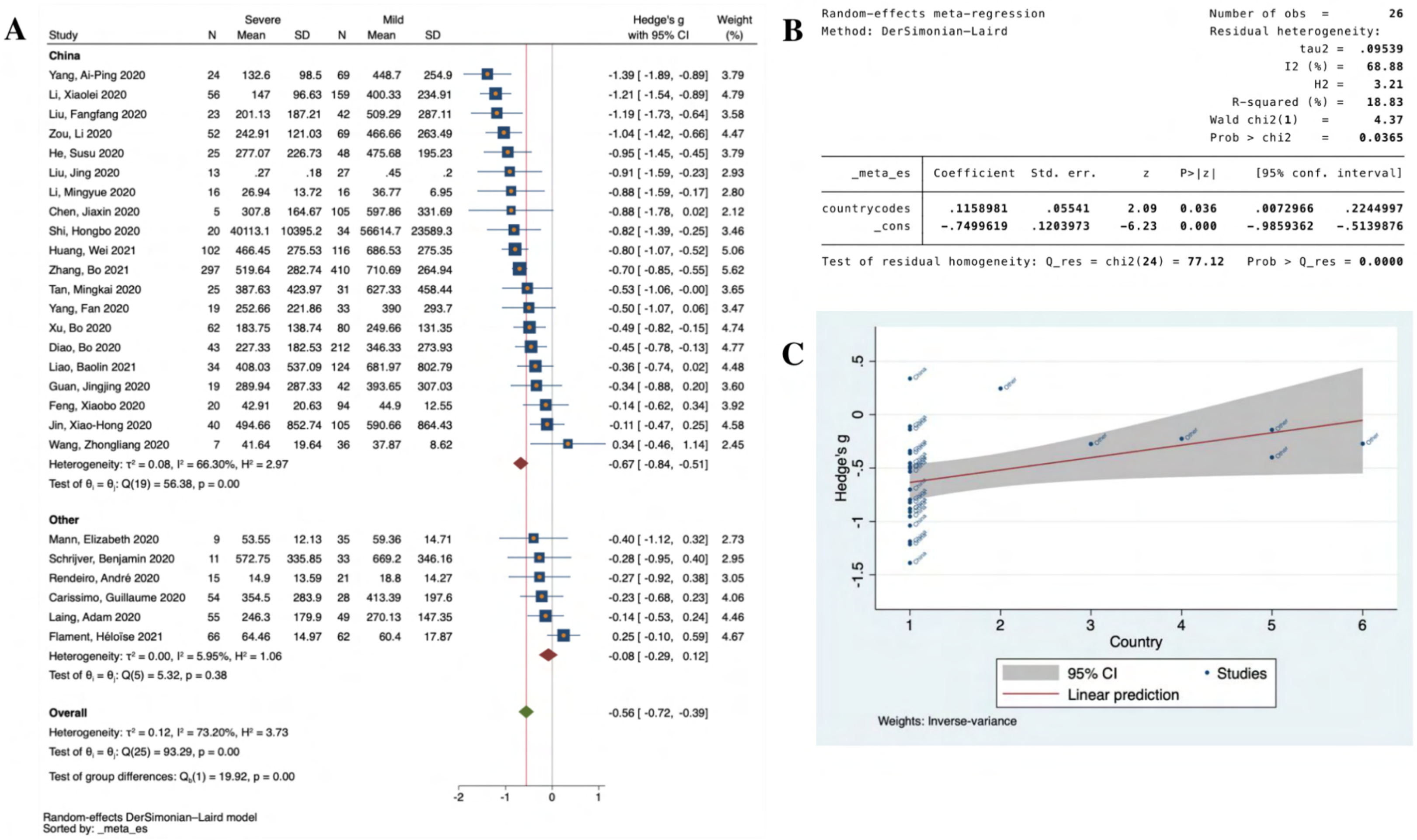

Figure S10.2. Subgroup analysis performed under the moderator (country) for CD4 T-cells in COVID-19 severity studies. (A) Subgroup forest plot. The no-effect line is represented at the value of zero. The diamond symbol represents estimated combined effect. (B) Subgroup meta-regression. (C) Subgroup meta-regression bubble plot. Studies are represented as (bubbles). The regression line (red). The horizontal axis represents countries.

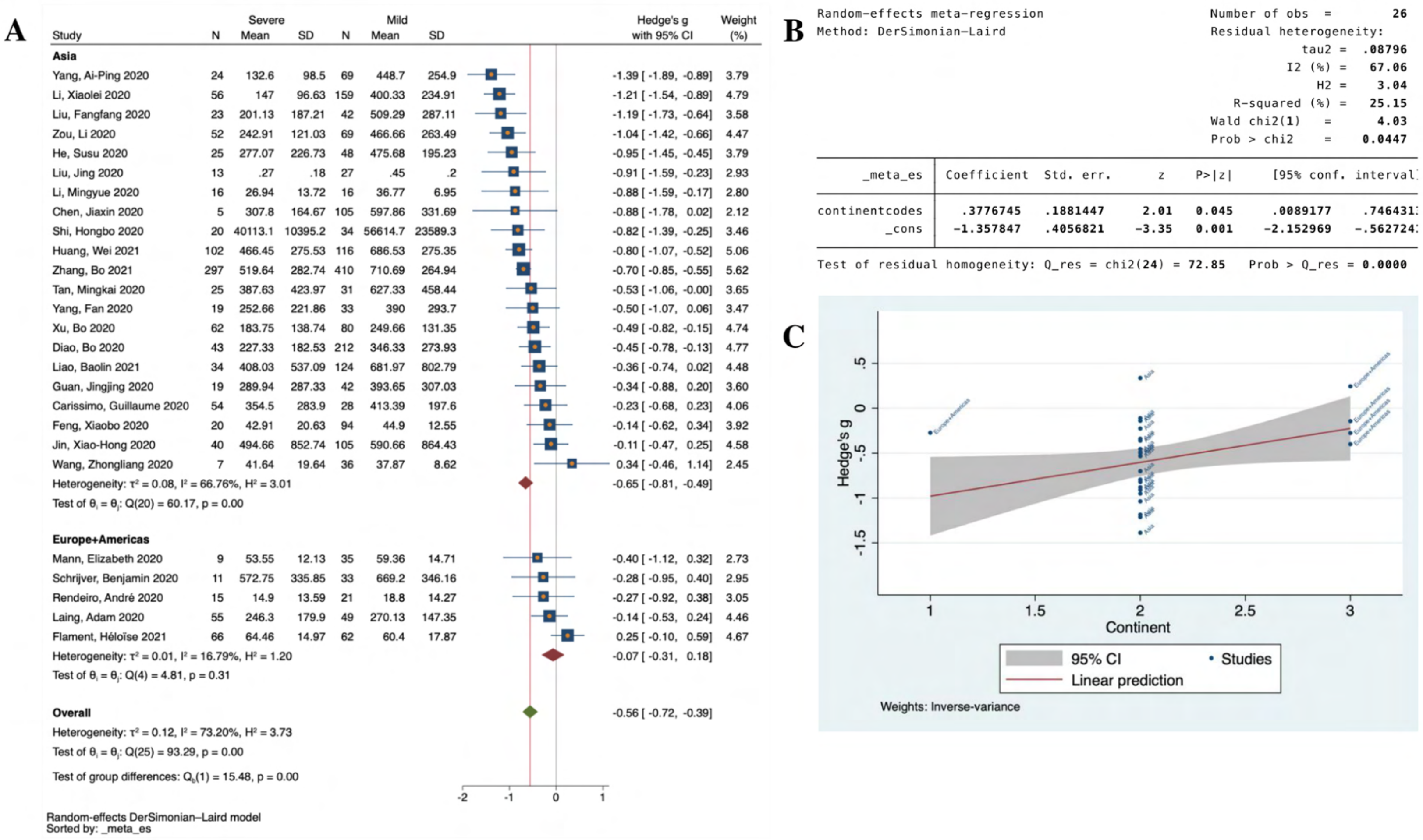

Figure S10.3. Subgroup analysis performed under the moderator (continent) for CD4 T-cells in COVID-19 severity studies. (A) Subgroup forest plot. The no-effect line is represented at the value of zero. The diamond symbol represents estimated combined effect. (B) Subgroup meta-regression. (C) Subgroup meta-regression bubble plot. Studies are represented as (bubbles). The regression line (red). The horizontal axis represents continents.

Subgroup analysis under moderator (study design) for CD4 T-cell in COVID-19 severity studies

A

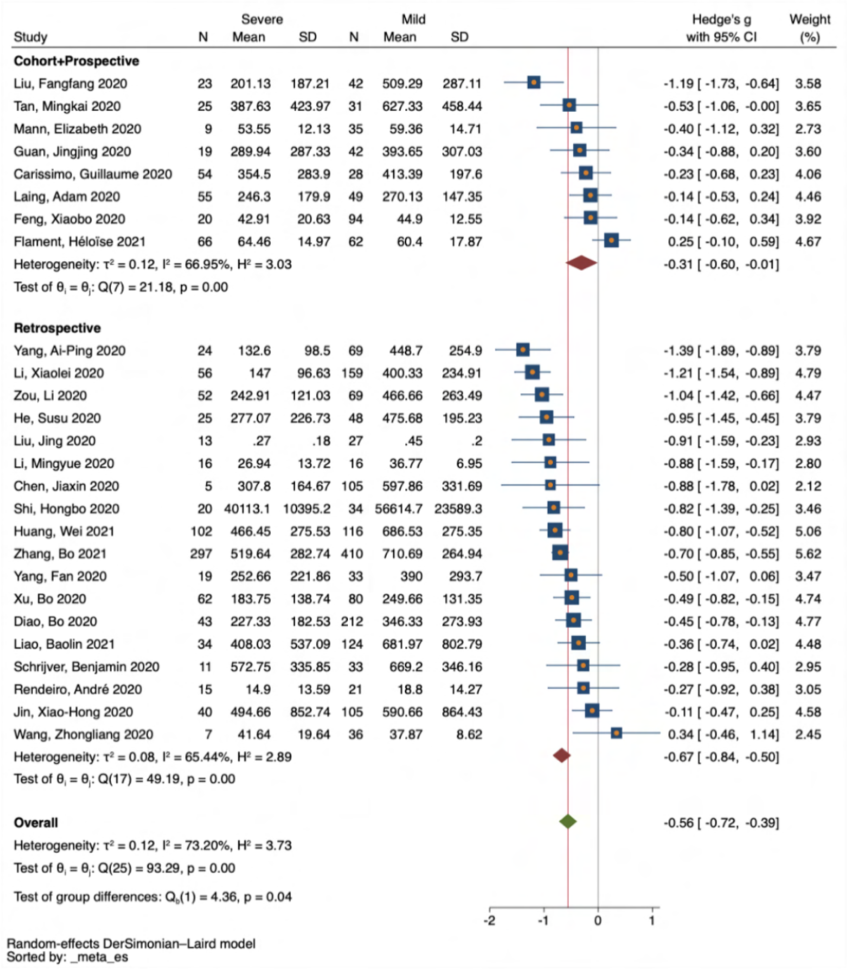

B

```
Random-effects meta-regression                  Number of obs  =         26
Method: DerSimonian-Laird                       Residual heterogeneity:
                                                             tau2 =  .08729
                                                           I2 (%) =   66.63
                                                               H2 =    3.00
                                                    R-squared (%) =   25.73
                                                Wald chi2(1)      =    4.41
                                                Prob > chi2       =  0.0358

      _meta_es   Coefficient  Std. err.      z    P>|z|     [95% conf. interval]

   designcodes    -.1800719   .0857927    -2.10   0.036    -.3482226   -.0119213
         _cons    -.1202733   .2217538    -0.54   0.588    -.5549027    .3143561

Test of residual homogeneity: Q_res = chi2(24) = 71.92    Prob > Q_res = 0.0000
```

C

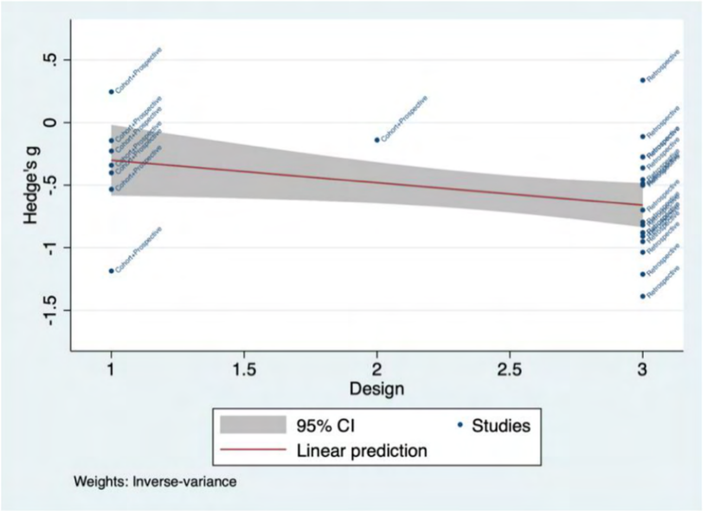

Figure S10.4. Subgroup analysis performed under the moderator (study design) for CD4 T-cells in COVID-19 severity studies. (A) Subgroup forest plot. The no-effect line is represented at the value of zero. The diamond symbol represents estimated combined effect. (B) Subgroup meta-regression. (C) Subgroup meta-regression bubble plot. Studies are represented as (bubbles). The regression line (red). The horizontal axis represents study design.

Subgroup analysis under moderator (classification protocol) for CD4 T-cell in COVID-19 severity studies

A

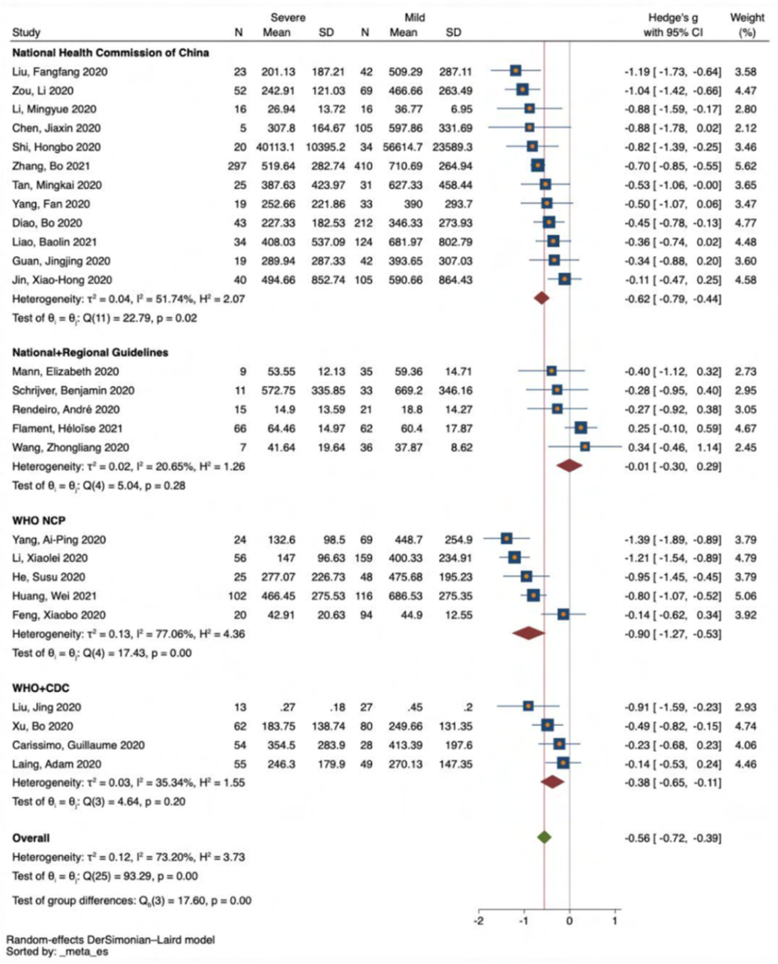

B

```
Random-effects meta-regression                  Number of obs  =        26
Method: DerSimonian–Laird                       Residual heterogeneity:
                                                          tau2 =    .09965
                                                        I2 (%) =     69.36
                                                            H2 =      3.26
                                                 R-squared (%) =     15.21
                                                 Wald chi2(1)  =      3.80
                                                 Prob > chi2   =    0.0512

     _meta_es   Coefficient  Std. err.      z    P>|z|     [95% conf. interval]

protocolcodes    -.0994524   .0510041   -1.95   0.051    -.1994187    .0005138
        _cons    -.1040061    .245656   -0.42   0.672     -.585483    .3774708

Test of residual homogeneity: Q_res = chi2(24) = 78.33   Prob > Q_res = 0.0000
```

C

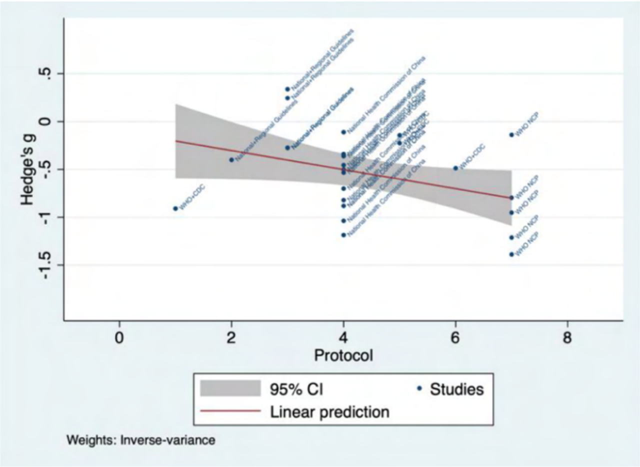

Figure S10.5. Subgroup analysis performed under the moderator (classification protocol) for CD4 T-cells in COVID-19 severity studies. (A) Subgroup forest plot. The no-effect line is represented at the value of zero. The diamond symbol represents estimated combined effect. (B) Subgroup meta-regression. (C) Subgroup meta-regression bubble plot. Studies are represented as (bubbles). The regression line (red). The horizontal axis represents classification protocols.

Subgroup analysis under moderator (sample acquisition time) for CD4 T-cell in COVID-19 severity studies

---

A

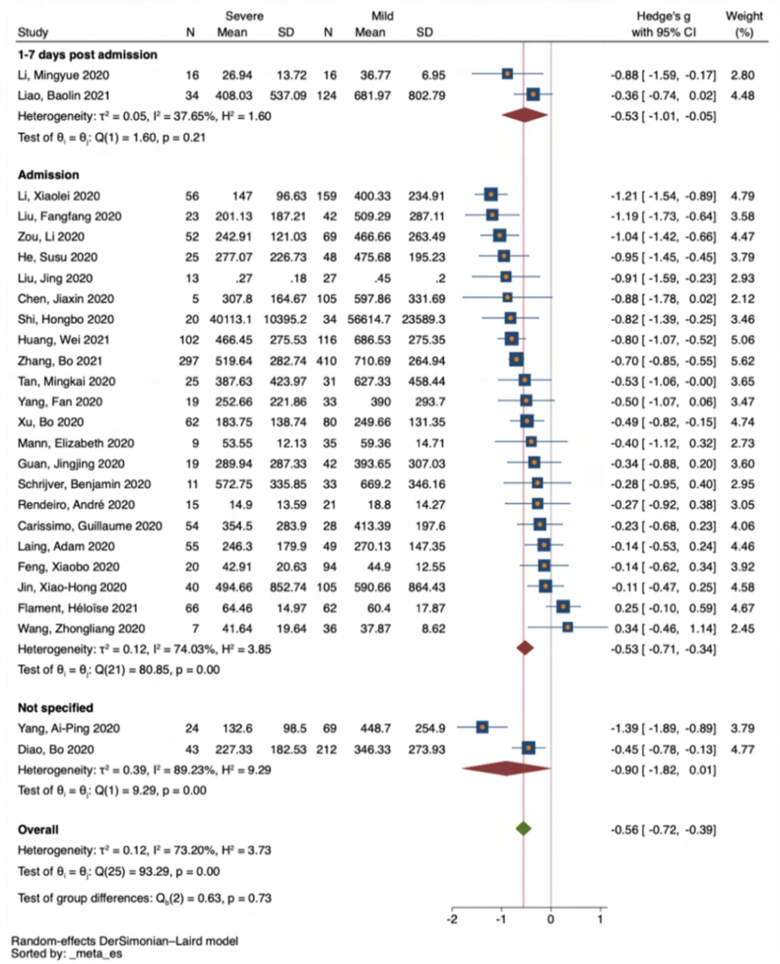

B

```
Random-effects meta-regression                  Number of obs  =        26
Method: DerSimonian-Laird                       Residual heterogeneity:
                                                          tau2 =     .1227
                                                        I2 (%) =     74.06
                                                            H2 =      3.86
                                                 R-squared (%) =      0.00
                                                   Wald chi2(1) =      0.17
                                                   Prob > chi2  =    0.6801

     _meta_es | Coefficient  Std. err.      z    P>|z|     [95% conf. interval]
  samplecodes |   -.0708004   .1717036   -0.41   0.680    -.4073333    .2657326
        _cons |   -.3460842   .5193556   -0.67   0.505    -1.364002    .6718341

Test of residual homogeneity: Q_res = chi2(24) = 92.53   Prob > Q_res = 0.0000
```

C

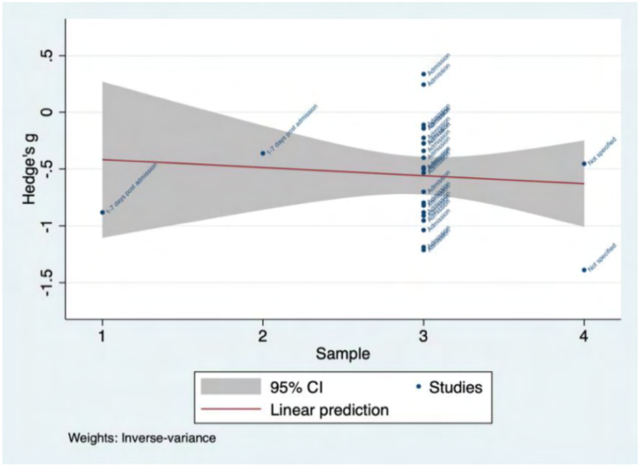

Figure S10.6. Subgroup analysis performed under the moderator (sample acquisition time) for CD4 T-cells in COVID-19 severity studies. (A) Subgroup forest plot. The no-effect line is represented at the value of zero. The diamond symbol represents estimated combined effect. (B) Subgroup meta-regression. (C) Subgroup meta-regression bubble plot. Studies are represented as (bubbles). The regression line (red). The horizontal axis represents sample acquisition time.

Subgroup analysis under moderator (total male number) for CD4 T-cell in COVID-19 severity studies

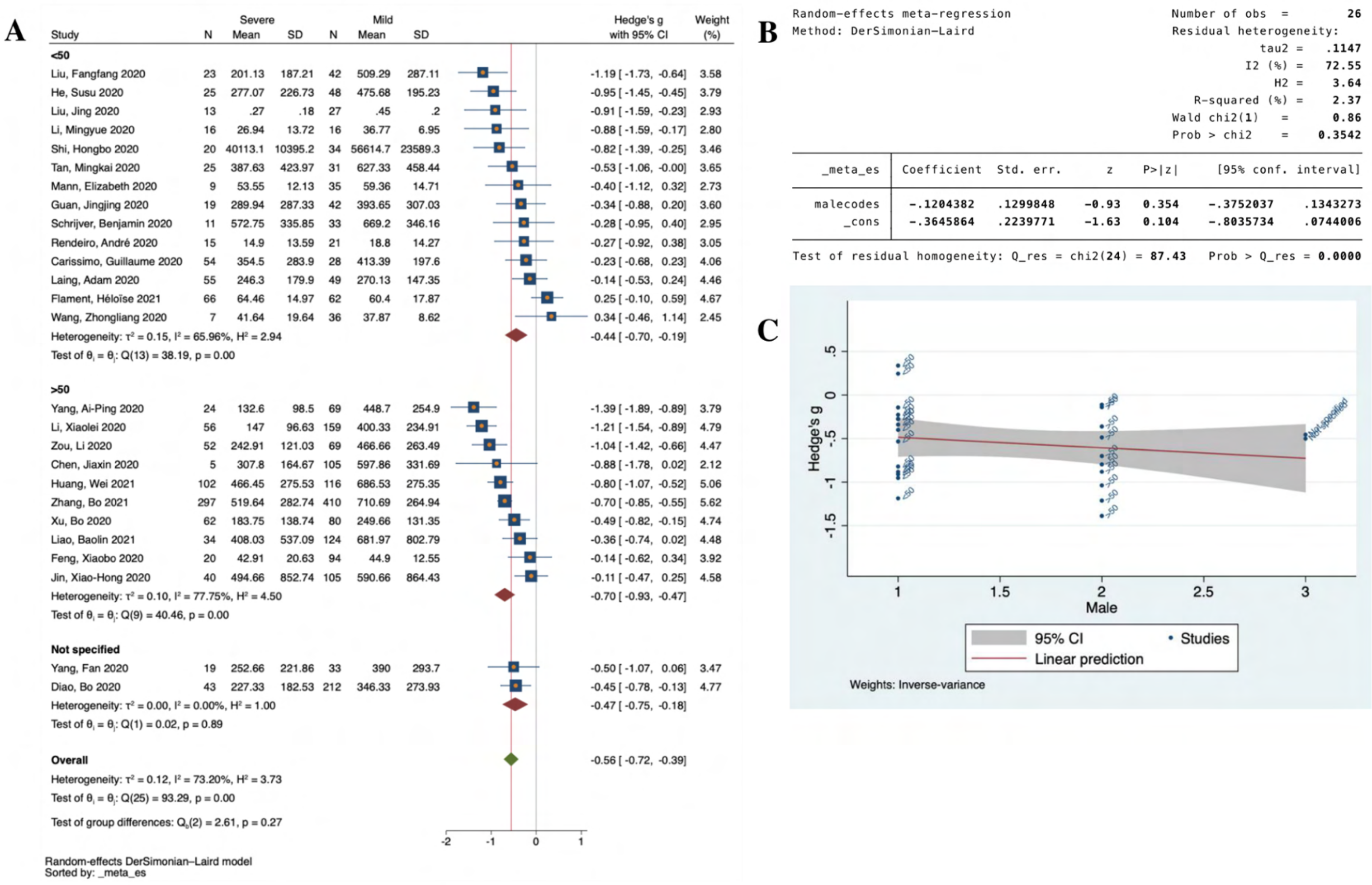

Figure S10.7. Subgroup analysis performed under the moderator (total male number) for CD4 T-cells in COVID-19 severity studies. (A) Subgroup forest plot. The no-effect line is represented at the value of zero. The diamond symbol represents estimated combined effect. (B) Subgroup meta-regression. (C) Subgroup meta-regression bubble plot. Studies are represented as (bubbles). The regression line (red). The horizontal axis represents total male number.

Subgroup analysis under moderator (total female number) for CD4 T-cell in COVID-19 severity studies

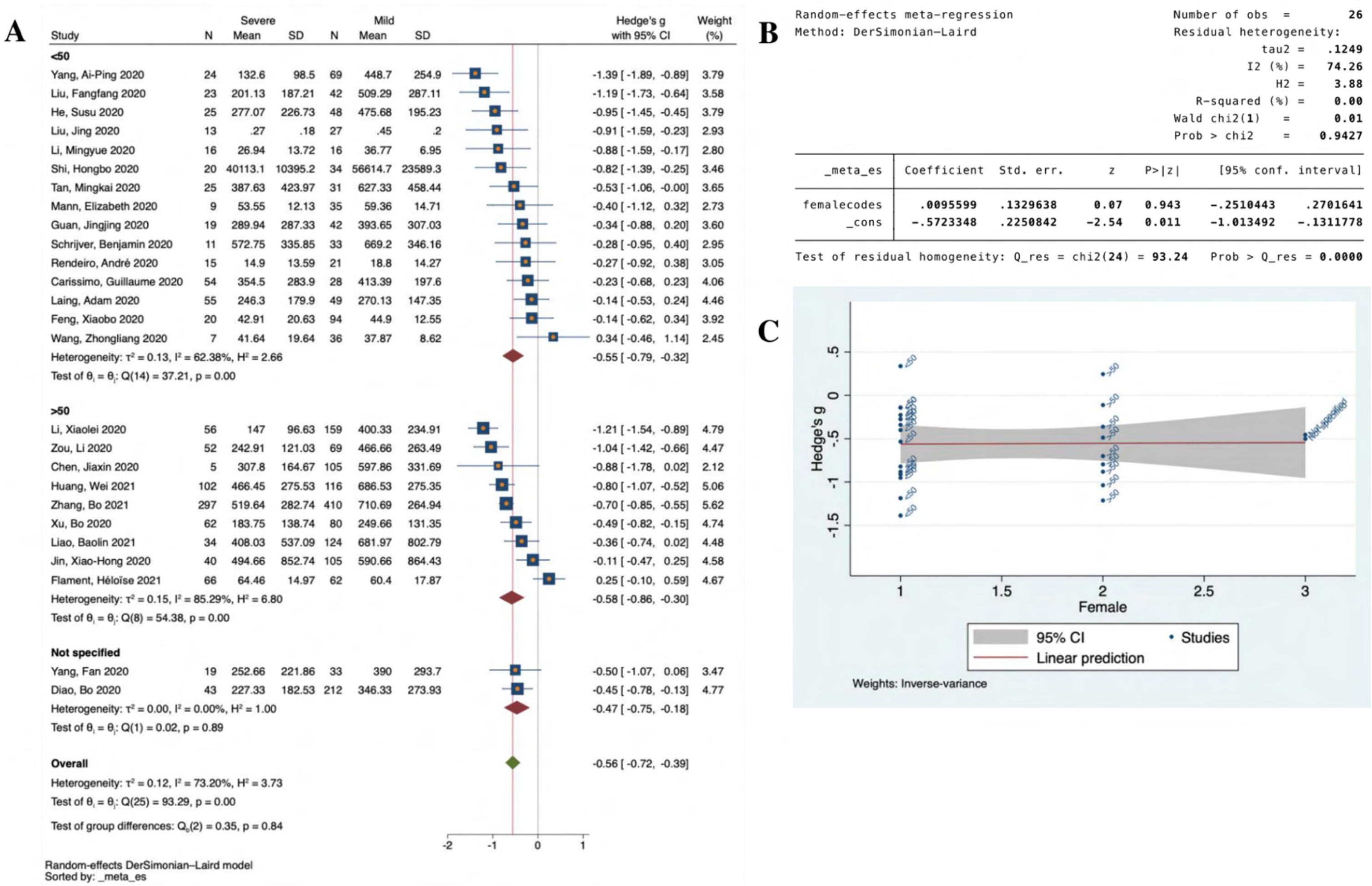

Figure S10.8. Subgroup analysis performed under the moderator (total female number) for CD4 T-cells in COVID-19 severity studies. (A) Subgroup forest plot. The no-effect line is represented at the value of zero. The diamond symbol represents estimated combined effect. (B) Subgroup meta-regression. (C) Subgroup meta-regression bubble plot. Studies are represented as (bubbles). The regression line (red). The horizontal axis represents total female number.

Subgroup analysis under moderator (mean age) for CD4 T-cell in COVID-19 severity studies

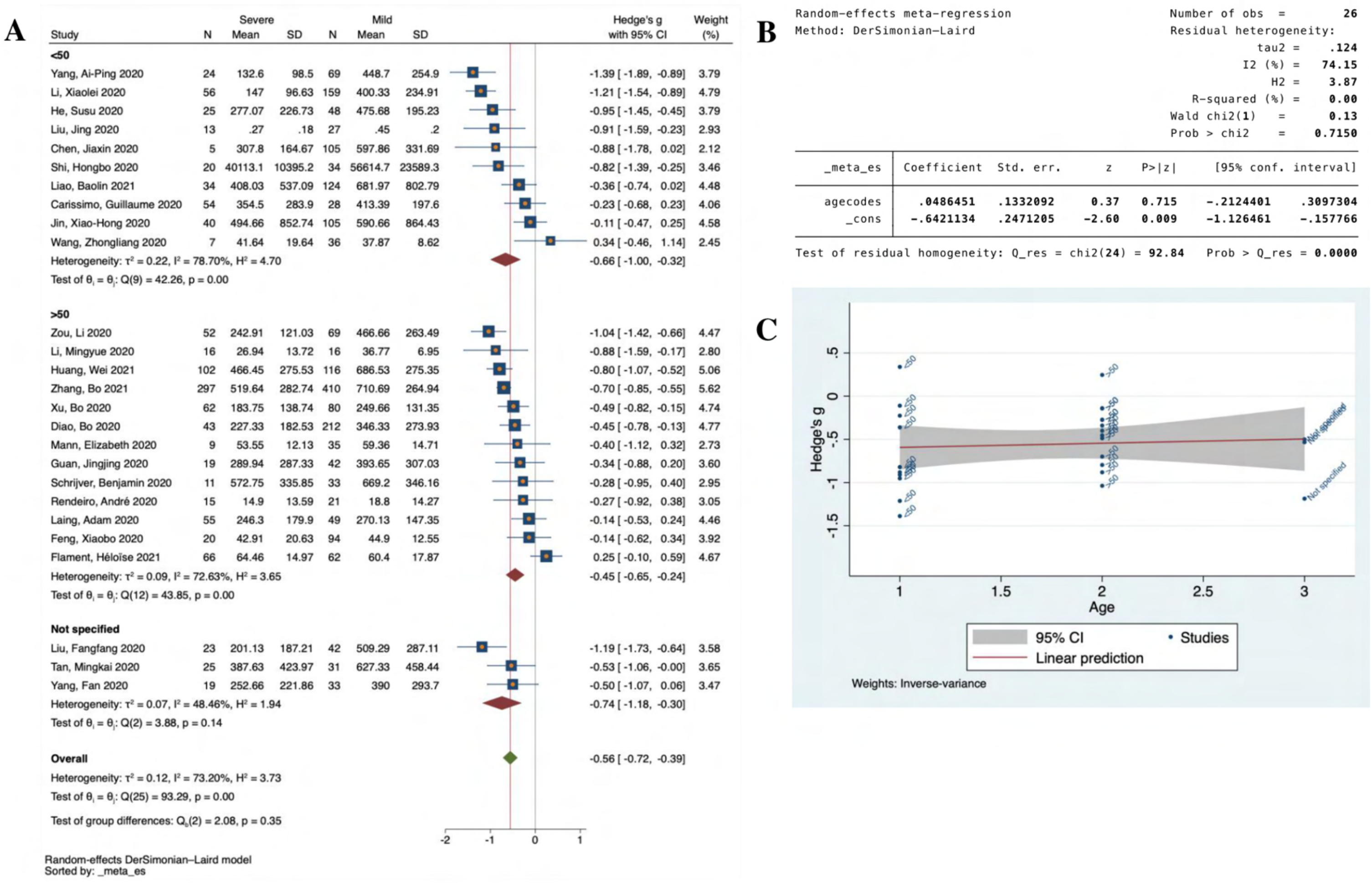

Figure S10.9. Subgroup analysis performed under the moderator (mean age) for CD4 T-cells in COVID-19 severity studies. (A) Subgroup forest plot. The no-effect line is represented at the value of zero. The diamond symbol represents estimated combined effect. (B) Subgroup meta-regression. (C) Subgroup meta-regression bubble plot. Studies are represented as (bubbles). The regression line (red). The horizontal axis represents mean age.

Subgroup analysis under moderator (test procedure) for CD4 T-cell in COVID-19 severity studies

A

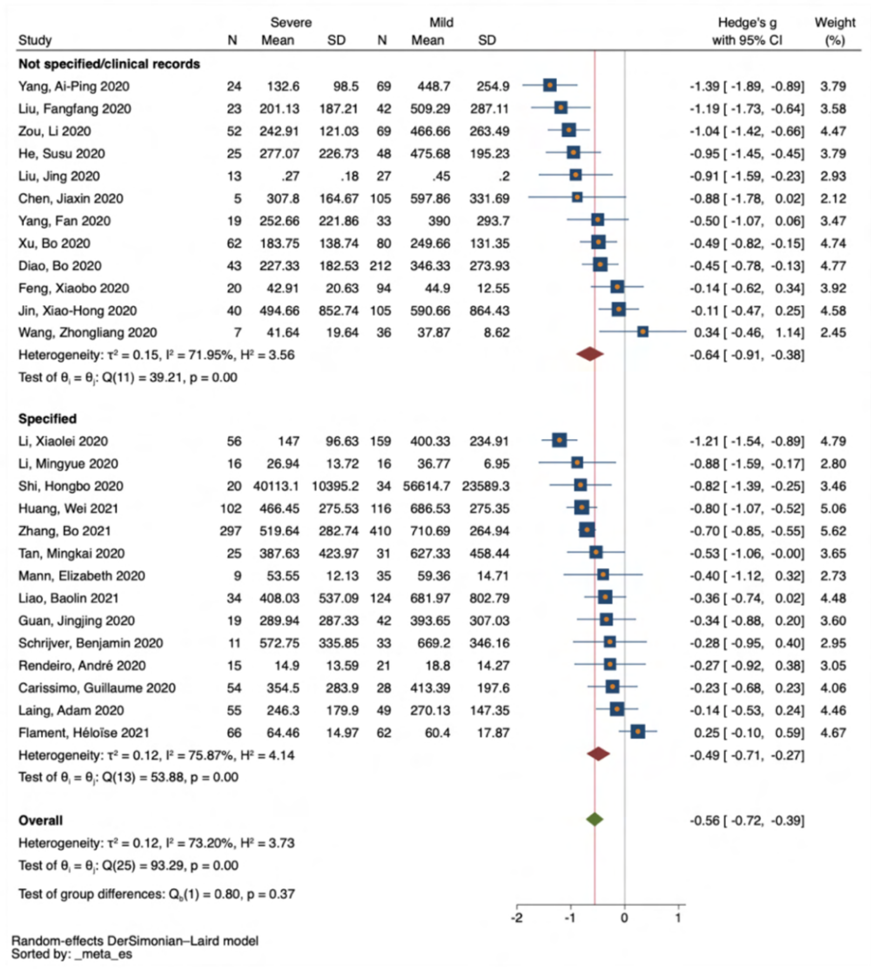

B

```
Random-effects meta-regression                  Number of obs  =         26
Method: DerSimonian–Laird                       Residual heterogeneity:
                                                            tau2 =    .1284
                                                          I2 (%) =    74.22
                                                              H2 =     3.88
                                                     R-squared (%) =     0.00
                                                Wald chi2(1)    =     0.83
                                                Prob > chi2     =   0.3626

    _meta_es | Coefficient  Std. err.      z    P>|z|     [95% conf. interval]
   testcodes |   .1573671   .1728394    0.91   0.363    -.1813918    .4961261
       _cons |  -.8017215    .281849   -2.84   0.004    -1.354135   -.2493076

Test of residual homogeneity: Q_res = chi2(24) = 93.09    Prob > Q_res = 0.0000
```

C

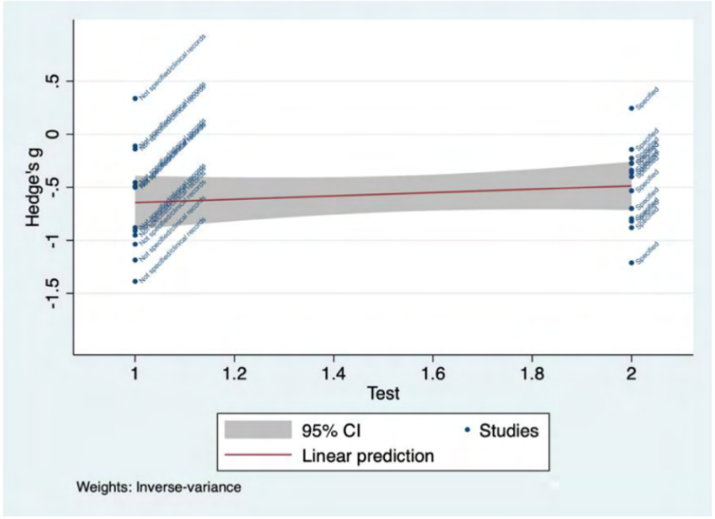

Figure S10.10. Subgroup analysis performed under the moderator (test procedure) for CD4 T-cells in COVID-19 severity studies. (A) Subgroup forest plot. The no-effect line is represented at the value of zero. The diamond symbol represents estimated combined effect. (B) Subgroup meta-regression. (C) Subgroup meta-regression bubble plot. Studies are represented as (bubbles). The regression line (red). The horizontal axis represents test procedure.

Subgroup analysis under moderator (city) for CD8 T-cell in COVID-19 severity studies

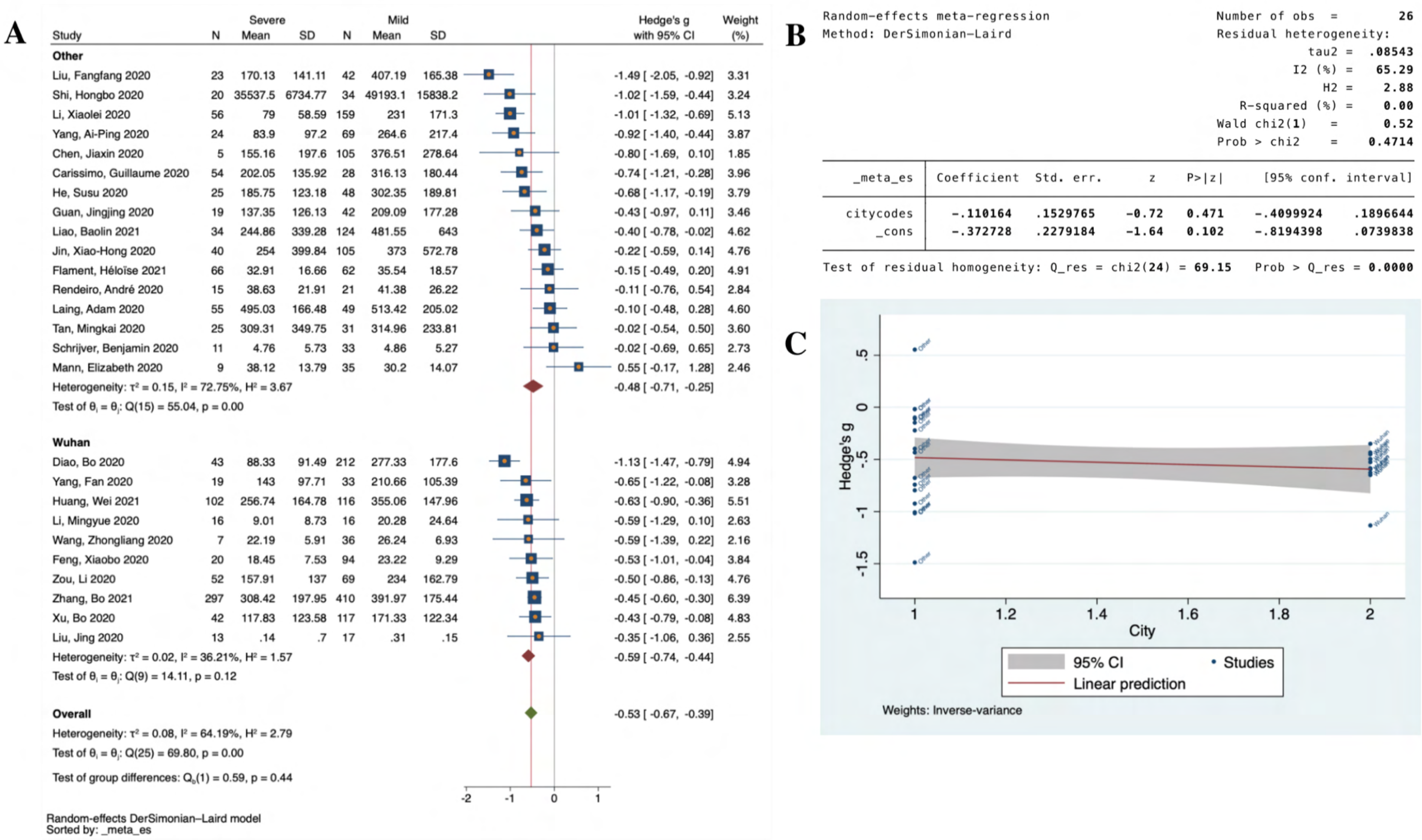

Figure S10.11. Subgroup analysis performed under the moderator (city) for CD8 T-cells in COVID-19 severity studies. (A) Subgroup forest plot. The no-effect line is represented at the value of zero. The diamond symbol represents estimated combined effect. (B) Subgroup meta-regression. (C) Subgroup meta-regression bubble plot. Studies are represented as (bubbles). The regression line (red). The horizontal axis represents cities.

Subgroup analysis under moderator (country) for CD8 T-cell in COVID-19 severity studies

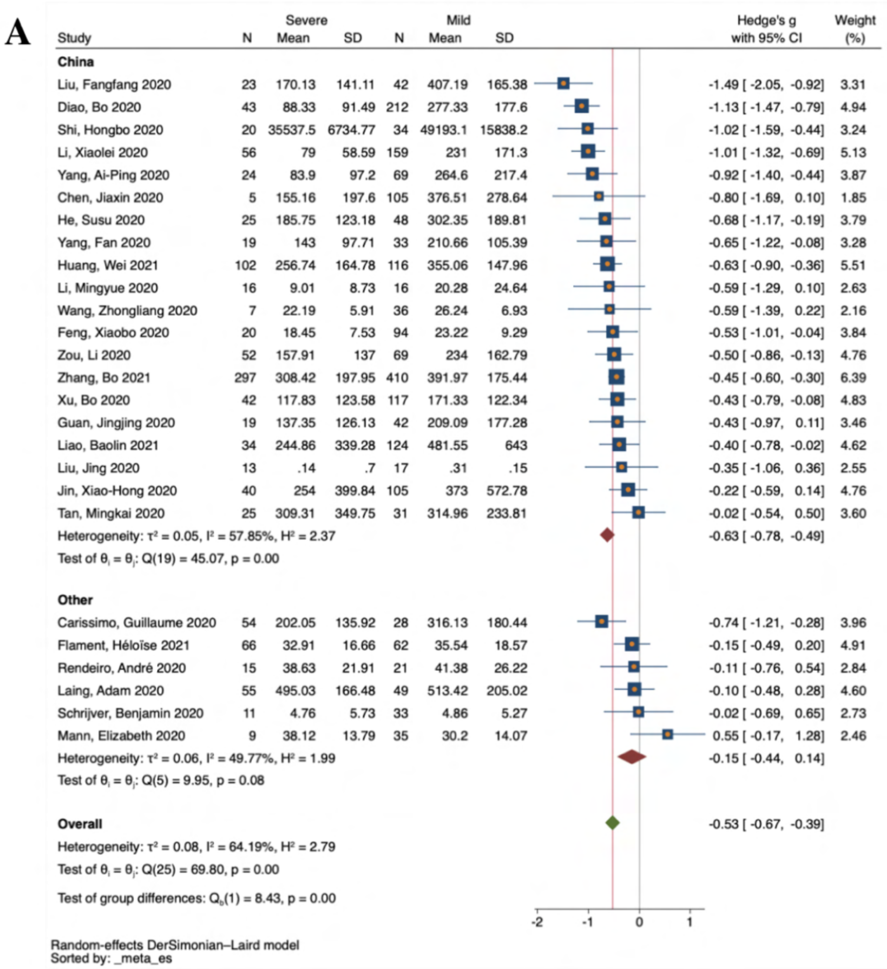

B

```
Random-effects meta-regression                 Number of obs  =         26
Method: DerSimonian-Laird                      Residual heterogeneity:
                                                          tau2 =     .0561
                                                        I2 (%) =     56.38
                                                            H2 =      2.29
                                                  R-squared (%) =     27.01
                                               Wald chi2(1)    =      8.72
                                               Prob > chi2     =    0.0031

    _meta_es | Coefficient  Std. err.      z    P>|z|     [95% conf. interval]
-------------+-----------------------------------------------------------------
countrycodes |   .4804058   .1626463    2.95   0.003      .161625    .7991866
       _cons |  -1.111856   .2079536   -5.35   0.000    -1.519438   -.7042747

Test of residual homogeneity: Q_res = chi2(24) = 55.03   Prob > Q_res = 0.0003
```

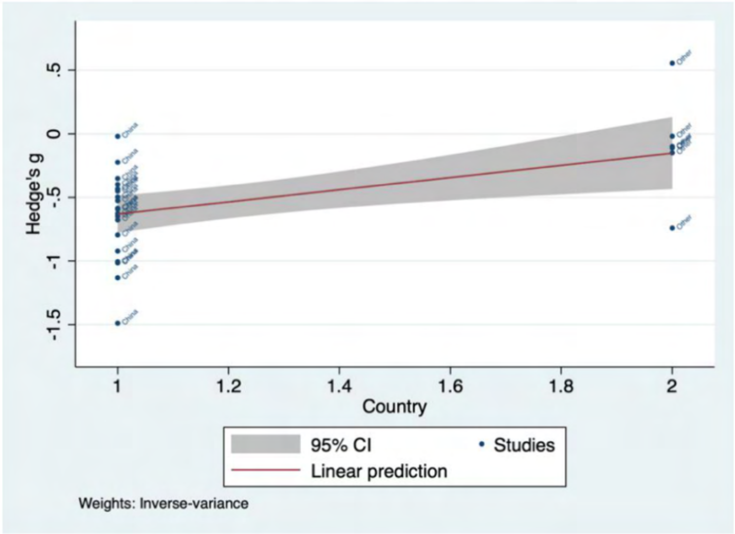

Figure S10.12. Subgroup analysis performed under the moderator (country) for CD8 T-cells in COVID-19 severity studies. (A) Subgroup forest plot. The no-effect line is represented at the value of zero. The diamond symbol represents estimated combined effect. (B) Subgroup meta-regression. (C) Subgroup meta-regression bubble plot. Studies are represented as (bubbles). The regression line (red). The horizontal axis represents countries.

Subgroup analysis under moderator (continent) for CD8 T-cell in COVID-19 severity studies

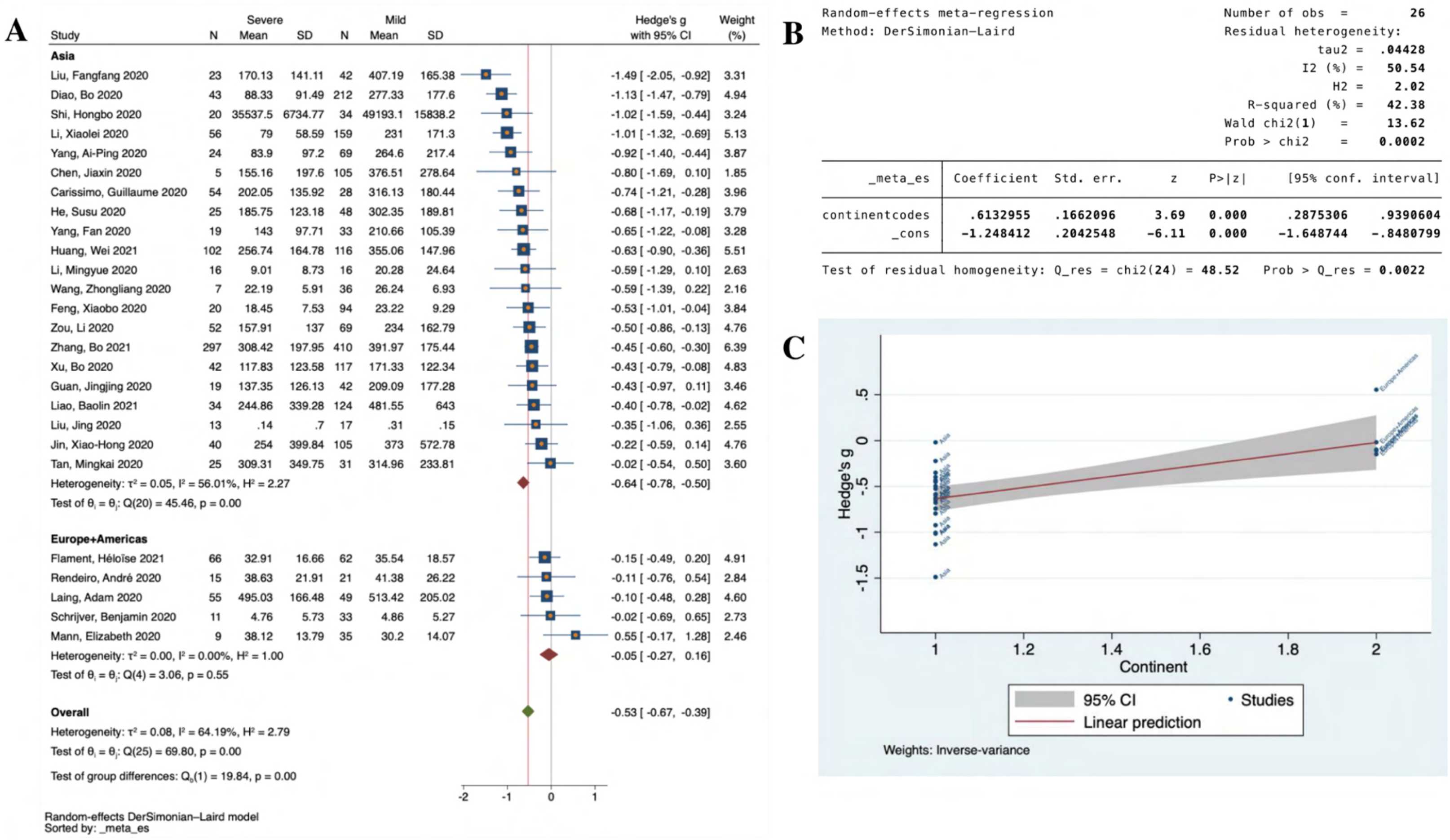

Figure S10.13. Subgroup analysis performed under the moderator (continent) for CD8 T-cells in COVID-19 severity studies. (A) Subgroup forest plot. The no-effect line is represented at the value of zero. The diamond symbol represents estimated combined effect. (B) Subgroup meta-regression. (C) Subgroup meta-regression bubble plot. Studies are represented as (bubbles). The regression line (red). The horizontal axis represents continents.

Subgroup analysis under moderator (study design) for CD8 T-cell in COVID-19 severity studies

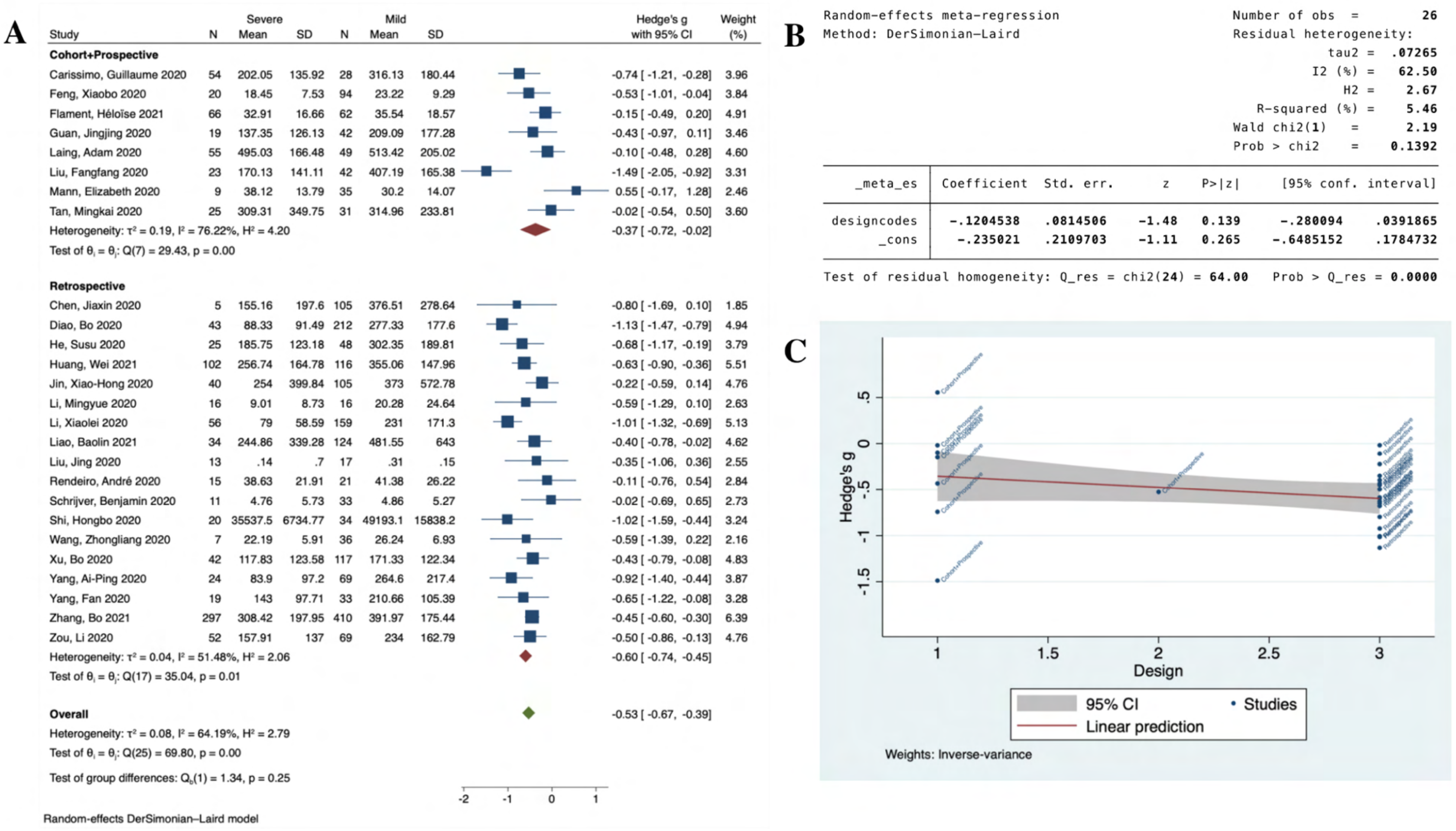

Figure S10.14. Subgroup analysis performed under the moderator (study design) for CD8 T-cells in COVID-19 severity studies. (A) Subgroup forest plot. The no-effect line is represented at the value of zero. The diamond symbol represents estimated combined effect. (B) Subgroup meta-regression. (C) Subgroup meta-regression bubble plot. Studies are represented as (bubbles). The regression line (red). The horizontal axis represents study design.

Subgroup analysis under moderator (classification protocol) for CD8 T-cell in COVID-19 severity studies

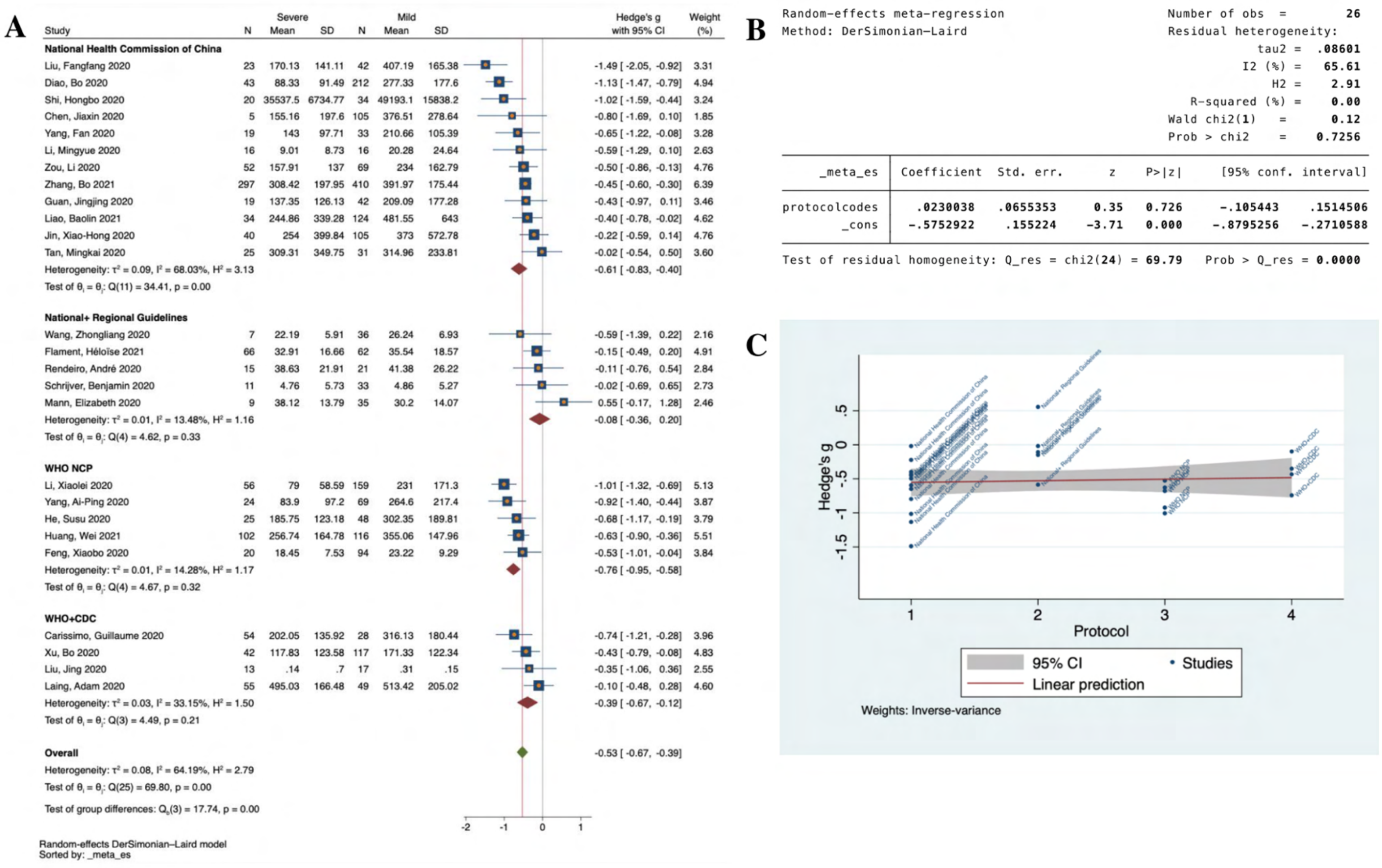

Figure S10.15. Subgroup analysis performed under the moderator (classification protocol) for CD8 T-cells in COVID-19 severity studies. (A) Subgroup forest plot. The no-effect line is represented at the value of zero. The diamond symbol represents estimated combined effect. (B) Subgroup meta-regression. (C) Subgroup meta-regression bubble plot. Studies are represented as (bubbles). The regression line (red). The horizontal axis represents classification protocols.

Subgroup analysis under moderator (sample acquisition time) for CD8 T-cell in COVID-19 severity studies

A

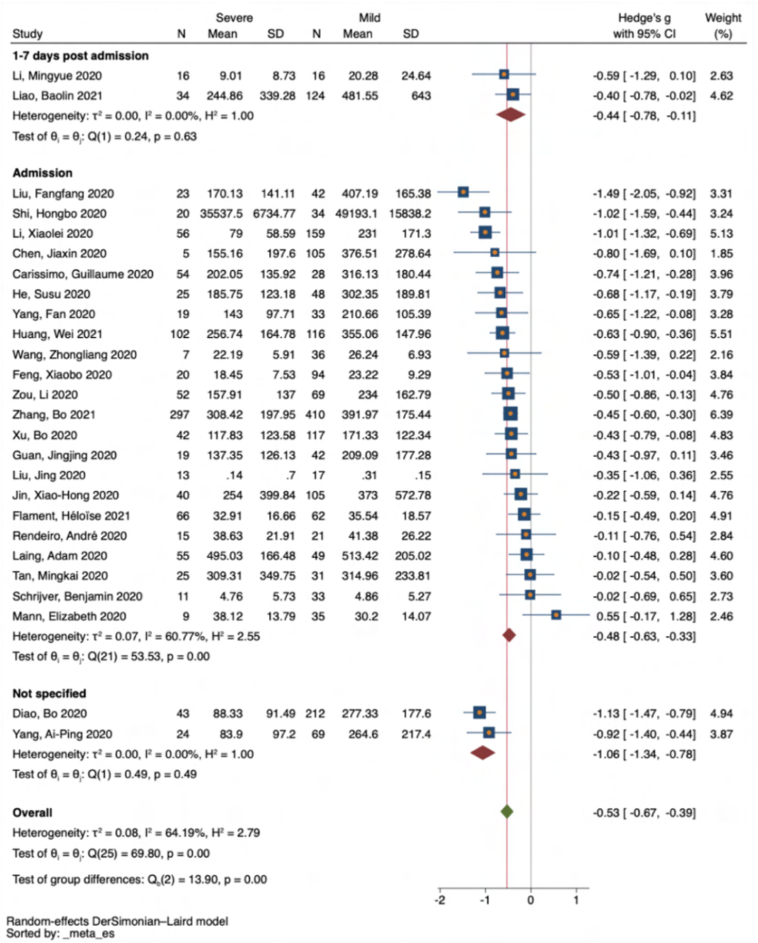

B

```
Random-effects meta-regression                  Number of obs    =        26
Method: DerSimonian-Laird                       Residual heterogeneity:
                                                           tau2 =    .06356
                                                         I2 (%) =     59.63
                                                             H2 =      2.48
                                                   R-squared (%) =     17.29
                                                Wald chi2(1)     =      3.29
                                                Prob > chi2      =    0.0696

    _meta_es   Coefficient  Std. err.      z    P>|z|     [95% conf. interval]

 samplecodes    -.3116321   .1717433   -1.81   0.070    -.6482427    .0249785
       _cons     .0992831   .3531353    0.28   0.779    -.5928494    .7914157

Test of residual homogeneity: Q_res = chi2(24) = 59.44   Prob > Q_res = 0.0001
```

C

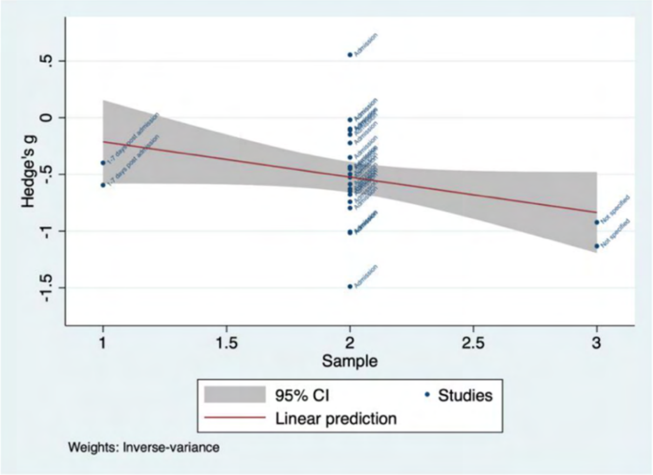

Figure S10.16. Subgroup analysis performed under the moderator (sample acquisition time) for CD8 T-cells in COVID-19 severity studies. (A) Subgroup forest plot. The no-effect line is represented at the value of zero. The diamond symbol represents estimated combined effect. (B) Subgroup meta-regression. (C) Subgroup meta-regression bubble plot. Studies are represented as (bubbles). The regression line (red). The horizontal axis represents sample acquisition time.

A

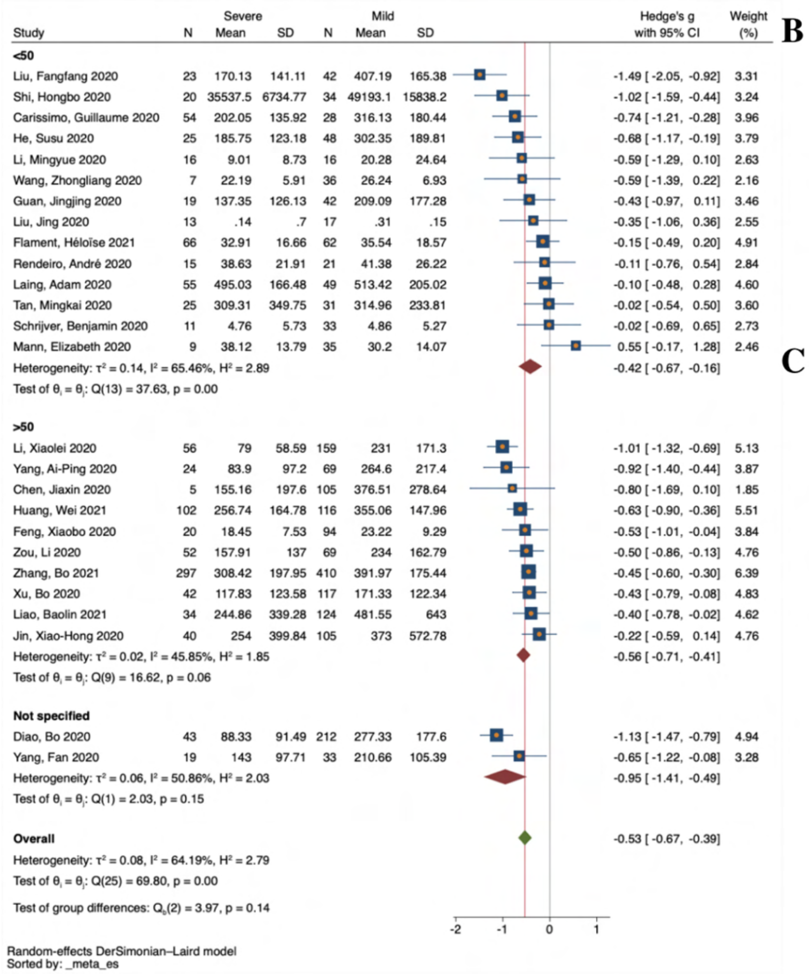

B

```
Random-effects meta-regression                 Number of obs  =        26
Method: DerSimonian-Laird                      Residual heterogeneity:
                                                          tau2 =    .06358
                                                        I2 (%) =     59.52
                                                            H2 =      2.47
                                                 R-squared (%) =     17.26
                                                  Wald chi2(1) =      3.95
                                                  Prob > chi2  =    0.0470

    _meta_es   Coefficient  Std. err.      z    P>|z|     [95% conf. interval]

   malecodes    -.2165711    .109028    -1.99   0.047     -.430262   -.0028802
       _cons    -.1764571   .1904461    -0.93   0.354    -.5497246    .1968104

Test of residual homogeneity: Q_res = chi2(24) = 59.29    Prob > Q_res = 0.0001
```

C

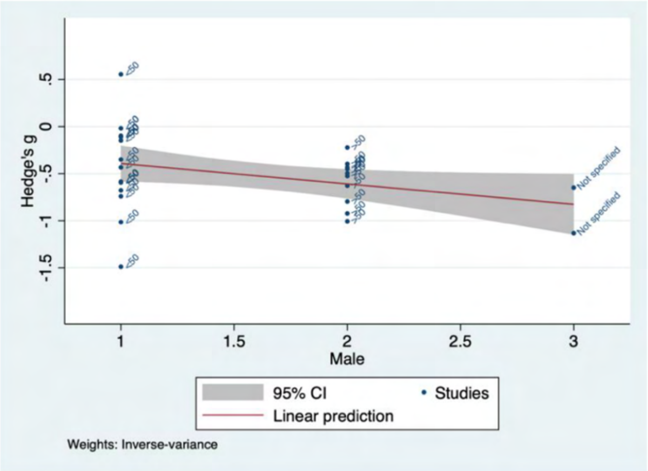

Figure S10.17. Subgroup analysis performed under the moderator (total male number) for CD8 T-cells in COVID-19 severity studies. (A) Subgroup forest plot. The no-effect line is represented at the value of zero. The diamond symbol represents estimated combined effect. (B) Subgroup meta-regression. (C) Subgroup meta-regression bubble plot. Studies are represented as (bubbles). The regression line (red). The horizontal axis represents total male number.

Subgroup analysis under moderator (total female number) for CD8 T-cell in COVID-19 severity studies

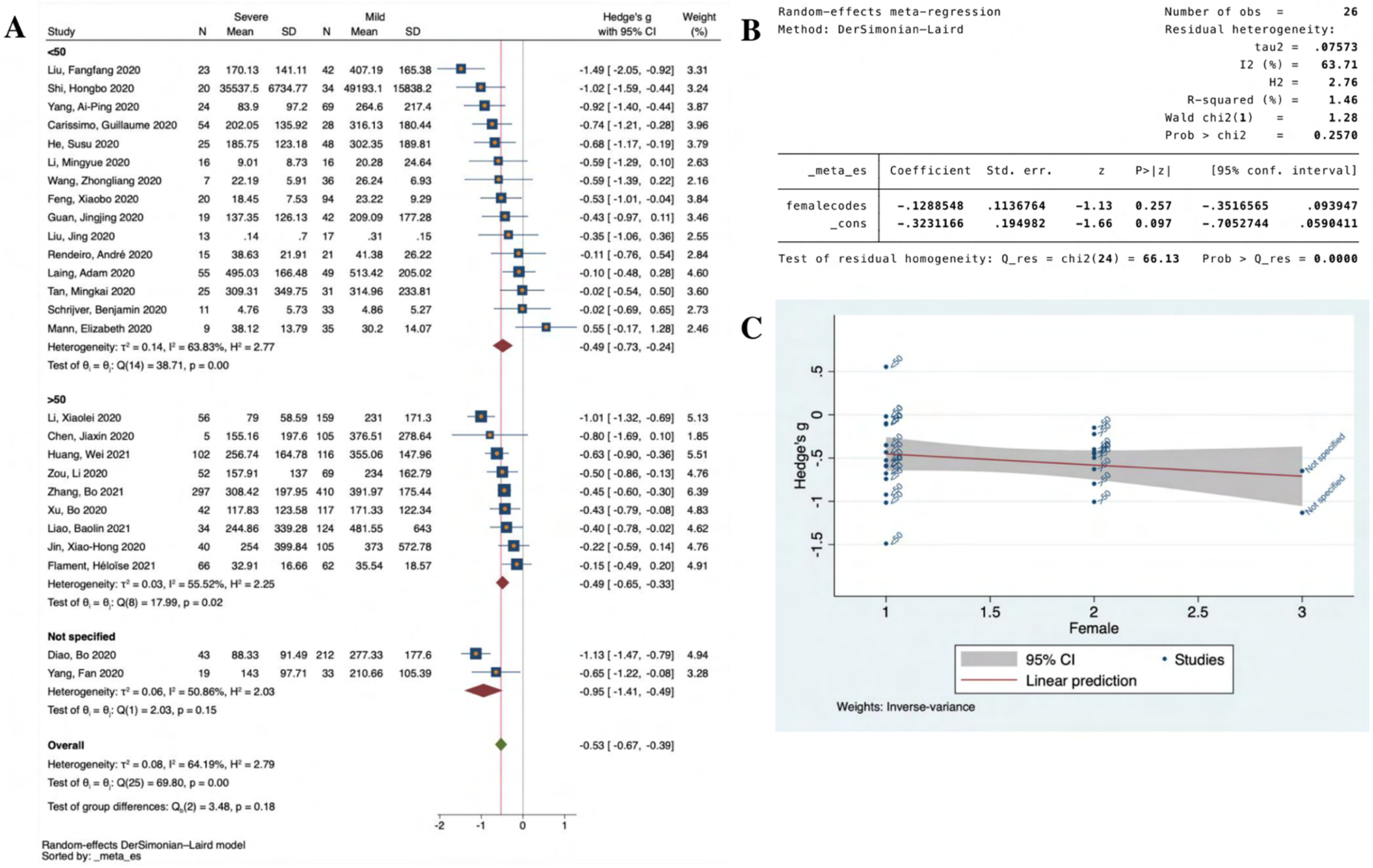

Figure S10.18. Subgroup analysis performed under the moderator (total female number) for CD8 T-cells in COVID-19 severity studies. (A) Subgroup forest plot. The no-effect line is represented at the value of zero. The diamond symbol represents estimated combined effect. (B) Subgroup meta-regression. (C) Subgroup meta-regression bubble plot. Studies are represented as (bubbles). The regression line (red). The horizontal axis represents total female number.

Subgroup analysis under moderator (mean age) for CD8 T-cell in COVID-19 severity studies

A

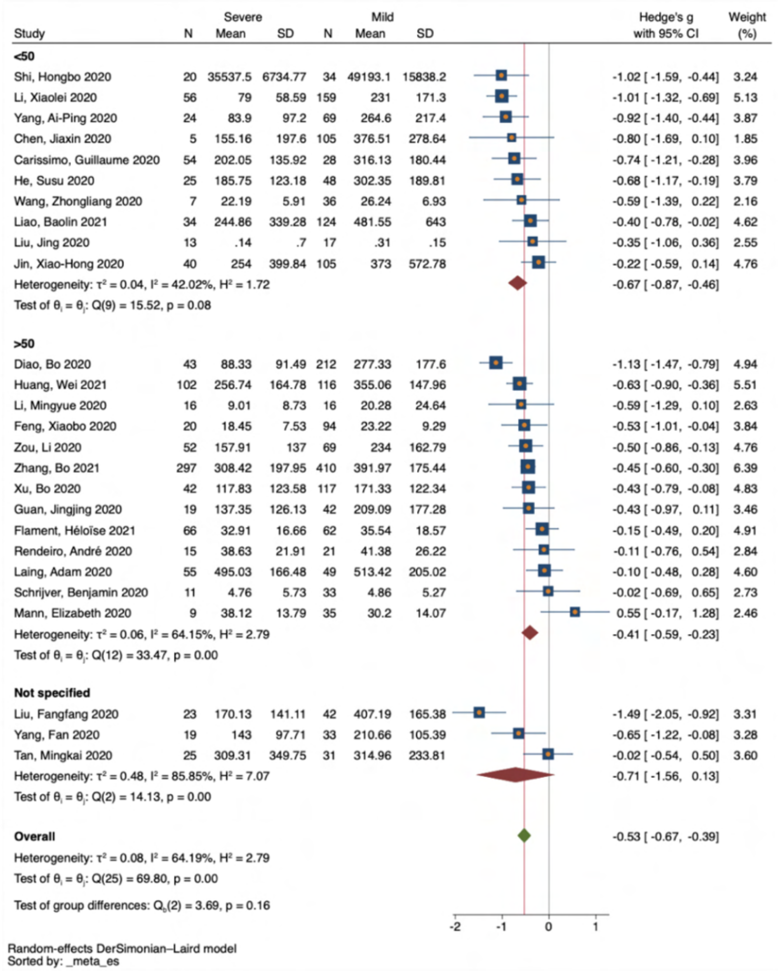

B

```
Random-effects meta-regression                 Number of obs  =         26
Method: DerSimonian-Laird                      Residual heterogeneity:
                                                          tau2 =  .07877
                                                        I2 (%) =   64.62
                                                            H2 =    2.83
                                                 R-squared (%) =    0.00
                                                Wald chi2(1)   =    0.52
                                                Prob > chi2    =  0.4701
------------------------------------------------------------------------------
    _meta_es | Coefficient  Std. err.      z    P>|z|     [95% conf. interval]
-------------+----------------------------------------------------------------
    agecodes |   .0841711    .116526     0.72   0.470    -.1442157    .3125578
       _cons |  -.6747966   .2158774    -3.13   0.002    -1.097909   -.2516847
------------------------------------------------------------------------------
Test of residual homogeneity: Q_res = chi2(24) = 67.83   Prob > Q_res = 0.0000
```

C

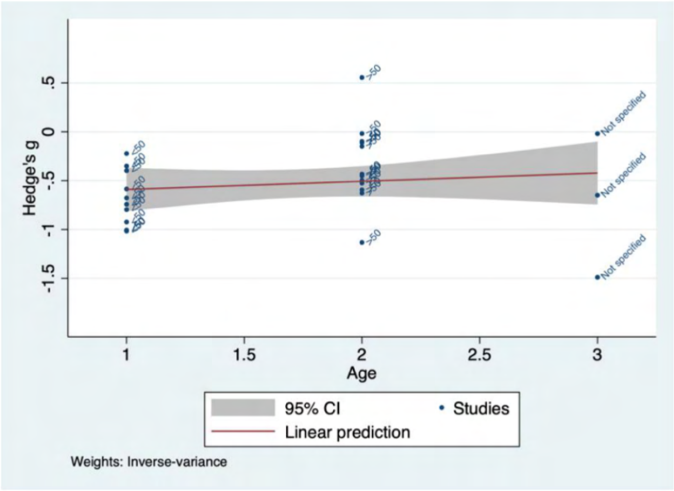

Figure S10.19. Subgroup analysis performed under the moderator (mean age) for CD8 T-cells in COVID-19 severity studies. (A) Subgroup forest plot. The no-effect line is represented at the value of zero. The diamond symbol represents estimated combined effect. (B) Subgroup meta-regression. (C) Subgroup meta-regression bubble plot. Studies are represented as (bubbles). The regression line (red). The horizontal axis represents mean age.

Subgroup analysis under moderator (test procedure) for CD8 T-cell in COVID-19 severity studies

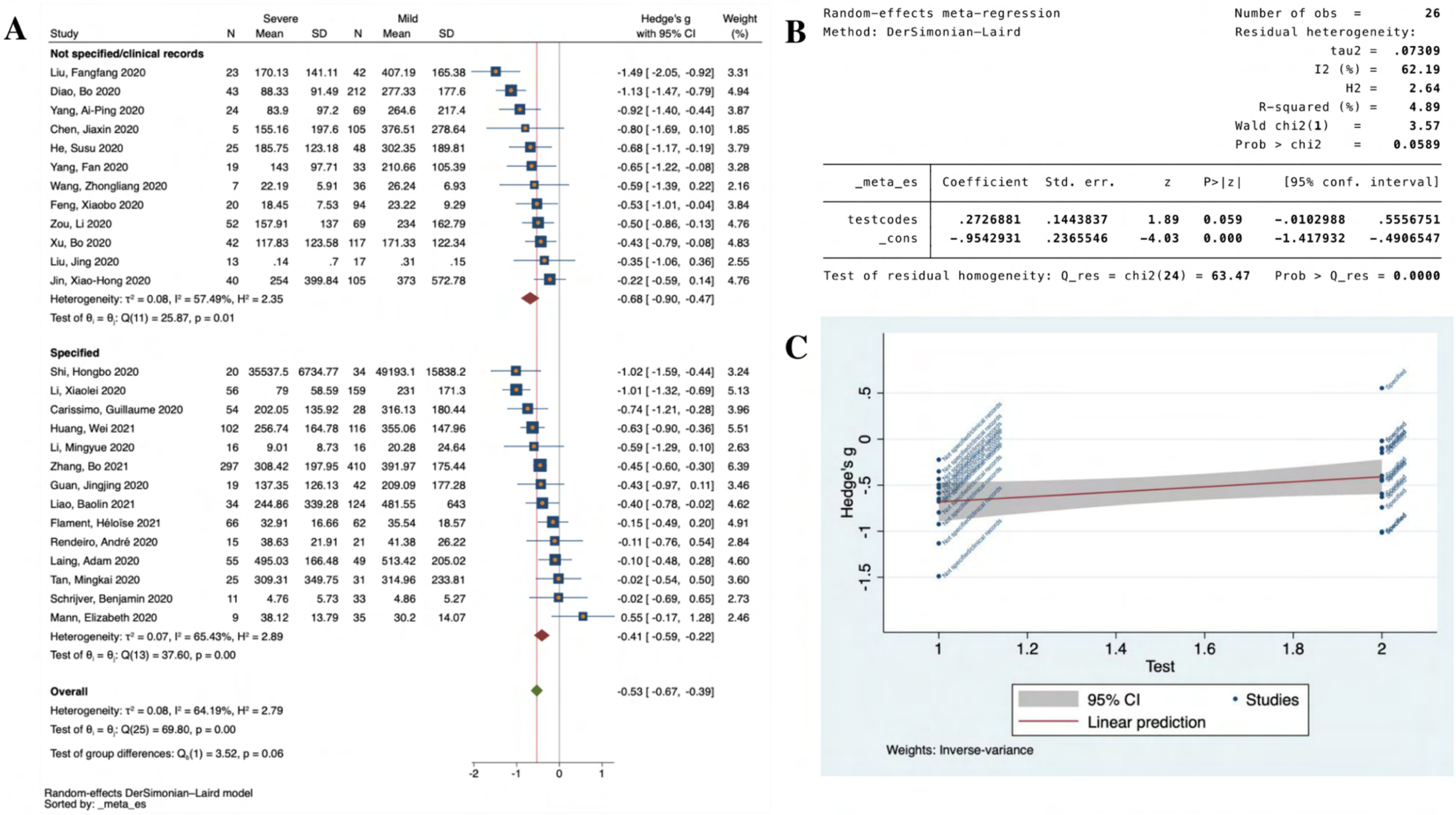

Figure S10.20. Subgroup analysis performed under the moderator (test procedure) for CD8 T-cells in COVID-19 severity studies. (A) Subgroup forest plot. The no-effect line is represented at the value of zero. The diamond symbol represents estimated combined effect. (B) Subgroup meta-regression. (C) Subgroup meta-regression bubble plot. Studies are represented as (bubbles). The regression line (red). The horizontal axis represents test procedure.

Subgroup analysis under moderator (city) for IL-10 in COVID-19 severity studies

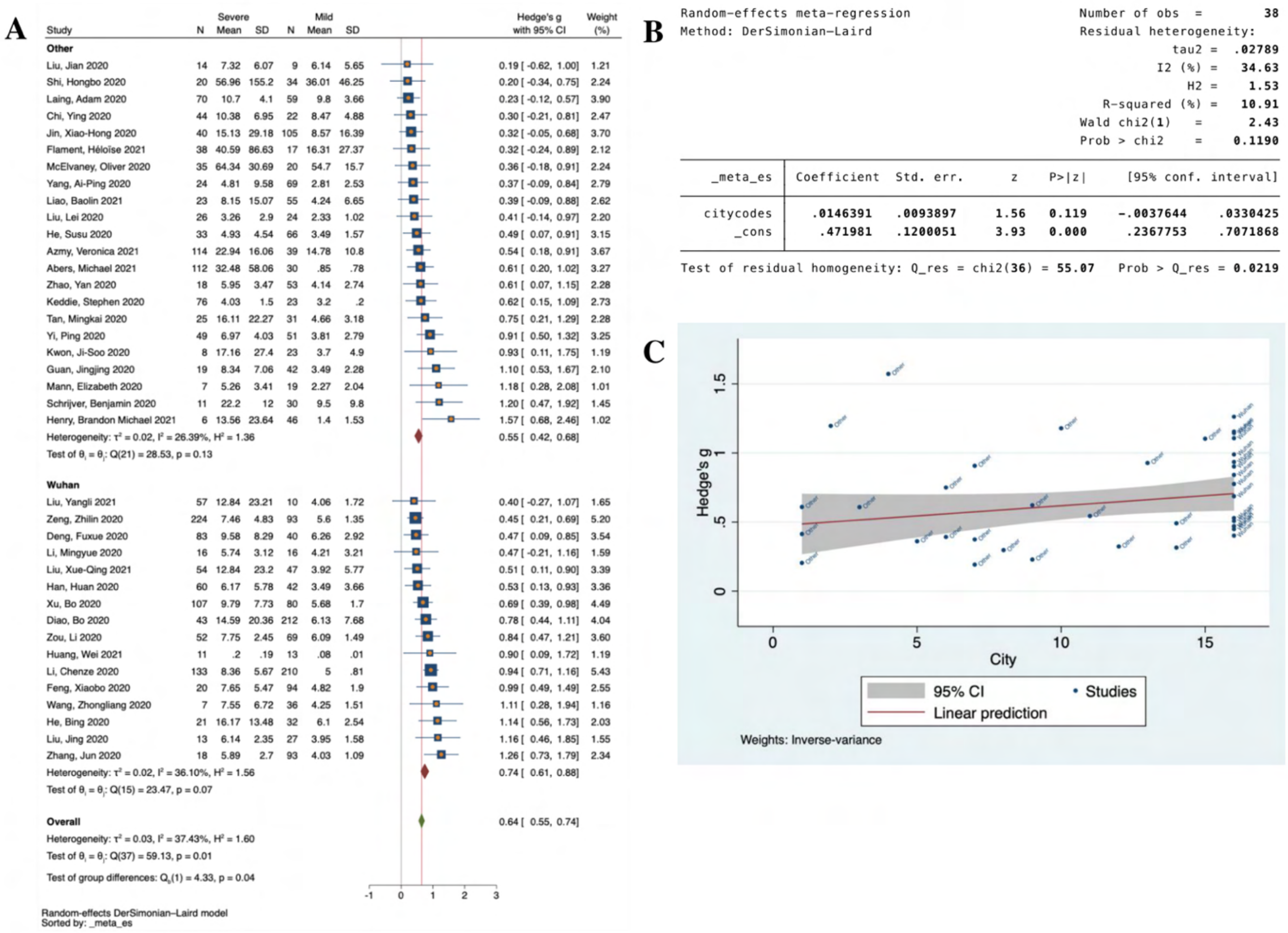

Figure S10.21. Subgroup analysis performed under the moderator (city) for IL-10 in COVID-19 severity studies. (A) Subgroup forest plot. The no-effect line is represented at the value of zero. The diamond symbol represents estimated combined effect. (B) Subgroup meta-regression. (C) Subgroup meta-regression bubble plot. Studies are represented as (bubbles). The regression line (red). The horizontal axis represents cities.

Subgroup analysis under moderator (country) for IL-10 in COVID-19 severity studies

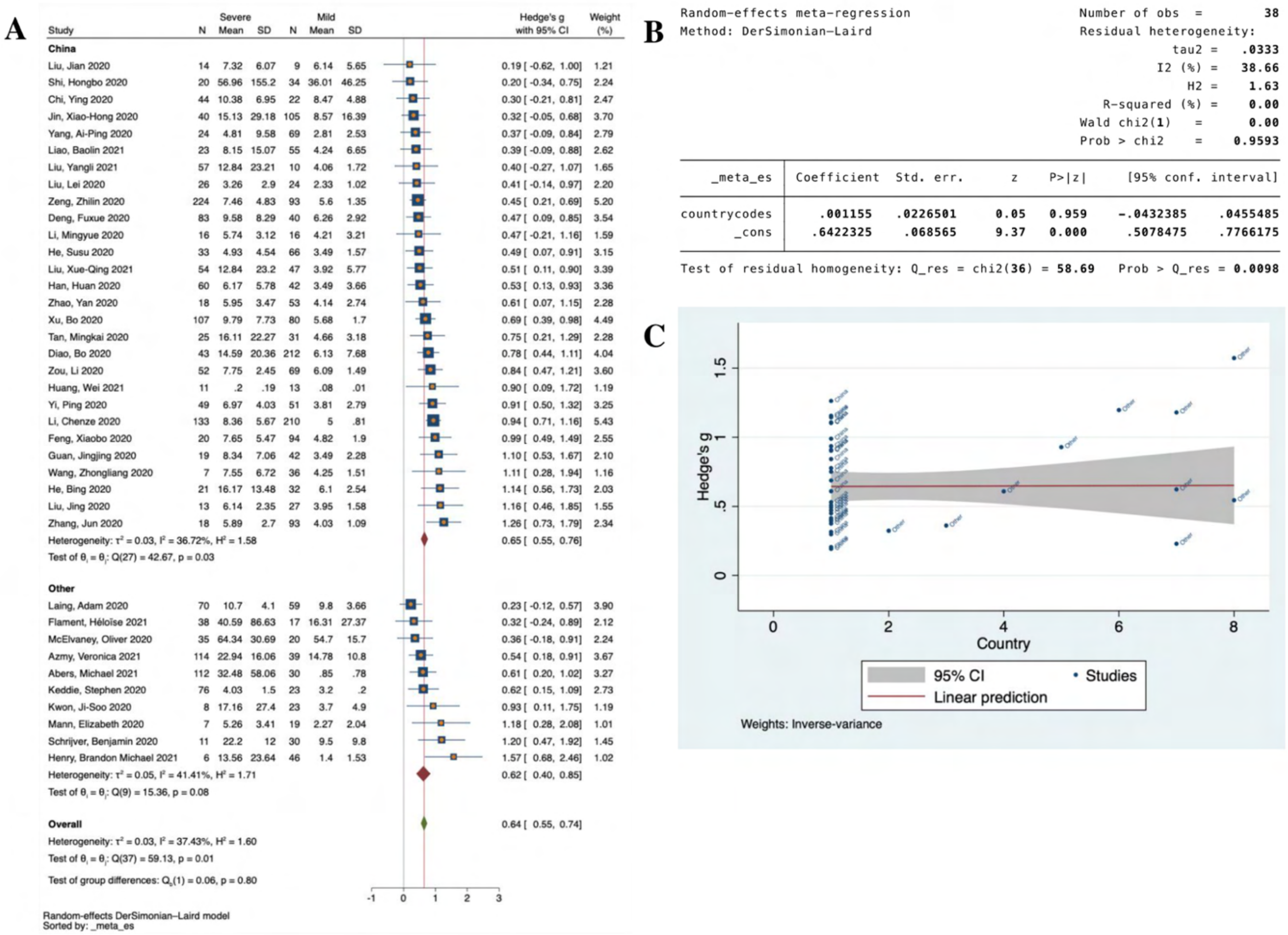

Figure S10.22. Subgroup analysis performed under the moderator (country) for IL-10 in COVID-19 severity studies. (A) Subgroup forest plot. The no-effect line is represented at the value of zero. The diamond symbol represents estimated combined effect. (B) Subgroup meta-regression. (C) Subgroup meta-regression bubble plot. Studies are represented as (bubbles). The regression line (red). The horizontal axis represents countries.

Subgroup analysis under moderator (continent) for IL-10 in COVID-19 severity studies

---

A

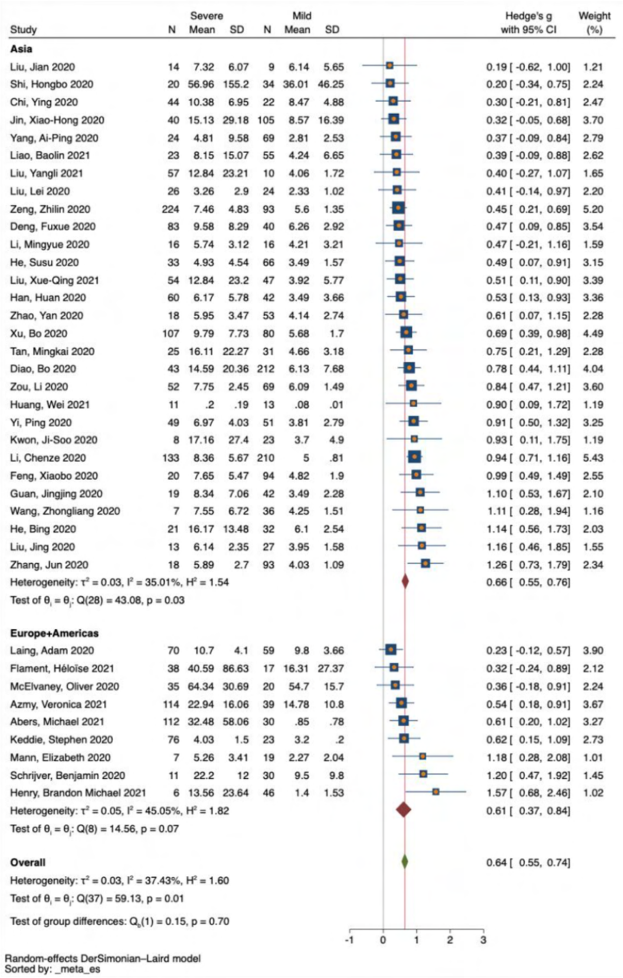

B

```
Random-effects meta-regression                    Number of obs  =          38
Method: DerSimonian-Laird                         Residual heterogeneity:
                                                              tau2 =    .03077
                                                            I2 (%) =     36.91
                                                                H2 =      1.59
                                                     R-squared (%) =      1.71
                                                  Wald chi2(1)     =      1.15
                                                  Prob > chi2      =    0.2845

      _meta_es | Coefficient  Std. err.      z    P>|z|     [95% conf. interval]
---------------+----------------------------------------------------------------
continentcodes |  -.1178141   .1100771    -1.07   0.284    -.3335612    .0979331
         _cons |   .8937855   .2385001     3.75   0.000     .4263338    1.361237

Test of residual homogeneity: Q_res = chi2(36) = 57.06   Prob > Q_res = 0.0142
```

C

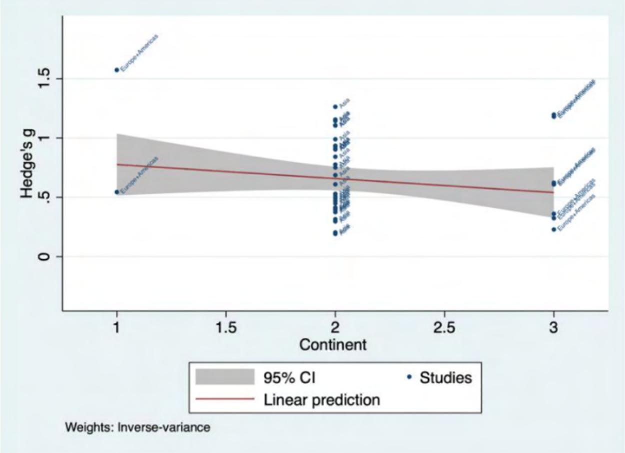

Figure S10.23. Subgroup analysis performed under the moderator (continent) for IL-10 in COVID-19 severity studies. (A) Subgroup forest plot. The no-effect line is represented at the value of zero. The diamond symbol represents estimated combined effect. (B) Subgroup meta-regression. (C) Subgroup meta-regression bubble plot. Studies are represented as (bubbles). The regression line (red). The horizontal axis represents continents.

Subgroup analysis under moderator (study design) for IL-10 in COVID-19 severity studies

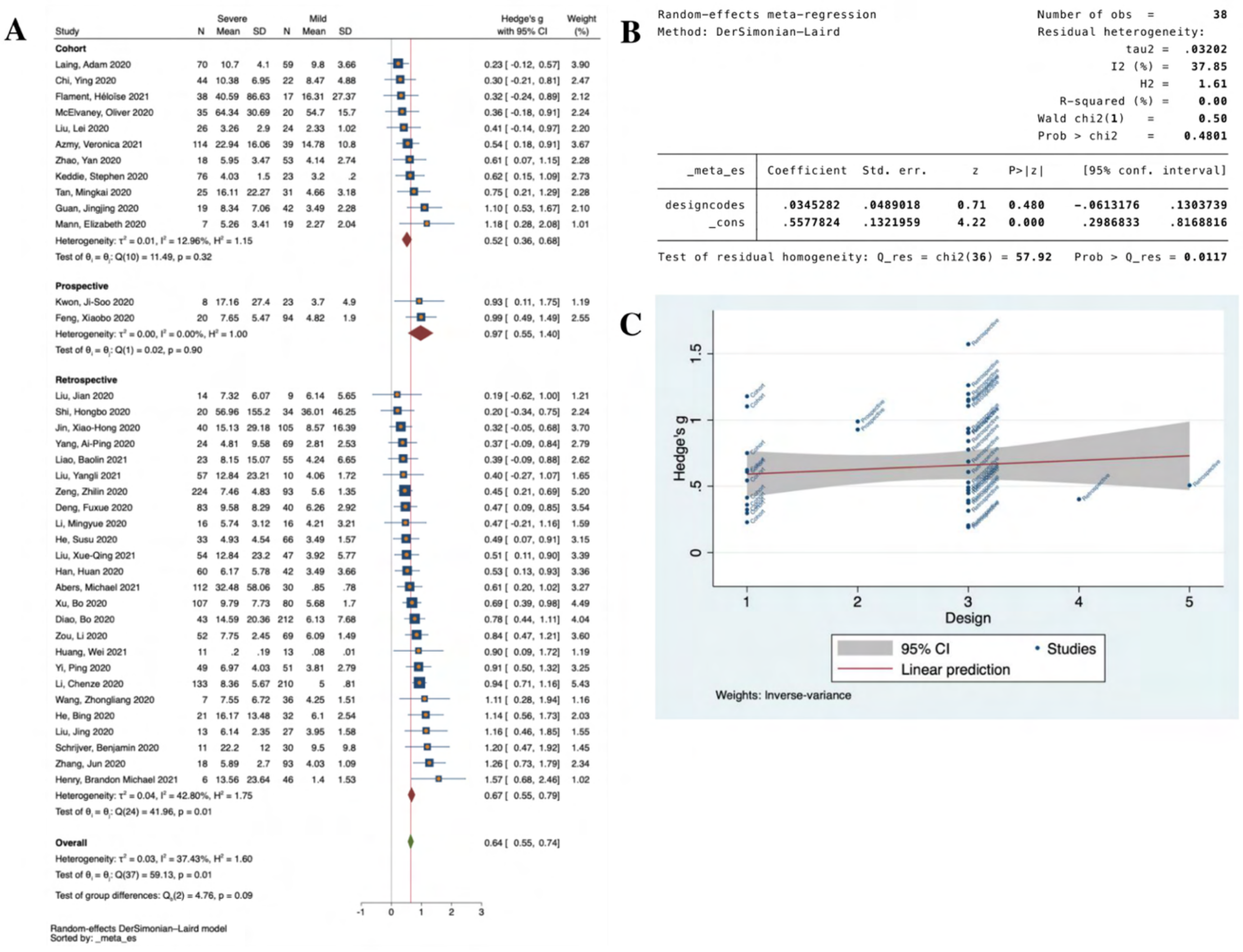

Figure S10.24. Subgroup analysis performed under the moderator (study design) for IL-10 in COVID-19 severity studies. (A) Subgroup forest plot. The no-effect line is represented at the value of zero. The diamond symbol represents estimated combined effect. (B) Subgroup meta-regression. (C) Subgroup meta-regression bubble plot. Studies are represented as (bubbles). The regression line (red). The horizontal axis represents study design.

Subgroup analysis under moderator (classification protocol) for IL-10 in COVID-19 severity studies

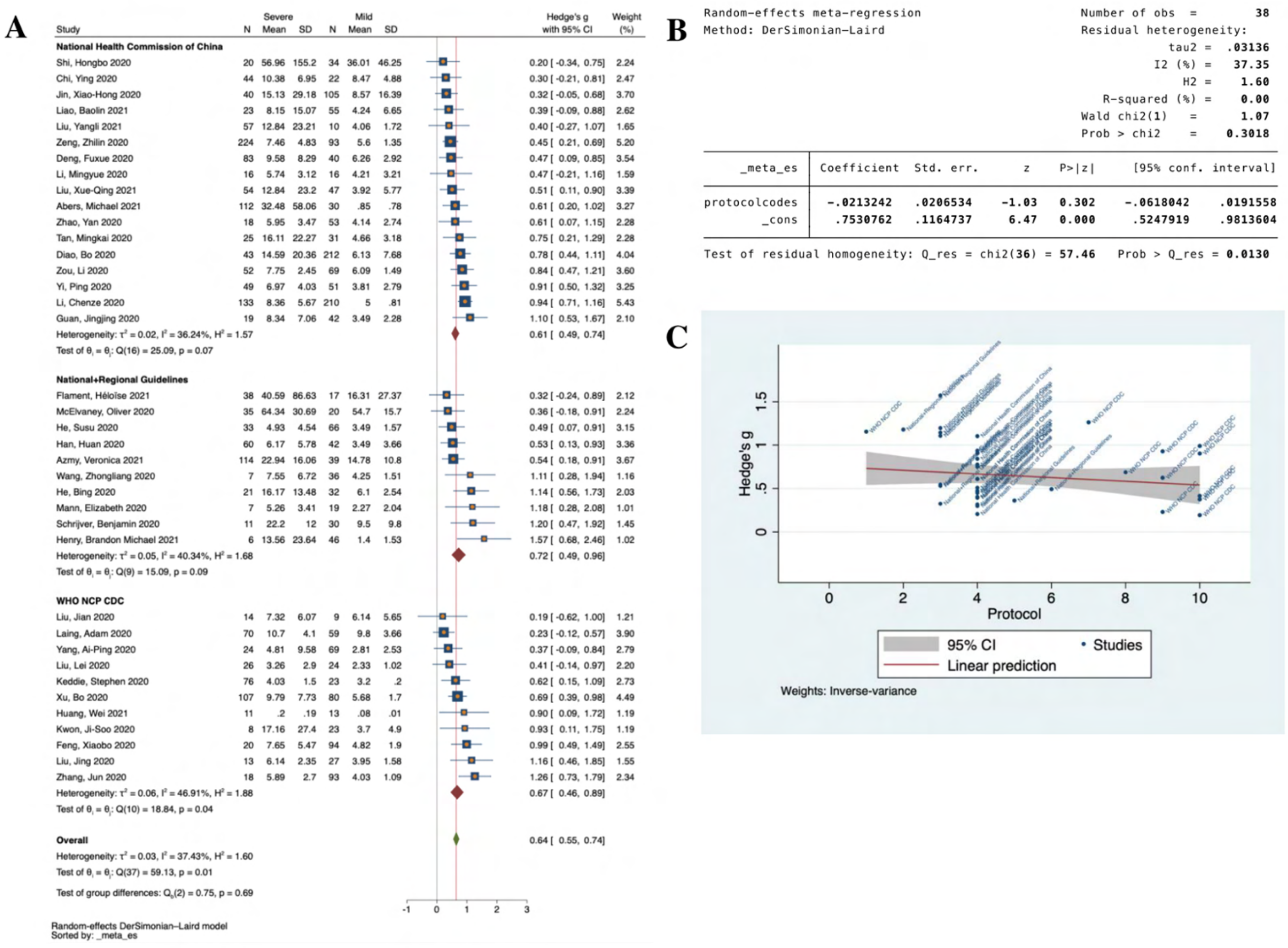

Figure S10.25. Subgroup analysis performed under the moderator (classification protocol) for IL-10 in COVID-19 severity studies. (A) Subgroup forest plot. The no-effect line is represented at the value of zero. The diamond symbol represents estimated combined effect. (B) Subgroup meta-regression. (C) Subgroup meta-regression bubble plot. Studies are represented as (bubbles). The regression line (red). The horizontal axis represents classification protocols.

Subgroup analysis under moderator (sample acquisition time) for IL-10 in COVID-19 severity studies

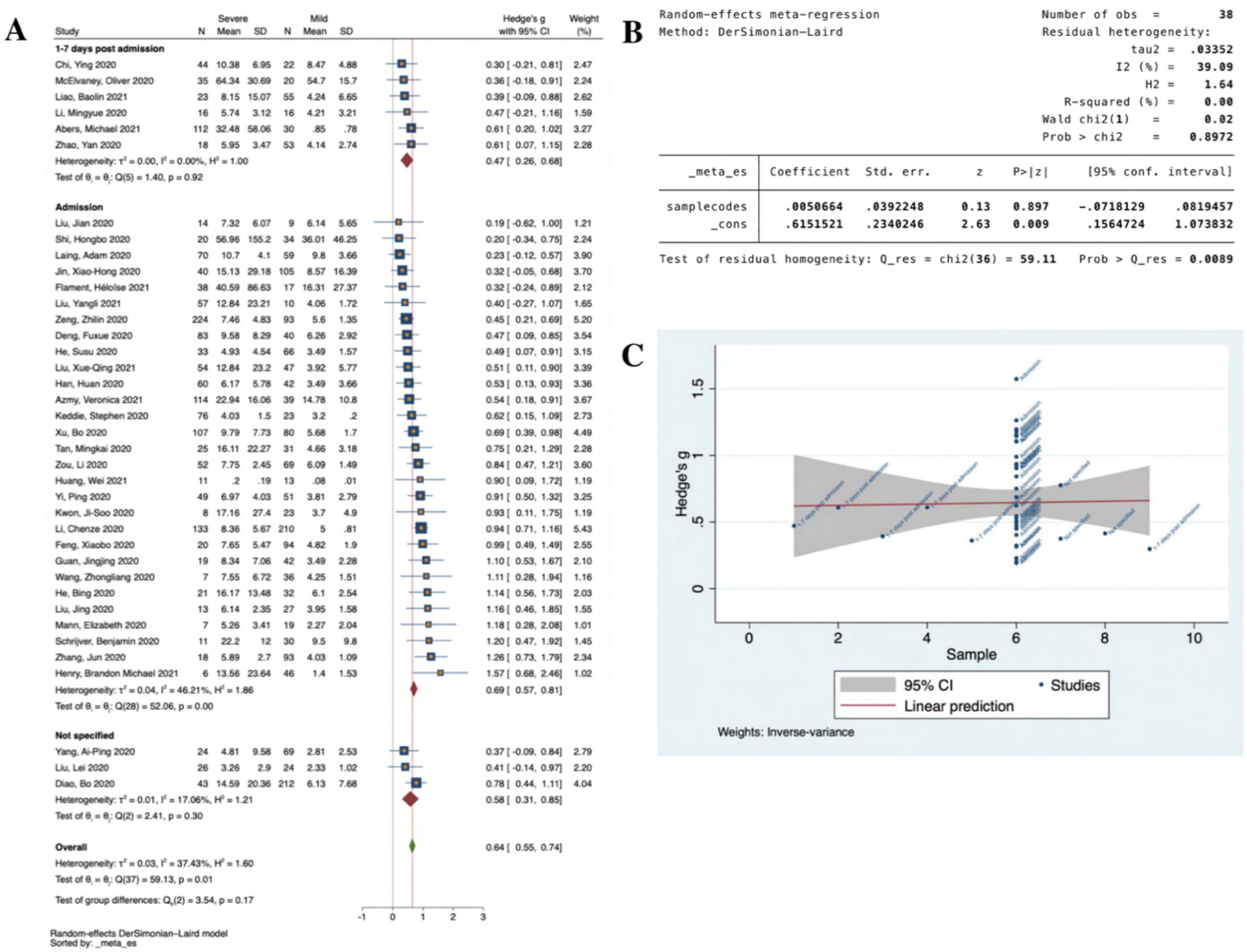

Figure S10.26. Subgroup analysis performed under the moderator (sample acquisition time) for IL-10 in COVID-19 severity studies. (A) Subgroup forest plot. The no-effect line is represented at the value of zero. The diamond symbol represents estimated combined effect. (B) Subgroup meta-regression. (C) Subgroup meta-regression bubble plot. Studies are represented as (bubbles). The regression line (red). The horizontal axis represents sample acquisition time.

Subgroup analysis under moderator (total male number) for IL-10 in COVID-19 severity studies

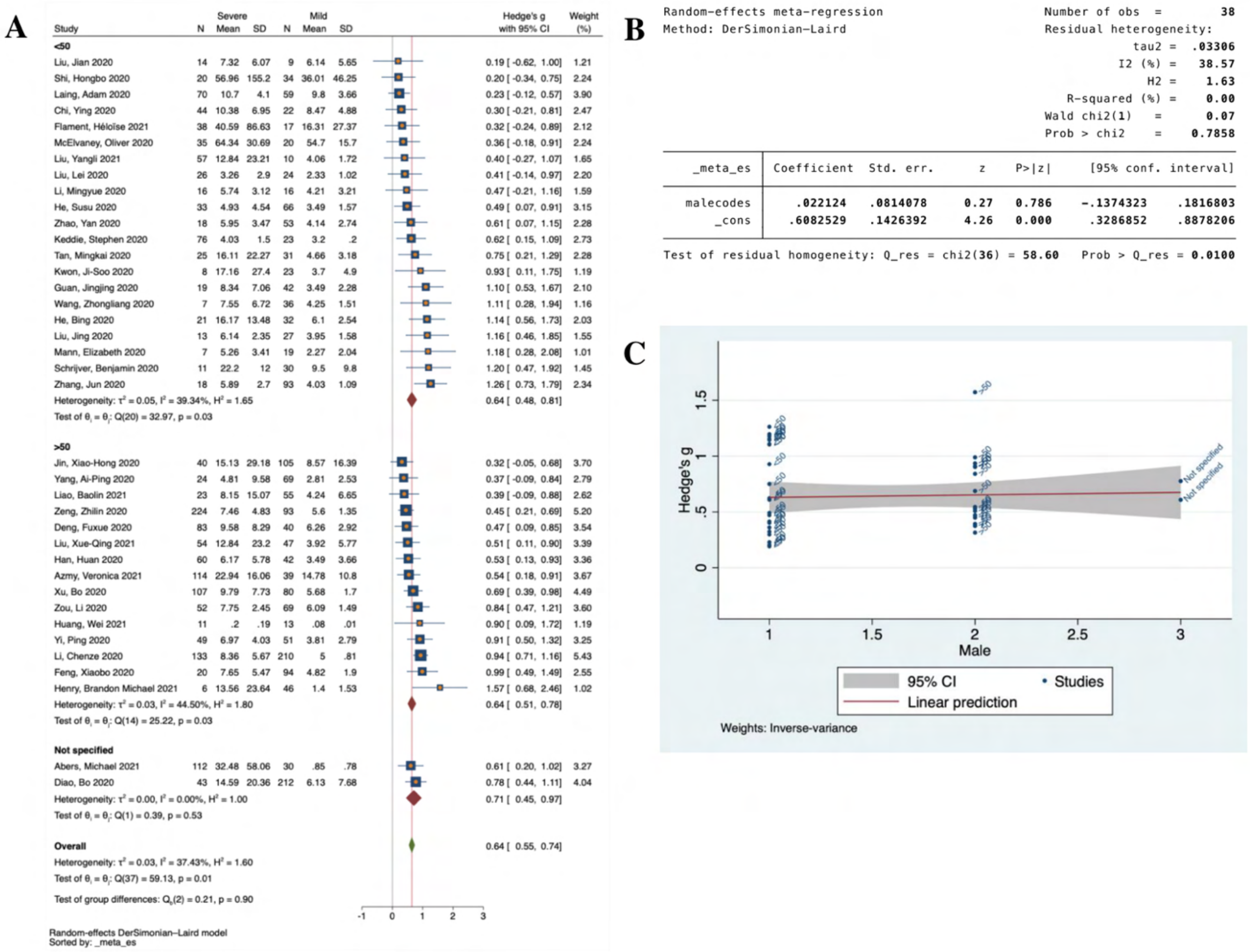

Figure S10.27. Subgroup analysis performed under the moderator (total male number) for IL-10 in COVID-19 severity studies. (A) Subgroup forest plot. The no-effect line is represented at the value of zero. The diamond symbol represents estimated combined effect. (B) Subgroup meta-regression. (C) Subgroup meta-regression bubble plot. Studies are represented as (bubbles). The regression line (red). The horizontal axis represents total male number.

Subgroup analysis under moderator (total female number) for IL-10 in COVID-19 severity studies

A

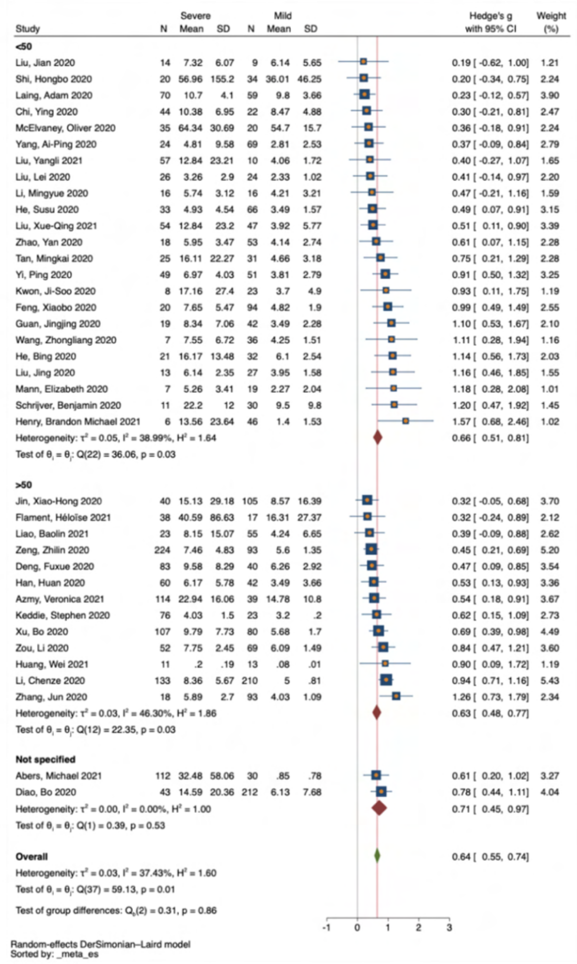

B

```
Random-effects meta-regression                  Number of obs  =         38
Method: DerSimonian–Laird                       Residual heterogeneity:
                                                            tau2 =   .03359
                                                          I2 (%) =    38.88
                                                              H2 =     1.64
                                                   R-squared (%) =     0.00
                                                   Wald chi2(1)  =     0.00
                                                   Prob > chi2   =   0.9881

    _meta_es   Coefficient  Std. err.      z    P>|z|     [95% conf. interval]

 femalecodes     .0011993   .0802941    0.01   0.988    -.1561743    .1585728
       _cons     .6427994   .1365837    4.71   0.000     .3751003    .9104985

Test of residual homogeneity: Q_res = chi2(36) = 58.90   Prob > Q_res = 0.0094
```

C

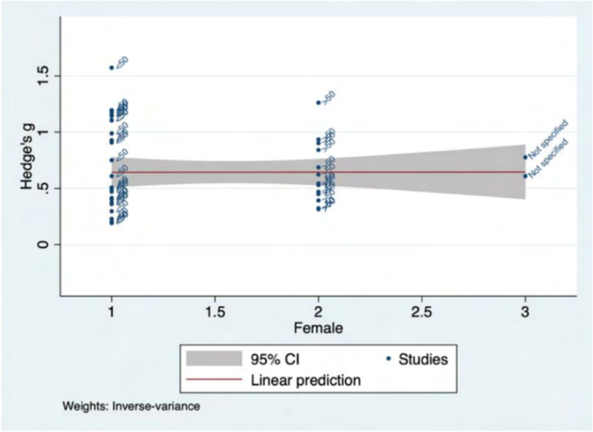

Figure S10.28. Subgroup analysis performed under the moderator (total female number) for IL-10 in COVID-19 severity studies. (A) Subgroup forest plot. The no-effect line is represented at the value of zero. The diamond symbol represents estimated combined effect. (B) Subgroup meta-regression. (C) Subgroup meta-regression bubble plot. Studies are represented as (bubbles). The regression line (red). The horizontal axis represents total female number.

Subgroup analysis under moderator (mean age) for IL-10 in COVID-19 severity studies

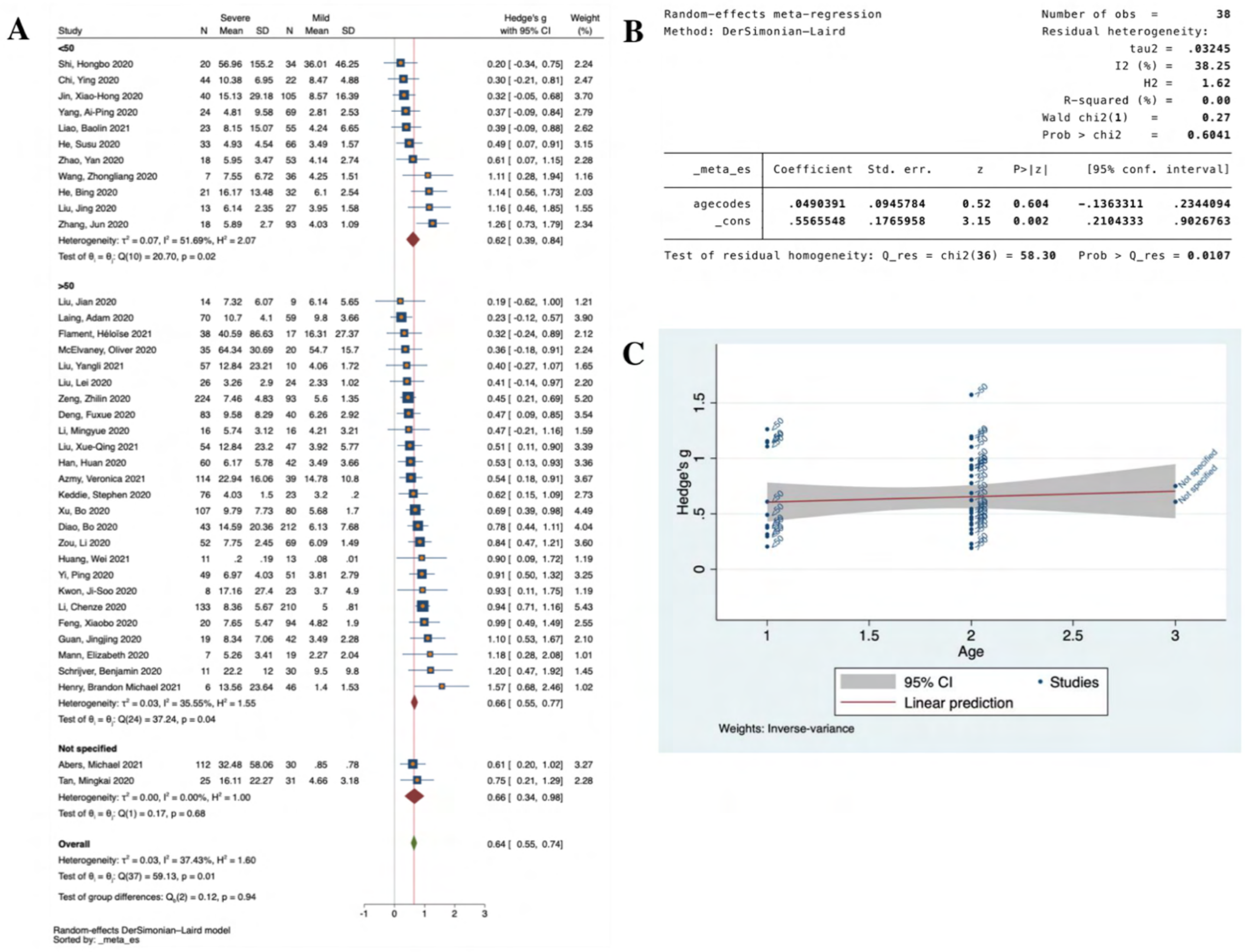

Figure S10.29. Subgroup analysis performed under the moderator (mean age) for IL-10 in COVID-19 severity studies. (A) Subgroup forest plot. The no-effect line is represented at the value of zero. The diamond symbol represents estimated combined effect. (B) Subgroup meta-regression. (C) Subgroup meta-regression bubble plot. Studies are represented as (bubbles). The regression line (red). The horizontal axis represents mean age.

Subgroup analysis under moderator (test procedure) for IL-10 in COVID-19 severity studies

---

A

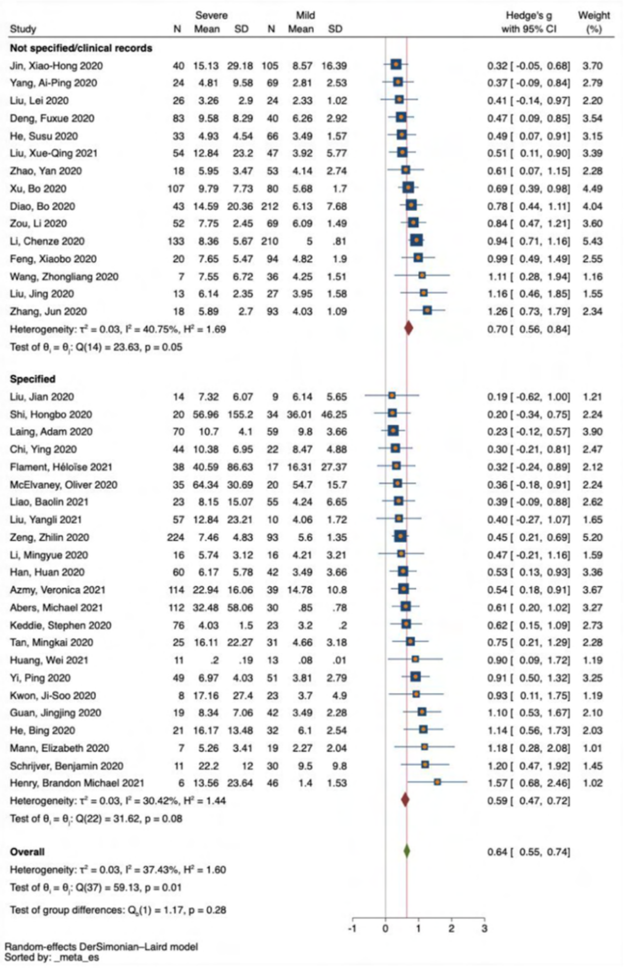

B

```
Random-effects meta-regression                  Number of obs  =         38
Method: DerSimonian–Laird                       Residual heterogeneity:
                                                        tau2 =   .02848
                                                      I2 (%) =    34.84
                                                          H2 =     1.53
                                                 R-squared (%) =    9.05
                                                 Wald chi2(1)  =     1.17
                                                 Prob > chi2   =   0.2797

  _meta_es   Coefficient  Std. err.      z    P>|z|     [95% conf. interval]

 testcodes    -.1049805   .0971216   -1.08   0.280    -.2953354    .0853743
     _cons     .8046741   .1568348    5.13   0.000     .4972835    1.112065

Test of residual homogeneity: Q_res = chi2(36) = 55.25   Prob > Q_res = 0.0211
```

C

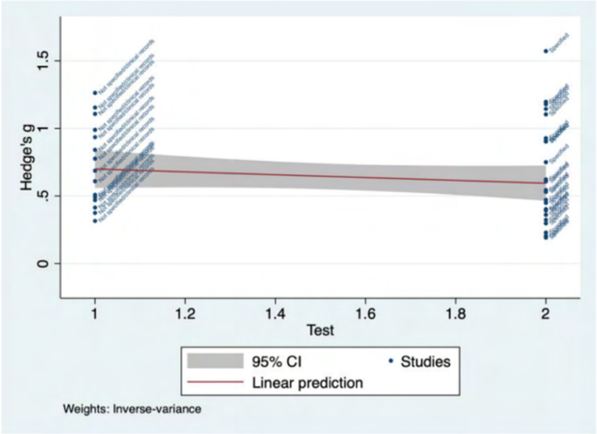

Figure S10.30. Subgroup analysis performed under the moderator (test procedure) for IL-10 in COVID-19 severity studies. (A) Subgroup forest plot. The no-effect line is represented at the value of zero. The diamond symbol represents estimated combined effect. (B) Subgroup meta-regression. (C) Subgroup meta-regression bubble plot. Studies are represented as (bubbles). The regression line (red). The horizontal axis represents test procedure.

Subgroup analysis under moderator (city) for CD4 T-cell in COVID-19 mortality studies

A

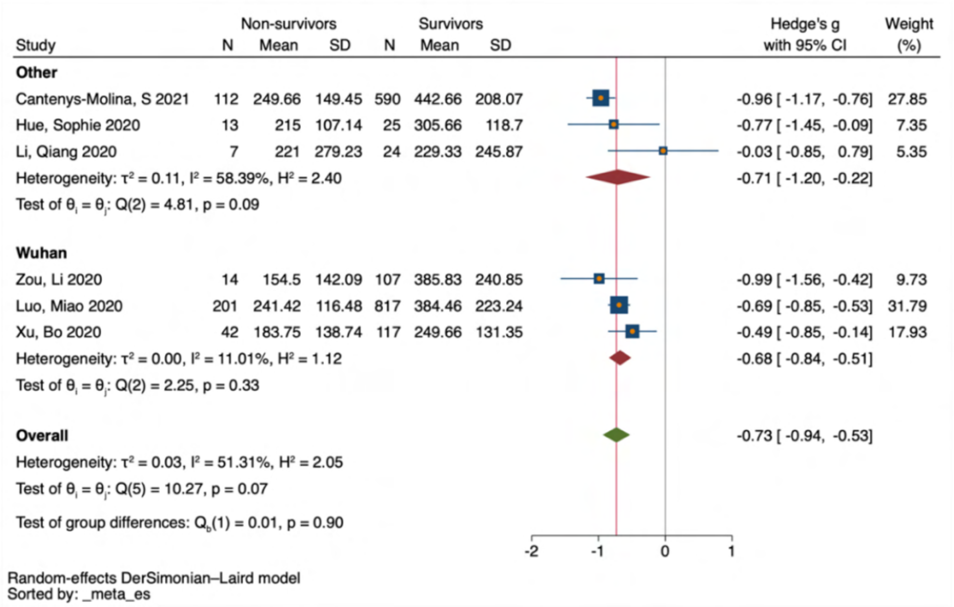

B

```
Random-effects meta-regression                  Number of obs  =          6
Method: DerSimonian-Laird                       Residual heterogeneity:
                                                           tau2 =    .03198
                                                         I2 (%) =     43.30
                                                             H2 =      1.76
                                                   R-squared (%) =      0.00
                                                Wald chi2(1)    =      0.27
                                                Prob > chi2     =    0.6011

    _meta_es   Coefficient  Std. err.      z    P>|z|     [95% conf. interval]

   citycodes     .1156111   .2211452     0.52   0.601    -.3178256    .5490477
       _cons    -.9128987   .3684517    -2.48   0.013    -1.635051   -.1907467

Test of residual homogeneity: Q_res = chi2(4) =  7.05    Prob > Q_res = 0.1330
```

C

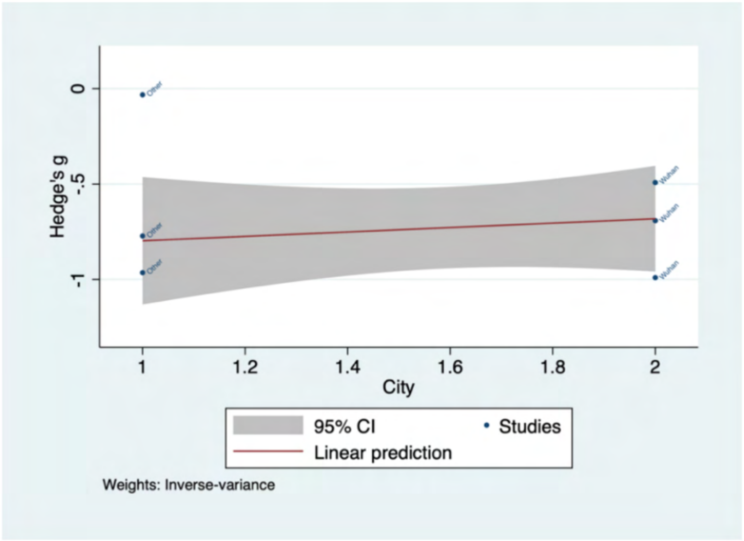

Figure S10.31. Subgroup analysis performed under the moderator (city) for CD4 T-cells in COVID-19 mortality studies. (A) Subgroup forest plot. The no-effect line is represented at the value of zero. The diamond symbol represents estimated combined effect. (B) Subgroup meta-regression. (C) Subgroup meta-regression bubble plot. Studies are represented as (bubbles). The regression line (red). The horizontal axis represents cities.

Subgroup analysis under moderator (country) for CD4 T-cell in COVID-19 mortality studies

A

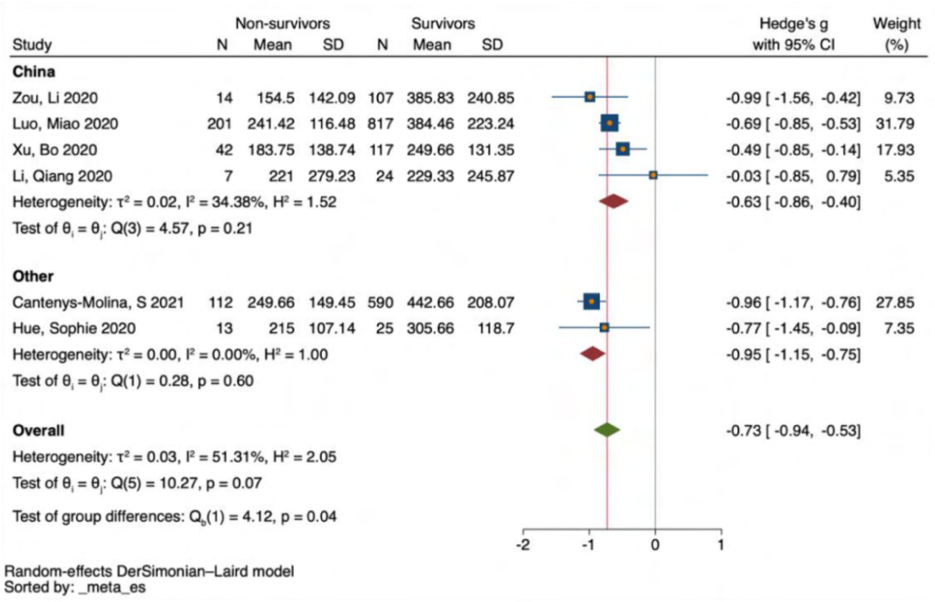

B

```
Random-effects meta-regression                  Number of obs  =          6
Method: DerSimonian–Laird                       Residual heterogeneity:
                                                           tau2 = .009012
                                                         I2 (%) =   17.59
                                                             H2 =    1.21
                                                  R-squared (%) =   67.46
                                                  Wald chi2(1)  =    3.23
                                                  Prob > chi2   =  0.0724

      _meta_es | Coefficient  Std. err.      z    P>|z|     [95% conf. interval]
---------------+----------------------------------------------------------------
  countrycodes |  -.2954842   .1644771    -1.80   0.072    -.6178534    .0268851
         _cons |   -.347221   .2359064    -1.47   0.141     -.809589    .1151471

Test of residual homogeneity: Q_res = chi2(4) =  4.85    Prob > Q_res = 0.3027
```

C

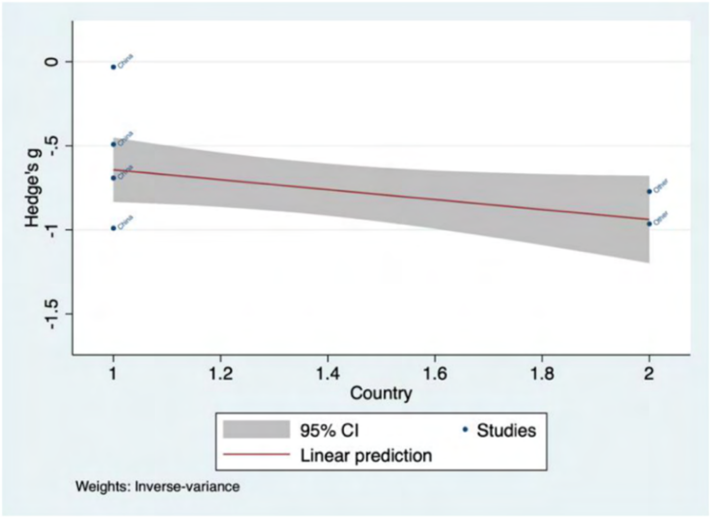

Figure S10.32. Subgroup analysis performed under the moderator (country) for CD4 T-cells in COVID-19 mortality studies. (A) Subgroup forest plot. The no-effect line is represented at the value of zero. The diamond symbol represents estimated combined effect. (B) Subgroup meta-regression. (C) Subgroup meta-regression bubble plot. Studies are represented as (bubbles). The regression line (red). The horizontal axis represents countries.

Subgroup analysis under moderator (continent) for CD4 T-cell in COVID-19 mortality studies

---

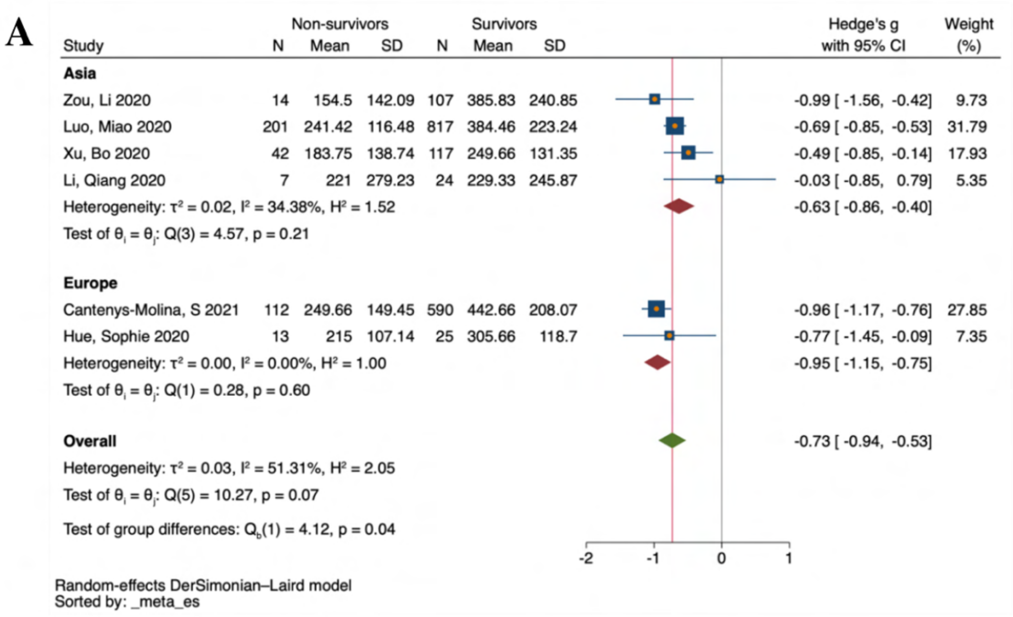

B

```
Random-effects meta-regression                  Number of obs  =          6
Method: DerSimonian-Laird                       Residual heterogeneity:
                                                           tau2 = .009012
                                                          I2 (%) =   17.59
                                                              H2 =    1.21
                                                     R-squared (%) =   67.46
                                                     Wald chi2(1)  =    3.23
                                                     Prob > chi2   =  0.0724

       _meta_es   Coefficient  Std. err.      z    P>|z|     [95% conf. interval]

 continentcodes    -.2954842   .1644771   -1.80   0.072    -.6178534    .0268851
          _cons     -.347221   .2359064   -1.47   0.141     -.809589    .1151471

Test of residual homogeneity: Q_res = chi2(4) =  4.85    Prob > Q_res = 0.3027
```

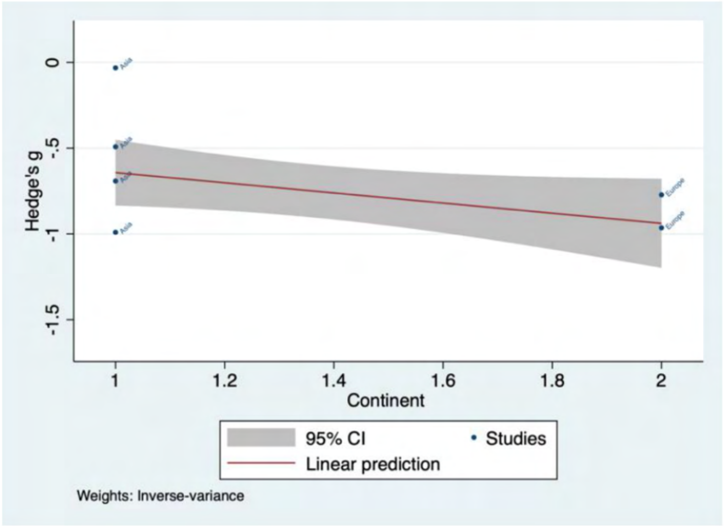

Figure S10.33. Subgroup analysis performed under the moderator (continent) for CD4 T-cells in COVID-19 mortality studies. (A) Subgroup forest plot. The no-effect line is represented at the value of zero. The diamond symbol represents estimated combined effect. (B) Subgroup meta-regression. (C) Subgroup meta-regression bubble plot. Studies are represented as (bubbles). The regression line (red). The horizontal axis represents continents.

Subgroup analysis under moderator (study design) for CD4 T-cell in COVID-19 mortality studies

A

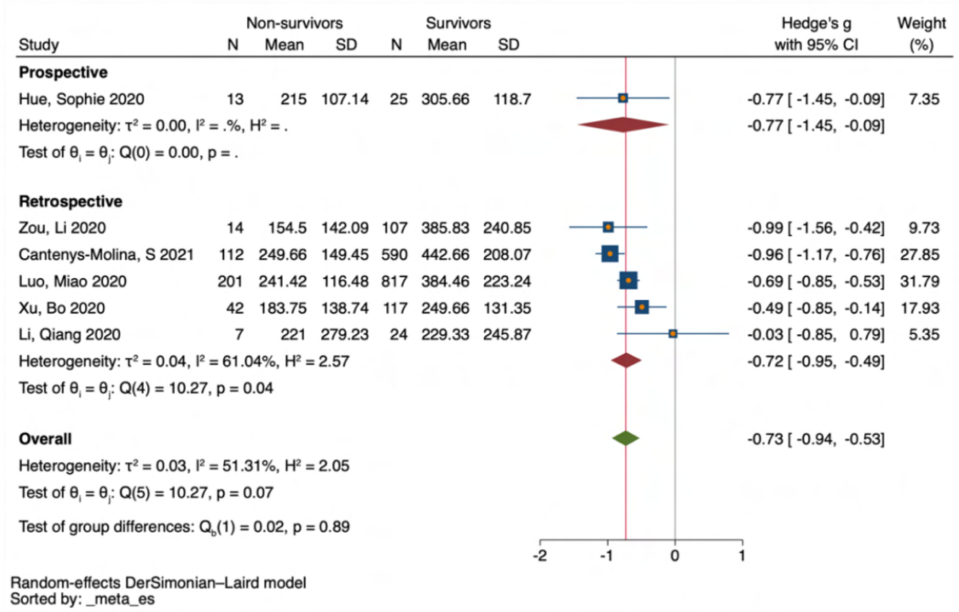

B

```
Random-effects meta-regression                    Number of obs  =          6
Method: DerSimonian-Laird                         Residual heterogeneity:
                                                              tau2 =   .03501
                                                            I2 (%) =    61.04
                                                                H2 =     2.57
                                                       R-squared (%) =     0.00
                                                  Wald chi2(1)   =       0.01
                                                  Prob > chi2    =     0.9054

     _meta_es | Coefficient  Std. err.      z    P>|z|     [95% conf. interval]
--------------+----------------------------------------------------------------
  designcodes |   .0487621   .4104738     0.12   0.905    -.7557518    .8532759
        _cons |  -.8207331   .7957605    -1.03   0.302    -2.380395    .7389288

Test of residual homogeneity: Q_res = chi2(4) = 10.27     Prob > Q_res = 0.0362
.
```

C

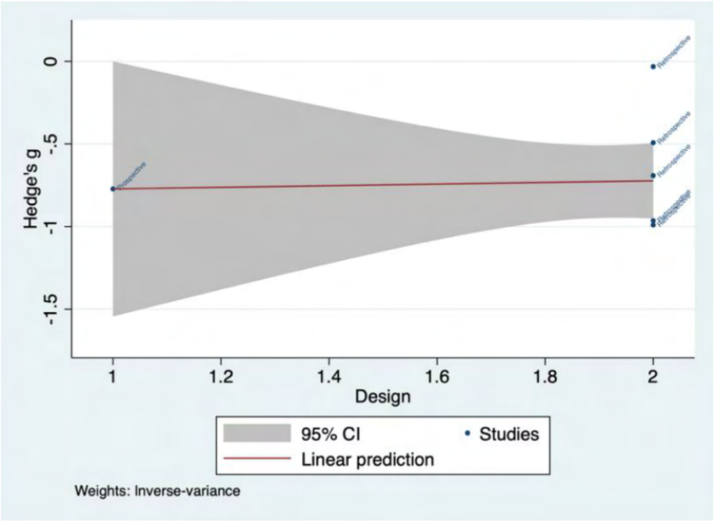

Figure S10.34. Subgroup analysis performed under the moderator (study design) for CD4 T-cells in COVID-19 mortality studies. (A) Subgroup forest plot. The no-effect line is represented at the value of zero. The diamond symbol represents estimated combined effect. (B) Subgroup meta-regression. (C) Subgroup meta-regression bubble plot. Studies are represented as (bubbles). The regression line (red). The horizontal axis represents study design.

Subgroup analysis under moderator (classification protocol) for CD4 T-cell in COVID-19 mortality studies

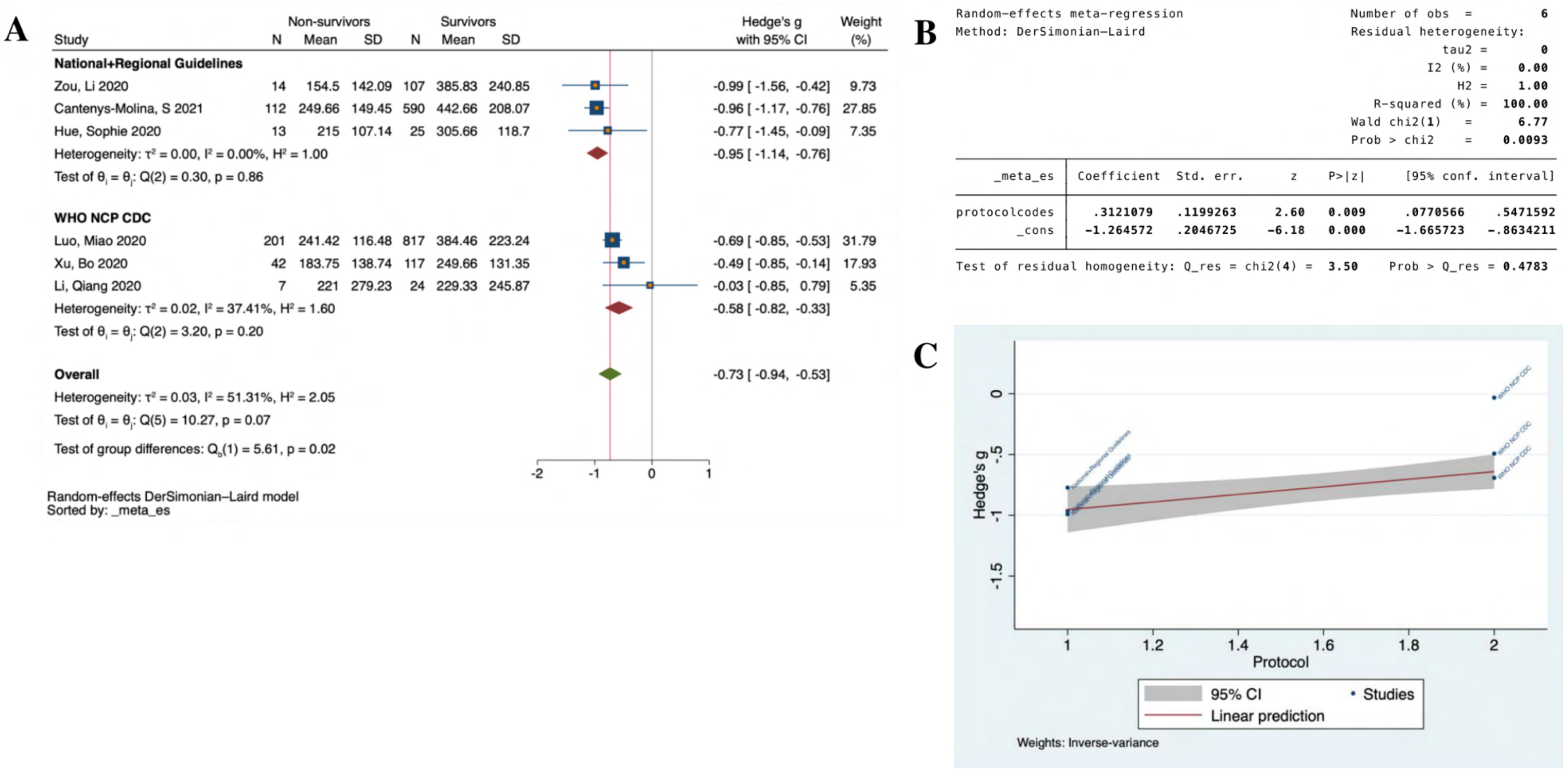

Figure S10.35. Subgroup analysis performed under the moderator (classification protocol) for CD4 T-cells in COVID-19 mortality studies. (A) Subgroup forest plot. The no-effect line is represented at the value of zero. The diamond symbol represents estimated combined effect. (B) Subgroup meta-regression. (C) Subgroup meta-regression bubble plot. Studies are represented as (bubbles). The regression line (red). The horizontal axis represents classification protocols.

Subgroup analysis under moderator (sample acquisition time) for CD4 T-cell in COVID-19 mortality studies

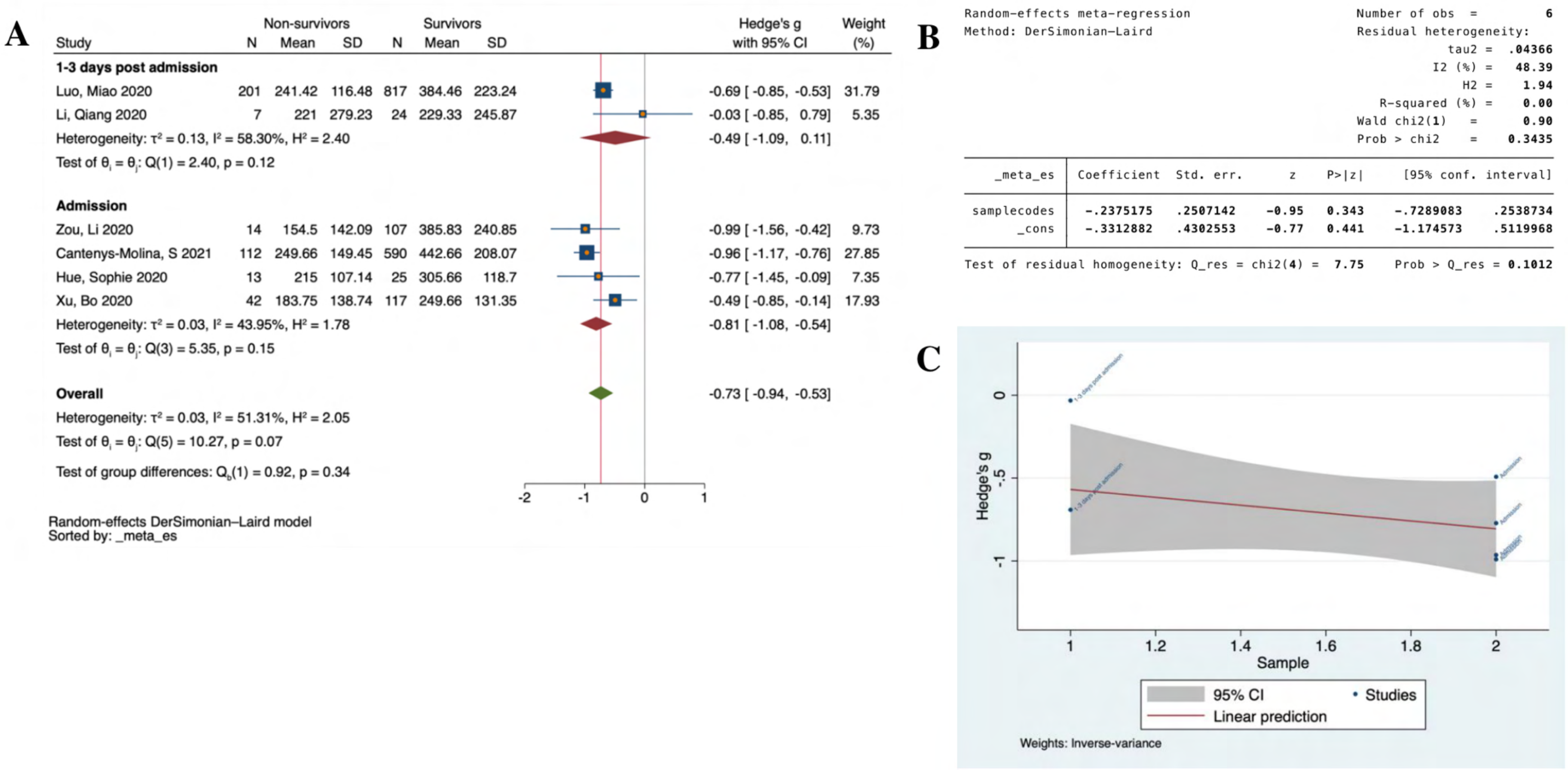

Figure S10.36. Subgroup analysis performed under the moderator (sample acquisition time) for CD4 T-cells in COVID-19 mortality studies. (A) Subgroup forest plot. The no-effect line is represented at the value of zero. The diamond symbol represents estimated combined effect. (B) Subgroup meta-regression. (C) Subgroup meta-regression bubble plot. Studies are represented as (bubbles). The regression line (red). The horizontal axis represents sample acquisition time.

Subgroup analysis under moderator (total male number) for CD4 T-cell in COVID-19 mortality studies

A

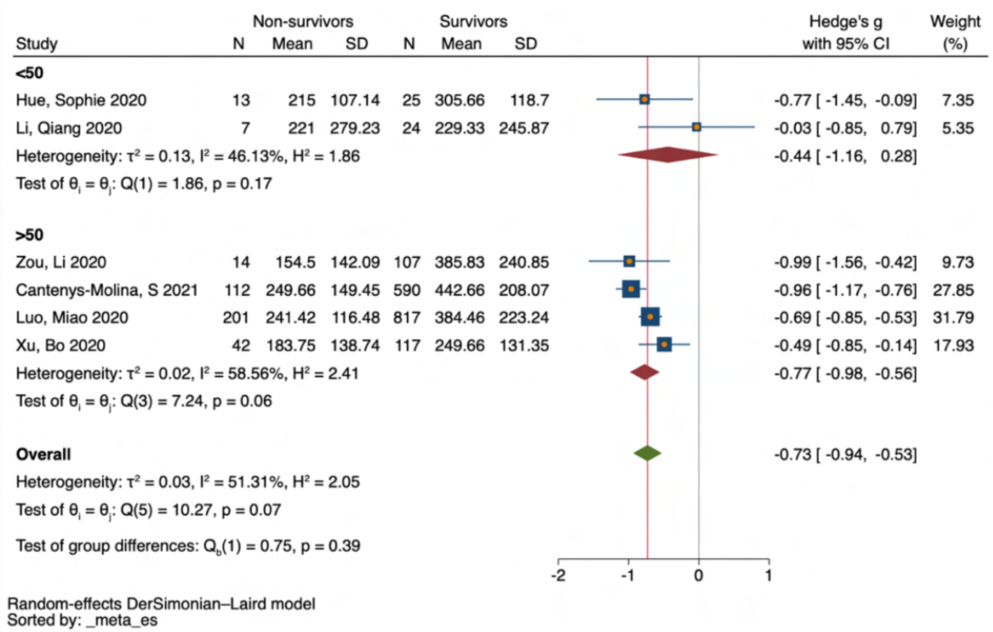

B

```
Random-effects meta-regression                  Number of obs   =          6
Method: DerSimonian–Laird                       Residual heterogeneity:
                                                         tau2 =     .02863
                                                       I2 (%) =      56.02
                                                           H2 =       2.27
                                                  R-squared (%) =       0.00
                                                  Wald chi2(1)  =       0.98
                                                  Prob > chi2   =     0.3219

    _meta_es | Coefficient  Std. err.      z    P>|z|     [95% conf. interval]
-------------+----------------------------------------------------------------
   malecodes |  -.3110161   .3139943    -0.99   0.322    -.9264337    .3044015
       _cons |  -.1489007   .5968917    -0.25   0.803    -1.318787    1.020986
------------------------------------------------------------------------------
Test of residual homogeneity: Q_res = chi2(4) =   9.09     Prob > Q_res = 0.0588
```

C

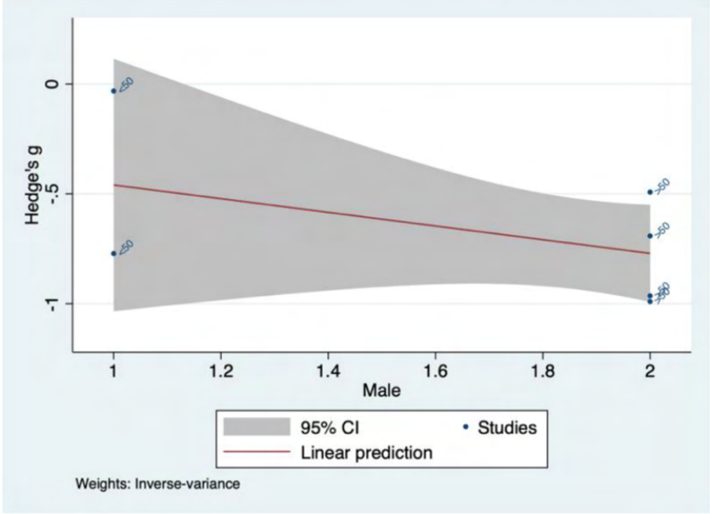

Figure S10.37. Subgroup analysis performed under the moderator (total male number) for CD4 T-cells in COVID-19 mortalitystudies. (A) Subgroup forest plot. The no-effect line is represented at the value of zero. The diamond symbol represents estimated combined effect. (B) Subgroup meta-regression. (C) Subgroup meta-regression bubble plot. Studies are represented as (bubbles). The regression line (red). The horizontal axis represents total male number.

Subgroup analysis under moderator (total female number) for CD4 T-cell in COVID-19 mortality studies

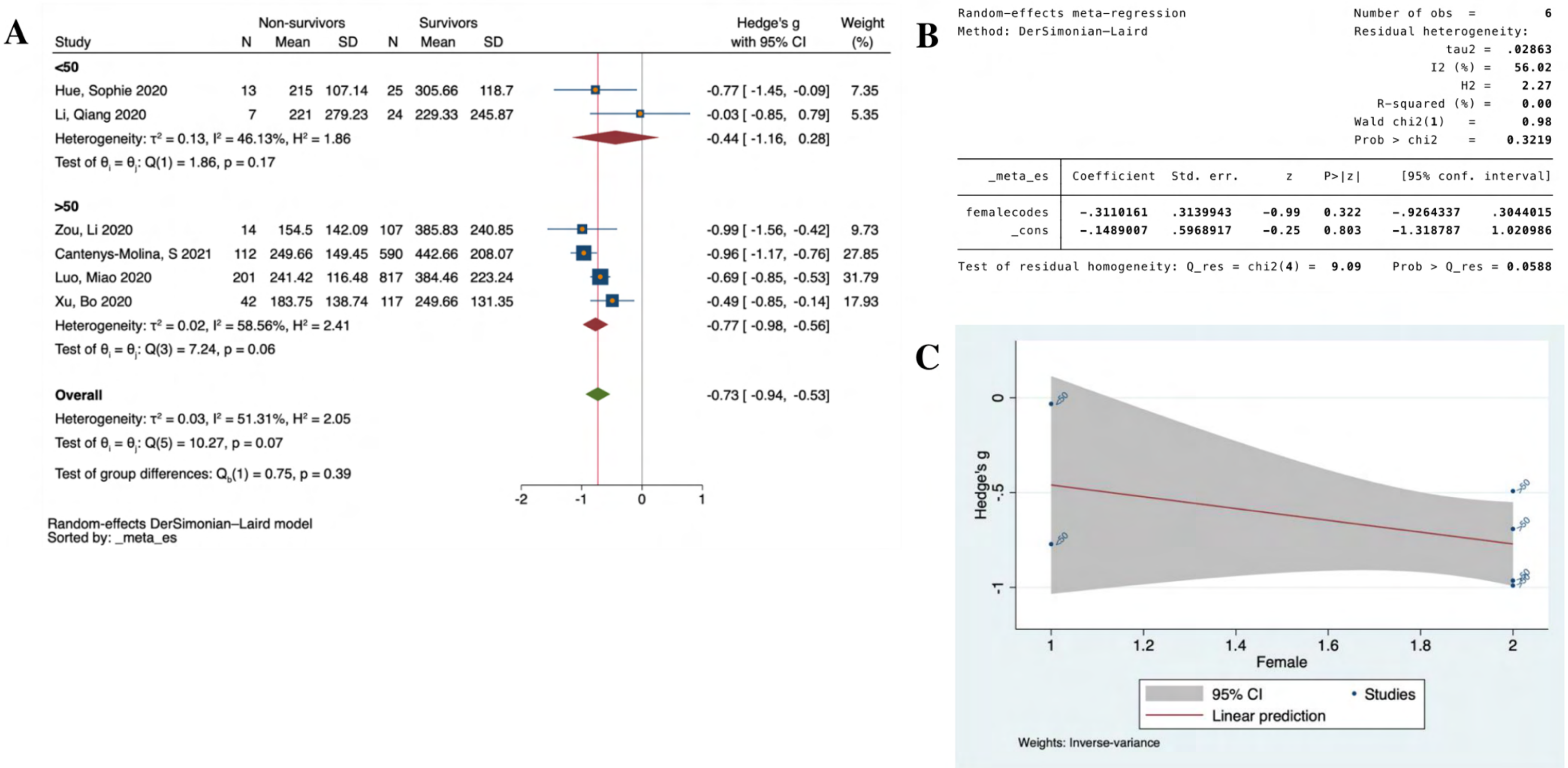

Figure S10.38. Subgroup analysis performed under the moderator (total female number) for CD4 T-cells in COVID-19 mortality studies. (A) Subgroup forest plot. The no-effect line is represented at the value of zero. The diamond symbol represents estimated combined effect. (B) Subgroup meta-regression. (C) Subgroup meta-regression bubble plot. Studies are represented as (bubbles). The regression line (red). The horizontal axis represents total female number.

Subgroup analysis under moderator (mean age) for CD4 T-cell in COVID-19 mortality studies

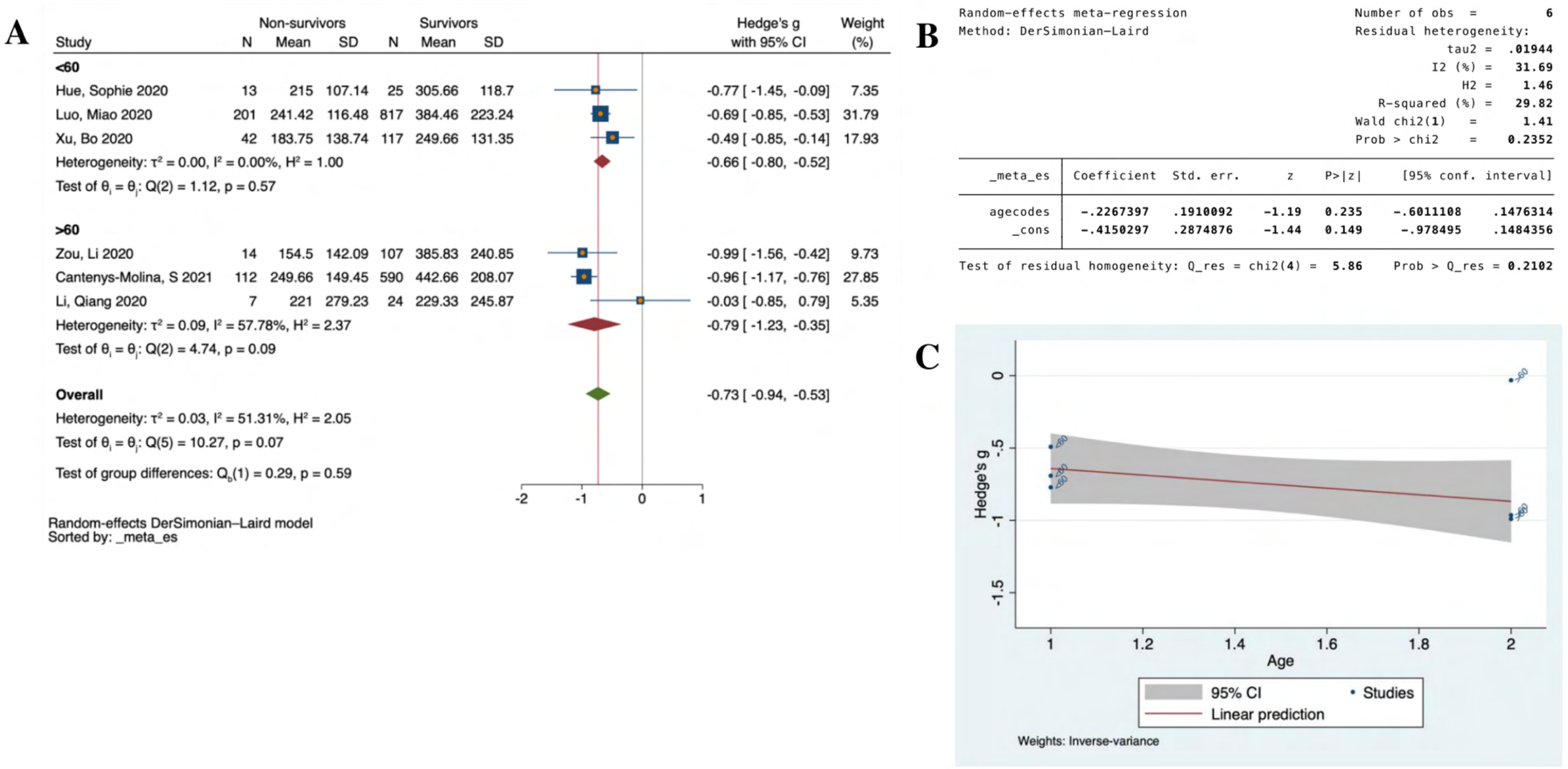

Figure S10.39. Subgroup analysis performed under the moderator (mean age) for CD4 T-cells in COVID-19 mortality studies. (A) Subgroup forest plot. The no-effect line is represented at the value of zero. The diamond symbol represents estimated combined effect. (B) Subgroup meta-regression. (C) Subgroup meta-regression bubble plot. Studies are represented as (bubbles). The regression line (red). The horizontal axis represents mean age.

Subgroup analysis under moderator (test procedure) for CD4 T-cell in COVID-19 mortality studies

---

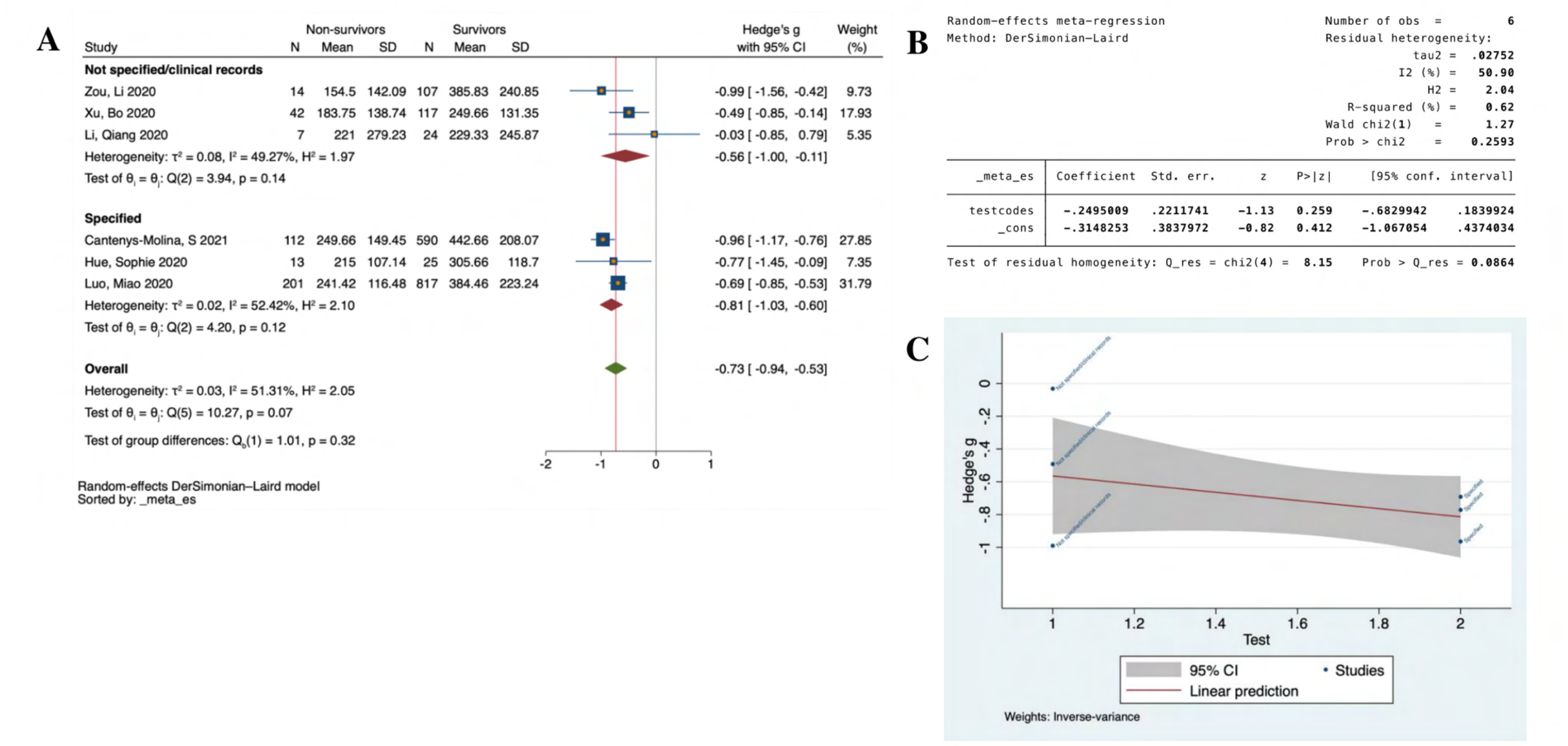

Figure S10.40. Subgroup analysis performed under the moderator (test procedure) for CD4 T-cells in COVID-19 mortality studies. (A) Subgroup forest plot. The no-effect line is represented at the value of zero. The diamond symbol represents estimated combined effect. (B) Subgroup meta-regression. (C) Subgroup meta-regression bubble plot. Studies are represented as (bubbles). The regression line (red). The horizontal axis represents test procedure.

Subgroup analysis under moderator (city) for CD8 T-cell in COVID-19 mortality studies

---

A

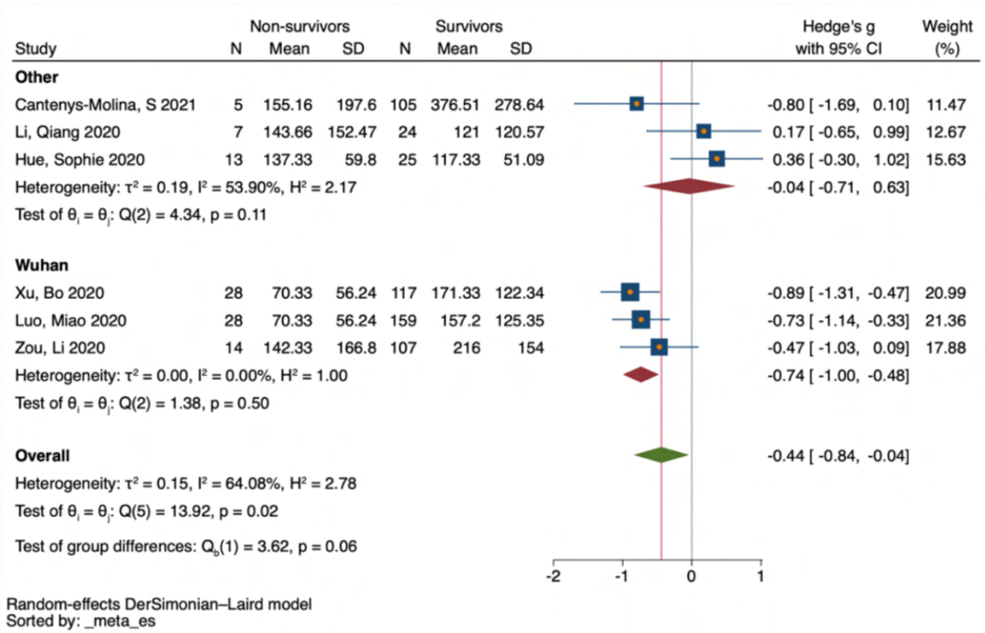

B

```
Random-effects meta-regression                  Number of obs  =          6
Method: DerSimonian–Laird                       Residual heterogeneity:
                                                          tau2 =    .03493
                                                        I2 (%) =     30.07
                                                            H2 =      1.43
                                                 R-squared (%) =     76.70
                                                 Wald chi2(1)  =      5.57
                                                 Prob > chi2   =    0.0183

    _meta_es | Coefficient  Std. err.      z    P>|z|     [95% conf. interval]
-------------+----------------------------------------------------------------
   citycodes |  -.7231451    .306384   -2.36   0.018    -1.323647   -.1226434
       _cons |   .7227862   .5353288    1.35   0.177    -.3264391    1.772011

Test of residual homogeneity: Q_res = chi2(4) =  5.72     Prob > Q_res = 0.2210
```

C

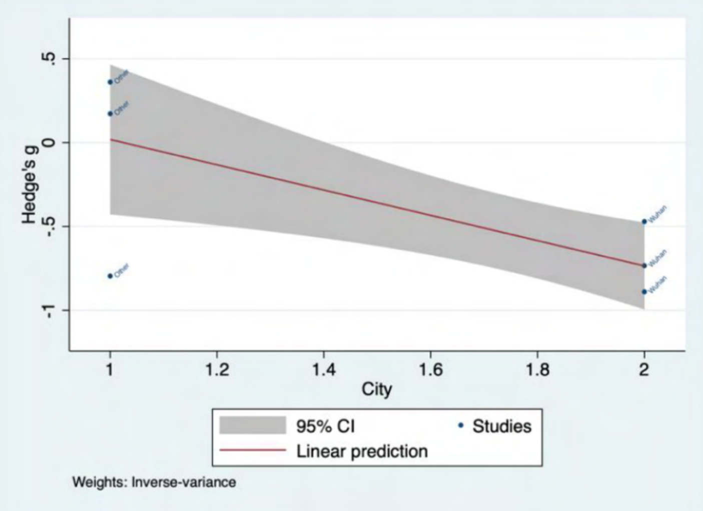

Figure S10.41. Subgroup analysis performed under the moderator (city) for CD8 T-cells in COVID-19 mortality studies. (A) Subgroup forest plot. The no-effect line is represented at the value of zero. The diamond symbol represents estimated combined effect. (B) Subgroup meta-regression. (C) Subgroup meta-regression bubble plot. Studies are represented as (bubbles). The regression line (red). The horizontal axis represents cities.

Subgroup analysis under moderator (country) for CD8 T-cell in COVID-19 mortality studies

A

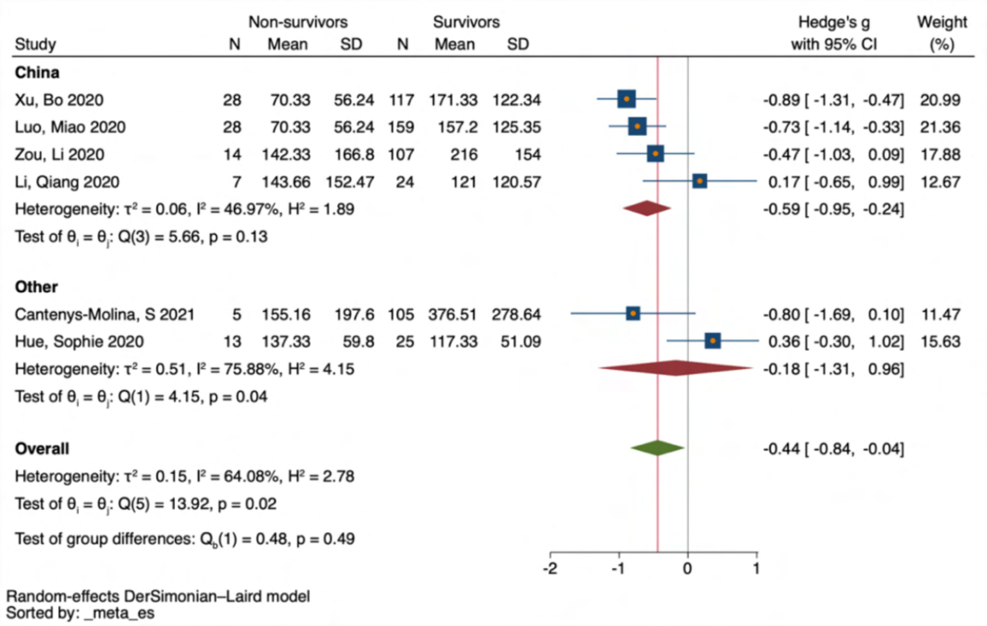

B

```
Random-effects meta-regression                     Number of obs  =          6
Method: DerSimonian-Laird                          Residual heterogeneity:
                                                              tau2 =     .1159
                                                            I2 (%) =     59.20
                                                                H2 =      2.45
                                                     R-squared (%) =     22.68
                                                   Wald chi2(1)    =      1.11
                                                   Prob > chi2     =    0.2917

------------------------------------------------------------------------------
    _meta_es | Coefficient  Std. err.      z    P>|z|     [95% conf. interval]
-------------+----------------------------------------------------------------
countrycodes |   .4502159   .4270263     1.05   0.292    -.3867403    1.287172
       _cons |   -1.01793   .5704878    -1.78   0.074    -2.136066    .1002055
------------------------------------------------------------------------------
Test of residual homogeneity: Q_res = chi2(4) = 9.80      Prob > Q_res = 0.0439
```

C

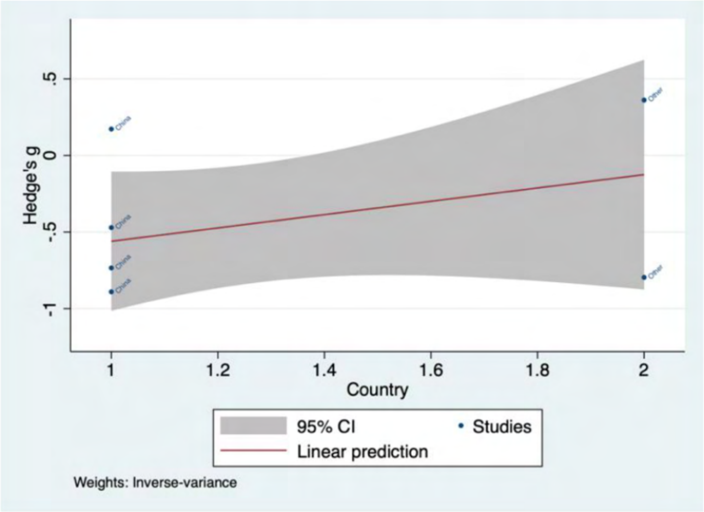

Figure S10.42. Subgroup analysis performed under the moderator (country) for CD8 T-cells in COVID-19 mortality studies. (A) Subgroup forest plot. The no-effect line is represented at the value of zero. The diamond symbol represents estimated combined effect. (B) Subgroup meta-regression. (C) Subgroup meta-regression bubble plot. Studies are represented as (bubbles). The regression line (red). The horizontal axis represents countries.

Subgroup analysis under moderator (continent) for CD8 T-cell in COVID-19 mortality studies

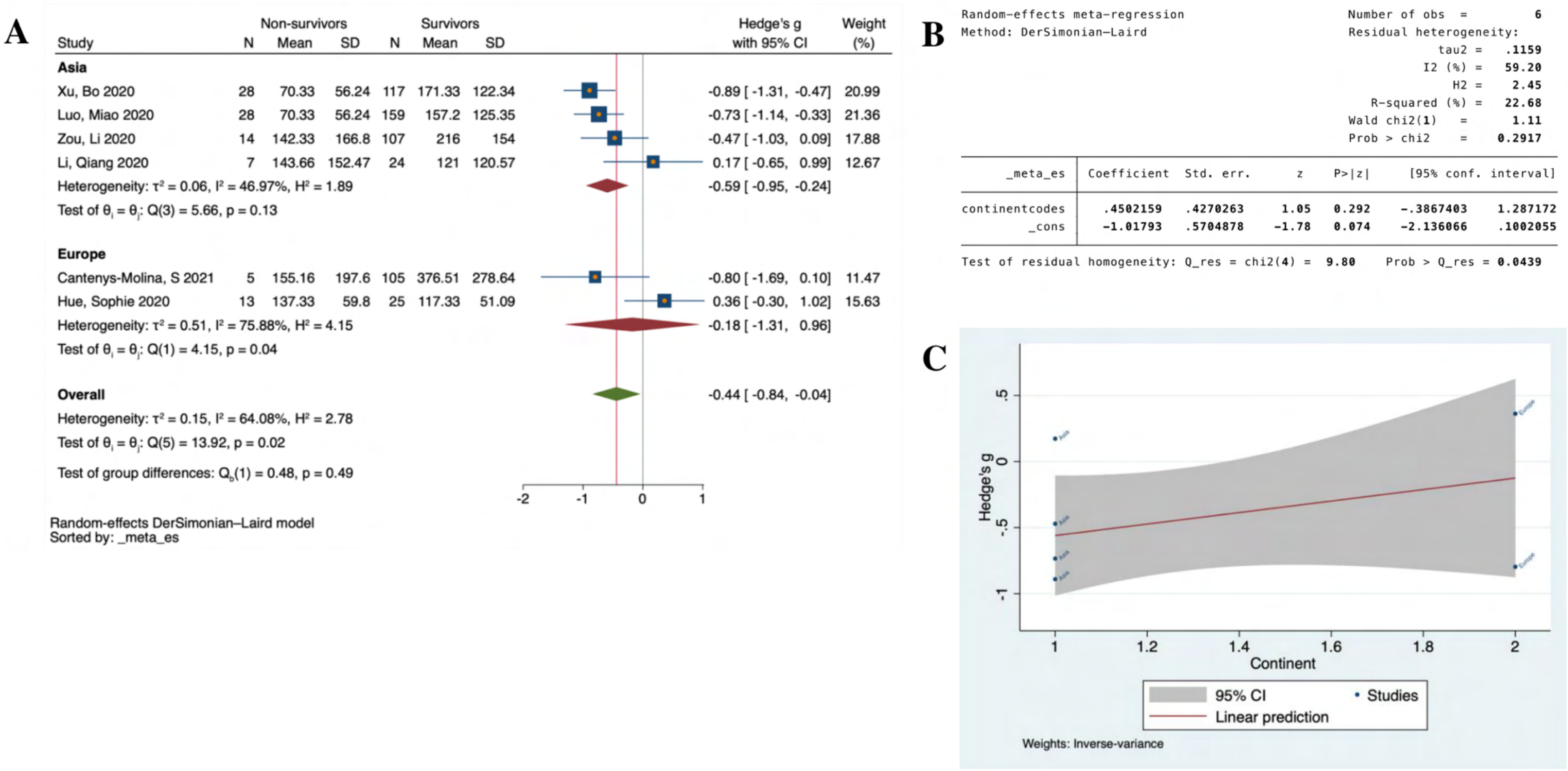

Figure S10.43. Subgroup analysis performed under the moderator (continent) for CD8 T-cells in COVID-19 mortality studies. (A) Subgroup forest plot. The no-effect line is represented at the value of zero. The diamond symbol represents estimated combined effect. (B) Subgroup meta-regression. (C) Subgroup meta-regression bubble plot. Studies are represented as (bubbles). The regression line (red). The horizontal axis represents continents.

Subgroup analysis under moderator (study design) for CD8 T-cell in COVID-19 mortality studies

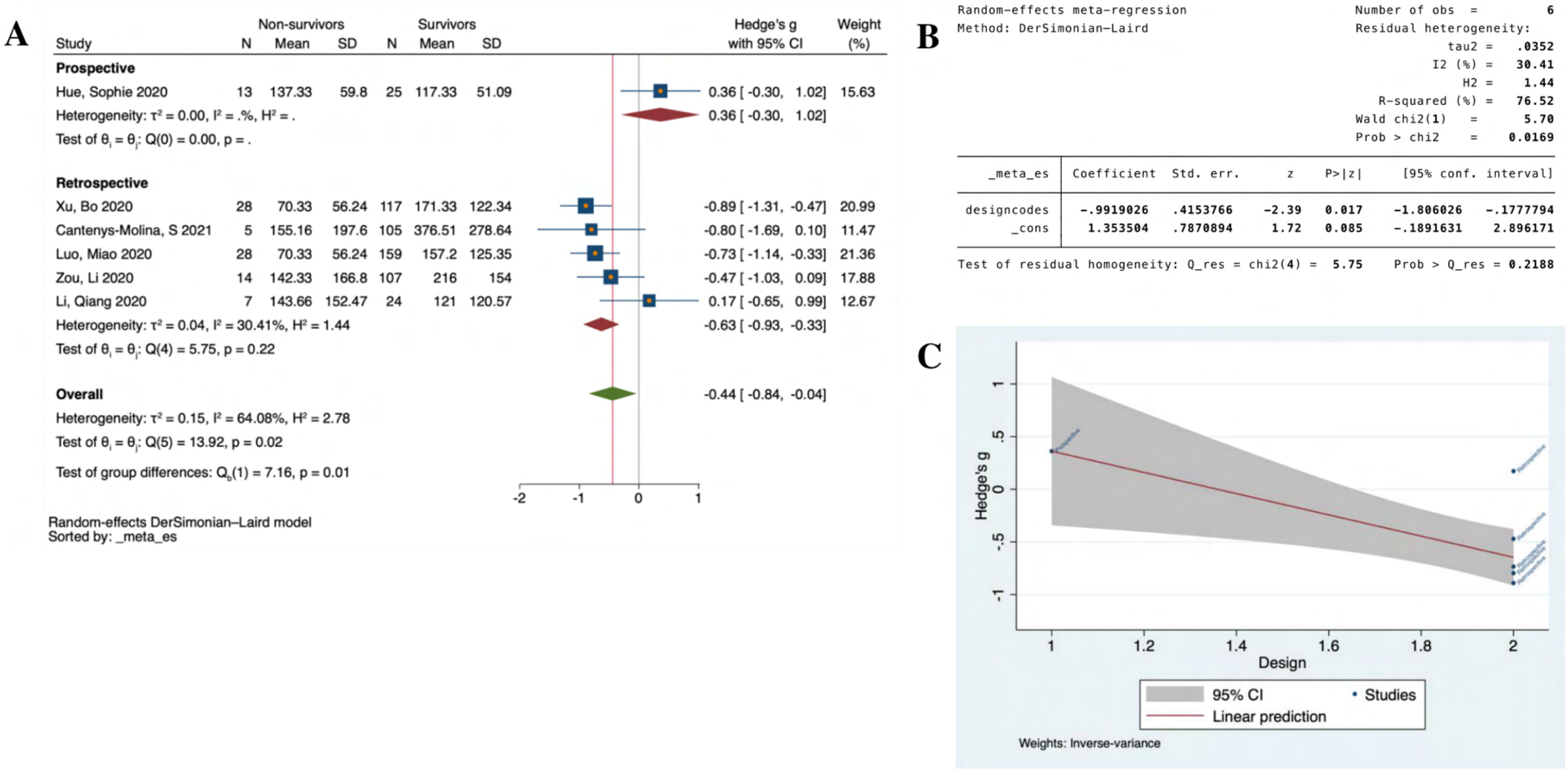

Figure S10.44. Subgroup analysis performed under the moderator (study design) for CD8 T-cells in COVID-19 mortality studies. (A) Subgroup forest plot. The no-effect line is represented at the value of zero. The diamond symbol represents estimated combined effect. (B) Subgroup meta-regression. (C) Subgroup meta-regression bubble plot. Studies are represented as (bubbles). The regression line (red). The horizontal axis represents study design.

Subgroup analysis under moderator (classification protocol) for CD8 T-cell in COVID-19 mortality studies

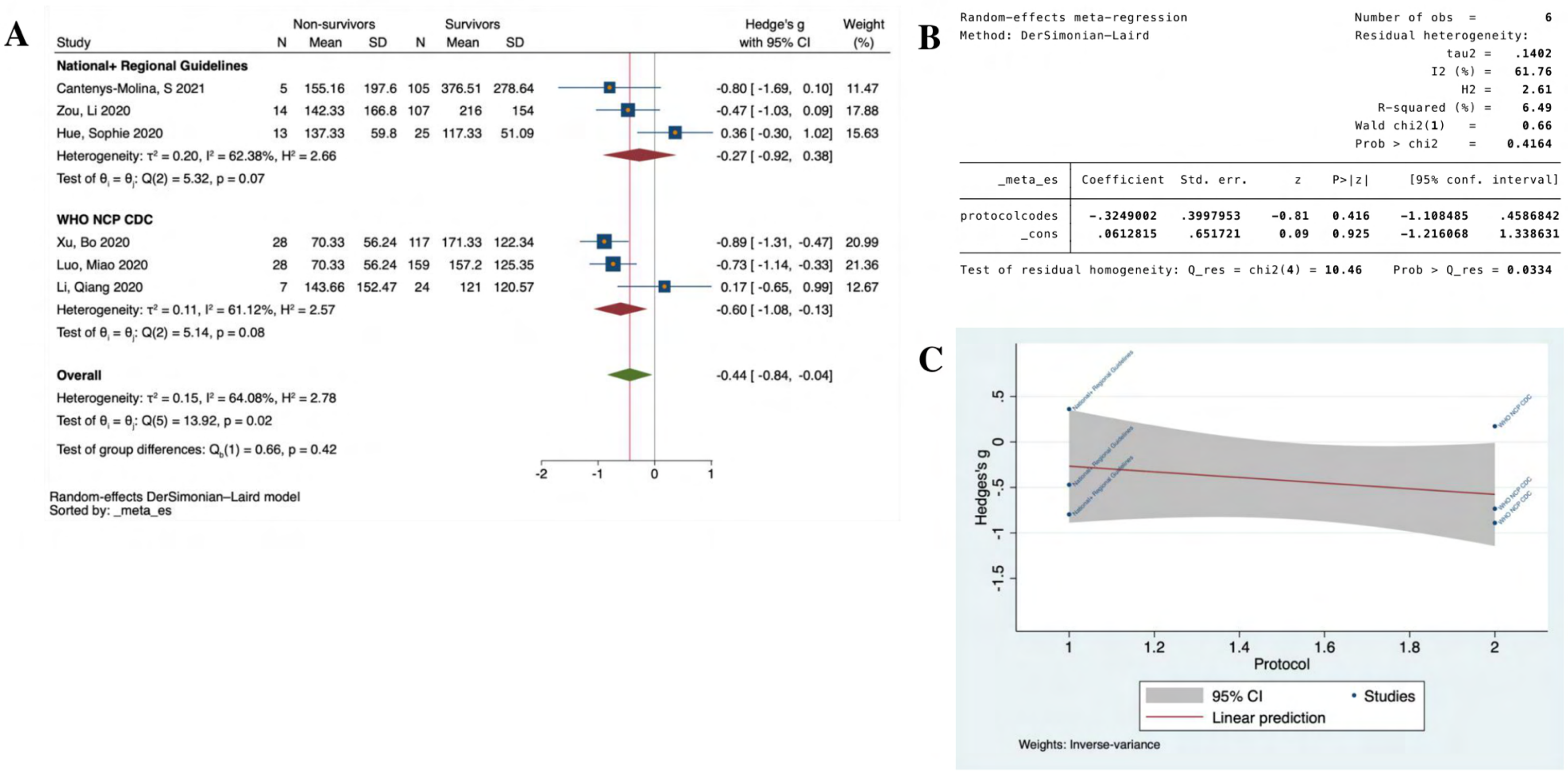

Figure S10.45. Subgroup analysis performed under the moderator (classification protocol) for CD8 T-cells in COVID-19 mortality studies. (A) Subgroup forest plot. The no-effect line is represented at the value of zero. The diamond symbol represents estimated combined effect. (B) Subgroup meta-regression. (C) Subgroup meta-regression bubble plot. Studies are represented as (bubbles). The regression line (red). The horizontal axis represents classification protocols.

Subgroup analysis under moderator (sample acquisition time) for CD8 T-cell in COVID-19 mortality studies

A

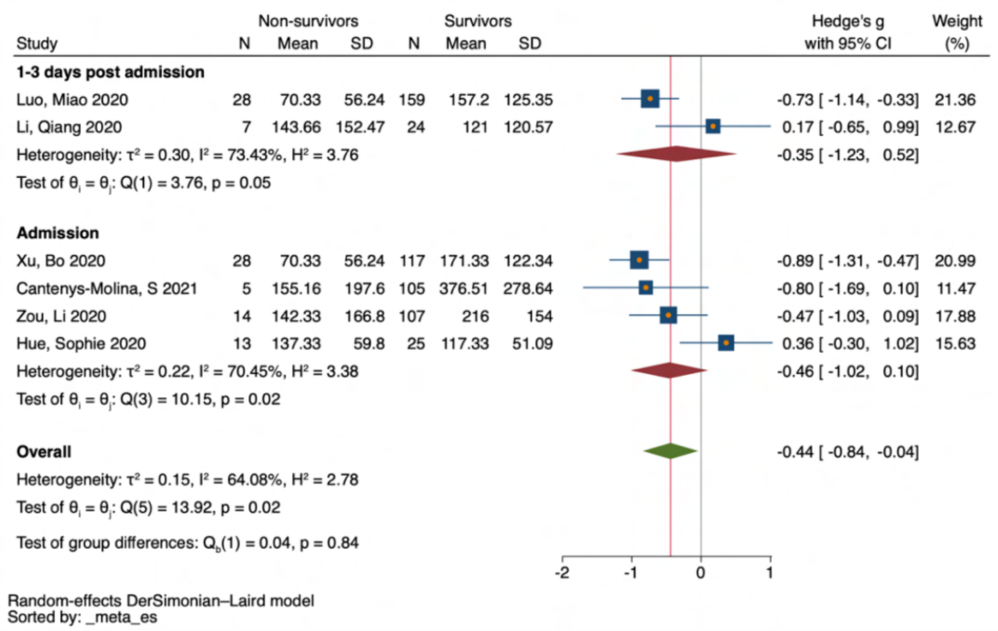

B

```
Random-effects meta-regression                    Number of obs  =          6
Method: DerSimonian–Laird                         Residual heterogeneity:
                                                              tau2 =    .2388
                                                            I2 (%) =    71.25
                                                                H2 =     3.48
                                                    R-squared (%) =     0.00
                                                    Wald chi2(1)  =     0.03
                                                    Prob > chi2   =   0.8586
```

_meta_es	Coefficient	Std. err.	z	P>|z|	[95% conf. interval]	
samplecodes	-.0896445	.5032133	-0.18	0.859	-1.075924	.8966354
_cons	-.277434	.8697774	-0.32	0.750	-1.982166	1.427298

Test of residual homogeneity: Q_res = chi2(4) = 13.91   Prob > Q_res = 0.0076

C

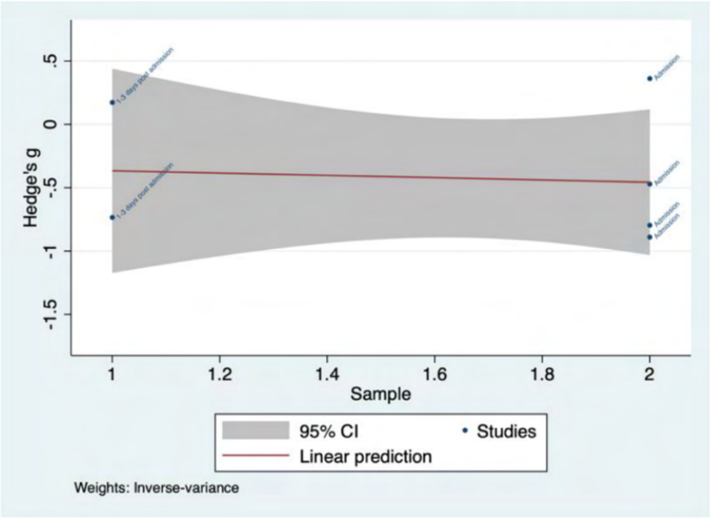

Figure S10.46. Subgroup analysis performed under the moderator (sample acquisition time) for CD8 T-cells in COVID-19 mortality studies. (A) Subgroup forest plot. The no-effect line is represented at the value of zero. The diamond symbol represents estimated combined effect. (B) Subgroup meta-regression. (C) Subgroup meta-regression bubble plot. Studies are represented as (bubbles). The regression line (red). The horizontal axis represents sample acquisition time.

Subgroup analysis under moderator (total male number) for CD8 T-cell in COVID-19 mortality studies

A

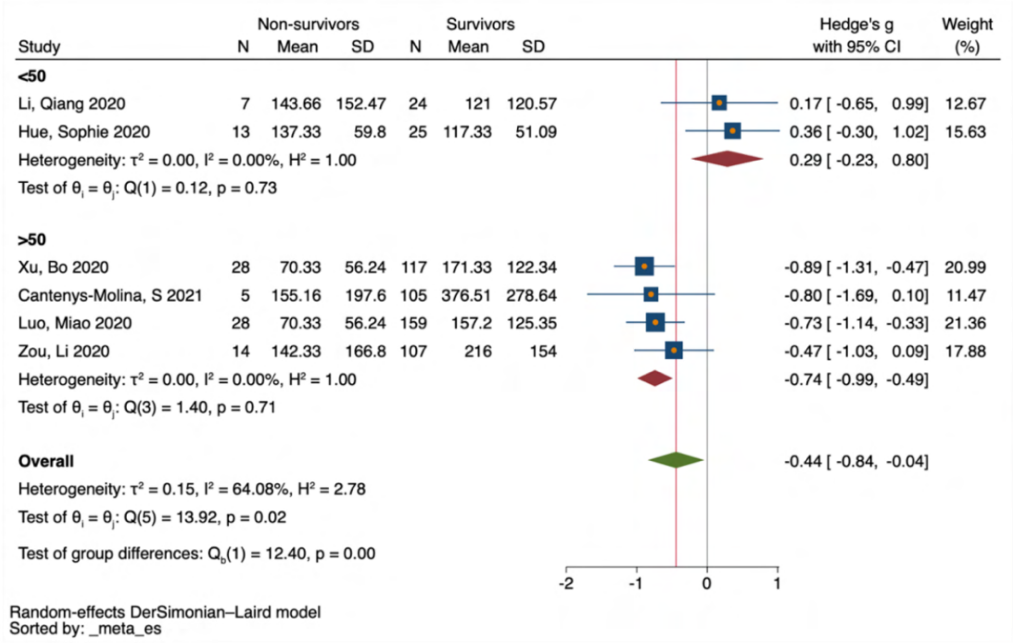

B

```
Random-effects meta-regression                 Number of obs    =        6
Method: DerSimonian-Laird                      Residual heterogeneity:
                                                          tau2 =        0
                                                        I2 (%) =     0.00
                                                            H2 =     1.00
                                                   R-squared (%) =   100.00
                                               Wald chi2(1)     =    12.40
                                               Prob > chi2      =   0.0004
```

_meta_es	Coefficient	Std. err.	z	P>|z|	[95% conf.	interval]
malecodes	-1.02775	.2918858	-3.52	0.000	-1.599835	-.455664
_cons	1.315029	.5406408	2.43	0.015	.2553928	2.374666

Test of residual homogeneity: Q_res = chi2(4) = 1.52 Prob > Q_res = 0.8228

C

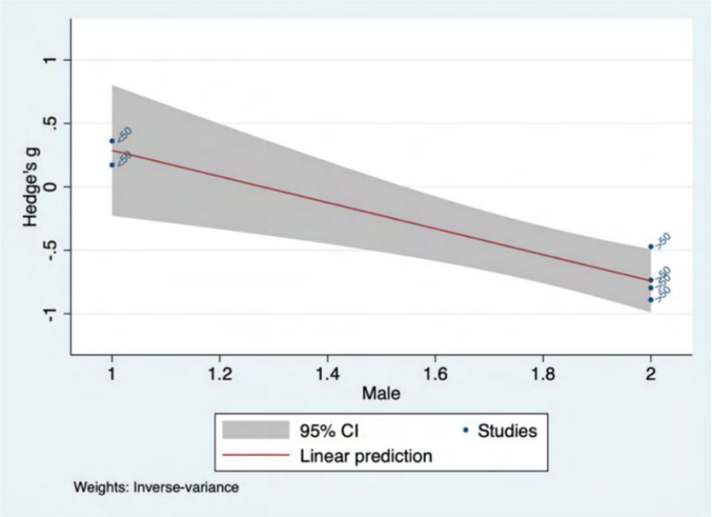

Figure S10.47. Subgroup analysis performed under the moderator (total male number) for CD8 T-cells in COVID-19 mortality studies. (A) Subgroup forest plot. The no-effect line is represented at the value of zero. The diamond symbol represents estimated combined effect. (B) Subgroup meta-regression. (C) Subgroup meta-regression bubble plot. Studies are represented as (bubbles). The regression line (red). The horizontal axis represents total male number.

Subgroup analysis under moderator (total female number) for CD8 T-cell in COVID-19 mortality studies

A

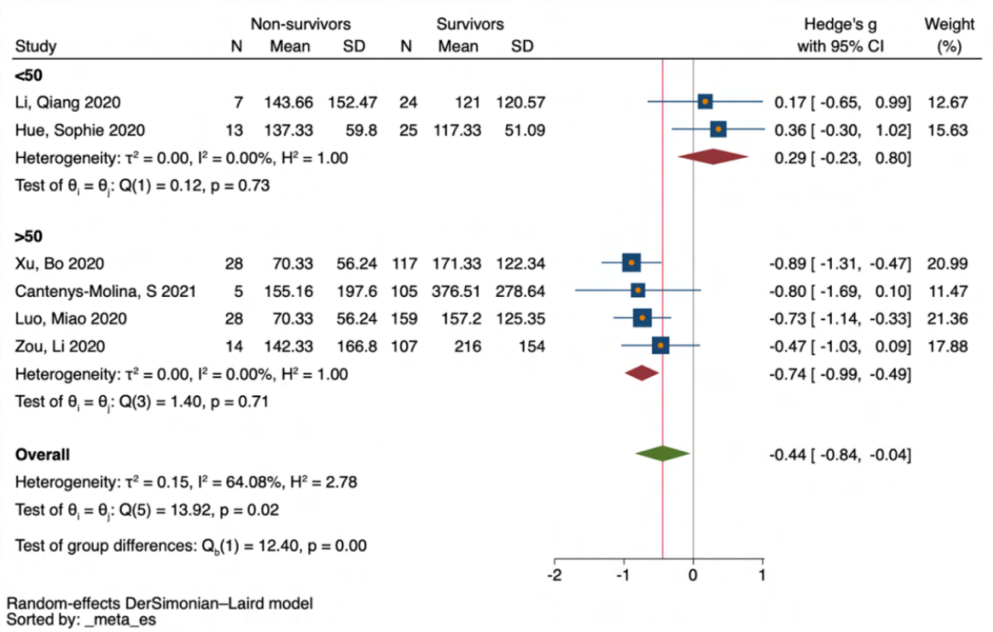

B

```
Random-effects meta-regression                  Number of obs  =          6
Method: DerSimonian-Laird                       Residual heterogeneity:
                                                             tau2 =       0
                                                           I2 (%) =    0.00
                                                               H2 =    1.00
                                                   R-squared (%) =  100.00
                                                Wald chi2(1)     =   12.40
                                                Prob > chi2      =  0.0004

    _meta_es | Coefficient  Std. err.      z    P>|z|     [95% conf. interval]
-------------+----------------------------------------------------------------
 femalecodes |   -1.02775   .2918858    -3.52   0.000    -1.599835   -.455664
       _cons |   1.315029   .5406408     2.43   0.015     .2553928   2.374666

Test of residual homogeneity: Q_res = chi2(4) =  1.52     Prob > Q_res = 0.8228
```

C

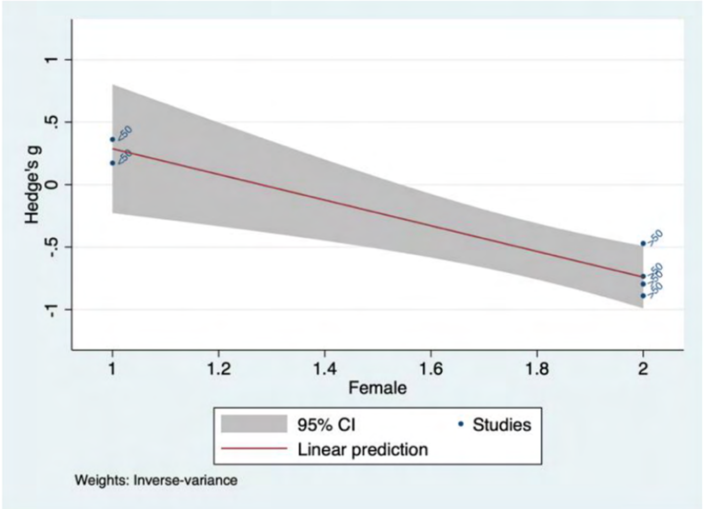

Figure S10.48. Subgroup analysis performed under the moderator (total female number) for CD8 T-cells in COVID-19 mortality studies. (A) Subgroup forest plot. The no-effect line is represented at the value of zero. The diamond symbol represents estimated combined effect. (B) Subgroup meta-regression. (C) Subgroup meta-regression bubble plot. Studies are represented as (bubbles). The regression line (red). The horizontal axis represents total female number.

Subgroup analysis under moderator (mean age) for CD8 T-cell in COVID-19 mortality studies

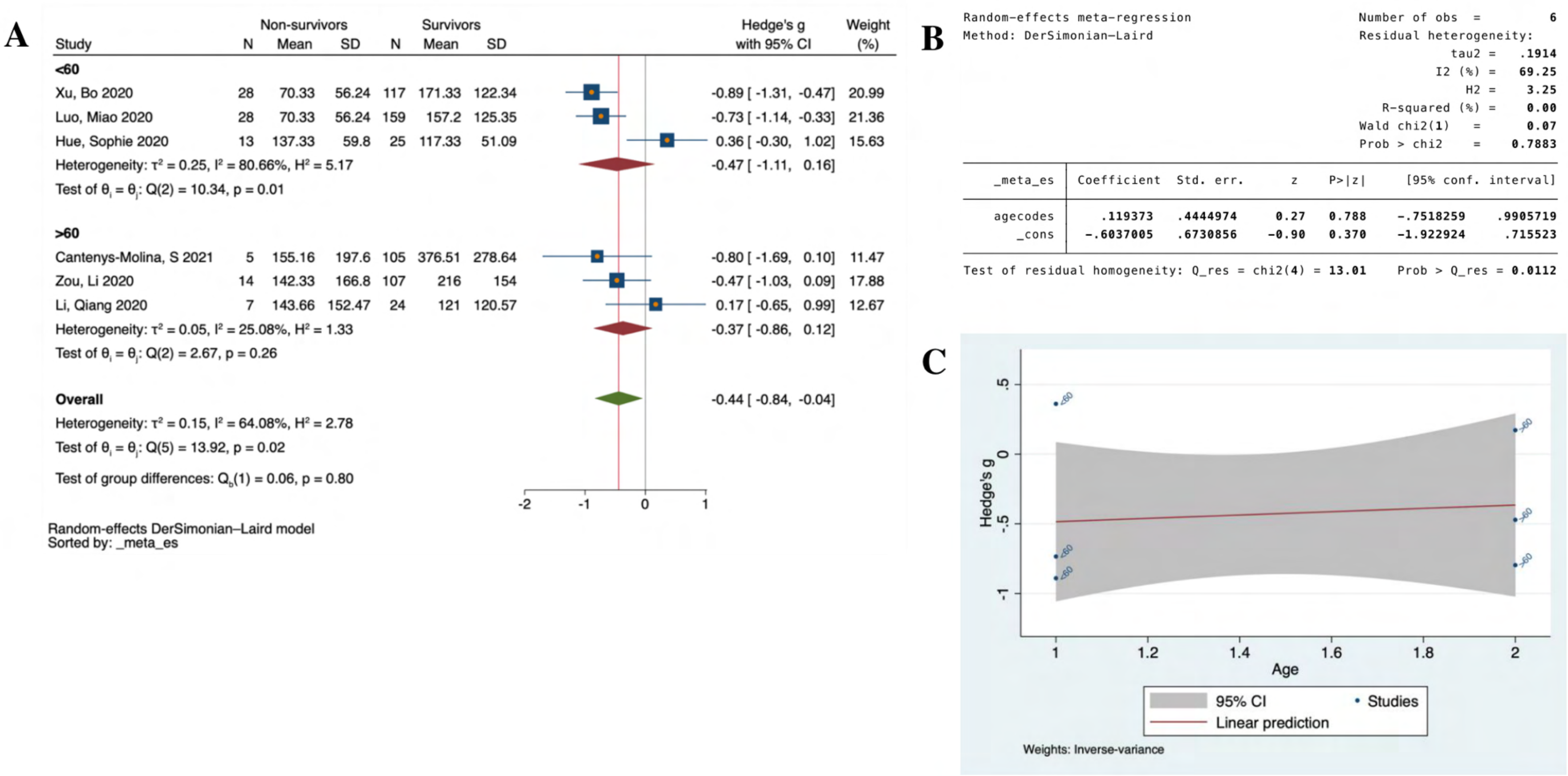

Figure S10.49. Subgroup analysis performed under the moderator (mean age) for CD8 T-cells in COVID-19 mortality studies. (A) Subgroup forest plot. The no-effect line is represented at the value of zero. The diamond symbol represents estimated combined effect. (B) Subgroup meta-regression. (C) Subgroup meta-regression bubble plot. Studies are represented as (bubbles). The regression line (red). The horizontal axis represents mean age.

Subgroup analysis under moderator (test procedure) for CD8 T-cell in COVID-19 mortality studies

A

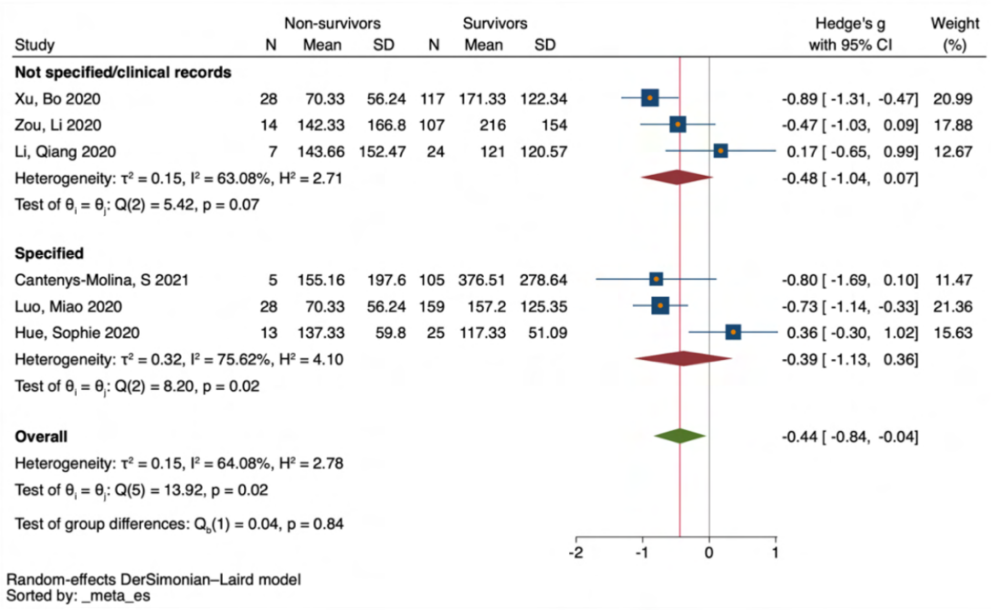

B

```
Random-effects meta-regression                  Number of obs  =          6
Method: DerSimonian-Laird                       Residual heterogeneity:
                                                          tau2 =      .2257
                                                        I2 (%) =      70.63
                                                            H2 =       3.40
                                                  R-squared (%) =       0.00
                                                  Wald chi2(1)  =       0.03
                                                  Prob > chi2   =     0.8738
```

_meta_es	Coefficient	Std. err.	z	P>|z|	[95% conf. interval]	
testcodes	.0740759	.466501	0.16	0.874	-.8402492	.988401
_cons	-.538205	.7319719	-0.74	0.462	-1.972844	.8964336

Test of residual homogeneity: Q_res = chi2(4) = 13.62   Prob > Q_res = 0.0086

C

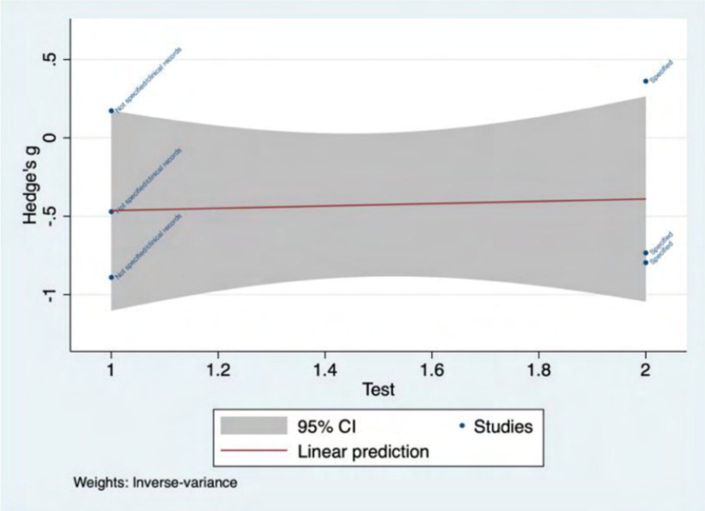

Figure S10.50. Subgroup analysis performed under the moderator (test procedure) for CD8 T-cells in COVID-19 mortality studies. (A) Subgroup forest plot. The no-effect line is represented at the value of zero. The diamond symbol represents estimated combined effect. (B) Subgroup meta-regression. (C) Subgroup meta-regression bubble plot. Studies are represented as (bubbles). The regression line (red). The horizontal axis represents test procedure.

Subgroup analysis under moderator (city) for IL-10 in COVID-19 mortality studies

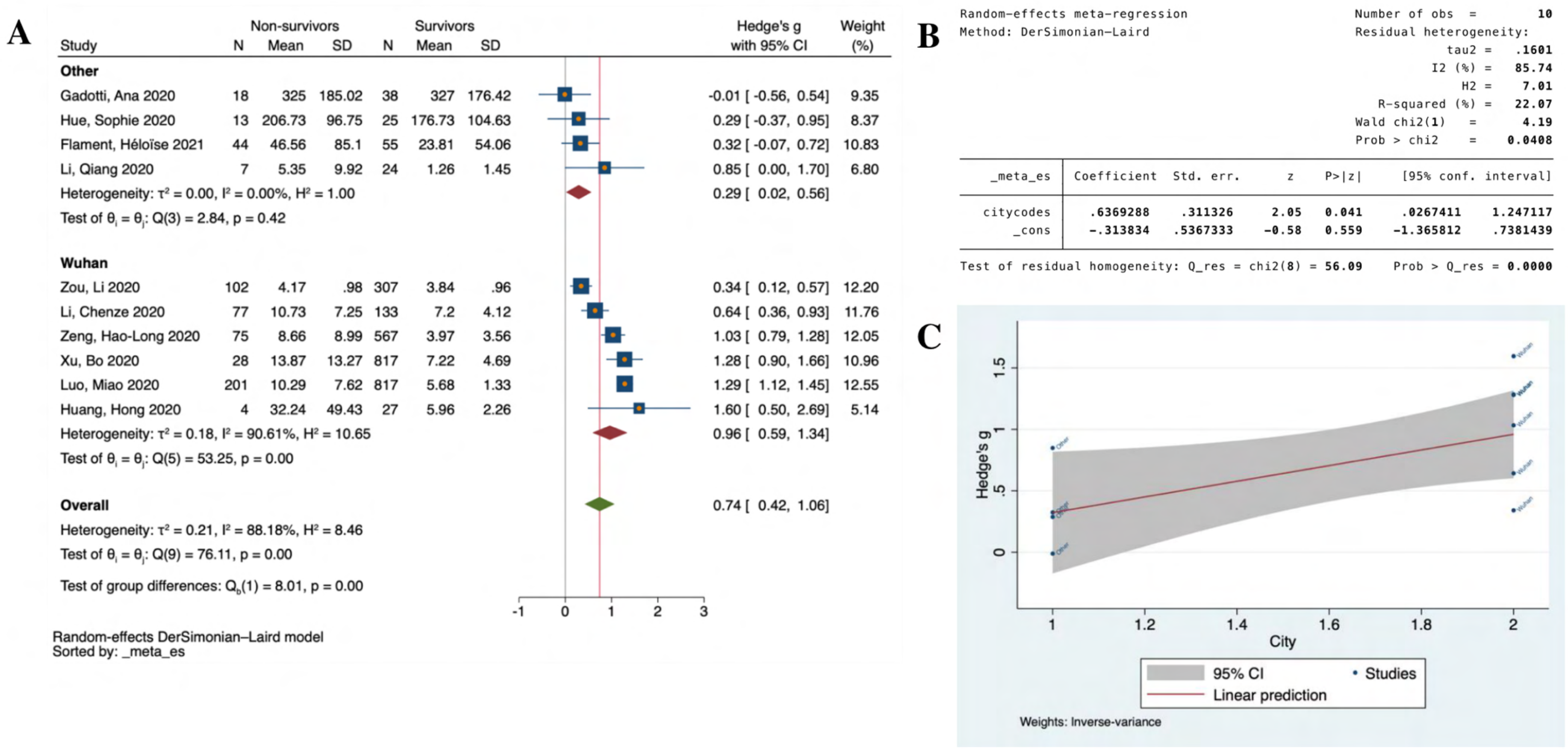

Figure S10.51. Subgroup analysis performed under the moderator (city) for IL-10 in COVID-19 mortality studies. (A) Subgroup forest plot. The no-effect line is represented at the value of zero. The diamond symbol represents estimated combined effect. (B) Subgroup meta-regression. (C) Subgroup meta-regression bubble plot. Studies are represented as (bubbles). The regression line (red). The horizontal axis represents cities.

Subgroup analysis under moderator (country) for IL-10 in COVID-19 mortality studies

---

A

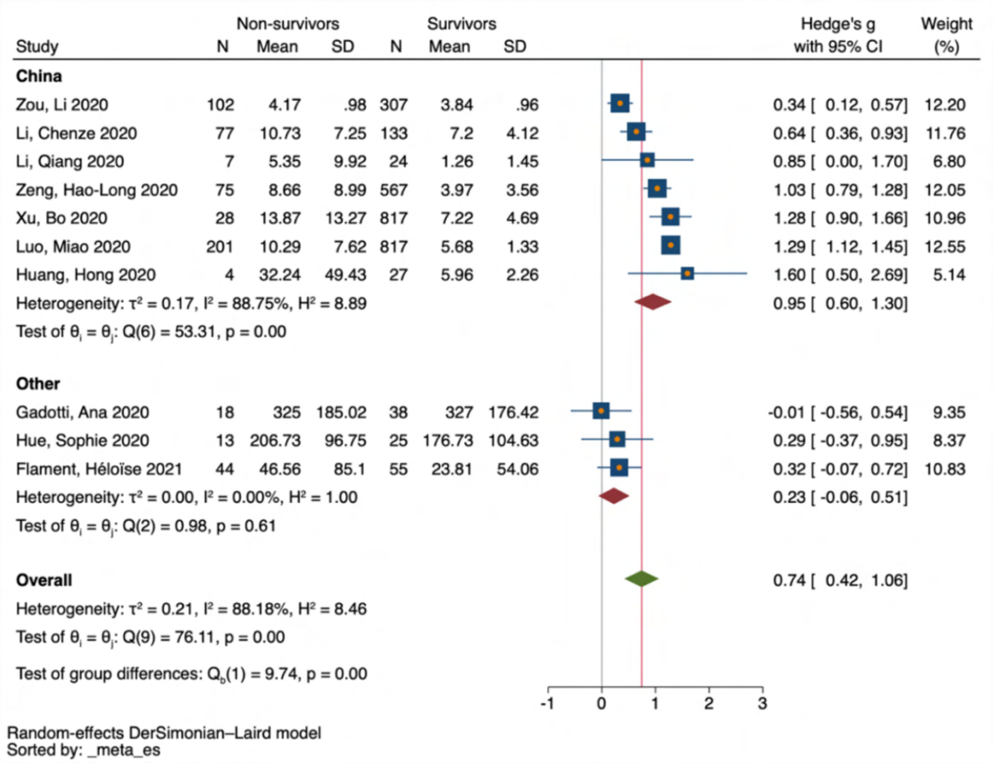

B

```
Random-effects meta-regression                  Number of obs  =        10
Method: DerSimonian–Laird                       Residual heterogeneity:
                                                          tau2 =     .1541
                                                        I2 (%) =     85.26
                                                            H2 =      6.79
                                                 R-squared (%) =     24.97
                                                Wald chi2(1)   =      5.26
                                                Prob > chi2    =    0.0218

    _meta_es | Coefficient  Std. err.      z    P>|z|     [95% conf. interval]
-------------+----------------------------------------------------------------
countrycodes |  -.7445618   .3246837    -2.29   0.022    -1.38093   -.1081933
       _cons |   1.694029   .4399227     3.85   0.000    .8317963    2.556262

Test of residual homogeneity: Q_res = chi2(8) = 54.29    Prob > Q_res = 0.0000
```

C

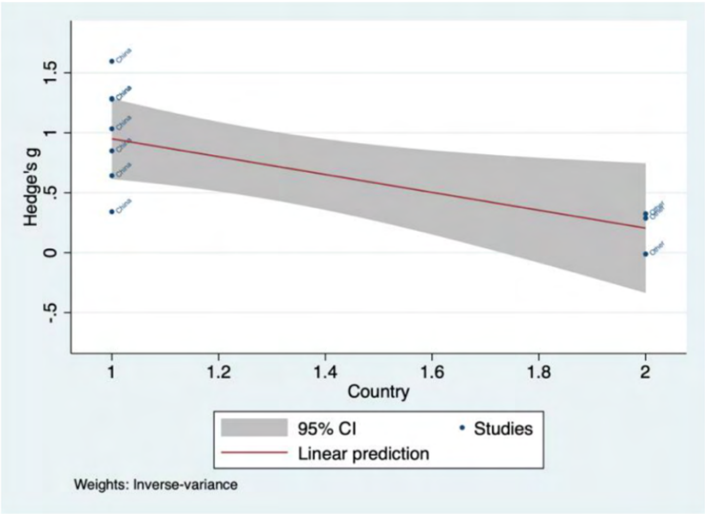

Figure S10.52. Subgroup analysis performed under the moderator (country) for IL-10 in COVID-19 mortality studies. (A) Subgroup forest plot. The no-effect line is represented at the value of zero. The diamond symbol represents estimated combined effect. (B) Subgroup meta-regression. (C) Subgroup meta-regression bubble plot. Studies are represented as (bubbles). The regression line (red). The horizontal axis represents countries.

Subgroup analysis under moderator (continent) for IL-10 in COVID-19 mortality studies

A

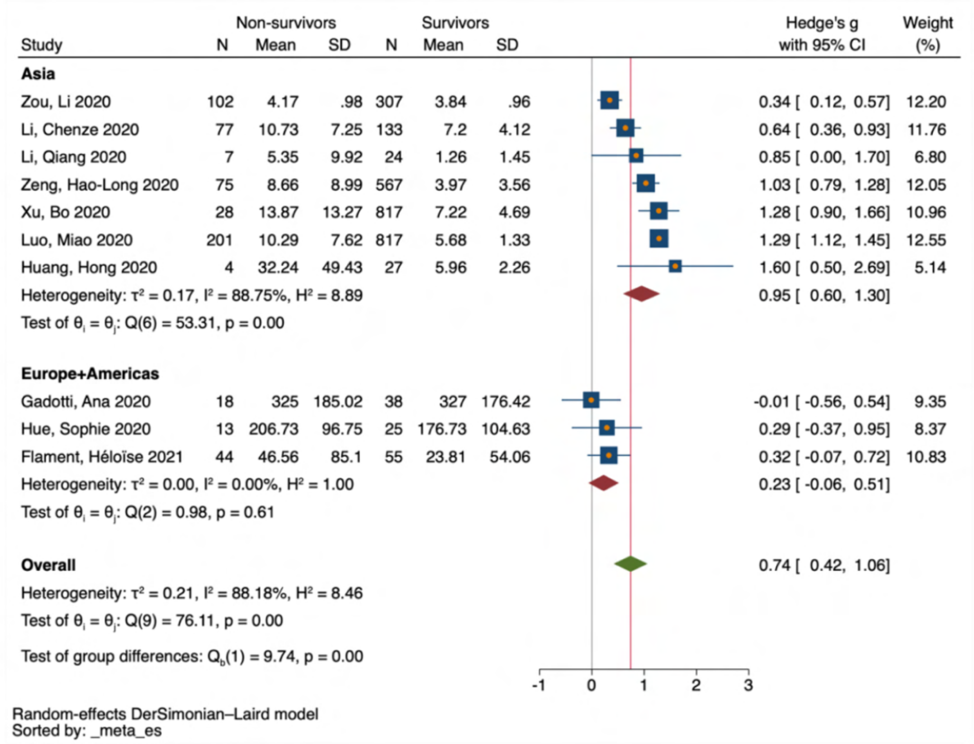

B

```
Random-effects meta-regression                  Number of obs  =        10
Method: DerSimonian–Laird                       Residual heterogeneity:
                                                        tau2 =     .1541
                                                      I2 (%) =     85.26
                                                          H2 =      6.79
                                                 R-squared (%) =     24.97
                                                 Wald chi2(1)  =      5.26
                                                 Prob > chi2   =    0.0218

-----------------------------------------------------------------------------
    _meta_es | Coefficient  Std. err.      z    P>|z|     [95% conf. interval]
-------------+---------------------------------------------------------------
continentcodes |  -.7445618  .3246837    -2.29   0.022    -1.38093   -.1081933
       _cons |   1.694029   .4399227     3.85   0.000    .8317963    2.556262
-----------------------------------------------------------------------------
Test of residual homogeneity: Q_res = chi2(8) = 54.29    Prob > Q_res = 0.0000
```

C

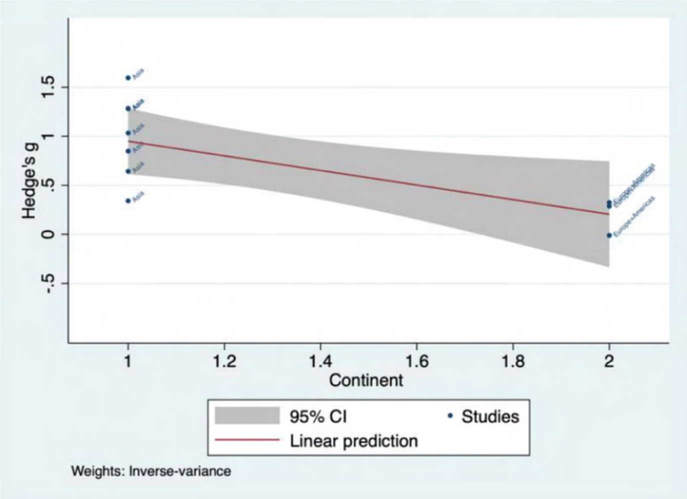

Figure S10.53. Subgroup analysis performed under the moderator (continent) for IL-10 in COVID-19 mortality studies. (A) Subgroup forest plot. The no-effect line is represented at the value of zero. The diamond symbol represents estimated combined effect. (B) Subgroup meta-regression. (C) Subgroup meta-regression bubble plot. Studies are represented as (bubbles). The regression line (red). The horizontal axis represents continents.

Subgroup analysis under moderator (study design) for IL-10 in COVID-19 mortality studies

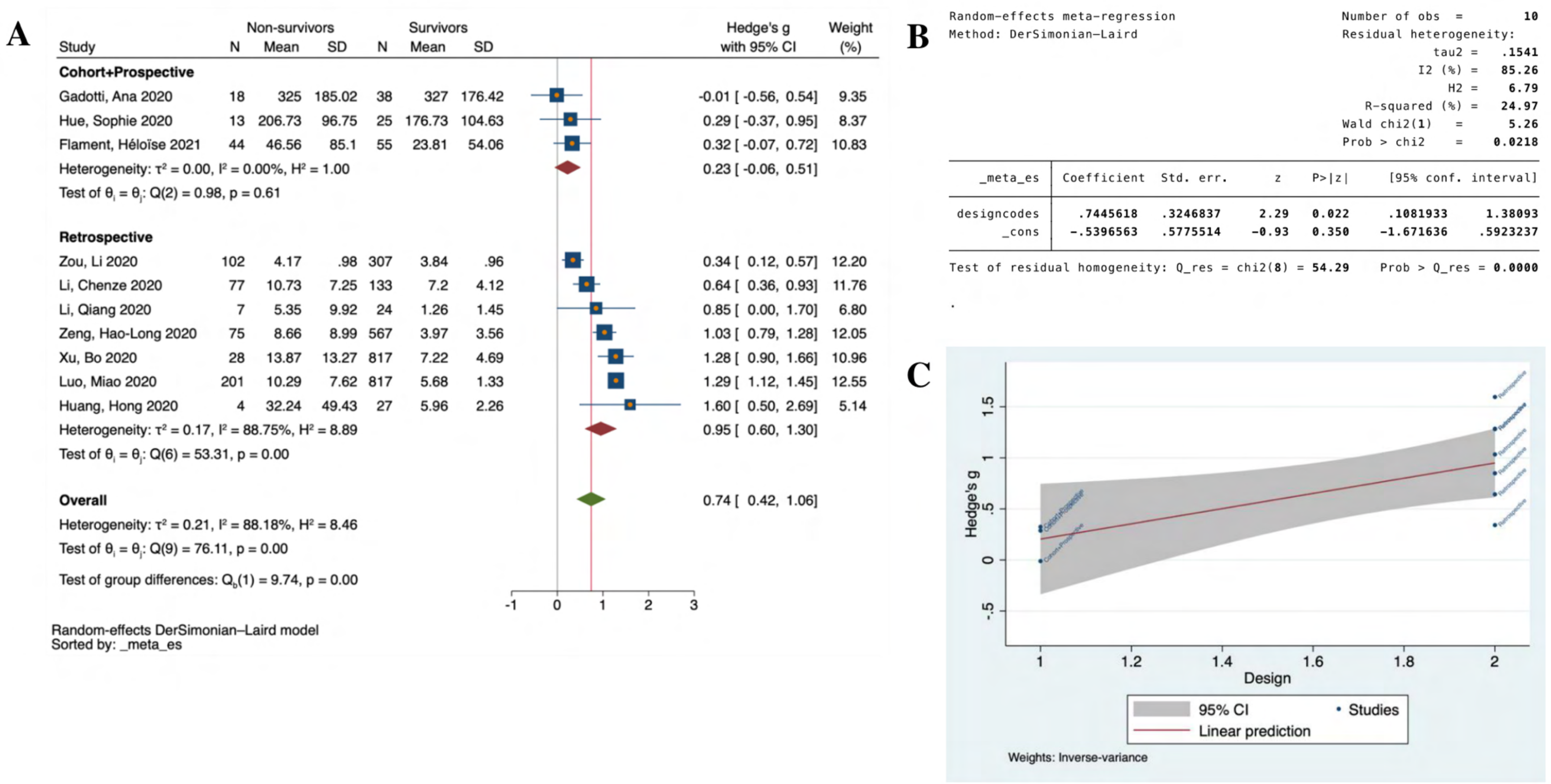

Figure S10.54. Subgroup analysis performed under the moderator (study design) for IL-10 in COVID-19 mortality studies. (A) Subgroup forest plot. The no-effect line is represented at the value of zero. The diamond symbol represents estimated combined effect. (B) Subgroup meta-regression. (C) Subgroup meta-regression bubble plot. Studies are represented as (bubbles). The regression line (red). The horizontal axis represents study design.

Subgroup analysis under moderator (classification protocol) for IL-10 in COVID-19 mortality studies

A

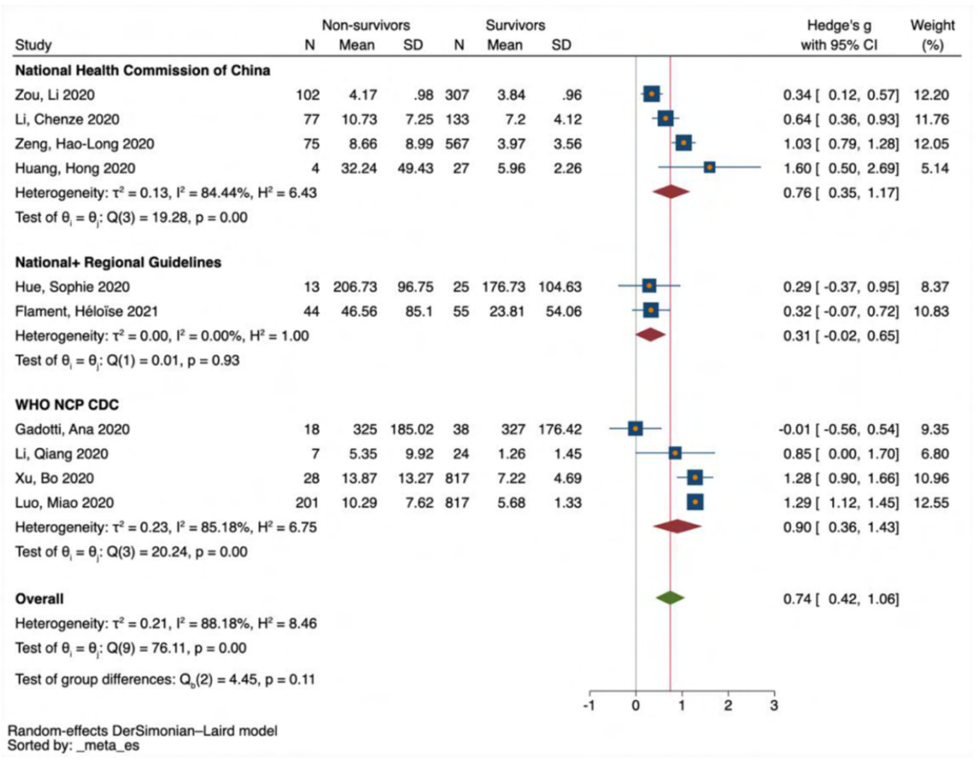

B

```
Random-effects meta-regression                    Number of obs  =        10
Method: DerSimonian-Laird                         Residual heterogeneity:
                                                           tau2 =    .1805
                                                         I2 (%) =    84.31
                                                             H2 =     6.37
                                                  R-squared (%) =    12.13
                                                  Wald chi2(1)   =     0.13
                                                  Prob > chi2    =   0.7166

    _meta_es   Coefficient  Std. err.      z    P>|z|     [95% conf. interval]

protocolcodes     .062528     .172255    0.36   0.717    -.2750856    .4001416
        _cons    .6174322    .3749203    1.65   0.100     -.117398    1.352262

Test of residual homogeneity: Q_res = chi2(8) = 50.99     Prob > Q_res = 0.0000
```

C

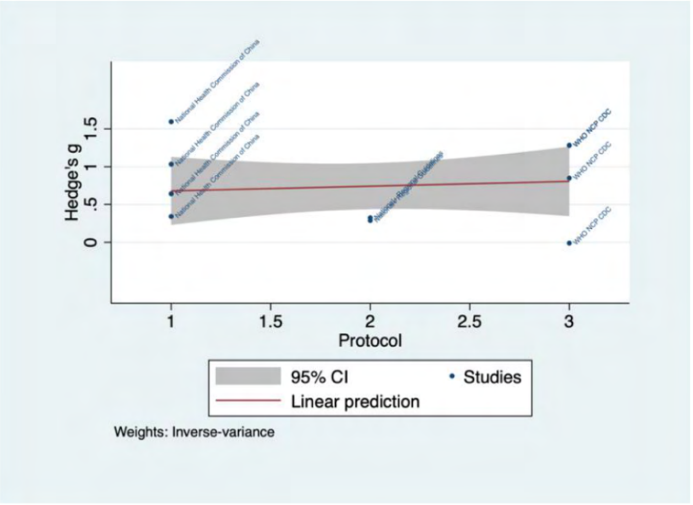

Figure S10.55. Subgroup analysis performed under the moderator (classification protocol) for IL-10 in COVID-19 mortality studies. (A) Subgroup forest plot. The no-effect line is represented at the value of zero. The diamond symbol represents estimated combined effect. (B) Subgroup meta-regression. (C) Subgroup meta-regression bubble plot. Studies are represented as (bubbles). The regression line (red). The horizontal axis represents classification protocols.

Subgroup analysis under moderator (sample acquisition time) for IL-10 in COVID-19 mortality studies

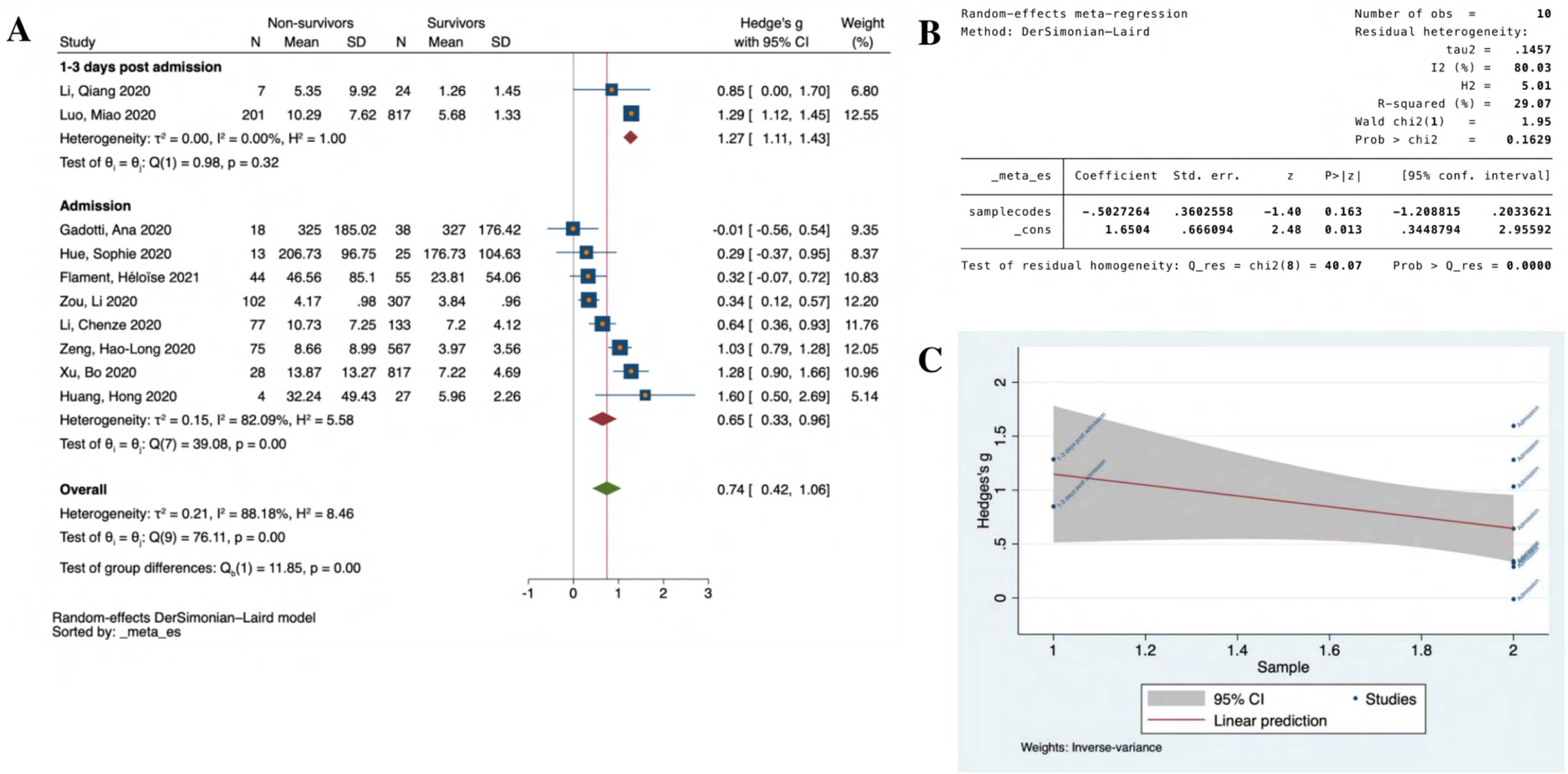

Figure S10.56. Subgroup analysis performed under the moderator (sample acquisition time) for IL-10 in COVID-19 mortality studies. (A) Subgroup forest plot. The no-effect line is represented at the value of zero. The diamond symbol represents estimated combined effect. (B) Subgroup meta-regression. (C) Subgroup meta-regression bubble plot. Studies are represented as (bubbles). The regression line (red). The horizontal axis represents sample acquisition time.

Subgroup analysis under moderator (total male number) for IL-10 in COVID-19 mortality studies

---

A

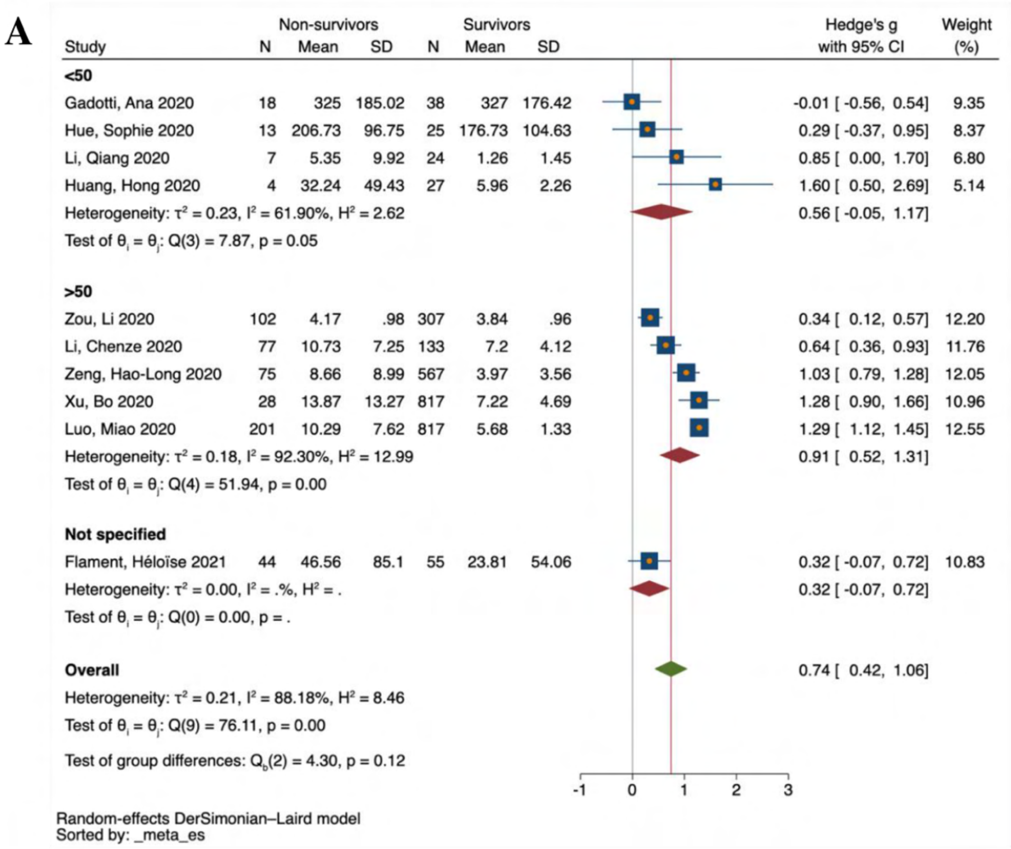

B

```
Random-effects meta-regression                    Number of obs  =        10
Method: DerSimonian–Laird                         Residual heterogeneity:
                                                              tau2 =    .2194
                                                            I2 (%) =    89.49
                                                                H2 =     9.51
                                                     R-squared (%) =     0.00
                                                     Wald chi2(1)  =     0.01
                                                     Prob > chi2   =   0.9104

    _meta_es   Coefficient  Std. err.      z    P>|z|     [95% conf. interval]

   malecodes      .0309549   .2751044   0.11   0.910    -.5082397    .5701496
       _cons      .6851802   .5247339   1.31   0.192    -.3432794     1.71364

Test of residual homogeneity: Q_res = chi2(8) = 76.10     Prob > Q_res = 0.0000
```

C

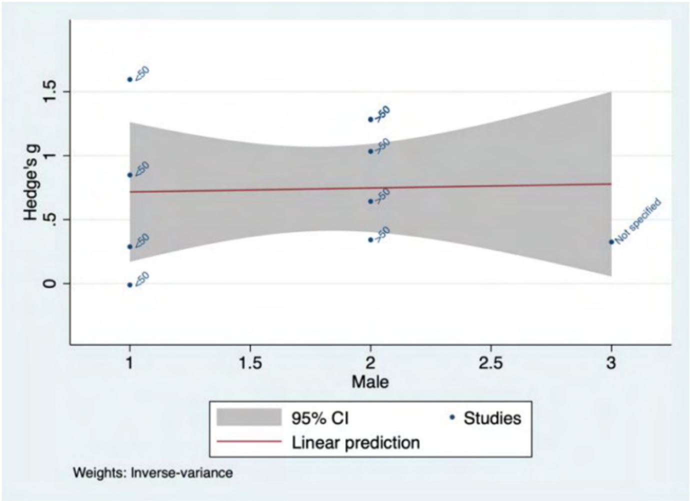

Figure S10.57. Subgroup analysis performed under the moderator (total male number) for IL-10 in COVID-19 mortality studies. (A) Subgroup forest plot. The no-effect line is represented at the value of zero. The diamond symbol represents estimated combined effect. (B) Subgroup meta-regression. (C) Subgroup meta-regression bubble plot. Studies are represented as (bubbles). The regression line (red). The horizontal axis represents total male number.

Subgroup analysis under moderator (total female number) for IL-10 in COVID-19 mortality studies

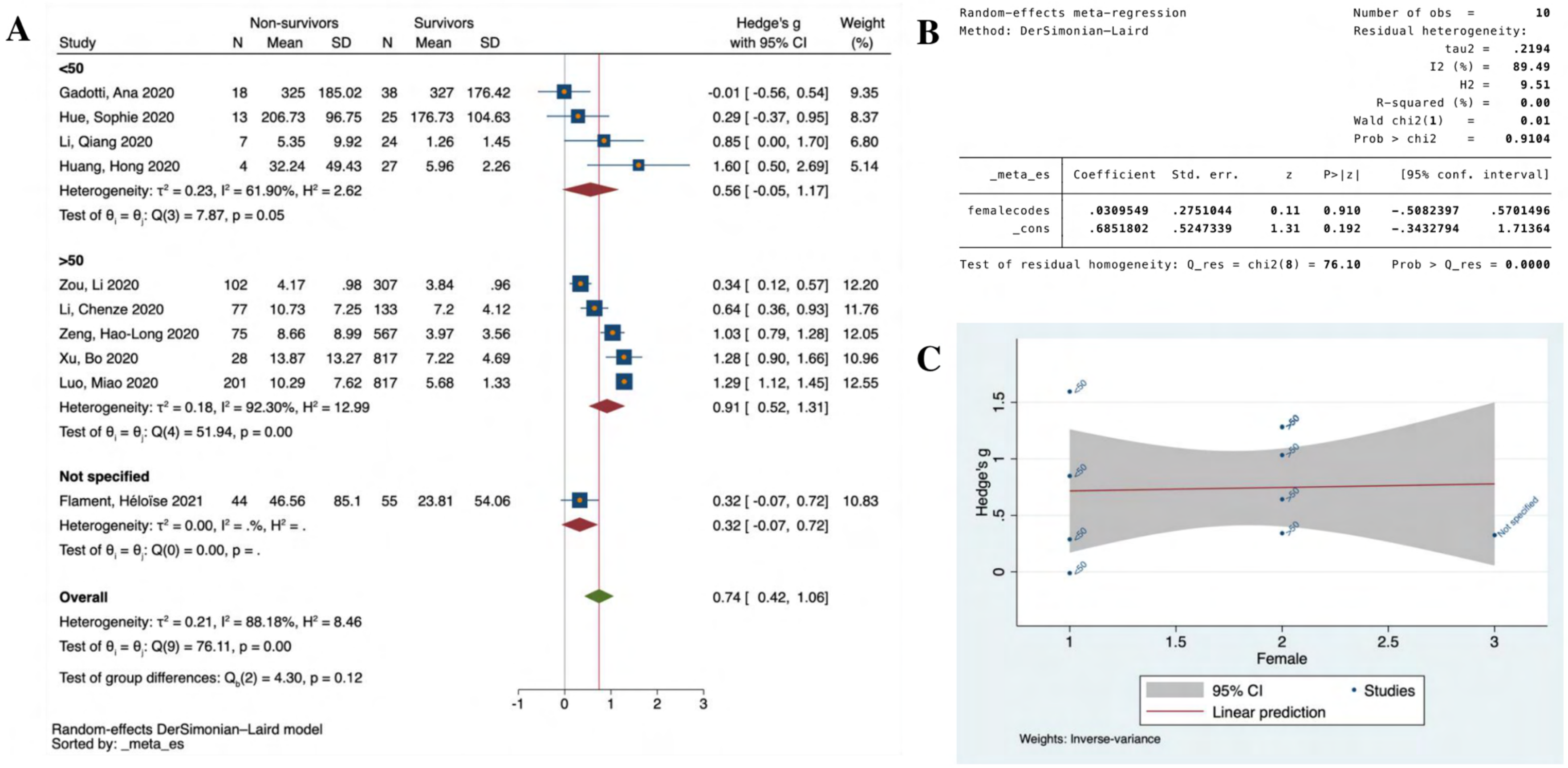

Figure S10.58. Subgroup analysis performed under the moderator (total female number) for IL-10 in COVID-19 mortality studies. (A) Subgroup forest plot. The no-effect line is represented at the value of zero. The diamond symbol represents estimated combined effect. (B) Subgroup meta-regression. (C) Subgroup meta-regression bubble plot. Studies are represented as (bubbles). The regression line (red). The horizontal axis represents total female number.

Subgroup analysis under moderator (mean age) for IL-10 in COVID-19 mortality studies

A

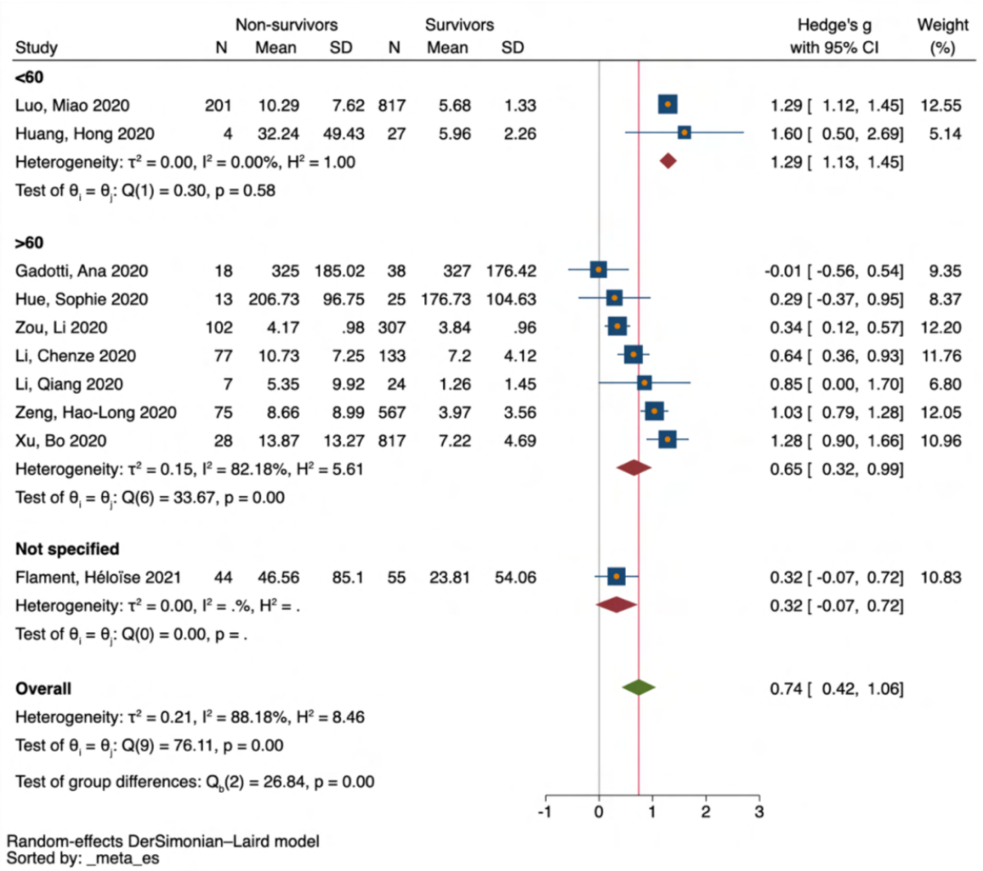

B

```
Random-effects meta-regression                  Number of obs  =         10
Method: DerSimonian-Laird                       Residual heterogeneity:
                                                          tau2 =      .1147
                                                        I2 (%) =      77.17
                                                            H2 =       4.38
                                                 R-squared (%) =      44.16
                                                Wald chi2(1)   =       4.99
                                                Prob > chi2    =     0.0255

------------------------------------------------------------------------------
    _meta_es | Coefficient  Std. err.      z    P>|z|     [95% conf. interval]
-------------+----------------------------------------------------------------
    agecodes |  -.5468169   .2447369    -2.23   0.025    -1.026492   -.0671414
       _cons |   1.800107   .4899828     3.67   0.000     .8397579    2.760455
------------------------------------------------------------------------------
Test of residual homogeneity: Q_res = chi2(8) = 35.03     Prob > Q_res = 0.0000
```

C

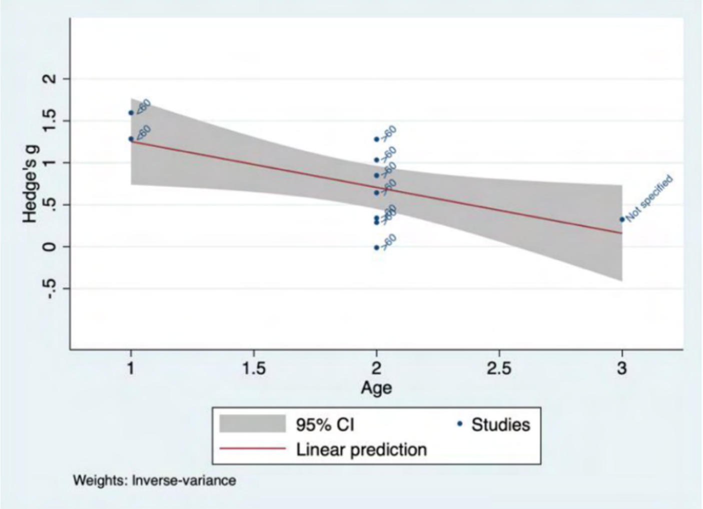

Figure S10.59. Subgroup analysis performed under the moderator (mean age) for IL-10 in COVID-19 mortality studies. (A) Subgroup forest plot. The no-effect line is represented at the value of zero. The diamond symbol represents estimated combined effect. (B) Subgroup meta-regression. (C) Subgroup meta-regression bubble plot. Studies are represented as (bubbles). The regression line (red). The horizontal axis represents mean age.

Subgroup analysis under moderator (test procedure) for IL-10 in COVID-19 mortality studies

---

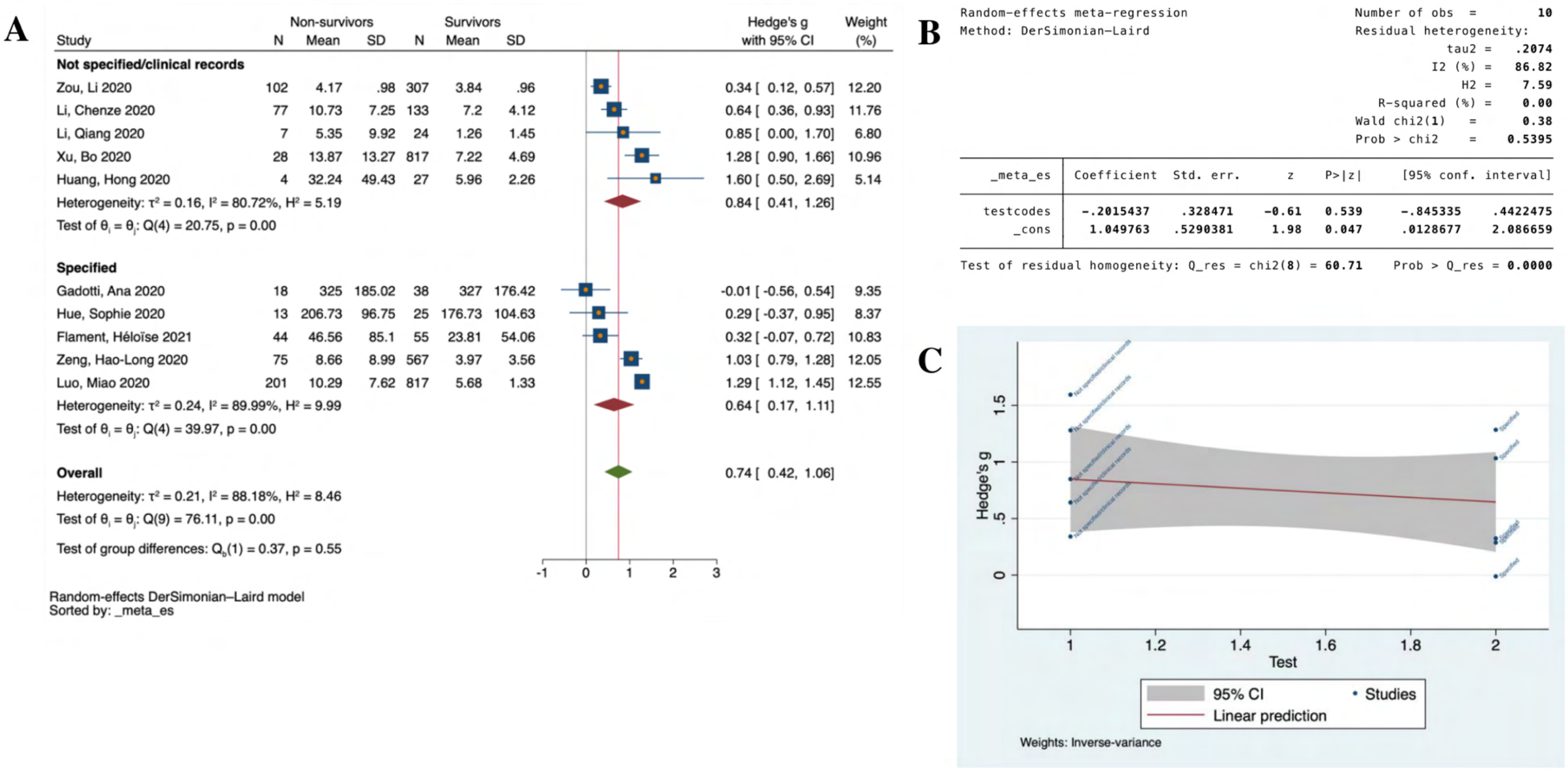

Figure S10.60. Subgroup analysis performed under the moderator (test procedure) for IL-10 in COVID-19 mortality studies. (A) Subgroup forest plot. The no-effect line is represented at the value of zero. The diamond symbol represents estimated combined effect. (B) Subgroup meta-regression. (C) Subgroup meta-regression bubble plot. Studies are represented as (bubbles). The regression line (red). The horizontal axis represents test procedure.

PART XI

INVESTIGATING THE SOURCE OF HETEROGENEITY

SMALL STUDY EFFECT

AND PUBLICATION BIAS

A

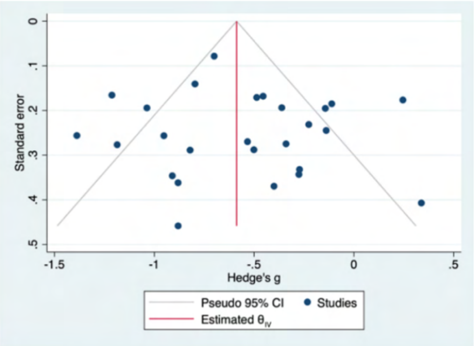

B

```
Regression-based Egger test for small-study effects
Random-effects model
Method: DerSimonian–Laird
Moderators: citycodes

H0: beta1 = 0; no small-study effects
            beta1 =      0.36
      SE of beta1 =     1.073
                z =      0.34
       Prob > |z| =    0.7360
```

```
Regression-based Egger test for small-study effects
Random-effects model
Method: DerSimonian–Laird
Moderators: countrycodes

H0: beta1 = 0; no small-study effects
            beta1 =     -0.27
      SE of beta1 =     0.991
                z =     -0.27
       Prob > |z| =    0.7877
```

```
Regression-based Egger test for small-study effects
Random-effects model
Method: DerSimonian–Laird
Moderators: continentcodes

H0: beta1 = 0; no small-study effects
            beta1 =      0.19
      SE of beta1 =     0.944
                z =      0.20
       Prob > |z| =    0.8430
```

```
Regression-based Egger test for small-study effects
Random-effects model
Method: DerSimonian–Laird
Moderators: designcodes

H0: beta1 = 0; no small-study effects
            beta1 =     -0.02
      SE of beta1 =     0.945
                z =     -0.02
       Prob > |z| =    0.9853
```

```
Regression-based Egger test for small-study effects
Random-effects model
Method: DerSimonian–Laird
Moderators: protocolcodes

H0: beta1 = 0; no small-study effects
            beta1 =     -0.69
      SE of beta1 =     1.076
                z =     -0.64
       Prob > |z| =    0.5226
```

```
Regression-based Egger test for small-study effects
Random-effects model
Method: DerSimonian–Laird
Moderators: samplecodes

H0: beta1 = 0; no small-study effects
            beta1 =      0.07
      SE of beta1 =     1.066
                z =      0.07
       Prob > |z| =    0.9481
```

```
Regression-based Egger test for small-study effects
Random-effects model
Method: DerSimonian–Laird
Moderators: malecodes

H0: beta1 = 0; no small-study effects
            beta1 =     -0.32
      SE of beta1 =     1.148
                z =     -0.28
       Prob > |z| =    0.7817
```

```
Regression-based Egger test for small-study effects
Random-effects model
Method: DerSimonian–Laird
Moderators: femalecodes

H0: beta1 = 0; no small-study effects
            beta1 =      0.24
      SE of beta1 =     1.220
                z =      0.20
       Prob > |z| =    0.8449
```

```
Regression-based Egger test for small-study effects
Random-effects model
Method: DerSimonian–Laird
Moderators: agecodes

H0: beta1 = 0; no small-study effects
            beta1 =      0.16
      SE of beta1 =     1.047
                z =      0.16
       Prob > |z| =    0.8756
```

```
Regression-based Egger test for small-study effects
Random-effects model
Method: DerSimonian–Laird
Moderators: testcodes

H0: beta1 = 0; no small-study effects
            beta1 =      0.29
      SE of beta1 =     1.061
                z =      0.27
       Prob > |z| =    0.7855
```

C

```
Begg's test for small-study effects

Kendall's score =     11.00
    SE of score =    45.369
              z =      0.22
     Prob > |z| =    0.8255
```

Figure 11.1. Analysis of publication bias for CD4 T-cell in COVID-19 severity studies. (A) Standard funnel plot analysis. Observed studies (blue). (B) Regression-based Egger test performed under the moderators (city, country, continent, study design, classification protocol, sample acquisition time, total male number, total female number, mean age, and test procedure). (C) Nonparametric rank correlation (Begg) test.

Standard funnel plot for CD8 severity studies

A

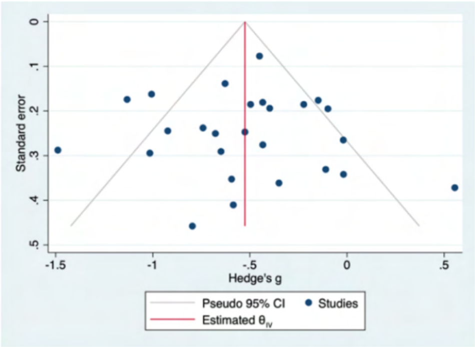

B

```
Regression-based Egger test for small-study effects
Random-effects model
Method: DerSimonian–Laird
Moderators: citycodes

H0: beta1 = 0; no small-study effects
            beta1 =       0.33
      SE of beta1 =      0.945
                z =       0.35
       Prob > |z| =     0.7272
```

```
Regression-based Egger test for small-study effects
Random-effects model
Method: DerSimonian–Laird
Moderators: countrycodes

H0: beta1 = 0; no small-study effects
            beta1 =       0.06
      SE of beta1 =      0.831
                z =       0.07
       Prob > |z| =     0.9421
```

```
Regression-based Egger test for small-study effects
Random-effects model
Method: DerSimonian–Laird
Moderators: continentcodes

H0: beta1 = 0; no small-study effects
            beta1 =      -0.04
      SE of beta1 =      0.783
                z =      -0.05
       Prob > |z| =     0.9611
```

```
Regression-based Egger test for small-study effects
Random-effects model
Method: DerSimonian–Laird
Moderators: designcodes

H0: beta1 = 0; no small-study effects
            beta1 =       0.31
      SE of beta1 =      0.889
                z =       0.35
       Prob > |z| =     0.7272
```

```
Regression-based Egger test for small-study effects
Random-effects model
Method: DerSimonian–Laird
Moderators: samplecodes

H0: beta1 = 0; no small-study effects
            beta1 =       0.27
      SE of beta1 =      0.866
                z =       0.32
       Prob > |z| =     0.7519
```

```
Regression-based Egger test for small-study effects
Random-effects model
Method: DerSimonian–Laird
Moderators: protocolcodes

H0: beta1 = 0; no small-study effects
            beta1 =       0.48
      SE of beta1 =      0.934
                z =       0.51
       Prob > |z| =     0.6069
```

```
Regression-based Egger test for small-study effects
Random-effects model
Method: DerSimonian–Laird
Moderators: malecodes

H0: beta1 = 0; no small-study effects
            beta1 =      -0.38
      SE of beta1 =      0.938
                z =      -0.40
       Prob > |z| =     0.6863
```

```
Regression-based Egger test for small-study effects
Random-effects model
Method: DerSimonian–Laird
Moderators: femalecodes

H0: beta1 = 0; no small-study effects
            beta1 =      -0.07
      SE of beta1 =      1.040
                z =      -0.07
       Prob > |z| =     0.9451
```

```
Regression-based Egger test for small-study effects
Random-effects model
Method: DerSimonian–Laird
Moderators: agecodes

H0: beta1 = 0; no small-study effects
            beta1 =       0.48
      SE of beta1 =      0.913
                z =       0.53
       Prob > |z| =     0.5973
```

```
Regression-based Egger test for small-study effects
Random-effects model
Method: DerSimonian–Laird
Moderators: testcodes

H0: beta1 = 0; no small-study effects
            beta1 =       0.74
      SE of beta1 =      0.894
                z =       0.83
       Prob > |z| =     0.4053
```

C

```
Begg's test for small-study effects

Kendall's score =     13.00
    SE of score =    45.369
              z =      0.26
     Prob > |z| =    0.7914
```

Figure 11.2. Analysis of publication bias for CD8 T-cell in COVID-19 severity studies. (A) Standard funnel plot analysis. Observed studies (blue). (B) Regression-based Egger test performed under the moderators (city, country, continent, study design, classification protocol, sample acquisition time, total male number, total female number, mean age, and test procedure). (C) Nonparametric rank correlation (Begg) test.

Standard funnel plot for IL-10 severity studies

A

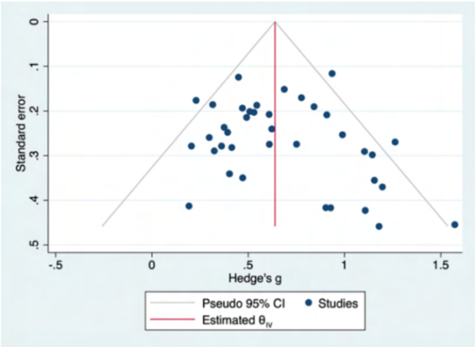

B

```
Regression-based Egger test for small-study effects
Random-effects model
Method: DerSimonian-Laird
Moderators: citycodes

H0: beta1 = 0; no small-study effects
            beta1 =      1.44
      SE of beta1 =     0.620
                z =      2.32
       Prob > |z| =    0.0204
```

```
Regression-based Egger test for small-study effects
Random-effects model
Method: DerSimonian-Laird
Moderators: countrycodes

H0: beta1 = 0; no small-study effects
            beta1 =      1.07
      SE of beta1 =     0.630
                z =      1.70
       Prob > |z| =    0.0888
```

```
Regression-based Egger test for small-study effects
Random-effects model
Method: DerSimonian-Laird
Moderators: continentcodes

H0: beta1 = 0; no small-study effects
            beta1 =      1.10
      SE of beta1 =     0.616
                z =      1.78
       Prob > |z| =    0.0746
```

```
Regression-based Egger test for small-study effects
Random-effects model
Method: DerSimonian-Laird
Moderators: designcodes

H0: beta1 = 0; no small-study effects
            beta1 =      1.15
      SE of beta1 =     0.626
                z =      1.84
       Prob > |z| =    0.0660
```

```
Regression-based Egger test for small-study effects
Random-effects model
Method: DerSimonian-Laird
Moderators: protocolcodes

H0: beta1 = 0; no small-study effects
            beta1 =      1.07
      SE of beta1 =     0.618
                z =      1.73
       Prob > |z| =    0.0836
```

```
Regression-based Egger test for small-study effects
Random-effects model
Method: DerSimonian-Laird
Moderators: samplecodes

H0: beta1 = 0; no small-study effects
            beta1 =      1.07
      SE of beta1 =     0.628
                z =      1.71
       Prob > |z| =    0.0875
```

```
Regression-based Egger test for small-study effects
Random-effects model
Method: DerSimonian-Laird
Moderators: malecodes

H0: beta1 = 0; no small-study effects
            beta1 =      1.62
      SE of beta1 =     0.733
                z =      2.21
       Prob > |z| =    0.0268
```

```
Regression-based Egger test for small-study effects
Random-effects model
Method: DerSimonian-Laird
Moderators: femalecodes

H0: beta1 = 0; no small-study effects
            beta1 =      1.43
      SE of beta1 =     0.721
                z =      1.99
       Prob > |z| =    0.0468
```

```
Regression-based Egger test for small-study effects
Random-effects model
Method: DerSimonian-Laird
Moderators: agecodes

H0: beta1 = 0; no small-study effects
            beta1 =      1.13
      SE of beta1 =     0.627
                z =      1.80
       Prob > |z| =    0.0723
```

```
Regression-based Egger test for small-study effects
Random-effects model
Method: DerSimonian-Laird
Moderators: testcodes

H0: beta1 = 0; no small-study effects
            beta1 =      1.32
      SE of beta1 =     0.626
                z =      2.10
       Prob > |z| =    0.0354
```

C

```
Begg's test for small-study effects

Kendall's score =    145.00
    SE of score =    79.542
              z =      1.81
     Prob > |z| =    0.0702
```

Figure 11.3. Analysis of publication bias for IL-10 in COVID-19 severity studies. (A) Standard funnel plot analysis. Observed studies (blue). (B) Regression-based Egger test performed under the moderators (city, country, continent, study design, classification protocol, sample acquisition time, total male number, total female number, mean age, and test procedure). (C) Nonparametric rank correlation (Begg) test.

Standard funnel plot for CD4 mortality studies

A

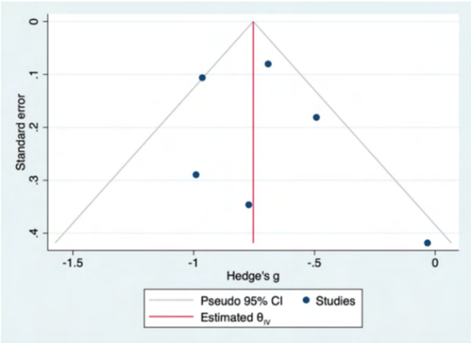

B

```
Regression-based Egger test for small-study effects
Random-effects model
Method: DerSimonian–Laird
Moderators: citycodes

H0: beta1 = 0; no small-study effects
          beta1 =      1.06
    SE of beta1 =     1.094
              z =      0.97
     Prob > |z| =    0.3328
```

```
Regression-based Egger test for small-study effects
Random-effects model
Method: DerSimonian–Laird
Moderators: countrycodes

H0: beta1 = 0; no small-study effects
          beta1 =      0.77
    SE of beta1 =     0.911
              z =      0.85
     Prob > |z| =    0.3963
```

```
Regression-based Egger test for small-study effects
Random-effects model
Method: DerSimonian–Laird
Moderators: continentcodes

H0: beta1 = 0; no small-study effects
          beta1 =      0.77
    SE of beta1 =     0.911
              z =      0.85
     Prob > |z| =    0.3963
```

```
Regression-based Egger test for small-study effects
Random-effects model
Method: DerSimonian–Laird
Moderators: designcodes

H0: beta1 = 0; no small-study effects
          beta1 =      1.28
    SE of beta1 =     1.261
              z =      1.01
     Prob > |z| =    0.3117
```

```
Regression-based Egger test for small-study effects
Random-effects model
Method: DerSimonian–Laird
Moderators: protocolcodes

H0: beta1 = 0; no small-study effects
          beta1 =      1.23
    SE of beta1 =     0.802
              z =      1.53
     Prob > |z| =    0.1254
```

```
Regression-based Egger test for small-study effects
Random-effects model
Method: DerSimonian–Laird
Moderators: samplecodes

H0: beta1 = 0; no small-study effects
          beta1 =      1.20
    SE of beta1 =     1.074
              z =      1.11
     Prob > |z| =    0.2652
```

```
Regression-based Egger test for small-study effects
Random-effects model
Method: DerSimonian–Laird
Moderators: malecodes

H0: beta1 = 0; no small-study effects
          beta1 =      0.19
    SE of beta1 =     1.823
              z =      0.10
     Prob > |z| =    0.9183
```

```
Regression-based Egger test for small-study effects
Random-effects model
Method: DerSimonian–Laird
Moderators: femalecodes

H0: beta1 = 0; no small-study effects
          beta1 =      0.19
    SE of beta1 =     1.823
              z =      0.10
     Prob > |z| =    0.9183
```

```
Regression-based Egger test for small-study effects
Random-effects model
Method: DerSimonian–Laird
Moderators: agecodes

H0: beta1 = 0; no small-study effects
          beta1 =      1.09
    SE of beta1 =     0.934
              z =      1.17
     Prob > |z| =    0.2413
```

```
Regression-based Egger test for small-study effects
Random-effects model
Method: DerSimonian–Laird
Moderators: testcodes

H0: beta1 = 0; no small-study effects
          beta1 =      0.36
    SE of beta1 =     1.323
              z =      0.27
     Prob > |z| =    0.7849
```

C

```
Begg's test for small-study effects

Kendall's score =      5.00
    SE of score =     5.323
              z =      0.75
     Prob > |z| =    0.4524
```

Figure 11.4. Analysis of publication bias for CD4 T-cell in COVID-19 mortality studies. (A) Standard funnel plot analysis. Observed studies (blue). (B) Regression-based Egger test performed under the moderators (city, country, continent, study design, classification protocol, sample acquisition time, total male number, total female number, mean age, and test procedure).

Standard funnel plot for CD8 mortality studies

A

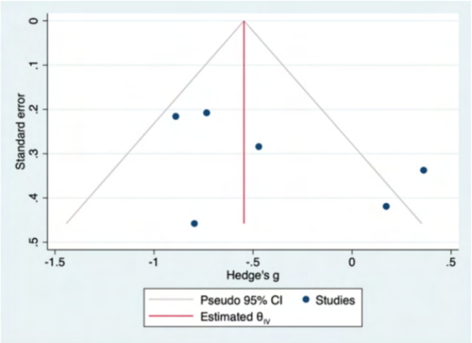

B

```
Regression-based Egger test for small-study effects
Random-effects model
Method: DerSimonian-Laird
Moderators: citycodes

H0: beta1 = 0; no small-study effects
            beta1 =     -2.64
      SE of beta1 =     4.003
                z =     -0.66
      Prob > |z| =    0.5092
```

```
Regression-based Egger test for small-study effects
Random-effects model
Method: DerSimonian-Laird
Moderators: countrycodes

H0: beta1 = 0; no small-study effects
            beta1 =      2.11
      SE of beta1 =     2.677
                z =      0.79
      Prob > |z| =    0.4305
```

```
Regression-based Egger test for small-study effects
Random-effects model
Method: DerSimonian-Laird
Moderators: continentcodes

H0: beta1 = 0; no small-study effects
            beta1 =      2.11
      SE of beta1 =     2.677
                z =      0.79
      Prob > |z| =    0.4305
```

```
Regression-based Egger test for small-study effects
Random-effects model
Method: DerSimonian-Laird
Moderators: designcodes

H0: beta1 = 0; no small-study effects
            beta1 =      2.19
      SE of beta1 =     1.591
                z =      1.38
      Prob > |z| =    0.1690
```

```
Regression-based Egger test for small-study effects
Random-effects model
Method: DerSimonian-Laird
Moderators: protocolcodes

H0: beta1 = 0; no small-study effects
            beta1 =      2.29
      SE of beta1 =     2.488
                z =      0.92
      Prob > |z| =    0.3569
```

```
Regression-based Egger test for small-study effects
Random-effects model
Method: DerSimonian-Laird
Moderators: samplecodes

H0: beta1 = 0; no small-study effects
            beta1 =      2.60
      SE of beta1 =     2.353
                z =      1.11
      Prob > |z| =    0.2686
```

```
Regression-based Egger test for small-study effects
Random-effects model
Method: DerSimonian-Laird
Moderators: malecodes

H0: beta1 = 0; no small-study effects
            beta1 =      0.35
      SE of beta1 =     1.800
                z =      0.20
      Prob > |z| =    0.8439
```

```
Regression-based Egger test for small-study effects
Random-effects model
Method: DerSimonian-Laird
Moderators: femalecodes

H0: beta1 = 0; no small-study effects
            beta1 =      0.35
      SE of beta1 =     1.800
                z =      0.20
      Prob > |z| =    0.8439
```

```
Regression-based Egger test for small-study effects
Random-effects model
Method: DerSimonian-Laird
Moderators: agecodes

H0: beta1 = 0; no small-study effects
            beta1 =      4.05
      SE of beta1 =     2.962
                z =      1.37
      Prob > |z| =    0.1713
```

```
Regression-based Egger test for small-study effects
Random-effects model
Method: DerSimonian-Laird
Moderators: testcodes

H0: beta1 = 0; no small-study effects
            beta1 =      2.53
      SE of beta1 =     2.337
                z =      1.08
      Prob > |z| =    0.2782
```

C

```
Begg's test for small-study effects

Kendall's score =      5.00
    SE of score =     5.323
              z =      0.75
     Prob > |z| =    0.4524
```

Figure 11.5. Analysis of publication bias for CD8 T-cell in COVID-19 mortality studies. (A) Standard funnel plot analysis. Observed studies (blue). (B) Regression-based Egger test performed under the moderators (city, country, continent, study design, classification protocol, sample acquisition time, total male number, total female number, mean age, and test procedure). (C) Nonparametric rank correlation (Begg) test.

Standard funnel plot for IL-10 mortality studies

A

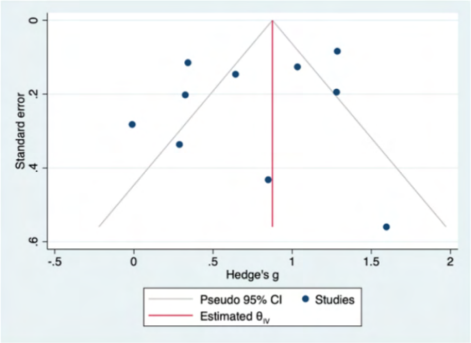

B

```
Regression-based Egger test for small-study effects
Random-effects model
Method: DerSimonian-Laird
Moderators: citycodes

H0: beta1 = 0; no small-study effects
          beta1 =      1.67
    SE of beta1 =     1.453
              z =      1.15
     Prob > |z| =    0.2490
```

```
Regression-based Egger test for small-study effects
Random-effects model
Method: DerSimonian-Laird
Moderators: countrycodes

H0: beta1 = 0; no small-study effects
          beta1 =      0.81
    SE of beta1 =     1.282
              z =      0.63
     Prob > |z| =    0.5284
```

```
Regression-based Egger test for small-study effects
Random-effects model
Method: DerSimonian-Laird
Moderators: continentcodes

H0: beta1 = 0; no small-study effects
          beta1 =      0.81
    SE of beta1 =     1.282
              z =      0.63
     Prob > |z| =    0.5284
```

```
Regression-based Egger test for small-study effects
Random-effects model
Method: DerSimonian-Laird
Moderators: designcodes

H0: beta1 = 0; no small-study effects
          beta1 =      0.81
    SE of beta1 =     1.282
              z =      0.63
     Prob > |z| =    0.5284
```

```
Regression-based Egger test for small-study effects
Random-effects model
Method: DerSimonian-Laird
Moderators: protocolcodes

H0: beta1 = 0; no small-study effects
          beta1 =     -0.10
    SE of beta1 =     1.256
              z =     -0.08
     Prob > |z| =    0.9372
```

```
Regression-based Egger test for small-study effects
Random-effects model
Method: DerSimonian-Laird
Moderators: samplecodes

H0: beta1 = 0; no small-study effects
          beta1 =     -0.01
    SE of beta1 =     1.206
              z =     -0.01
     Prob > |z| =    0.9917
```

```
Regression-based Egger test for small-study effects
Random-effects model
Method: DerSimonian-Laird
Moderators: malecodes

H0: beta1 = 0; no small-study effects
          beta1 =      0.27
    SE of beta1 =     1.769
              z =      0.15
     Prob > |z| =    0.8793
```

```
Regression-based Egger test for small-study effects
Random-effects model
Method: DerSimonian-Laird
Moderators: femalecodes

H0: beta1 = 0; no small-study effects
          beta1 =      0.27
    SE of beta1 =     1.769
              z =      0.15
     Prob > |z| =    0.8793
```

```
Regression-based Egger test for small-study effects
Random-effects model
Method: DerSimonian-Laird
Moderators: agecodes

H0: beta1 = 0; no small-study effects
          beta1 =     -0.14
    SE of beta1 =     1.127
              z =     -0.12
     Prob > |z| =    0.9044
```

```
Regression-based Egger test for small-study effects
Random-effects model
Method: DerSimonian-Laird
Moderators: testcodes

H0: beta1 = 0; no small-study effects
          beta1 =     -0.10
    SE of beta1 =     1.337
              z =     -0.08
     Prob > |z| =    0.9398
```

C

```
Begg's test for small-study effects

Kendall's score =     -3.00
   SE of score =    11.180
             z =     -0.36
    Prob > |z| =    0.8580
```

Figure 11.6. Analysis of publication bias for IL-10 in COVID-19 mortality studies. (A) Standard funnel plot analysis. Observed studies (blue). (B) Regression-based Egger test performed under the moderators (city, country, continent, study design, classification protocol, sample acquisition time, total male number, total female number, mean age, and test procedure). (C) Nonparametric rank correlation (Begg) test.

PART XII

THE LEAVE-ONE-OUT SENSITIVITY TEST

The Leave-One-Out sensitivity test for CD4 T-cells in COVID-19 severity studies

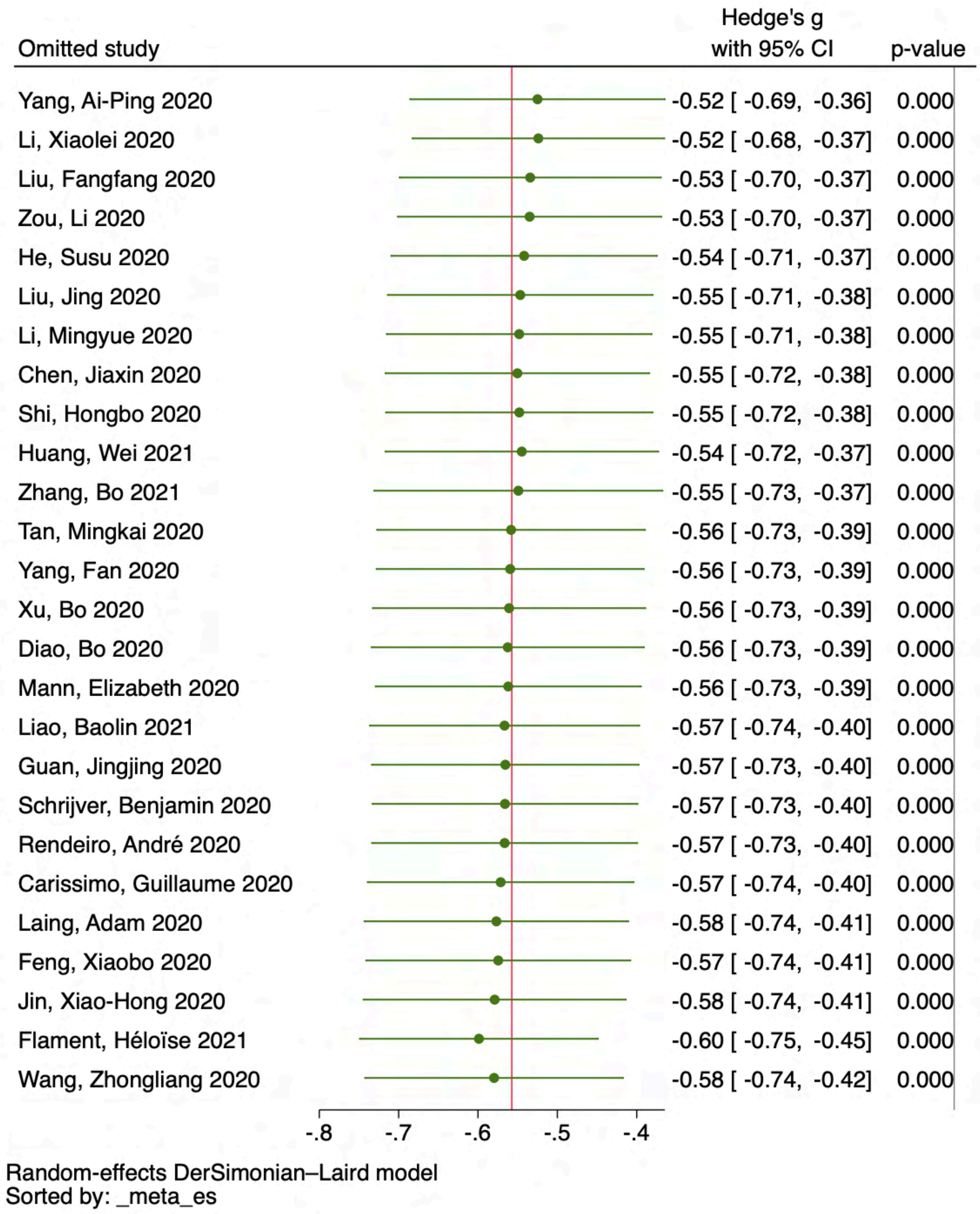

Figure S12.1. The Leave-One-Out sensitivity test for CD4 T-cell in COVID-19 severity studies. Given name study is omitted.

The Leave-One-Out sensitivity test for CD8 T-cells in COVID-19 severity studies

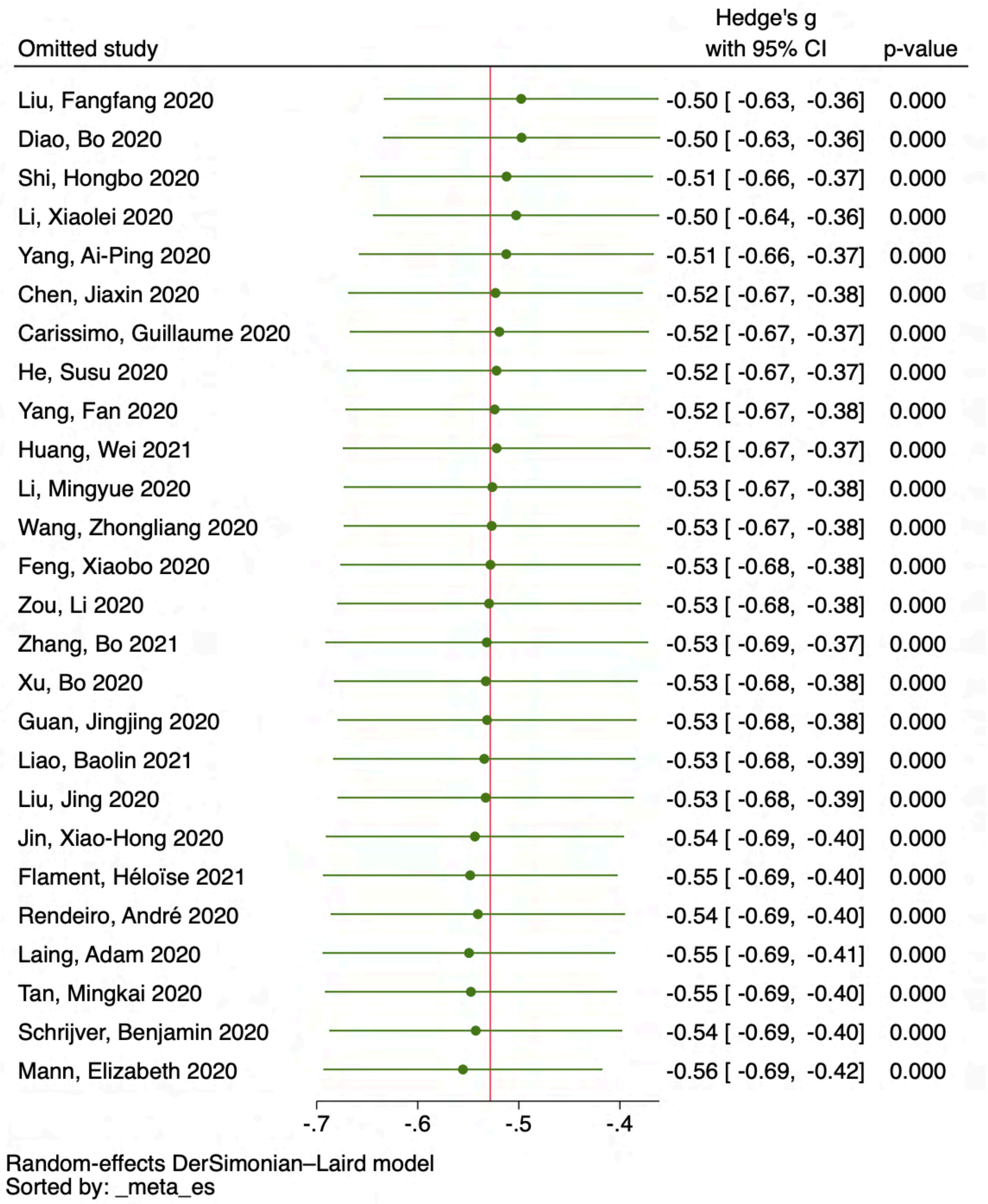

Figure S12.2. The Leave-One-Out sensitivity test for CD4 T-cell in COVID-19 severity studies. Given name study is omitted.

The Leave-One-Out sensitivity test for IL-10 in COVID-19 severity studies

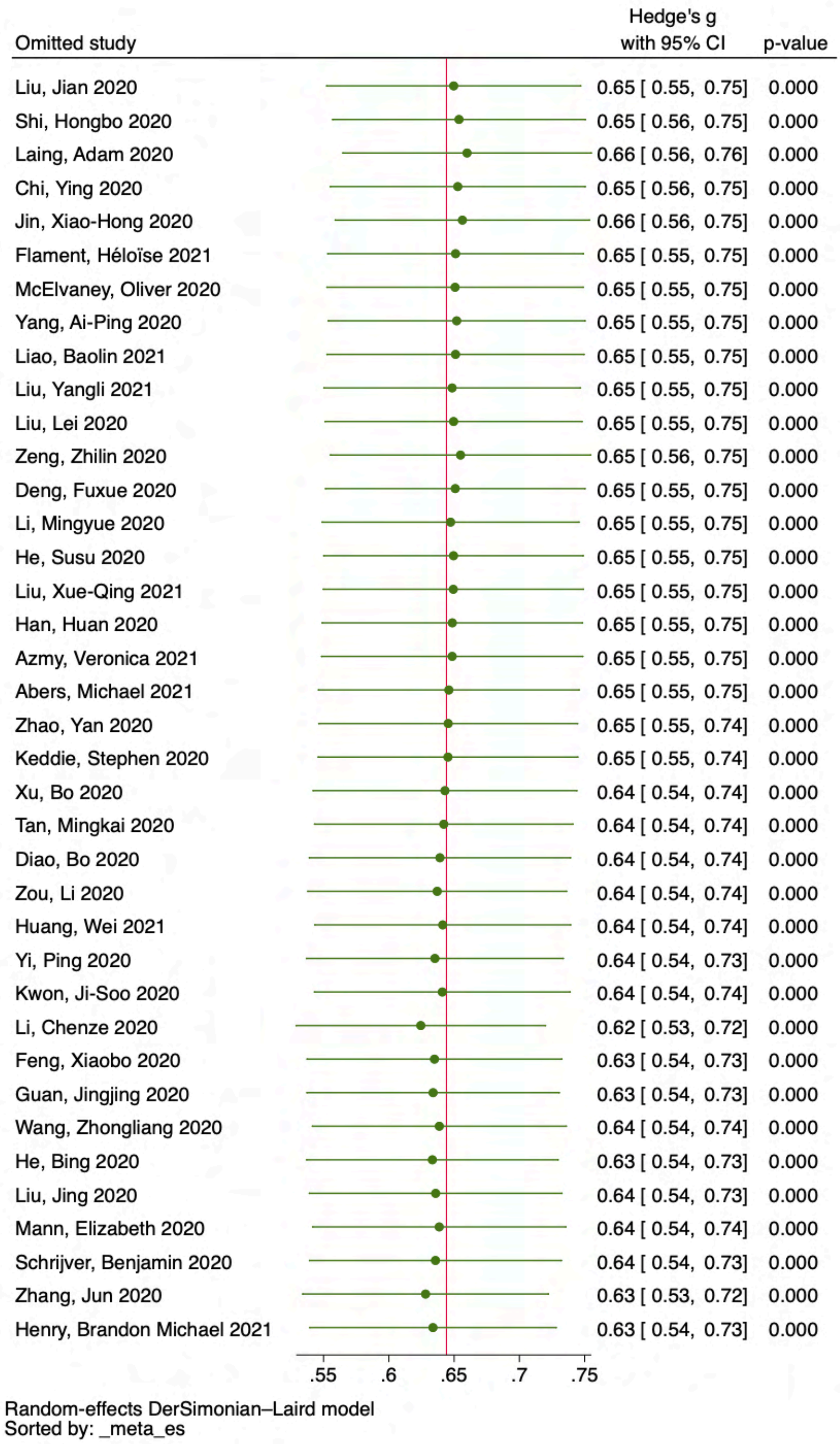

Figure S12.3. The Leave-One-Out sensitivity test for IL-10 in COVID-19 severity studies. Given name study is omitted.

The Leave-One-Out sensitivity test for CD4 T-cells in COVID-19 mortality studies

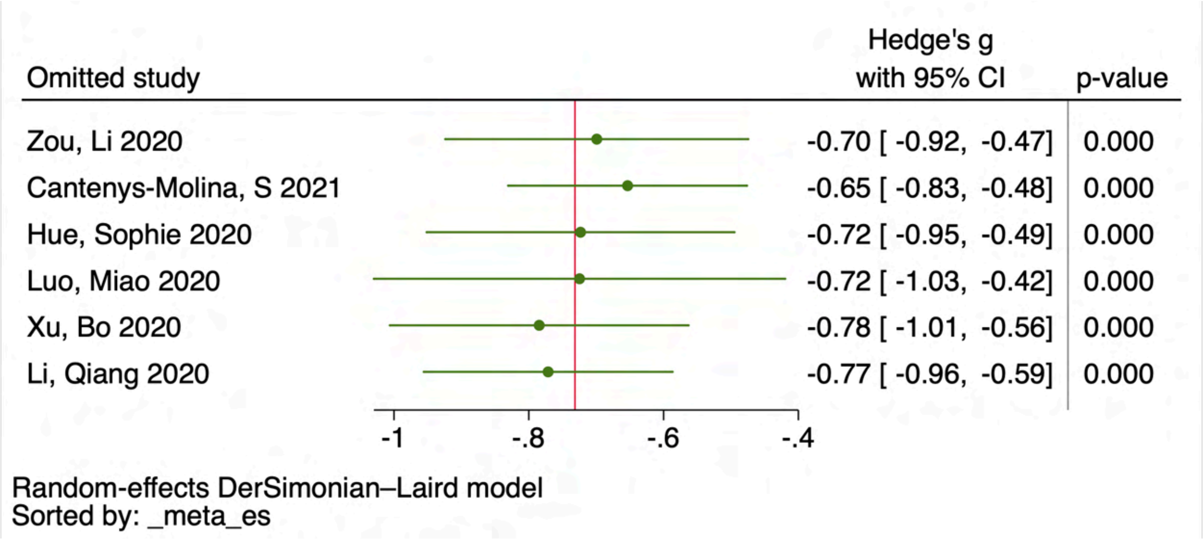

Figure 12.4. The Leave-One-Out sensitivity test for CD4 T-cells in COVID-19 mortality studies. Given name study is omitted.

The Leave-One-Out sensitivity test for CD8 T-cells in COVID-19 mortality studies

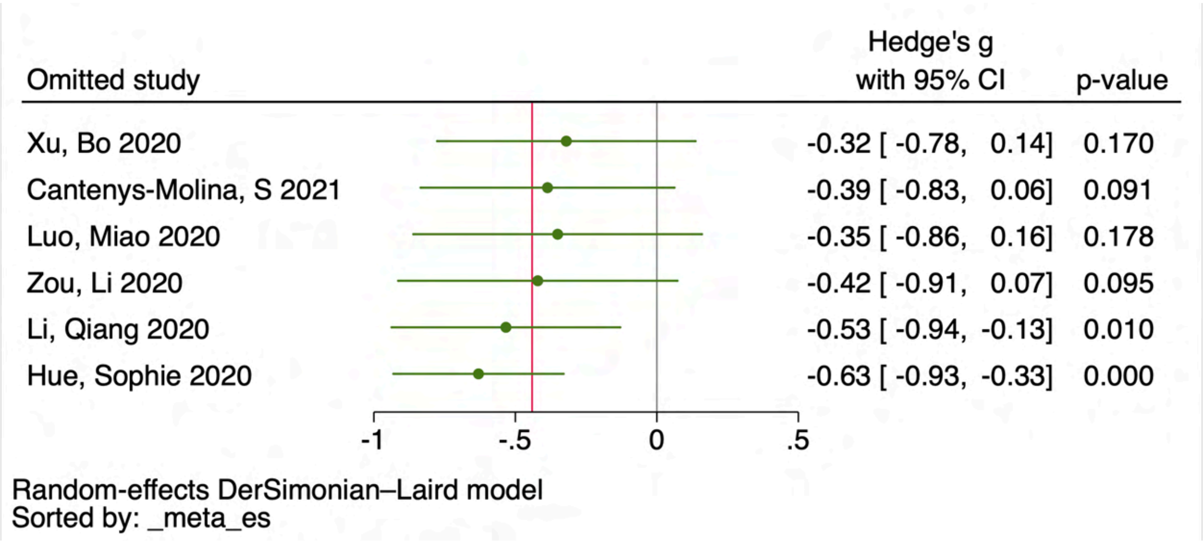

Figure 12.5. The Leave-One-Out sensitivity test for CD8 T-cells in COVID-19 mortality studies. Given name study is omitted.

The Leave-One-Out sensitivity test for IL-10 in COVID-19 mortality studies

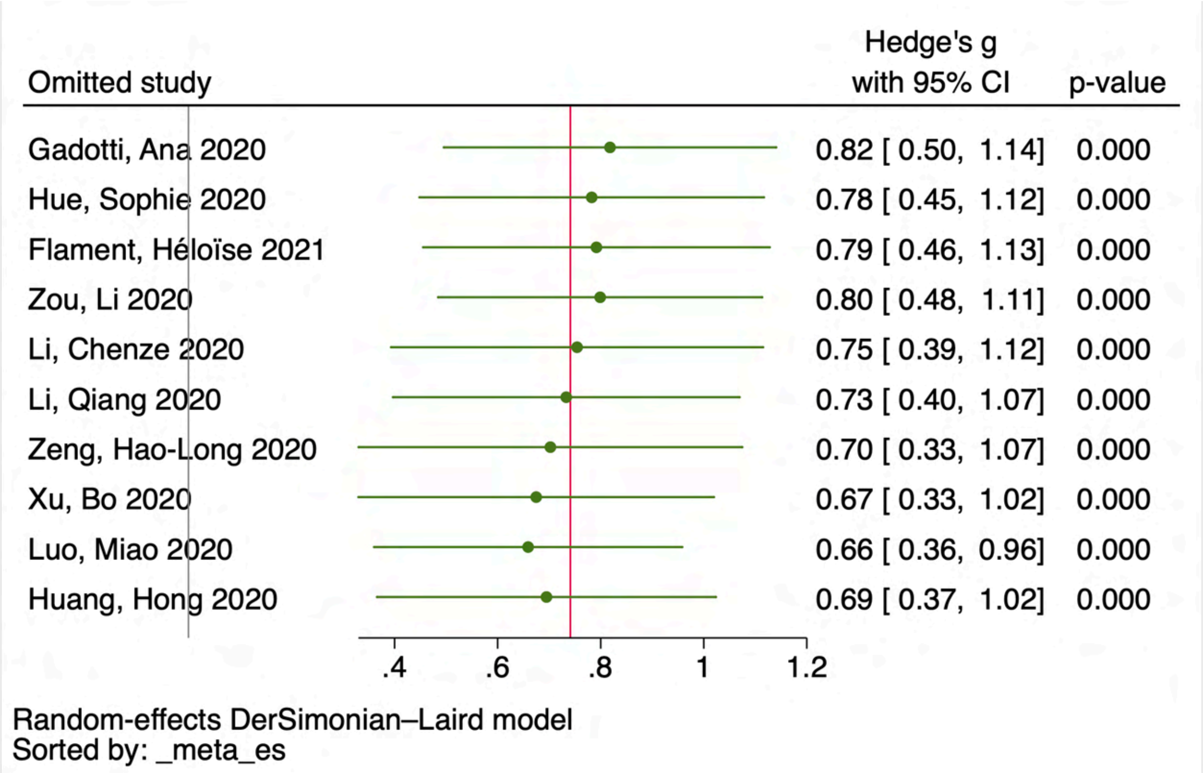

Table S12.6. The Leave-One-Out sensitivity test for IL-10 in COVID-19 mortality studies. Given name study is omitted.
